# Diagnosis and management of cystinosis: systematic review for a clinical practice guideline

**DOI:** 10.1186/s13023-025-03974-z

**Published:** 2025-08-28

**Authors:** Dominic Ledinger, Barbara Nussbaumer-Streit, Brigitte Piso, Andreea Dobrescu, Arianna Gadinger, Irma Klerings, Katharina Hohenfellner, Isolde Sommer

**Affiliations:** 1 Department for Evidence-based Medicine and Evaluation, University for Continuing Education Krems, Krems, Austria; 2 Department of Pediatrics, Pediatric Nephrology, RoMed Kliniken, Rosenheim, Germany

**Keywords:** Cystinosis, Nephropathic cystinosis, Orphan disease, Genetic autosomal recessive disorder, Multisystemic disease, Systematic review, Guideline development

## Abstract

**Background:**

Cystinosis is a rare genetic disorder, with the majority of patients suffering from infantile nephropathic cystinosis, the most severe form. If left untreated, cystinosis causes serious morbidity, initially through progressive kidney and eye disease, followed by systemic and multiorgan involvement, ultimately leading to premature death. In this systematic review, we summarize the evidence for cystinosis to support the development of an evidence-based clinical practice guideline (SELECT - S3 guideline for cystinosis).

**Methods:**

We searched MEDLINE, Embase, CENTRAL, CINAHL, and other databases for relevant studies published from 1980 onward. We screened literature dually and independently for eligibility. Primary researchers extracted data, rated the risk of bias of included studies, and rated the certainty of the evidence (CoE). Secondary researchers reviewed for completeness and accuracy. We applied a staggered approach, prioritizing available controlled studies and synthesizing results narratively.

**Results:**

We considered 56 studies in our synthesis and assessed findings relevant to 17 of 31 key questions. We identified evidence for 62 of 213 outcomes. Fifty-two outcomes had very low CoE, three low, four moderate, and three high. The moderate and high CoE findings came from indirect comparisons (other chronic multi-organ diseases). There was low evidence that delayed-release cysteamine therapy makes little to no difference on cystine levels compared to immediate-release cysteamine therapy; however, delayed-release cysteamine was associated with a slight increase in adverse events. Starting systemic cysteamine treatment early likely improves renal function compared to a later start. Most included studies lacked a control group, had a high risk of bias, and had a small sample size.

**Conclusion:**

Evidence informing the diagnosis and management of cystinosis is limited, with most findings rated as very low certainty. Few direct comparisons involving only individuals with cystinosis yielded low certainty findings, while moderate to high certainty evidence was found only in indirect comparisons. These findings underscore a critical challenge in managing cystinosis: the balancing act of clinical decision-making in the context of lacking evidence. Nonetheless, this systematic review synthesized the best available data for a clinical practice guideline and highlighted specific areas where future research could strengthen the evidence base.

**Supplementary Information:**

The online version contains supplementary material available at 10.1186/s13023-025-03974-z.

## Introduction

Cystinosis is a rare genetic disorder with an estimated incidence of 1:100,000 to 1:200,000 [1]. This autosomal recessive lysosomal storage disease is characterized by accumulation and crystallization of cystine. Cystine transport out of the lysosomal compartment is disrupted due to a defective cystinosin transporter function [2, 3]. Consequently, cystine accumulates in all organs and tissues and causes a multisystemic disease [4].

Cystinosis (ORPHA 213) has three main classifications: infantile nephropathic cystinosis (INC; ORPHA 411629), juvenile (intermediate; ORPHA 411634), and adult (non-nephropathic ocular; ORPHA 411641) cystinosis. The majority of patients (95%) suffer from infantile nephropathic cystinosis, the most severe form [3]. This condition presents between 12 and 24 months of life with symptoms such as excessive urination and electrolyte loss, vomiting, acidosis, and failure to thrive. If untreated, children will likely experience renal failure within their first decade of life [5].

An early diagnosis and timely initiation of systemic treatment can prevent or delay pathological manifestations and progression of disease. Diagnostic tests for suspected pre-symptomatic or symptomatic individuals include the quantification of intracellular cystine levels within white blood cells, molecular genetic testing, and identification of corneal cystine crystals [6, 7]. Currently, the only specific treatment for cystinosis is cysteamine, which depletes cells of cystine [8, 9].

Due to the complexity and rarity of the disease, management is challenging. Clinical practice guidelines help clinicians and patients make evidence-based decisions and are especially relevant for rare disease treatment [10]. The German Society for Pediatric Nephrology has joined forces with international experts in the field and patient support groups to develop the first evidence-based clinical practice guideline (SELECT - S3 guideline for cystinosis) for cystinosis [11]. To date, analyses and syntheses of existing knowledge surrounding the diagnosis and management of cystinosis mostly comprises expert guidance, narrative reviews, and international consensus statements [12–15]. To our knowledge, there is only one existing systematic review about cystinosis that specifically investigates the efficacy and safety of topical cysteamine [16]. As outlined in the report from the Kidney Disease: Improving Global Outcomes Conference, the research gap for cystinosis diagnosis and management must be addressed to systematically collect and synthesize evidence for guideline development [17]. This systematic review aimed to identify evidence for cystinosis diagnosis and treatment to support the development of evidence-based guidelines.

## Methods

We followed the Cochrane Handbook for Systematic Reviews of Interventions [18] and adhered to the Preferred Reporting Items for Systematic Reviews and Meta-Analyses (PRISMA) statement throughout this review [19]. We registered the protocol a priori in the Open Science Framework (https://osf.io/rb6py/). Subsequently, but before the search, we extended the list of eligible populations for indirect comparisons in key questions (KQs) 29–31 to include neurofibromatosis (NF1), Pompe disease (glycogen storage disease), coenzyme Q10 deficiency, and alkaptonuria (Tables S29–S31).

### Key questions and eligibility criteria

In this systematic review, we investigated 31 KQs and 21 corresponding sub-questions from 14 different topics related to the diagnosis and management of cystinosis. In the overall guideline project, the guideline group formulated 16 additional non-PICO clinical questions, based on recommendations of topic experts in the field.

We defined sub-questions for different subgroups, mostly for age groups, starting age of treatment, dosages and modes of administration, type of cystinosis, stages of kidney disease, and other population or specific disease characteristics. All detailed eligibility criteria for the KQs and sub-questions can be retrieved from Appendix section B (Tables S2–S31), while an overview of all investigated key questions with corresponding comparisons is listed in Table 1 below. We adjusted the eligibility criteria for included studies after our protocol was published, originally limited to those published from 1997 onward, the year cysteamine was first approved under the trade name Cystagon^®^ by the European Medicines Agency (EMA). This criterion was expanded to include works published from 1980 onward, reflecting the period when cysteamine began being used off-label in the United States.

**Table 1:** Key questions, listed as comparisons and categorized into broader topics

Topic	Numbers of key questions and corresponding comparisons
**Diagnostics**	1) Cystine detection in pure granulocytes vs. mixed leukocytes
**Newborn screening**	2) Screening vs. no screening 3) Glycosuria test vs. genetic testing
**Cysteamine treatment**	4) Systemic cysteamine vs. no treatment* 5) Immediate-release vs. delayed-release cysteamine*
**Kidney disease (Fanconi syndrome)**	6) Electrolytes vs. no intervention* 7) Alkali deficiency vs. no intervention* 8) Vitamin D vs. no intervention* 9) Indomethacin therapy vs. no indomethacin therapy* 10) Thiazide therapy plus bicarbonate/citrate vs. bicarbonate/citrate alone* 11) RAAS blockade vs. no intervention* 12) RAAS blockade combined with indomethacin vs. RAAS alone or indomethacin alone or no intervention*
**Kidney transplantation**	13) Surgical/pharmacological nephrectomy vs. no nephrectomy*
**Bone disease**	14) Phosphate combined with active vitamin D vs. phosphate alone* 15) Calcium vs. no calcium supplementation* 16) Cysteamine treatment early in life vs. late in life
**Endocrinological disorders**	17) Testosterone replacement therapy vs. no testosterone replacement therapy* § 18) Chronic kidney disease and cystinosis vs. chronic kidney disease and no cystinosis 19) Cysteamine therapy in pregnancy vs. no cysteamine therapy in pregnancy
**Gastrointestinal disorders**	20) Additional proton pump inhibitors added to cysteamine therapy vs. cysteamine therapy alone* §
**Muscle weakness**	21) L-carnitine and/or coenzyme Q10 supplementation vs. no supplementation* 22) Preventive physiotherapy vs. no physiotherapy*
**Neurological disorders**	23) Early imaging or psychological testing or senso-motoric testing vs. no intervention 24) Occupational therapy or educational interventions or behavioral interventions or psychotherapy or other co-interventions vs. no interventions*
**Eye disease**	25) Systemic cysteamine with concomitant cysteamine eye drops vs. systemic cysteamine treatment alone* 26) Systemic cysteamine treatment vs. no treatment* 27) Cysteamine eye drops with benzalkonium chloride vs. cysteamine eye drops without benzalkonium chloride* 28) Optical coherence tomography or corneal densitometry or slit lamp photography vs. in vivo confocal microscopy
**Interdisciplinary care**	29) Integrated care vs. standard care* §
**Transition**	30) Interventions built on a transition of care model vs. other or no interventions* §
**Psychosocial well-being**	31) Psychosocial support vs. no psychosocial support* §

Abbreviations: RAAS, Renin-Angiotensin-Aldosterone-System; vs., versus

* KQ with sub-question (see Appendix, section B)

§ indirect comparisons with populations without cystinosis but with comparable symptoms or needs

### Systematic literature search

Our literature searches followed a stepwise approach, comprising three iterations. All database searches were designed and conducted by an experienced information specialist (IK). A first set of relevant studies was provided by the guideline panel and supplemented by additional preliminary searching. We used these known relevant references to identify search terms and test the sensitivity of the MEDLINE search strategy.

Where possible, we used a combination of free text and controlled vocabulary (e.g., Medical Subject Headings) and limited the searches to English-language and human-only studies (Appendix C).

In the first step, we conducted literature searches in April 2023 in the following databases to identify published research literature, study protocols, and dissertations about cystinosis: Ovid MEDLINE, Embase.com (Elsevier), Cochrane Central Register of Controlled Trials (CENTRAL) via the Cochrane Library, CINAHL Database (Ebsco), ClinicalTrials.gov, the World Health Organization’s International Clinical Trials Registry Platform (ICTRP), Dissertations & Theses Global (ProQuest), and Bielefeld Academic Search Engine (BASE [https://www.base-search.net]).

After the first round of study selection, we conducted additional searches for guidelines and systematic reviews in other populations (e.g., rare systemic diseases) that could provide indirect evidence relevant to KQ17, KQ17a, KQ24, KQ24a, KQ29, KQ29a, KQ30, KQ30a, KQ31, and KQ31a. Searches were conducted in July 2023 using the following databases: Ovid MEDLINE, Cochrane Database of Systematic Reviews (Cochrane Library/Wiley), Epistemonikos Database (Epistemonikos.org), Trip Medical Database (tripdatabase.com/), AWMF Leitlinien-Register (register.awmf.org/), Guidelines International Network (GIN) Library (guidelines.ebmportal.com), and National Center for Biotechnology Information (NCBI) Bookshelf (https://www.ncbi.nlm.nih.gov/books/).

Additionally, we searched the websites of select guideline providers for relevant documents: National Institute for Health and Care Excellence (NICE), Canadian Agency for Drugs and Technologies in Health (CADTH), and the US Agency for Healthcare Research and Quality (AHRQ).

To identify published research literature about participants with Fanconi syndrome (KQ15, KQ15a) and those with a lifelong need for eye therapy (KQ27, KQ27a), we conducted searches in July 2023 in Ovid MEDLINE, Cochrane Database of Systematic Reviews (Cochrane Library/Wiley), and the Epistemonikos Database.

### Study selection

All researchers involved in the study selection (IS, DL, BNS, BP) performed a pilot exercise on 50 references from the search at the title and abstract levels to ensure a clear and common understanding of the eligibility criteria. We subsequently screened all references as they were available from the searches dually. Conflicts were resolved through discussion between the two reviewers or by consulting a third team member. For studies with insufficient information for inclusion or exclusion, we retrieved the full text for determination. After piloting full-text screening with five references, two trained reviewers independently reviewed each full-text article for inclusion or exclusion, with conflicts resolved by discussion or by a third reviewer. The literature screening was conducted using the systematic review software DistillerSR [20].

We used a predefined staggered approach for the inclusion of study designs. We considered both controlled and uncontrolled study designs during the study selection process. However, if we identified at least one study with a control group for an outcome, we did not consider studies without a control group. To ensure clear comparability, we categorized the designs of the included studies according to the US AHRQ classification [21].

### Data extraction

For studies that met our inclusion criteria, we extracted characteristics of study populations (country, setting, age, gender), study design, interventions, comparators, and relevant outcomes (definition of outcomes, raw numbers and effect estimate reported in the publication) into predefined extraction forms. We developed these forms and piloted them with two studies to ensure correct and consistent data extraction. Trained reviewers (IS, DL, BNS, BP) first extracted the relevant data and, in a second step, reviewed the data extractions for completeness and accuracy.

### Risk of bias assessment

To assess the risk of bias in the included studies, we used the Cochrane Risk of Bias (RoB 2) tool [22] for randomized controlled trials (RCTs), the Risk Of Bias In Non-randomized Studies-of Interventions (ROBINS-I) tool [23] for non-randomized trials and cohort studies, and the Effective Public Health Practice Project (EPHPP) [24] tool for before–after studies. We critically appraised guidelines with the Appraisal of Guidelines for Research & Evaluation II (AGREE II) instrument [25], case reports with the JBI checklist for case reports [26], and case series with the JBI critical appraisal tool for case series [27]. Critical appraisal was conducted independently by two experienced reviewers (AD, AG).

### Data synthesis and analysis

We synthesized data narratively and structured the synthesis by KQ, comparisons, and outcomes. We plotted an evidence map to show the body of evidence per KQ (Fig. 2).

### Interest holder involvement

During the refinement of the KQs, we asked the guideline development group to rate the importance of outcomes with a nine-part Likert scale according to the Grading of Recommendations Assessment, Development and Evaluations (GRADE) approach [28]. A total of four patient representatives and 82 clinical experts were involved in ranking the outcomes in two survey rounds, as well as helping to refine all KQs and eligibility criteria. We used the web application Surveylet (Calibrum Inc., available from https://calibrum.com/), a collaborative research platform with a multi-round real-time Delphi approach, in April and May 2023.

### Certainty of evidence rating

We rated the CoE based on the guidance established by the GRADE Working Group [29], using the GRADEpro online tool (GRADEpro GDT: GRADEpro Guideline Development Tool. McMaster University and Evidence Prime, 2024. Available from https://www.gradepro.org/). One researcher (IS) rated the CoE, while a second researcher (AD) reviewed all decisions for completeness and accuracy. We only considered outcomes deemed to be of critical importance (7–9) or of importance to decision-making (4–6). We developed a summary-of-findings table to support the guideline development group’s decision-making process.

## Results

**Fig. 1:**
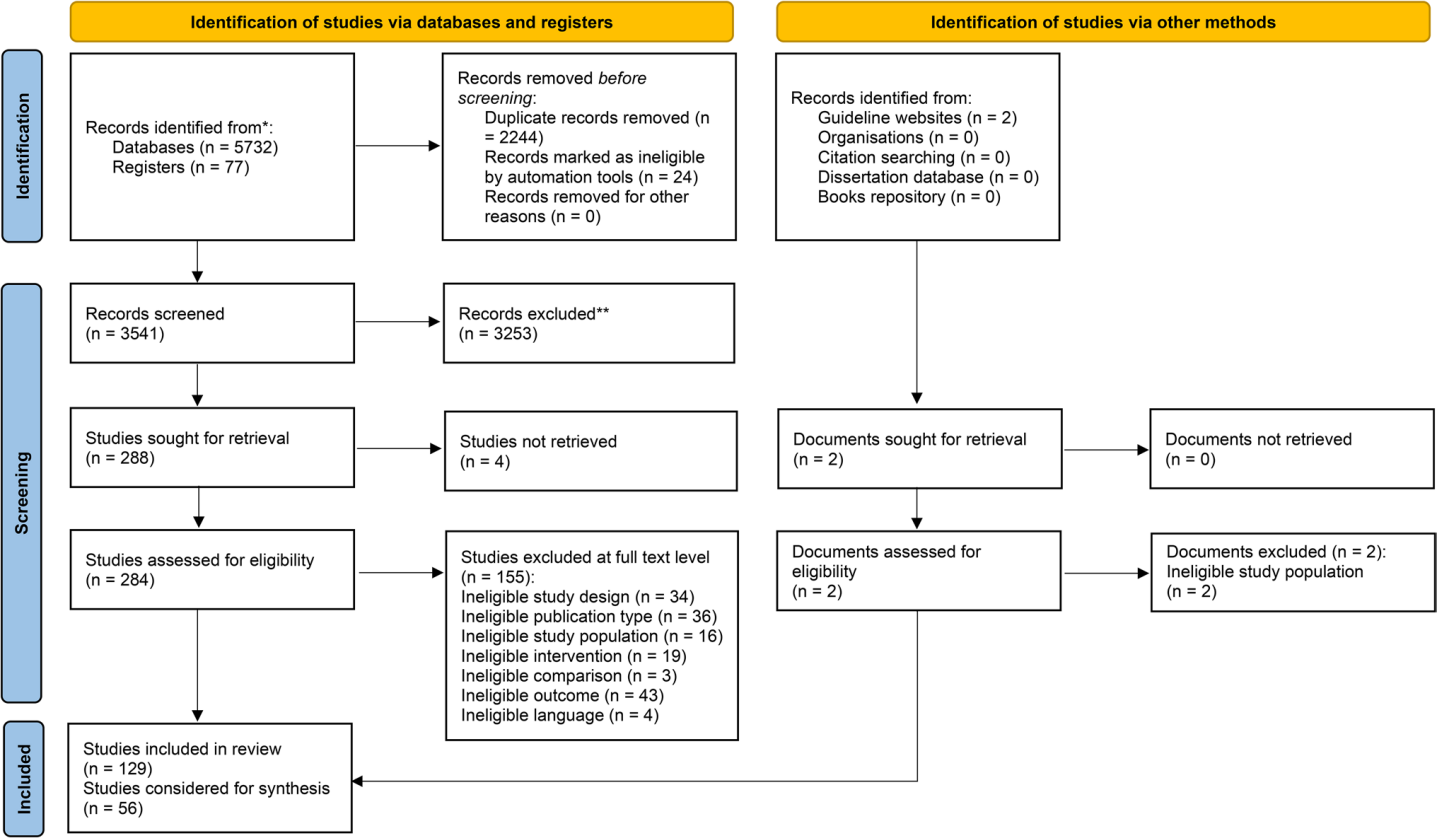
PRISMA flow chart of the study selection process

Our searches returned 3543 hits after deduplication, from which we checked 290 for eligibility at the full-text level (Fig. 1). A total of 129 studies met our general eligibility criteria, of which we considered 56 in our analysis due to the staggered approach. We included one guideline [30], two systematic reviews [31, 32], seven RCTs [33–39], one non-randomized comparative trial [40], fifteen controlled cohort studies [41–55], fifteen before–after studies [56–70], eight case series [71–78], and seven case reports [79–85]. We excluded 157 references on the full-text level and recorded their reasons for exclusion (Appendix D). We were not able to retrieve 4 full-texts (Appendix D) and we did not consider 73 studies (Appendix E), due to the predefined staggered approach of only utilizing controlled studies if any were available. We rated 35 studies as high risk for bias, the majority of which, 74% (26/35), was due to confounding. We had some concerns in eighteen studies, and two systematic reviews and one guideline were considered to have a low risk of bias (detailed reasons for ratings can be retrieved from Appendix G).

**Fig. 2:**
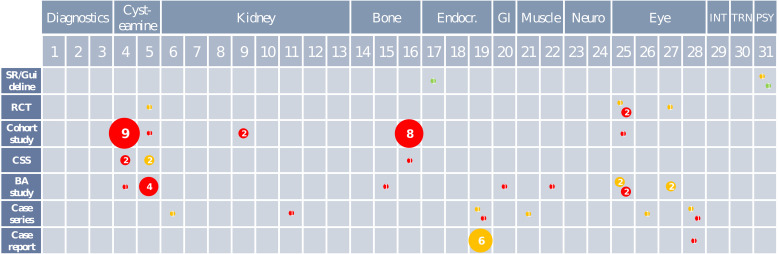
Map of the available evidence. Abbreviations: BA study, before–after study; CSS, cross-sectional study; Endocr., Endocrinological; GI, gastrointestinal; INT, interdisciplinary care; PSY, psychosocial support; RCT, randomized controlled trial; SR, systematic review; TRN, transition of care Risk of bias: High Some concerns Low 1^st^ row: topic categories; 2^nd^ row: KQs; 1^st^ column: study designs Bubble size and #: Number of studies per KQ

We found eligible studies for 17 of 31 predefined KQs. For 52 outcomes, we rated the CoE as very low. Three outcomes had low CoE, four had moderate CoE, and three had high CoE. Across all 31 KQs, there were 151/213 (71%) outcomes without any evidence. We prepared summary-of-findings tables for all KQs with at least one outcome with evidence (see Appendix, Section H). We plotted the available evidence as a map, depicting the type of studies considered for each KQ as bubbles. The bubbles were sized after the number of included studies and colored according to their risk of bias (Fig. 2).

### Study and participant characteristics

This systematic review encompasses studies from all over the world. The 56 considered studies (see Appendix, Section I) included 5835 participants in total, from which there were 2403 participants with confirmed cystinosis. The remainder came from indirect comparisons (other chronic multi-organ diseases). We assessed indirect evidence from 3432 individuals with other rare diseases that present with similar secondary conditions like Fanconi syndrome [58] and testosterone deficiency [32] or that have comparable multi-organ conditions that require similar interventions, such as psychosocial support [30, 31].

While most studies only included participants with confirmed nephropathic cystinosis, one case report included an adult with non-nephropathic ocular cystinosis [82]. Four studies did not report the phenotype of cystinosis [34, 58, 59, 75], even though two of them only included children with a mean age at diagnosis of 1.8 and 1.2 years, respectively [59, 75], indicating nephropathic cystinosis [82].

In studies that reported the age when cystinosis was diagnosed, participants ranged in age from younger than 2 months to 23 years. The largest cohort study with 453 participants, the mean age of initiation of cysteamine therapy was 1.6 years (range 1.0 to 2.8) [43].

### Systemic cysteamine treatment (KQ4-5)

We found no evidence for 151 outcomes from all KQs. Identified subgroup results for KQs 4, 5, 16, 26, and 28 can be retrieved from the Appendix, Section J.

KQ4 compared systemic cysteamine treatment with no therapy or usual care in cystinosis participants. The evidence from three cohort studies (*n* = 184) regarding the effects of cysteamine on survival is of very low CoE. The largest study by Gahl et al., published in 1987 [45], included 148 cystinosis participants and showed a survival rate of 98.9% (92/93) for those treated with cysteamine versus 92.7% (51/55) for untreated participants who received either a placebo or ascorbic acid, after a maximum follow-up of 73 months. A more recent study [53] reported similar survival rates between treated and untreated participants, whereas another study [42] confirmed higher survival rates among participants receiving cysteamine treatment, as shown in the 1987 study by Gahl et al.

Four cohort studies [42, 45, 49, 50] investigated the effect of systemic cysteamine on renal involvement in cystinosis participants (*n* = 337, very low CoE). The largest cohort study [50] with 147 cystinosis participants found that those treated with systemic cysteamine reached end-stage renal disease (ESRD) later than those without cysteamine (mean age 15.4 years [standard error of the mean [SEM] 0.7], *n* = 53 vs. 10.3 years [SEM 0.3], *n* = 94). Across three other studies [42, 45, 49] participants treated with cysteamine showed similar renal outcomes when compared with untreated participants.

We found evidence of very low certainty for extra-renal involvement in cysteamine-treated participants from one cohort study with 78 individuals [42]. Cystinosis participants with cysteamine treatment were older when diagnosed with hypothyroidism (13.5 vs. 11.7 years) and diabetes (17.6 vs. 14.4 years), but younger when diagnosed with neuromuscular disorders (17.8 vs. 21.9 years) compared with participants who had no treatment.

For the outcome cystine level (very low CoE), there was one cohort study with 44 cystinosis participants [49]. Those treated with cysteamine had cystine levels of 1.1 ± 0.7 (mean ± standard deviation [SD]) nmol 1/2 cystine/mg of protein vs. 8.8 ± 5.5 (mean ± SD) nmol 1/2 cystine/mg of protein for untreated participants.

We rated the CoE for the outcome growth as very low, including one cohort study (*n* = 148) [45]. Participants treated with cysteamine increased in height by 0.24 SD of the normal mean during the first year of treatment, whereas controls decreased on average by 0.59 SD. During the first year of treatment, the mean height of treated participants was 73.5% of the normal, as compared to 59.2% in untreated participants (Table S36).

KQ5 compared delayed-release (DR) cysteamine therapy to the immediate-release (IR) formulation. For cystine level (nmol 1/2 cystine/mg protein) and adverse events, there was low certainty evidence from one cross-over trial (*n* = 43) with a treatment period of three weeks per arm [37]. Participants treated with IR cysteamine had mean cystine levels of 0.97 (s ± 0.19), compared to mean levels of 0.70 ± 0.19 after they switched to the DR formulation (mean difference [MD]: -0.27 ± 0.36; 95% confidence interval [CI] -0.63 to 0.09). There were 26 adverse events in 43 participants with IR cysteamine and 75 events in the same group of participants after switching to DR cysteamine.

Treatment adherence was investigated in one cohort study [46] with 21 participants. The median percentage of days with partial or good adherence was 88 (range: 1–99) with DR formulation, compared to 2 (range: 0–22) with IR formulation (very low CoE). Improvements in quality of life resulting from treatment with IR were maintained in 40 cystinosis participants who switched from IR to DR formulation in one before–after study with a 24-month follow-up [63]. The subcategories of social function, school function, and total function for quality-of-life scores showed significant changes when compared with the scores before treatment (very low CoE). We included three before–after studies [56, 63, 67] with a total of 67 participants who reported on renal involvement and extra-renal involvement, with both outcomes having a very low CoE. The largest study with 40 participants reported a mean estimated glomerular filtration rate (eGFR) (ml/min per 1.73 m²) of 63 (SD ± 25) for IR cysteamine [63]. After a switch to DR cysteamine, at a follow-up of 24 months, these participants had an eGFR of 57 (SD ± 25). The height z-scores were − 1.15 (SD ± 0.93) for IR and − 1.21 (SD ± 0.96) after switching to DR cysteamine in participants from the same study. The smaller studies reported similar effects on renal and extra-renal involvement (Table S37).

### Kidney disease (Fanconi syndrome) (KQ6–12)

There is very low CoE for the effectiveness and safety of electrolytes vs. no intervention on serum electrolyte levels and growth in participants with cystinosis (KQ6) from a case series with six participants [73]. Two of six participants received oral electrolyte supplementation while the others received no supplementation. Serum electrolyte levels of all participants were within the normal range for potassium [mmol/l]: 3.5 to 4.4, with similar outcomes for other documented serum levels. Anthropometric scores of all children with ages ranging from 2 to 18 years were within the normal range: height (percentiles, range) 27 to 90; weight (percentile, range) 14 to 88; weight (kilogram, range) 15.1 to 62.7 (Table S38).

For KQ9, there is very low CoE for the effectiveness and safety of indomethacin therapy vs. no indomethacin therapy on growth and disease progression in participants with cystinosis. In one cohort study with 23 participants, the hazard ratio (HR) for an association of indomethacin with poor growth was 0.30 (95% CI 0.05 to 1.94, *P* = 0.20) [47]. In the largest available cohort study, when comparing 186 children who received indomethacin to 247 who did not, the HR of progressing to stage 5 chronic kidney disease (CKD) was 0.95 (95% CI 0.68 to 1.34, *P* = 0.78) [43]. A smaller cohort study showed results for CKD stage 3 similar to the reported study above (Table S39) [47].

For KQ11, there is very low CoE for effectiveness and safety of RAAS blockade (angiotensin converting enzyme [ACE]-inhibitor/angiotensin receptor blockers [ARB]) compared to no intervention in participants with cystinosis. One case series with five participants reported data on adverse events and albuminuria [75]. Two out of five participants (40%) treated with the ACE inhibitor experienced hypotension. Albuminuria diminished in all five participants within three months, with a mean reduction of 43% (range 4–72%) (Table S40).

### Bone disease (KQ14-16)

We identified one before–after study with 11 participants with Fanconi syndrome investigating calcium supplementation (KQ15) [58]. Findings from very low CoE showed mean calcium serum levels (nmol/l, SEM) of 2.33 (0.04) before and 2.20 (0.03) after treatment with calcium, phosphate, and various types and dosages of vitamin D replacement (normal calcium range according to study: 2.23 to 2.53; Table S41).

For the comparison of early (treatment start of < 18–24 months of life) versus late-in-life systemic cysteamine treatment (KQ16), the evidence (low CoE) suggests that early cysteamine treatment improves renal function (CKD stage) compared to late cysteamine treatment. Overall, seven included studies [41–43, 51, 52, 54, 55] with 755 participants provided data for this outcome. The largest cohort study showed that younger age at the start of treatment was associated with a lower risk of stage 5 CKD progression (HR = 1.24, 95% CI 1.09 to 1.42) [43]. Smaller studies reported slightly different outcomes (renal survival, ESRD, kidney transplantation, eGFR), all in favor of an early start of treatment [41, 42, 51, 52, 54, 55].

For the outcome growth (very low CoE), we included two cohort studies [48, 54] with 203 cystinosis participants, in which 139 provided data for the comparison of early vs. late start of treatment. Early treatment start resulted in mean height z-scores (± SEM/SD) of -2.17 ± 0.39 and − 3.64 ± 1.92, compared to scores for later-age treatment start of -4.07 ± 0.39 and − 4.16 ± 1.37. (Z-scores refer to standard deviations above or below the mean of a reference population, so values of 0 would reflect growth in line with the population mean.)

In one cross-sectional study, children (mean age 7.3 years) had better intellectual function (mean ± SD, full-scale intelligence quotient) when receiving early cysteamine treatment (94.0 ± 12.5) compared to late treatment (83.0 ± 15.1) (*n* = 46; very low CoE) [70]. The risk of spatial problems from the same study was assessed with the Woodcock–Johnson Psychoeducational Battery (higher scores indicate better visual–spatial ability), with mean scores (± SD) of 104.5 ± 12.0 for early versus 92.7 ± 22.6 for late treatment (Table S42).

### Endocrinological disorders (KQ17–19)

Regarding KQ17, one included systematic review investigated adolescents and adult males with endocrine deficits and low testosterone levels [32]. The review found a slight increase in libido (standardized mean difference [SMD] 0.17; 95% CI 0.01 to 0.34; *n* = 1383) and erectile function (SMD 0.16; 95% CI 0.06 to 0.27; *n* = 1344) in participants receiving testosterone replacement therapy compared to no therapy (high CoE). In addition, testosterone replacement therapy probably results in a slight improvement in mood compared to no therapy (SMD 0.08; 95% CI -0.03 to 0.20; *n* = 1179; moderate CoE). Adverse events, assessed with lower urinary tract infections, probably increase with testosterone replacement therapy compared to no therapy (MD 0.38; 95% CI -0.67 to 1.43; *n* = 866; moderate CoE; Table S43).

For KQ19 (cysteamine therapy in pregnancy compared to no cysteamine therapy), we only identified case series and case studies. In the largest case series 7 of 19 women (36.8%) had already stopped systemic cysteamine treatment at pre-pregnancy, while 11 of 19 (57.9%) stopped on confirmation of pregnancy, and 1 woman stopped after 5 weeks of pregnancy [77]. No pregnant women continued treatment through pregnancy. We are very uncertain about the effects of cysteamine therapy on pregnant patients, on the progression of cystinosis and CKD (*n* = 16; for detailed results see Table S44) [71, 77, 79, 81], and on child development (*n* = 16) [77, 79, 80, 84].

All 16 children from the Servais study were healthy and developing well at last follow-up (range 11.5 months to 35 years). With regard to stillbirth [71, 77, 79–81, 83–85], with *n* = 20, 19 of the women did not experience stillbirth, and 1 woman experienced stillbirth at 25 weeks of gestation [83]. Pregnancy and birth complications were detailed in several studies with *n* = 20; for detailed results, see Table S44 [71, 77, 79–81, 83–85]. Child ICU admission was addressed with a total of *n* = 21; 9 out of 21 children were admitted to ICU [71, 77, 79–81, 84, 85]. A group of *n* = 21 was small for gestational age [71, 77, 79–81, 84, 85]. In studies 1–6, birth weight was appropriate for age in eight children; in study 7, birth weight was [median, range, gram] 2175 [620 to 3374]; see Table S44 [77].

### Gastrointestinal disorders (KQ20)

For the effectiveness and safety of adding proton pump inhibitors (PPI) to cysteamine therapy compared to cysteamine therapy alone, we found one before–after study with 12 participants [59]. The mean symptom scores (0–2 points; 0 = no symptoms; 1 = mild symptoms; 2 = symptoms severe enough to interrupt daily activities) decreased from 1 at baseline to 0 for heartburn, from 1.2 at baseline to 0.1 for nausea, and 1.2 at baseline to 0.1 for vomiting. Weight change ranged from − 0.8 to 2 kg after 16 weeks of PPI therapy. None of the participants (0/12) had gastric ulcers or gastrointestinal (GI) perforation. One out of 12 children (8.3%) experienced adverse events, including mild headache and recurrence of aphthous ulcers (oral canker sores) during therapy (all reported outcomes with very low CoE; Table S45).

### Muscle weakness (KQ21–22)

Regarding KQ21, we found very low CoE on the effectiveness and safety of L-carnitine and/or coenzyme Q10 supplementation compared to no supplementation in one case series with 12 cystinosis participants [72]. Participants receiving L-carnitine had higher total muscle carnitine levels than untreated children (mean ± SD, nmol/mg protein): 27.1 ± 5.4 (within normal range) vs. 10.9 ± 3.6 (outside normal range). None of the children (0/6) treated long-term with oral L-carnitine experienced any adverse events (Table S46).

We found one before–after study for the effectiveness and safety of preventive physiotherapy compared to no physiotherapy with 42 cystinosis participants (KQ22) [65]. The evidence is very uncertain about the effects of preventive physiotherapy, with no significant changes after five weeks of therapy for muscle strength, quality of life, and physical functioning. Respiratory function measured as maximum expiratory pressure improved from 63.4 cm H_2_O (95% CI 45.4 to 81.4) to 80.9 cm H_2_O (95% CI 60.4 to 101.5), with similar results for other respiratory function outcomes (for detailed results, see Table S47).

### Eye disease (KQ25–28)

For KQ25, we identified one RCT with five participants reporting a slight improvement in visual acuity (outcome vision loss) in the treated eye (0.2% cysteamine drops) compared with the eye treated with placebo drops (saline) in 3/5 (60%) participants [33]. All participants (5/5) reported improvements in photophobia in a follow-up of six months (both outcomes very low CoE).

Itchy eyes were documented in two RCTs involving a total of 30 participants [38, 39]. After treatment with local cysteamine eye drops, itchy eyes were reported in 40% (6/15) of participants in one trial [38] and 6.7% (1/15) in the other trial [39]. None of the 27 treated participants had adverse events, according to data from two additional RCTs [35, 36]. Adherence ranged from 60% (3/5) “good-very good” [33], to 100% (2/2) “excellent” [35], to better adherence in those responding to treatment (decrease in crystal density in treated eye): 40% (10/25) responded to treatment All KQ25 outcomes are of very low CoE (Table S48).

For KQ 26 (cysteamine treatment compared to no treatment), we identified one case series with 208 participants documenting systemic cysteamine treatment and a follow-up of 28 years [78]. Visual acuity (outcome vision loss) in participants receiving oral cysteamine treatment was 94.5% (188/199). The remaining 5.5% (11/199) were classified as no light perception vision up to being able to count fingers in at least one eye. Among 153 participants with retinal pigment epithelium changes, 58.8% (90/153) had hypopigmentation of the retinal pigment epithelium in the periphery with pigmentary stippling, among other reported alterations.

Approximately half of 112 participants receiving oral cysteamine therapy had mild to severe abnormalities of the visual field (outcome visual field loss), and maculopathy (macular pigmentary changes) were observed in 9.2% (14/153) of cases, with one patient (0.7%) suffering from retinal pigment epithelial atrophy in the peri-macular area (Table S49).

We included one RCT with 14 participants for KQ27 [34]. Adherence (very low CoE) using eye drops eight or more times a day was reported in 57.1% (8/14) of participants and 5–7 times a day in 14.3% (2/14), independent of eye drops with or without benzalkonium chloride. Adverse events (burning sensation in the eyes) occurred equally often in participants using eye drops with and without benzalkonium chloride (7.1% [1/14] vs. 7.1% [1/14]) (very low CoE; Table S50).

For KQ28, we included two case series detailing the effects of optical coherence tomography (OCT), corneal densitometry, or slit lamp photography compared to in vivo confocal microscopy (IVCM) on cornea thinning [74, 82]. With anterior segment optical coherence tomography (AS-OCT), the mean central corneal thickness (± SD, µm) was 543.47 ± 29.62; IVCM was 531.87 ± 34.77 in the larger study [74]. The other included case series shows similar results (very low CoE) [82]. In a case series involving 75 participants, clinician- and self-assessed estimations of photophobia were found to be correlated (very low CoE; Table S51) [76].

### Psychosocial well-being (KQ31)

There is a high CoE that psychosocial support increased adherence (assessed with percentage of prescribed inhaled therapies taken for cystic fibrosis participants) compared to no psychosocial support in 588 participants with rare multi-organ diseases on a follow-up of 12 months (systematic review; MD 9.5; 95% CI 8.6 to 10.4) [31]. Psychosocial support probably increases quality of life but showed no effect on the incidence or severity of psychosocial disorders compared to no psychosocial support for the indirect population of participants with cystic fibrosis (Quality of life: *n* = 539; MD 3.9; 95% CI 1.2 to 6.6; moderate CoE; incidence or severity of psychosocial disease: *n* = 535; MD 0.3; 95% CI -0.4 to 1.0; moderate CoE; Table S52).

We identified a guideline about psychosocial care for children and adults with epidermolysis bullosa and their families [30]. It issued a strong recommendation for easy access to psychosocial support to improve quality of life. This involves psychological support and close monitoring, facilitation of participation in social activities, considering restrictions in physical and social activities (Grade strength: B; quality of evidence [average]: 2++; 5 studies). In addition, the guideline strongly recommends psychosocial family support to improve the family quality of life, as the caregiver’s quality of life may also be impacted (Grade strength: B; quality of evidence [average]: 2+; 3 studies). For psychosocial well-being in the same population (epidermis bullosa), there is a strong recommendation for psychosocial support to improve the well-being of participants and their families (Grade strength: C and C; quality of evidence [average]: 2 + and 2-; 5 studies and 6 studies).

## Discussion

This is the first comprehensive systematic review on the diagnosis and management of cystinosis. The review was produced in collaboration with an international guideline development group to inform recommendations for the first evidence-based cystinosis guideline (SELECT - S3 guideline for cystinosis). We synthesized data from 56 studies and found evidence for 62 out of 213 predefined outcomes of 31 key questions, indicating a large research gap on diagnosis and management of this rare disease. In addition, the evidence identified was very uncertain about the effectiveness and safety of investigated interventions. For systemic cysteamine treatment, we found only evidence of low to very low certainty or no evidence at all. There was low CoE from one non-inferiority trial for little to no difference in DR compared to IR cysteamine therapy (KQ5) on the effect on cystine levels and a slight increase in adverse events. Furthermore, there was low CoE that early cysteamine treatment better sustained renal function, reported as CKD or renal survival, than late cysteamine treatment (KQ16). We identified moderate to high CoE only for indirect comparisons, like other rare multi-organ diseases, rather than cystinosis with similar or comparable symptoms. However, for the majority of the questions asked by the guideline development group, there was either no evidence or very low CoE.

Systematic reviews of cystinosis are scarce. The only identified systematic review focused on the efficacy and safety of topical cysteamine treatment in corneal cystinosis [16]. Due to methodological limitations within this review (major discrepancies between protocol and publication on outcome selection without reason or explanation), we chose not to include its findings in our analysis, but we reviewed and considered all the included primary studies.

The findings of this systematic review highlight a critical challenge in rare diseases such as cystinosis: the need for making decisions in clinical care despite a limited evidence-base. In situations, when high-certainty evidence is lacking clinical decision-making may be informed by lower-certainty evidence, and factors like practitioner expertise and patient preferences gain more weight – an approach that aligns with the core principles of evidence-based practice [86]. In some instances guideline groups formulate strong recommendations for a treatment, even if the certainty of evidence is low or very low – this type of recommendations are referred to as “discordant recommendations” and they are present in multiple clinical guidelines. For example, in an analysis of 33 WHO guidelines, Alexander et al. (2016) identified 160 strong recommendations based on low or very low certainty evidence [87]. This pattern is particularly pronounced in rare diseases, where the vast majority of recommendations is usually made on the basis of low to very low certainty evidence due to the scarcity of robust clinical data [88–90]. But even in situations with limited evidence the applied GRADE approach provides a structured and transparent approach that guides the panel from formulating the questions, involving multiple interest-holders, to gather and critical appraise the evidence, and consider contextual factors when formulating recommendations for clinical practice [91]. However, these methodological capabilities do not address the fundamental challenge that clinical practice guidelines for rare diseases remain much less available than for common diseases, with guidelines for extremely rare diseases being particularly scarce [92].

Despite the lack of robust trials, cysteamine therapy has become an indispensable component in the management of cystinosis. This widespread clinical acceptance is based primarily on observational studies and clinical experience. Recent international consensus papers and expert statements, emphasize a multidisciplinary approach and highlight the importance of systemic cysteamine for all cystinosis patients as early as possible [8, 14]. This makes new trials challenging, as withholding cysteamine from a control group would mean withholding state-of-the art treatment and introducing risk of disease progression [86].

Furthermore, clinicians in another consensus statement emphasized the importance of adherence to cysteamine treatment, as it remarkably influences the progression of the disease and its manifestations [9]. Adherence is particularly critical during the transition from childhood to adolescence, when patients begin to self-administer their medication, where a significant drop in adherence has been observed [93]. Supporting this, our systematic review identified low-quality evidence that underscores the necessity of treatment adherence. In addition, a very recent modeling study concluded that, besides clinical and patient-relevant outcomes, improved treatment adherence for nephropathic cystinosis patients would also result in remarkable lifetime cost savings for patients in the UK due to better kidney function and the potential to avoid or postpone dialysis and kidney transplantation [94].

Cystinosis is a lifelong, treatable but incurable disease with numerous secondary manifestations that place a heavy burden on patients and caregivers. As the majority of those affected are infants, children and their families, quality of life and ways to improve it are critical. We only found very low evidence for changes in quality of life when comparing DR and IR cysteamine formulation and for preventive physiotherapy compared to no therapy in patients with confirmed cystinosis. For all 13 other comparisons that investigated the impact on quality of life, we did not identify any evidence. In addition, a recent scoping review identifies the gap of sufficient study data to assess the quality of life of cystinosis patients and highlights the need for validated measures to assess the impact of rare diseases on health-related quality of life [95].

In the published literature, little is known about side effects of cysteamine and other treatments for cystinosis. According to data from a small RCT with a cross-over design and 43 participants, DR cysteamine may result in a slight increase of adverse events compared to the IR formulation [37]. Most of the increases in these events were related to gastrointestinal symptoms. The trial authors discussed that a 87% reduction in PPI use during DR cysteamine periods could potentially explain the increase in adverse events, particularly gastrointestinal symptoms. However, in a prospective single-arm follow-up study by the same authors [63], the monthly incidence of side effects per individual among 40 cystinosis patients treated with DR cysteamine decreased from 0.30 at baseline to 0.059 at 24 months, with no serious adverse events reported. Two recent studies investigating the effect of switching from IR to DR cysteamine report a slight reduction in side effects such as halitosis in cystinosis patients, which is an important factor in treatment adherence [96, 97].

### Strengths and limitations

We adhered to rigorous systematic review methodology when synthesizing the available evidence for a comprehensive set of 31 key questions with more than 200 outcomes. This approach allowed us to assess the most comprehensive and complete evidence base on cystinosis to date. Another strength of this review is the involvement of a variety of clinical experts and patient support groups in the PICO and outcome selection and ranking, which enabled us to investigate the most important questions relevant to clinical decision-making.

Although non-controlled studies like uncontrolled before–after studies, case reports and case series are valuable scientific literature for making new observations, generating hypotheses, and reporting rare adverse events and abnormalities in the treatment and progression of diseases, they lack a control group, which strongly limits their statistical validity [98, 99].

The staggered approach to evidence inclusion, prioritizing controlled studies over uncontrolled studies, may limit the comprehensiveness of the analysis, particularly in areas where such studies are scarce. However, randomized controlled studies are considered the gold standard for assessing the benefits and harms of healthcare interventions, as they minimize bias by randomly allocating participants to either an intervention group or a control group. Therefore, we only considered controlled studies for KQs when available. We synthesized results from non-randomized and non-controlled studies only for KQs where no other studies were available [100].

The dearth of evidence on the effectiveness and safety of the diagnosis and management of cystinosis necessitates further investigation to strengthen the evidence and to support clinical decision-making. Consequently, there is an imperative for multi-center studies and RCTs to generate robust data that will inform future advancements in the treatment of cystinosis. Collaboration between researchers may be essential to achieving these goals.

## Conclusion

Evidence on the diagnosis and management of cystinosis remains limited. Most included studies lacked a control group, had a high risk of bias, and had small sample sizes. For most key questions and outcomes, we did not identify any evidence, while the evidence was of very low certainty for the majority of those for which we synthesized findings. Despite these limitations, our review identified specific areas where some evidence exists to inform clinical decision-making. There was low evidence for KQ5 regarding DR cysteamine therapy on cystine levels and adverse events compared to IR cysteamine therapy. For KQ16, there was low evidence of increased renal function in participants with an early start of systemic cysteamine treatment compared to a later start. For KQ17, moderate evidence suggests that testosterone replacement therapy likely increases mood but probably also results in more adverse events, while there is a high CoE for a slight increase of libido and erectile function, compared to no supplementation in adolescents and adult males with endocrine deficits and low testosterone levels. Psychological support (KQ31) for patients suffering from rare multi-organ diseases probably increases the quality of life and the incidence or severity of psychosocial disease (moderate CoE), and it increases adherence to prescribed therapies (high CoE). While robust data are lacking, these findings provided the foundation for the development of a clinical practice guideline. In accordance with the principles of evidence-based practice, clinicians may integrate the best available evidence with their own expertise and patient preferences to support shared decision-making in the care of patients with cystinosis.

## Electronic Supplementary Material

Below is the link to the electronic supplementary material.

Supplementary Material 1

## Data Availability

The data supporting the findings of this review are included within the article and its additional file.
