# Supplementary material for: Diagnosis and management of cystinosis: systematic review for a clinical practice guideline
Source: Orphanet J Rare Dis. 2025 Aug 28;20:463. doi: 10.1186/s13023-025-03974-z (PMC12395644; doi:10.1186/s13023-025-03974-z)
Supplement: Supplementary file 1 — Supplementary Material 1 [file 13023_2025_3974_MOESM1_ESM.docx]

Appendix

Contents

[A. PRISMA checklist 6](#_Toc202531979)

[B. Key questions and eligibility criteria 9](#_Toc202531980)

[**Diagnostics** 9](#_Toc202531981)

[**Newborn screening** 9](#_Toc202531982)

[**Cysteamine treatment** 11](#_Toc202531983)

[**Kidney disease** 13](#_Toc202531984)

[**Kidney transplantation** 19](#_Toc202531985)

[**Bone disease** 20](#_Toc202531986)

[**Endocrinological disorders** 23](#_Toc202531987)

[**Gastrointestinal disorders** 25](#_Toc202531988)

[**Muscle weakness** 26](#_Toc202531989)

[**Neurological disorders** 28](#_Toc202531990)

[**Eye disease** 30](#_Toc202531991)

[**Interdisciplinary care** 34](#_Toc202531992)

[**Transition of care** 35](#_Toc202531993)

[**Psychosocial support** 36](#_Toc202531994)

[C. Search Strategies 38](#_Toc202531995)

[**Round 1** 39](#_Toc202531996)

[**Round 2** 41](#_Toc202531997)

[**Round 3** 58](#_Toc202531998)

[**Additional searches** 61](#_Toc202531999)

[D. List of excluded studies 63](#_Toc202532000)

[**Ineligible study design (n=34)** 63](#_Toc202532001)

[**Ineligible publication type (n=36)** 65](#_Toc202532002)

[**Ineligible study population (n=16+2)** 67](#_Toc202532003)

[**Ineligible or no intervention (n=19)** 68](#_Toc202532004)

[**Ineligible or no comparison (n=3)** 70](#_Toc202532005)

[**Ineligible outcome(s) (n=43)** 70](#_Toc202532006)

[**Ineligible language (n=4)** 73](#_Toc202532007)

[**Full text not retrievable (n=4)** 73](#_Toc202532008)

[E. List of included studies but not considered due to staggered approach 74](#_Toc202532009)

[F. Study characteristics of included studies 80](#_Toc202532010)

[G. Risk of bias of included studies 105](#_Toc202532011)

[**Risk of bias of RCTs, assessed with RoB 2.0** 105](#_Toc202532012)

[**Risk of bias of non-randomized trials and cohort studies, assessed with ROBINS-I** 106](#_Toc202532013)

[**Risk of bias of uncontrolled before-after studies, assessed with EPHHP** 107](#_Toc202532014)

[**Risk of bias of case series, assessed with the JBI checklist for case series** 108](#_Toc202532015)

[**Risk of bias of case reports, assessed with the JBI checklist for case reports** 109](#_Toc202532016)

[**Risk of bias of guidelines, assessed with the AGREE II tool** 110](#_Toc202532017)

[**Risk of bias of systematic reviews, assessed with the AMSTAR 2 tool** 110](#_Toc202532018)

[H. Summary of findings tables* 111](#_Toc202532019)

[I. Results 131](#_Toc202532020)

[KQ4 131](#_Toc202532021)

[KQ5 137](#_Toc202532022)

[KQ6 141](#_Toc202532023)

[KQ9 142](#_Toc202532024)

[KQ11 143](#_Toc202532025)

[KQ15 143](#_Toc202532026)

[KQ16 144](#_Toc202532027)

[KQ17 148](#_Toc202532028)

[KQ19 148](#_Toc202532029)

[KQ20 150](#_Toc202532030)

[KQ21 150](#_Toc202532031)

[KQ22 151](#_Toc202532032)

[KQ25 152](#_Toc202532033)

[KQ26 155](#_Toc202532034)

[KQ27 156](#_Toc202532035)

[KQ28 157](#_Toc202532036)

[KQ31 158](#_Toc202532037)

[J. Subgroup results 161](#_Toc202532038)

[KQ 4 161](#_Toc202532039)

[KQ 5 163](#_Toc202532040)

[KQ 16 163](#_Toc202532041)

[KQ 26 164](#_Toc202532042)

[KQ 28 164](#_Toc202532043)

[References 165](#_Toc202532044)

# PRISMA checklist

Table S1: PRISMA checklist

| **Section and Topic** | **Item #** | **Checklist item** | **Location where item is reported** |
| --- | --- | --- | --- |
| **TITLE** | | |  |
| Title | 1 | Identify the report as a systematic review. | p1 |
| **ABSTRACT** | | |  |
| Abstract | 2 | See the PRISMA 2020 for Abstracts checklist. | p2 |
| **INTRODUCTION** | | |  |
| Rationale | 3 | Describe the rationale for the review in the context of existing knowledge. | p3-4 |
| Objectives | 4 | Provide an explicit statement of the objective(s) or question(s) the review addresses. | p4 |
| **METHODS** | | |  |
| Eligibility criteria | 5 | Specify the inclusion and exclusion criteria for the review and how studies were grouped for the syntheses. | p5, Appendix B: S2-S31 |
| Information sources | 6 | Specify all databases, registers, websites, organisations, reference lists and other sources searched or consulted to identify studies. Specify the date when each source was last searched or consulted. | p6-7 |
| Search strategy | 7 | Present the full search strategies for all databases, registers and websites, including any filters and limits used. | Appendix C |
| Selection process | 8 | Specify the methods used to decide whether a study met the inclusion criteria of the review, including how many reviewers screened each record and each report retrieved, whether they worked independently, and if applicable, details of automation tools used in the process. | p7 |
| Data collection process | 9 | Specify the methods used to collect data from reports, including how many reviewers collected data from each report, whether they worked independently, any processes for obtaining or confirming data from study investigators, and if applicable, details of automation tools used in the process. | p8 |
| Data items | 10a | List and define all outcomes for which data were sought. Specify whether all results that were compatible with each outcome domain in each study were sought (e.g. for all measures, time points, analyses), and if not, the methods used to decide which results to collect. | Appendix B: S2-S31, Appendix F |
|  | 10b | List and define all other variables for which data were sought (e.g. participant and intervention characteristics, funding sources). Describe any assumptions made about any missing or unclear information. | Appendix F |
| Study risk of bias assessment | 11 | Specify the methods used to assess risk of bias in the included studies, including details of the tool(s) used, how many reviewers assessed each study and whether they worked independently, and if applicable, details of automation tools used in the process. | p8 |
| Effect measures | 12 | Specify for each outcome the effect measure(s) (e.g. risk ratio, mean difference) used in the synthesis or presentation of results. | p8 |
| Synthesis methods | 13a | Describe the processes used to decide which studies were eligible for each synthesis (e.g. tabulating the study intervention characteristics and comparing against the planned groups for each synthesis (item #5)). | Appendix F |
|  | 13b | Describe any methods required to prepare the data for presentation or synthesis, such as handling of missing summary statistics, or data conversions. | p9 |
|  | 13c | Describe any methods used to tabulate or visually display results of individual studies and syntheses. | p9 |
|  | 13d | Describe any methods used to synthesize results and provide a rationale for the choice(s). If meta-analysis was performed, describe the model(s), method(s) to identify the presence and extent of statistical heterogeneity, and software package(s) used. | p9 |
|  | 13e | Describe any methods used to explore possible causes of heterogeneity among study results (e.g. subgroup analysis, meta-regression). | p9 |
|  | 13f | Describe any sensitivity analyses conducted to assess robustness of the synthesized results. | Not applicable |
| Reporting bias assessment | 14 | Describe any methods used to assess risk of bias due to missing results in a synthesis (arising from reporting biases). | p8-9 |
| Certainty assessment | 15 | Describe any methods used to assess certainty (or confidence) in the body of evidence for an outcome. | p9 |
| **RESULTS** | | |  |
| Study selection | 16a | Describe the results of the search and selection process, from the number of records identified in the search to the number of studies included in the review, ideally using a flow diagram. | p9-10 |
|  | 16b | Cite studies that might appear to meet the inclusion criteria, but which were excluded, and explain why they were excluded. | p9-10; Appendix D+E |
| Study characteristics | 17 | Cite each included study and present its characteristics. | p9-10; Appendix F |
| Risk of bias in studies | 18 | Present assessments of risk of bias for each included study. | p10; Appendix G |
| Results of individual studies | 19 | For all outcomes, present, for each study: (a) summary statistics for each group (where appropriate) and (b) an effect estimate and its precision (e.g. confidence/credible interval), ideally using structured tables or plots. | P11-18; Appendix H-J |
| Results of syntheses | 20a | For each synthesis, briefly summarise the characteristics and risk of bias among contributing studies. | P10-18; Appendix G |
|  | 20b | Present results of all statistical syntheses conducted. If meta-analysis was done, present for each the summary estimate and its precision (e.g. confidence/credible interval) and measures of statistical heterogeneity. If comparing groups, describe the direction of the effect. | Not applicable |
|  | 20c | Present results of all investigations of possible causes of heterogeneity among study results. | Not applicable |
|  | 20d | Present results of all sensitivity analyses conducted to assess the robustness of the synthesized results. | Not applicable |
| Reporting biases | 21 | Present assessments of risk of bias due to missing results (arising from reporting biases) for each synthesis assessed. | Not applicable |
| Certainty of evidence | 22 | Present assessments of certainty (or confidence) in the body of evidence for each outcome assessed. | Appendix H |
| **DISCUSSION** | | |  |
| Discussion | 23a | Provide a general interpretation of the results in the context of other evidence. | p19-20 |
|  | 23b | Discuss any limitations of the evidence included in the review. | p20-21 |
|  | 23c | Discuss any limitations of the review processes used. | p21-22 |
|  | 23d | Discuss implications of the results for practice, policy, and future research. | p22 |
| **OTHER INFORMATION** | | |  |
| Registration and protocol | 24a | Provide registration information for the review, including register name and registration number, or state that the review was not registered. | p5 |
|  | 24b | Indicate where the review protocol can be accessed, or state that a protocol was not prepared. | p5 |
|  | 24c | Describe and explain any amendments to information provided at registration or in the protocol. | Not applicable |
| Support | 25 | Describe sources of financial or non-financial support for the review, and the role of the funders or sponsors in the review. | p23 |
| Competing interests | 26 | Declare any competing interests of review authors. | p23 |
| Availability of data, code and other materials | 27 | Report which of the following are publicly available and where they can be found: template data collection forms; data extracted from included studies; data used for all analyses; analytic code; any other materials used in the review. | Appendix |

# Key questions and eligibility criteria

## **Diagnostics**

**KQ1: What is the diagnostic accuracy of elevated cystine detection in pure granulocytes compared to elevated cystine detection in mixed leukocytes to diagnose cystinosis in infants, children, adolescents, and adults?**

Table S2: Inclusion/exclusion criteria for KQ1

| Category | Criteria | |
| --- | --- | --- |
|  | Inclusion | Exclusion |
| Population | Infants, children, adolescents and adults with suspected cystinosis | - Patients with confirmed cystinosis - Patients with other rare diseases |
| Index test | - Cystine detection in pure granulocytes to diagnose cystinosis | - Any other cystine detection method - Any other test to diagnose cystinosis |
| Reference test | - Cystine detection in mixed leukocytes to diagnose cystinosis | - Any other cystine detection method - Any other test to diagnose cystinosis |
| Outcomes | - Sensitivity - Specificity - Positive predictive value - Negative predictive value - Diagnostics odds ratio - Area under the curve - Receiver operating characteristics | - Any outcomes other than diagnostic test accuracy (e.g., reliability) |
| Geography | - No limitations |  |
| Settings | - Any setting |  |
| Publication language | - English, German | - All other languages |
| Study design | - RCTs - Nonrandomized controlled trials - Cohort studies - Case-control studies - Cross-sectional studies - Diagnostic test accuracy studies - Systematic reviews and meta-analyses | - Nonsystematic reviews |
| Publication type | Any peer-reviewed publication reporting primary data | Abstracts, preprints, publications not reporting primary data (e.g. protocols) |

## **Newborn screening**

**KQ2: What is the effectiveness and safety of newborn screening for cystinosis compared to no screening or targeted screening of index cases on health outcomes?**

Table S3: Inclusion/exclusion criteria for KQ2

| Category | Criteria | |
| --- | --- | --- |
|  | Inclusion | Exclusion |
| Population | Newborns | - Infants older than 3 months |
| Intervention | - Screening for cystinosis | - Clinical assessment of suspected cystinosis |
| Comparison | - No screening or targeted screening (high risk siblings) | - Clinical assessment of suspected cystinosis |
| Outcomes | - Age at start of treatment - Glomerular and tubular function (surrogate) - Clinical progression - Quality of life - Survival - Growth - Start of intensified nutrition - Adverse events | - Any other outcomes |
| Geography | No limitations |  |
| Settings | - Any setting |  |
| Publication language | - English, German | - All other languages |
| Study design | - RCTs - Nonrandomized controlled trials - Cohort studies - Case-control studies - Cross-sectional studies - Systematic reviews and meta-analyses | - Nonsystematic reviews |
| Publication type | Any peer-reviewed publication reporting primary data | Abstracts, preprints, publications not reporting primary data (e.g. protocols) |

**KQ3: What is the diagnostic accuracy of glycosuria test compared to genetic testing in diagnosing infants up to 3 months with cystinosis?**

Table S4: Inclusion/exclusion criteria for KQ3

| Category | Criteria | |
| --- | --- | --- |
|  | Inclusion | Exclusion |
| Population | Infants up to 3 months | - Infants older than 3 months |
| Index test | - Glycosuria test to diagnose cystinosis | Any other test to diagnose cystinosis |
| Reference test | - Genetic testing to diagnose cystinosis | - Any other test to diagnose cystinosis |
| Outcomes | - Sensitivity - Specificity - Positive predictive value - Negative predictive value - Diagnostics odds ratio - Area under the curve - Receiver operating characteristics | - Any outcomes other than diagnostic test accuracy (e.g., reliability) |
| Geography | No limitations |  |
| Settings | - Any setting |  |
| Publication language | - English, German | - All other languages |
| Study design | - RCTs - Nonrandomized controlled trials - Cohort studies - Case-control studies - Cross-sectional studies - Diagnostic accuracy studies - Systematic reviews and meta-analyses | - Nonsystematic reviews |
| Publication type | Any peer-reviewed publication reporting primary data | Abstracts, preprints, publications not reporting primary data (e.g. protocols) |

## **Cysteamine treatment**

**KQ4: What is the effectiveness and safety of systemic cysteamine therapy compared to no therapy/usual care in patients with confirmed cystinosis?**

**KQ4a: Are there any differences by age, starting age, dosage (per age and body weight, dosage regimes, maximum dosage), frequency of administration, type of cystinosis, pre-/post transplantation (when to stop/resume)?**

Table S5: Inclusion/exclusion criteria for KQ4/KQ4a

| Category | Criteria | |
| --- | --- | --- |
|  | Inclusion | Exclusion |
| Population | Patients with diagnosed cystinosis (all age groups) | - Patients with other rare diseases |
|  | Subgroups:   - Age, starting age of treatment, dosage per age and body weight, dosage, dosage regimes, maximum dosage, frequency of administration, type of cystinosis, pre-/post transplantation (when to stop/resume) |  |
| Intervention | - Systemic cysteamine therapy | - Local cysteamine therapy |
| Comparison | - No systemic cysteamine therapy/ usual care (electrolyte replacement/multiorgan care) |  |
| Outcomes | - Survival - Renal involvement (FANCONI-syndrome, kidney function, renal survival) - Quality of life (general discomfort, pain) - Extra-renal involvement: Muscle involvement (including general muscle involvement, pulmonary involvement, late swallowing difficulty); Neurological involvement (including neurocognitive involvement, pseudo tumor cerebri, strokes, seizures); Endocrinological involvement (including hypothyroidism, diabetes, hypogonadism, infertility); Bone involvement (including bone health (orthopedic involvement, rickets, fractures; including growth retardation (final height, age at requirement for growth hormones); Gastrointestinal involvement (including, delayed gastric emptying, motility problems, ulcers, liver involvement, pancreas involvement) - Cystine level (surrogate outcome) - Growth - Adverse events (including halitosis, skin lesions, skin striae, vomiting, diarrhea) - Cardiovascular involvement | - Any other outcomes |
| Geography | No limitations |  |
| Settings | - Any setting |  |
| Publication language | - English, German | - All other languages |
| Study design | - RCTs - Nonrandomized controlled trials - Cohort studies - Case-control studies - Cross-sectional studies - Before-after studies - Case series - Case reports - Systematic reviews and meta-analyses | - Nonsystematic reviews |
| Publication type | Any peer-reviewed publication reporting primary data | Abstracts, preprints, publications not reporting primary data (e.g. protocols) |

**KQ5: What is the effectiveness and safety of slow-release cysteamine therapy compared to immediate-release cysteamine therapy in patients with confirmed cystinosis?**

**KQ5a: Are there any differences by age, frequency of administration, dietary recommendation for administering?**

Table S6: Inclusion/exclusion criteria for KQ5/KQ5a

| Category | Criteria | |
| --- | --- | --- |
|  | Inclusion | Exclusion |
| Population | Patients with diagnosed cystinosis (all age groups) | - Patients with other rare diseases |
|  | Subgroups:   - age, frequency of administration, dietary recommendation for administering |  |
| Intervention | - Slow-release/delayed release cysteamine therapy | - Local cysteamine therapy - Any other cysteamine therapy |
| Comparison | - Immediate-release cysteamine | - Any other cysteamine therapy |
| Outcomes | - Treatment adherence - Quality of life (general discomfort, pain, family quality of life, night sleep) - Cystine level (surrogate outcome) - Survival - Adverse events (including halitosis, skin lesions, skin striae, vomiting, diarrhea) - Renal involvement (FANCONI-syndrome, kidney function, renal survival) - Extra-renal involvement: Neurological involvement (including neurocognitive involvement, pseudo tumor cerebri, strokes, seizures); Gastrointestinal involvement (including, delayed gastric emptying, motility problems, ulcers, liver involvement, pancreas involvement); Muscle involvement (including general muscle involvement, pulmonary involvement, late swallowing difficulty); Endocrinological involvement (including hypothyroidism, diabetes, hypogonadism, infertility); Bone involvement (including bone health (orthopedic involvement, rickets, fractures; including growth retardation (final height, age at requirement for growth hormones) - Cardiovascular involvement - Eye involvement (including retinopathy) | - Any other outcomes |
| Geography | No limitations |  |
| Settings | - Any setting |  |
| Publication language | - English, German | - All other languages |
| Study design | - RCTs - Nonrandomized controlled trials - Cohort studies - Case-control studies - Cross-sectional studies - Before-after studies - Case series - Case reports - Systematic reviews and meta-analyses | - Nonsystematic reviews |
| Publication type | Any peer-reviewed publication reporting primary data | Abstracts, preprints, publications not reporting primary data (e.g. protocols) |

## **Kidney disease**

**KQ6: What is the effectiveness and safety of electrolytes vs no intervention on consequences of FANCONI-syndrome in patients with cystinosis?**

**KQ6a: Are there any differences by dosage (starting and maintenance/tailored dose), administration mode, age, chronic kidney disease (CKD) stage, optimal frequency of administration, combined administration of medications?**

Table S7: Inclusion/exclusion criteria for KQ6/KQ6a

| Category | Criteria | |
| --- | --- | --- |
|  | Inclusion | Exclusion |
| Population | Patients with cystinosis and electrolyte deficiency | - Patients with cystinosis and without electrolyte deficiency - Patients with other rare diseases |
|  | Subgroups:   - dosage (starting and maintenance/tailored dose), administration mode, age, CKD stage, optimal frequency of administration, combined administration of medications |  |
| Intervention | - Electrolytes (potassium, phosphate, calcium) - Other co-interventions such as cysteamine therapy | - Other electrolytes |
| Comparison | - No electrolyte administration - Other co-interventions such as cysteamine therapy |  |
| Outcomes | - Rickets (clinical and biochemical markers) - Serum levels of electrolytes (surrogate) - Dehydration episodes - Growth - Quality of life - Neurologic development and milestones - Disease progression - Adverse events (e.g. nephrocalcinosis, urolithiasis) - Alkaline phosphate levels | - Any other outcomes |
| Geography | No limitations |  |
| Settings | - Any setting |  |
| Publication language | - English, German | - All other languages |
| Study design | - RCTs - Nonrandomized controlled trials - Cohort studies - Case-control studies - Cross-sectional studies - Before-after studies - Case series - Case reports - Systematic reviews and meta-analyses | - Nonsystematic reviews |
| Publication type | Any peer-reviewed publication reporting primary data | Abstracts, preprints, publications not reporting primary data (e.g. protocols) |

**KQ7: What is the effectiveness and safety of alkali deficiency vs no intervention on consequences of FANCONI-syndrome in patients with cystinosis?**

**KQ7a: Are there any differences by dosage (starting and maintenance/tailored dose), administration mode, age, CKD stage, optimal frequency of administration, combined administration of medications?**

Table S8: Inclusion/exclusion criteria for KQ7/KQ7a

| Category | Criteria | |
| --- | --- | --- |
|  | Inclusion | Exclusion |
| Population | Patients with cystinosis and alkali deficiency | - Patients with cystinosis and without alkali deficiency - Patients with other rare diseases |
|  | Subgroups:   - dosage (starting and maintenance/tailored dose), administration mode, age, CKD stage, optimal frequency of administration, combined administration of medications |  |
| Intervention | - Alkali (bicarbonate or citrate) - Other co-interventions such as cysteamine therapy |  |
| Comparison | - No alkali administration - Other co-interventions such as cysteamine therapy |  |
| Outcomes | - Rickets (clinical and biochemical markers) - Bicarbonate - Serum levels of electrolytes (surrogate) - Growth - Quality of life - Neurologic development and milestones - Disease progression - Adverse events (e.g. nephrocalcinosis, urolithiasis) - Alkaline phosphate levels | - Any other outcomes |
| Geography | No limitations |  |
| Settings | - Any setting |  |
| Publication language | - English, German | - All other languages |
| Study design | - RCTs - Nonrandomized controlled trials - Cohort studies - Case-control studies - Cross-sectional studies - Before-after studies - Case series - Case reports - Systematic reviews and meta-analyses | - Nonsystematic reviews |
| Publication type | Any peer-reviewed publication reporting primary data | Abstracts, preprints, publications not reporting primary data (e.g. protocols) |

**KQ8: What is the effectiveness and safety of Vitamin D vs no intervention on consequences of FANCONI-syndrome in patients with cystinosis?**

**KQ8a: Are there any differences by dosage (starting and maintenance/tailored dose), administration mode (e.g. vitamin D by mouth or tube), age, CKD stage, optimal frequency of administration, combined administration of medications?**

Table S9: Inclusion/exclusion criteria for KQ8/KQ8a

| Category | Criteria | |
| --- | --- | --- |
|  | Inclusion | Exclusion |
| Population | Patients with cystinosis and vitamin D deficiency | - Patients with cystinosis and without vitamin D deficiency - Patients with other rare diseases |
|  | Subgroups:   - dosage (starting and maintenance/tailored dose), administration mode, age, CKD stage, optimal frequency of administration, combined administration of medications |  |
| Intervention | - Vitamin D supplementation (any dose) - Other co-interventions such as cysteamine therapy | - Multivitamin supplements, combined supplements |
| Comparison | - No Vitamin D supplementation - Other co-interventions such as cysteamine therapy |  |
| Outcomes | - Rickets (clinical and biochemical markers) - Phosphate - Growth - Quality of life - Neurologic development and milestones - Disease progression - Adverse events (e.g. nephrocalcinosis, urolithiasis) - Alkaline phosphatase levels | - Studies not reporting on included outcomes |
| Geography | No limitations |  |
| Settings | - Any setting |  |
| Publication language | - English, German | - All other languages |
| Study design | - RCTs - Nonrandomized controlled trials - Cohort studies - Case-control studies - Cross-sectional studies - Before-after studies - Case series - Case reports - Systematic reviews and meta-analyses | - Nonsystematic reviews |
| Publication type | Any peer-reviewed publication reporting primary data | Abstracts, preprints, publications not reporting primary data (e.g. protocols) |

**KQ9: What is the effectiveness and safety of indomethacine therapy vs no indomethacine therapy on consequences of Fanconi syndrome in patients with cystinosis?**

**KQ9a: Are there any differences by age, until which age, dosage, combined administration of PPI and indomethacine, type of indomethacine (enteric/coated)?**

Table S10: Inclusion/exclusion criteria for KQ9/K9a

| Category | Criteria | |
| --- | --- | --- |
|  | Inclusion | Exclusion |
| Population | Patients with cystinosis and polyuria and hypokalemia | - Patients with cystinosis and without polyuria and hypokalemia - Patients with other rare diseases |
|  | Subgroups:   - age, until which age, dosage, type of indomethacin – enteric/coated |  |
| Intervention | - Indomethacin therapy - Indomethacin therapy combined with protein pump inhibitors (PPI) - Other co-interventions such as cysteamine therapy |  |
| Comparison | - No indomethacin therapy - Other co-interventions such as cysteamine therapy |  |
| Outcomes | - Dehydration episodes/polyuria - Quality of life - Serum levels of electrolytes - Growth - Rickets (clinical and biochemical markers) - Bicarbonate - Disease progression (deterioration of kidney function) - Adverse events (e.g. stomach ulcers) - Phosphate serum level - Hypokalemia - Neurologic development and milestones - Alkaline phosphate levels | - Any other outcomes |
| Geography | No limitations |  |
| Settings | - Any setting |  |
| Publication language | - English, German | - All other languages |
| Study design | - RCTs - Nonrandomized controlled trials - Cohort studies - Case-control studies - Cross-sectional studies - Before-after studies - Case series - Case reports - Systematic reviews and meta-analyses | - Nonsystematic reviews |
| Publication type | Any peer-reviewed publication reporting primary data | Abstracts, preprints, publications not reporting primary data (e.g. protocols) |

**KQ10: What is the effectiveness and safety of thiazide therapy plus bicarbonate/citrate compared with bicarbonate/citrate for treating metabolic acidosis in patients with cystinosis?**

**KQ10a: Are there any differences by population characteristic (e.g. severe acidosis), CKD stage?**

Table S11: Inclusion/exclusion criteria for KQ10/KQ10a

| Category | Criteria | |
| --- | --- | --- |
|  | Inclusion | Exclusion |
| Population | Patients with cystinosis and metabolic acidosis | - Patients with cystinosis and without and metabolic acidosis - Patients with other rare diseases |
|  | Subgroups:   - population characteristic (severe acidosis), CKD stage |  |
| Intervention | - Thiazide plus bicarbonate/citrate - Other co-interventions such as cysteamine therapy |  |
| Comparison | - Bicarbonate/citrate alone - Other co-interventions such as cysteamine therapy |  |
| Outcomes | - Adverse events - Quality of life - Severity of acidosis | - Any other outcomes |
| Geography | No limitations |  |
| Settings | - Any setting |  |
| Publication language | - English, German | - All other languages |
| Study design | - RCTs - Nonrandomized controlled trials - Cohort studies - Case-control studies - Cross-sectional studies - Before-after studies - Case series - Case reports - Systematic reviews and meta-analyses | - Nonsystematic reviews |
| Publication type | Any peer-reviewed publication reporting primary data | Abstracts, preprints, publications not reporting primary data (e.g. protocols) |

**KQ11: What is the effectiveness and safety of RAAS blockade (Angiotensin Converting Enzyme [ACE]-inhibitor/Angiotensin receptor blockers [ARB]) in patients with cystinosis compared to no intervention in terms of renal function?**

**KQ11a: Are there any differences age, CKD stage, age at initial treatment?**

**KQ12: What is the effectiveness and safety of RAAS blockade (ACE-inhibitor therapy/ARB) combined with indomethacine (other prostaglandin inhibitors) in patients with cystinosis compared to no intervention, RAAS blockade (ACE-inhibitor therapy/ARB) alone or indomethacine alone in terms of renal function?**

**KQ12a: Are there any differences age, CKD stage, age at initial treatment?**

Table S12: Inclusion/exclusion criteria for KQ11/KQ11a and KQ12/KQ12a

| Category | Criteria | |
| --- | --- | --- |
|  | Inclusion | Exclusion |
| Population | Patients with cystinosis | - Patients with other rare diseases |
|  | Subgroups:   - age, CKD stage, age at initial treatment |  |
| Intervention | - RAAS blockade (ACE inhibitor/ARB) [KQ11/KQ11a] - RAAS blockade (ACE-inhibitor therapy/ARB) combined with indomethacin (other prostaglandin inhibitors) [KQ12/12a] - Other co-interventions such as cysteamine therapy |  |
| Comparison | - No intervention [KQ11/KQ11a] [KQ12/12a] - RAAS blockade (ACE inhibitor/ARB) [KQ12/12a] - Indomethacin (other prostaglandin inhibitors) [KQ12/12a] - Other co-interventions such as cysteamine therapy |  |
| Outcomes | - Adverse events (including hyperkalemia, acute renal failure, hypotension, hypokalemia, ulcer, GI bleeding, hypertension) - Estimated glomerular filtration rate (eGFR) - Albuminuria - Quality of life | - Any other outcomes |
| Geography | No limitations |  |
| Settings | - Any setting |  |
| Publication language | - English, German | - All other languages |
| Study design | - RCTs - Nonrandomized controlled trials - Cohort studies - Case-control studies - Cross-sectional studies - Before-after studies - Case series - Case reports - Systematic reviews and meta-analyses | - Nonsystematic reviews |
| Publication type | Any peer-reviewed publication reporting primary data | Abstracts, preprints, publications not reporting primary data (e.g. protocols) |

*RAAS=* *Renin-Angiotensin-Aldosterone-System; ACE=Angiotensin Converting Enzyme; ARB=Angiotensin receptor blockers*

## **Kidney transplantation**

**KQ13: What is the effectiveness and safety of surgical/pharmacological nephrectomy vs. no nephrectomy in patients with cystinosis undergoing kidney transplantation?**

**KQ13: Are there any differences by age group, primary vs. second and subsequent transplant, timing of transplantation/nephrectomy, polyuria severity, preemptive transplantation?**

Table S13: Inclusion/exclusion criteria for KQ13/KQ13a

| Category | Criteria | |
| --- | --- | --- |
|  | Inclusion | Exclusion |
| Population | Patients with cystinosis undergoing kidney transplantation | - Patients with cystinosis not undergoing kidney transplantation - Patients with other rare diseases |
|  | Subgroups:   - age, primary vs. second and subsequent transplant, timing of transplantation/nephrectomy, polyuria severity, preemptive transplantation? |  |
| Intervention | - Surgical/pharmacological nephrectomy - Other co-interventions such as cysteamine therapy |  |
| Comparison | - No nephrectomy - Other co-interventions such as cysteamine therapy |  |
| Outcomes | - Persistence of FANCONI-syndrome - Transplant function - Adverse events (e.g. dehydration, acidosis, hypokalemia, hypophosphatemia) - Quality of life - Incidence of bone diseases - Survival - Dialysis incidence - Time until dialysis initiation - Burden of disease | - Any other outcomes |
| Geography | No limitations |  |
| Settings | - Any setting |  |
| Publication language | - English, German | - All other languages |
| Study design | - RCTs - Nonrandomized controlled trials - Cohort studies - Case-control studies - Cross-sectional studies - Before-after studies - Case series - Case reports - Systematic reviews and meta-analyses | - Nonsystematic reviews |
| Publication type | Any peer-reviewed publication reporting primary data | Abstracts, preprints, publications not reporting primary data (e.g. protocols) |

## **Bone disease**

**KQ14: What is the effectiveness and safety of phosphate and treatment with active vitamin D compared to phosphate alone in patients with cystinosis and Fanconi-syndrome on risk of hyperparathyroidism and persistent rickets/osteomalacia?**

**KQ14a: Are there any differences according to age, CKD stage, tailored vs. non-tailored management?**

Table S14: Inclusion/exclusion criteria for KQ14/KQ14a

| Category | Criteria | |
| --- | --- | --- |
|  | Inclusion | Exclusion |
| Population | Patients with cystinosis and Fanconi-Syndrome | - Patients with cystinosis and without FANCONI Syndrome - Patients with other rare diseases |
|  | Subgroups:   - age, CKD stage, tailored vs. non-tailored management |  |
| Intervention | - Phosphate and treatment with (active) vitamin D - Other co-interventions such as cysteamine therapy | - Multivitamin supplements beyond combination of phosphate and vitamin D |
| Comparison | - Phosphate supplementation alone - Other co-interventions such as cysteamine therapy | - Multivitamin supplements |
| Outcomes | - Persistence of FANCONI-syndrome - Transplant function - Bone deformity - Quality of life - Rate and severity of rickets/osteomalacia - Rate of fractures - Growth - Plasma calcium - Alkaline phosphatase levels - Radiologic evidence of active rickets - Peak or trough serum phosphate - Risk of hyperparathyroidism - Adverse events - Hypercalciuria | - Any other outcomes |
| Geography | No limitations |  |
| Settings | - Any setting |  |
| Publication language | - English, German | - All other languages |
| Study design | - RCTs - Nonrandomized controlled trials - Cohort studies - Case-control studies - Cross-sectional studies - Before-after studies - Case series - Case reports - Systematic reviews and meta-analyses | - Nonsystematic reviews |
| Publication type | Any peer-reviewed publication reporting primary data | Abstracts, preprints, publications not reporting primary data (e.g. protocols) |

**KQ15: What is the effectiveness and safety of calcium supplementation compared to no calcium in patients with Fanconi-syndrome on risk of hyperparathyroidism, and persistent rickets/osteomalacia?**

**KQ15a: Are there any differences according to age, CKD stage, tailored vs. non-tailored management?**

Table S15: Inclusion/exclusion criteria for KQ15/KQ15a

| Category | Criteria | |
| --- | --- | --- |
|  | Inclusion | Exclusion |
| Population | Patients with Fanconi-Syndrome | - Patients with or without cystinosis and without FANCONI Syndrome - Patients with other rare diseases |
|  | Subgroups:   - age, CKD stage, tailored vs. non-tailored management |  |
| Intervention | - Calcium supplementation - Other co-interventions such as cysteamine therapy | - Multivitamin supplements |
| Comparison | - No calcium supplementation - Other co-interventions such as cysteamine therapy |  |
| Outcomes | - Rate of fractures - Rate and severity of rickets/osteomalacia - Plasma calcium - Radiologic evidence of active rickets - Bone deformity - Growth impairment - Quality of life - Risk of hyperparathyroidism - Linear height - Hypercalciuria - Adverse events - Peak or trough serum phosphate - Hypercalciuria | - Any other outcomes |
| Geography | No limitations |  |
| Settings | - Any setting |  |
| Publication language | - English, German | - All other languages |
| Study design | - RCTs - Nonrandomized controlled trials - Cohort studies - Case-control studies - Cross-sectional studies - Before-after studies - Case series - Case reports - Systematic reviews and meta-analyses | - Nonsystematic reviews |
| Publication type | Any peer-reviewed publication reporting primary data | Abstracts, preprints, publications not reporting primary data (e.g. protocols) |

**KQ16: Does early cysteamine treatment compared to late cysteamine treatment affect growth impairment and risk of neurological symptoms in infants, children, and adolescents with cystinosis?**

Table S16: Inclusion/exclusion criteria for KQ16

| Category | Criteria | |
| --- | --- | --- |
|  | Inclusion | Exclusion |
| Population | Infants, children, adolescents with cystinosis | - Adults with cystinosis - Patients with other rare diseases |
| Intervention | - Early cysteamine treatment (as defined by the studies) |  |
| Comparison | - Late cysteamine treatment (as defined by the studies) |  |
| Outcomes | - Renal function (CKD stage) - Quality of life - Growth - Adverse events - Intellectual function - Risk of visual spatial problems - Motor coordination - Extra-renal complications | - Any other outcomes |
| Geography | No limitations |  |
| Settings | - Any setting |  |
| Publication language | - English, German | - All other languages |
| Study design | - RCTs - Nonrandomized controlled trials - Cohort studies - Case-control studies - Cross-sectional studies - Before-after studies - Case series - Case reports - Systematic reviews and meta-analyses | - Nonsystematic reviews |
| Publication type | Any peer-reviewed publication reporting primary data | Abstracts, preprints, publications not reporting primary data (e.g. protocols) |

## **Endocrinological disorders**

**KQ17: What is the effectiveness and safety of testosterone replacement therapy compared to no testosterone replacement therapy on health outcomes in adolescent and adult males with endocrine deficits (i.e. hypogonadism) and low testosterone levels?**

**KQ17a: Are there any differences for patients with hypergonadotropic hypogonadism compared to those without?**

Table S17: Inclusion/exclusion criteria for KQ17/KQ17a

| Category | Criteria | |
| --- | --- | --- |
|  | Inclusion | Exclusion |
| Population | Adolescent and adult males with hypogonadism and low testosterone levels | - Adolescent and adult females with endocrine deficits - Children with cystinosis or endocrine deficits - Adolescent and adult males with endocrine deficits but normal testosterone levels |
|  | Subgroups:   - hypergonadotropic hypogonadism |  |
| Intervention | - Testosterone replacement therapy - Other co-interventions | - Other hormonal therapy |
| Comparison | - No testosterone replacement therapy - Other co-interventions | - Other hormonal therapy |
| Outcomes | - Mood (depression) - Well-being - Muscle strength - Quality of life - Libido - Erectile function - Sexual competence - Adverse events - Sleep disorders - Fertility - Osteoporosis | - Any other outcomes |
| Geography | No limitations |  |
| Settings | - Any setting |  |
| Publication language | - English, German | - All other languages |
| Study design | - RCTs - Nonrandomized controlled trials - Cohort studies - Case-control studies - Cross-sectional studies - Before-after studies - Case series - Case reports - Systematic reviews and meta-analyses | - Nonsystematic reviews |
| Publication type | Any peer-reviewed publication reporting primary data | Abstracts, preprints, publications not reporting primary data (e.g. protocols) |

**KQ18: Does cystinosis with CKD compared to CKD and no cystinosis affect maternal and child health in pregnant women?**

Table S18: Inclusion/exclusion criteria for KQ18

| Category | Criteria | |
| --- | --- | --- |
|  | Inclusion | Exclusion |
| Population | Pregnant women with CKD | - Other patients with CKD |
| Exposure | - With cystinosis | - Any other disease |
| Comparison | - Without cystinosis | - Any other disease |
| Outcomes | - Progression of cystinosis and CKD - Pregnancy success rate - Pregnancy and birth complications (miscarriage, gestational diabetes, preeclampsia, cesarean section,…) - Prematurity - SGA (small for gestational age) - Child development - Stillbirth - Child ICU admission - Neonatal death | - Any other outcomes |
| Geography | No limitations |  |
| Settings | - Any setting |  |
| Publication language | - English, German | - All other languages |
| Study design | - Cohort studies - Case-control studies - Cross-sectional studies - Before-after studies - Case series - Case reports - Systematic reviews and meta-analyses | - Nonsystematic reviews |
| Publication type | Any peer-reviewed publication reporting primary data | Abstracts, preprints, publications not reporting primary data (e.g. protocols) |

**KQ19: Does cysteamine therapy in pregnancy compared to no cysteamine therapy affect maternal and child health in women with cystinosis?**

Table S19: Inclusion/exclusion criteria for KQ19

| Category | Criteria | |
| --- | --- | --- |
|  | Inclusion | Exclusion |
| Population | Pregnant women with cystinosis and CKD | - Other patients with CKD or cystinosis |
| Intervention | - Cysteamine therapy | - Any other intervention |
| Comparison | - No Cysteamine therapy | - Any other intervention |
| Outcomes | - Progression of cystinosis and CKD - Pregnancy success rate - Pregnancy and birth complications (miscarriage, gestational diabetes, preeclampsia, cesarean section,…) - Prematurity - SGA (small for gestational age) - Child development - Stillbirth - Child ICU admission - Neonatal death - Fetotoxicity (e.g. malformations) | - Any other outcomes |
| Geography | No limitations |  |
| Settings | - Any setting |  |
| Publication language | - English, German | - All other languages |
| Study design | - RCTs - Nonrandomized controlled trials - Cohort studies - Case-control studies - Cross-sectional studies - Before-after studies - Case series - Case reports - Systematic reviews and meta-analyses | - Nonsystematic reviews |
| Publication type | Any peer-reviewed publication reporting primary data | Abstracts, preprints, publications not reporting primary data (e.g. protocols) |

## **Gastrointestinal disorders**

**KQ20: What is the effectiveness and safety of additional PPI (proton pump inhibitors) to cysteamine therapy compared to cysteamine therapy alone on health outcomes/ adverse events in patients with cystinosis?**

**KQ20a: Are there any differences by age, presence of gastric acid hypersecretion or acid reflux?**

Table S20: Inclusion/exclusion criteria for KQ20/KQ20a

| Category | Criteria | |
| --- | --- | --- |
|  | Inclusion | Exclusion |
| Population | Patients with cystinosis | - Patients with other rare diseases |
|  | Subgroups:   - age, presence gastric acid hypersecretion or acid reflux yes/no |  |
| Intervention | - Cysteamine therapy plus additional PPI (proton pump inhibitors) - Other co-interventions |  |
| Comparison | - Cysteamine therapy - Other co-interventions |  |
| Outcomes | - Quality of life - Heart burn - Rate of gastric ulcers - Nausea - GI perforation - Vomiting - Appetite - Weight gain/BMI - Adverse events - Halitosis - Cystine level in leukocytes | - Any other outcomes |
| Geography | No limitations |  |
| Settings | - Any setting |  |
| Publication language | - English, German | - All other languages |
| Study design | - RCTs - Nonrandomized controlled trials - Cohort studies - Case-control studies - Cross-sectional studies - Before-after studies - Case series - Case reports - Systematic reviews and meta-analyses | - Nonsystematic reviews |
| Publication type | Any peer-reviewed publication reporting primary data | Abstracts, preprints, publications not reporting primary data (e.g. protocols) |

## **Muscle weakness**

**KQ21: What is the effectiveness and safety of L- carnitine and/or Co-Enzyme Q10 supplementation compared to no supplementation on muscle function in patients with cystinosis?**

**KQ21a: Are there any differences by age, dosage?**

Table S21: Inclusion/exclusion criteria for KQ21/KQ21a

| Category | Criteria | |
| --- | --- | --- |
|  | Inclusion | Exclusion |
| Population | Patients with cystinosis | - Patients with other rare diseases |
|  | Subgroups:   - age, dosage |  |
| Intervention | - L- carnitine and/or Co-Enzyme Q10 supplementation - Other co-interventions | - Multivitamin supplements |
| Comparison | - No supplementation - Other co-interventions | - Multivitamin supplements |
| Outcomes | - Muscle strength - Participation in professional and social life - Quality of life - Muscle function - Physical functioning - Respiratory function - Adverse events | - Any other outcomes |
| Geography | No limitations |  |
| Settings | - Any setting |  |
| Publication language | - English, German | - All other languages |
| Study design | - RCTs - Nonrandomized controlled trials - Cohort studies - Case-control studies - Cross-sectional studies - Before-after studies - Case series - Case reports - Systematic reviews and meta-analyses | - Nonsystematic reviews |
| Publication type | Any peer-reviewed publication reporting primary data | Abstracts, preprints, publications not reporting primary data (e.g. protocols) |

**KQ22: What is the effectiveness and safety of preventive physiotherapy (i.e. strength training) compared to no physiotherapy on muscle function in patients with cystinosis?**

**KQ22a: Are there any differences by age?**

Table S22: Inclusion/exclusion criteria for KQ22/KQ22a

| Category | Criteria | |
| --- | --- | --- |
|  | Inclusion | Exclusion |
| Population | Patients with cystinosis | - Patients with other rare diseases |
|  | Subgroups:   - age |  |
| Intervention | - Preventive physiotherapy (i.e. strength training) - Other co-interventions | - Curative physiotherapy |
| Comparison | - No physiotherapy - Other co-interventions |  |
| Outcomes | - Muscle strength - Participation in professional and social life - Quality of life - Muscle function - Physical functioning - Respiratory function - Adverse events | - Any other outcomes |
| Geography | No limitations |  |
| Settings | - Any setting |  |
| Publication language | - English, German | - All other languages |
| Study design | - RCTs - Nonrandomized controlled trials - Cohort studies - Case-control studies - Cross-sectional studies - Before-after studies - Case series - Case reports - Systematic reviews and meta-analyses | - Nonsystematic reviews |
| Publication type | Any peer-reviewed publication reporting primary data | Abstracts, preprints, publications not reporting primary data (e.g. protocols) |

## **Neurological disorders**

**KQ23: What is the effectiveness and safety of early imaging or psychological or sensomotoric testing compared to no intervention on health outcomes in patients with cystinosis?**

Table S23: Inclusion/exclusion criteria for KQ23

| Category | Criteria | |
| --- | --- | --- |
|  | Inclusion | Exclusion |
| Population | Patients with cystinosis | - Patients with other rare diseases |
| Intervention | - Early imaging (MRI or CT) or psychological or sensomotoric testing - Other co-interventions |  |
| Comparison | - No intervention - Other co-interventions |  |
| Outcomes | - Vomiting - Headache - Seizures - Reduced intracranial pressure - Optic nerve changes - Participation in social and professional life - Quality of life | - Any other outcomes |
| Geography | No limitations |  |
| Settings | - Any setting |  |
| Publication language | - English, German | - All other languages |
| Study design | - RCTs - Nonrandomized controlled trials - Cohort studies - Case-control studies - Cross-sectional studies - Before-after studies - Case series - Case reports - Systematic reviews and meta-analyses | - Nonsystematic reviews |
| Publication type | Any peer-reviewed publication reporting primary data | Abstracts, preprints, publications not reporting primary data (e.g. protocols) |

**KQ24: What is the effectiveness and safety of occupational therapy, educational interventions, behavioral interventions or psychotherapy) compared to no interventions on neurological outcomes in patients with cystinosis?**

**KQ24a: Are there any differences by age, cognitive function?**

Table S24: Inclusion/exclusion criteria for KQ24/KQ24a

| Category | Criteria | |
| --- | --- | --- |
|  | Inclusion | Exclusion |
| Population | Patients with cystinosis | - Patients with other rare diseases |
|  | Subgroups:   - age, cognitive function |  |
| Intervention | - Occupational therapy - Educational interventions - Behavioral interventions - Psychotherapy - Other co-interventions |  |
| Comparison | - No intervention - Other co-interventions |  |
| Outcomes | - Participation in social and professional life - Quality of life - School performance (e.g. special needs education, high school graduation) - Treatment adherence - Neurological outcomes - Disease progression - Adverse events | - Any other outcomes |
| Geography | No limitations |  |
| Settings | - Any setting |  |
| Publication language | - English, German | - All other languages |
| Study design | - RCTs - Nonrandomized controlled trials - Cohort studies - Case-control studies - Cross-sectional studies - Before-after studies - Case series - Case reports - Systematic reviews and meta-analyses | - Nonsystematic reviews |
| Publication type | Any peer-reviewed publication reporting primary data | Abstracts, preprints, publications not reporting primary data (e.g. protocols) |

## **Eye disease**

**KQ25: What is the effectiveness and safety of combined treatment of cysteamine with concomitant cysteamine eye drops compared to systemic cysteamine treatment alone on vision loss and photophobia in patients with cystinosis?**

**KQ25a: Are there any differences by age, start of treatment, type of eye drops (Cystadrops, Cystern, made-up drops), dosage, frequency of administration?**

Table S25: Inclusion/exclusion criteria for KQ25/KQ25a

| Category | Criteria | |
| --- | --- | --- |
|  | Inclusion | Exclusion |
| Population | Patients with cystinosis | - Patients with other rare diseases |
|  | Subgroups:   - age, start of treatment, type of eye drops (Cystadrops, Cystern, made-up drops), dosage, frequency of administration |  |
| Intervention | - Systemic cysteamine with concomitant cysteamine eye drops - Other co-interventions |  |
| Comparison | - Systemic cysteamine - Other co-interventions |  |
| Outcomes | - Vision loss - Photophobia - Neovascularization of the cornea, need for corneal transplantation - Quality of life - Ocular surface disease - Itchy eyes - Adherence - Dry eye - Adverse events | - Any other outcomes |
| Geography | No limitations |  |
| Settings | - Any setting |  |
| Publication language | - English, German | - All other languages |
| Study design | - RCTs - Nonrandomized controlled trials - Cohort studies - Case-control studies - Cross-sectional studies - Before-after studies - Case series - Case reports - Systematic reviews and meta-analyses | - Nonsystematic reviews |
| Publication type | Any peer-reviewed publication reporting primary data | Abstracts, preprints, publications not reporting primary data (e.g. protocols) |

**KQ26: What is the effectiveness and safety of systemic cysteamine treatment compared to no treatment on vision loss and photophobia in patients with cystinosis?**

**KQ26a: Are there any differences by age, start of treatment, type of eye drops, dosage, frequency of administration?**

Table S26: Inclusion/exclusion criteria for KQ26/KQ26a

| Category | Criteria | |
| --- | --- | --- |
|  | Inclusion | Exclusion |
| Population | Patients with cystinosis | - Patients with other rare diseases |
|  | Subgroups:   - age, start of treatment, type of eye drops, dosage, frequency of administration |  |
| Intervention | - Systemic cysteamine treatment - Other co-interventions |  |
| Comparison | - No systemic cysteamine treatment - Other co-interventions |  |
| Outcomes | - Vision loss - Retinal pigment epithelium (RPE) changes - Visual field loss - Maculopathy - Quality of life - Intracranial changes - Optic nerve changes - Neovascularization of the cornea - Adverse events - Photophobia - Electroretinogram (ERG) changes - Adherence - Crystal load in retina - Need for corneal transplantation | - Any other outcomes |
| Geography | No limitations |  |
| Settings | - Any setting |  |
| Publication language | - English, German | - All other languages |
| Study design | - RCTs - Nonrandomized controlled trials - Cohort studies - Case-control studies - Cross-sectional studies - Before-after studies - Case series - Case reports - Systematic reviews and meta-analyses | - Nonsystematic reviews |
| Publication type | Any peer-reviewed publication reporting primary data | Abstracts, preprints, publications not reporting primary data (e.g. protocols) |

**KQ27: What is the adherence and safety of cysteamine eye drops with benzalkonium chloride compared to cysteamine eye drops without benzalkonium chloride on adverse events in patients with lifelong need for eye therapy?**

**KQ27a: Are there any differences by age?**

Table S27: Inclusion/exclusion criteria for KQ27/KQ27a

| Category | Criteria | |
| --- | --- | --- |
|  | Inclusion | Exclusion |
| Population | Patients with lifelong need for eye therapy (starting from childhood) | - Patients with short term need for eye therapy (e.g. after surgery) |
|  | Subgroups:   - age |  |
| Intervention | - Any eye drops with benzalkonium chloride - Other co-interventions |  |
| Comparison | - Eye drops without benzalkonium chloride - Other co-interventions |  |
| Outcomes | - Adherence - Quality of life - Adverse events - Obstacles/access to care | - Any other outcomes |
| Geography | No limitations |  |
| Settings | - Any setting |  |
| Publication language | - English, German | - All other languages |
| Study design | - RCTs - Nonrandomized controlled trials - Cohort studies - Case-control studies - Cross-sectional studies - Before-after studies - Case reports - Systematic reviews and meta-analyses | - Nonsystematic reviews |
| Publication type | Any peer-reviewed publication reporting primary data | Abstracts, preprints, publications not reporting primary data (e.g. protocols) |

**KQ28: What is the accuracy, reliability, repeatability of Optical Coherence Tomography (OCT) or corneal densitometry or slit lamp photography compared to in vivo confocal microscopy (IVCM) to monitor corneal cystine crystals in patients with cystinosis?**

Table S28: Inclusion/exclusion criteria for KQ28

| Category | Criteria | |
| --- | --- | --- |
|  | Inclusion | Exclusion |
| Population | Patients with cystinosis | - Patients with other rare diseases |
| Index test | - Optical Coherence Tomography (OCT) - Corneal densitometry - Slit lamp photography |  |
| Reference test | - In vivo confocal microscopy (IVCM) |  |
| Outcomes | - Dynamics of crystal deposition or resolution under treatment - Crystal load - Reliability - Distribution of crystals - Repeatability - Cornea thinning - Photophobia - Depth of crystal deposition in corneas - Opacification - Accuracy - Ulcer - Vision loss | - Any other outcomes |
| Geography | No limitations |  |
| Settings | - Any setting |  |
| Publication language | - English, German | - All other languages |
| Study design | - RCTs - Nonrandomized controlled trials - Cohort studies - Case-control studies - Cross-sectional studies - Descriptive studies without a control group - Case series - Case reports - Systematic reviews and meta-analyses | - Nonsystematic reviews |
| Publication type | Any peer-reviewed publication reporting primary data | Abstracts, preprints, publications not reporting primary data (e.g. protocols) |

## **Interdisciplinary care**

**KQ29: What is the effectiveness and safety of integrated care compared to standard care on health outcomes and adherence in patients with rare multi-organ disease?**

**KQ29a: Are there any differences by age?**

Table S29: Inclusion/exclusion criteria for KQ29/KQ29a

| Category | Criteria | |
| --- | --- | --- |
|  | Inclusion | Exclusion |
| Population | Patients with the following rare multi-organ disease:   - Cystinosis - Collagenoses - Mitochondropathies - Mixed connective tissue - Mucoviscidosis - Fabry disease - Hyperoxaluria - Neurofibromatosis (NF1)* - M. Gaucher - M. Pompe* - Coenzyme Q 10 deficiency* - Epidermolysis bullosa - Alkaptonuria* | - Patients with other rare diseases |
|  | Subgroup:   - Age |  |
| Intervention | - Integrated care (1 coordinator, at least two different professions) |  |
| Comparison | - Standard care |  |
| Outcomes | - Disease outcome (kidney and transplant function, extrarenal complications (ophthalmology, growth, pulmonary function, fertility, endocrinology, neurology, muscle involvement (e.g. swallowing), somatic and laboratory parameters (malnutrition)) - Quality of life (physical and psychological QoL) - Quality of care - Treatment adherence - Social and professional participation (friends, school graduation, active in work live) - Survival - Adverse events | - Any other outcomes |
| Geography | No limitations |  |
| Settings | Any setting |  |
| Publication language | - English, German | - All other languages |
| Study design | - RCTs - Nonrandomized controlled trials - Cohort studies - Case-control studies - Cross-sectional studies - Before-after studies - Case series - Case reports - Systematic reviews and meta-analyses | - Nonsystematic reviews |
| Publication type | Any peer-reviewed publication reporting primary data | Abstracts, preprints, publications not reporting primary data (e.g. protocols) |

* change of eligible populations after protocol was published

## **Transition of care**

**KQ30: What is the effectiveness and safety of interventions built on a transition of care model compared to other or no interventions on adherence and psychosocial outcomes in adolescence with rare multi-organ disease and their parents/legal guardians?**

**KQ30a: Are there any differences by age at start of program, pre-/post—kidney transplantation?**

Table S30: Inclusion/exclusion criteria for KQ30/KQ30a

| Category | Criteria | |
| --- | --- | --- |
|  | Inclusion | Exclusion |
| Population | Patients with the following rare multi-organ disease and their legal guardians:   - Cystinosis - Collagenoses - Mitochondropathies - Mixed connective tissue - Mucoviscidosis - Fabry disease - Hyperoxaluria - Neurofibromatosis (NF1)* - M. Gaucher - M. Pompe* - Coenzyme Q 10 deficiency* - Epidermolysis bullosa - Alkaptonuria* | - Patients with other rare diseases |
|  | Subgroup:   - Age at start of program, pre-/post—kidney transplantation |  |
| Intervention | - Interventions built on transition of care models |  |
| Comparison | - Other or no interventions |  |
| Outcomes | - Disease management/literacy (multiorgan disease) - Graft loss - Treatment adherence (systemic and local) - Survival - Quality of life - Psychosocial outcomes (parents and adolescents) - Loss to follow-up (long term outcome) - Rate of regular monitoring - eGFR - Education and employment outcomes - Frequency of visits - Hospital admissions - Cystine level - Adverse events - Malnutrition | - Any other outcomes |
| Geography | No limitations |  |
| Settings | - Any setting |  |
| Publication language | - English, German | - All other languages |
| Study design | - RCTs - Nonrandomized controlled trials - Cohort studies - Case-control studies - Cross-sectional studies - Before-after studies - Case series - Case reports - Systematic reviews and meta-analyses - Evidence-based guidelines | - Nonsystematic reviews |
| Publication type | Any peer-reviewed publication reporting primary data | Abstracts, preprints, publications not reporting primary data (e.g. protocols) |

* change of eligible populations after protocol was published

## **Psychosocial support**

**KQ31: What is the efficacy and safety of interventions for psychosocial support compared to no psychosocial support to improve psychosocial well-being in patients with rare multi-organ diseases and caregivers?**

**KQ31a: Are there any differences by age, start of intervention, type, and mode of intervention?**

Table S31: Inclusion/exclusion criteria for KQ31/KQ31a

| Category | Criteria | |
| --- | --- | --- |
|  | Inclusion | Exclusion |
| Population | Patients with the following rare multi-organ disease:   - Cystinosis - Collagenoses - Mitochondropathies - Mixed connective tissue - Mucoviscidosis - Fabry disease - Hyperoxaluria - Neurofibromatosis (NF1)* - M. Gaucher - M. Pompe* - Coenzyme Q 10 deficiency* - Epidermolysis bullosa - Alkaptonuria* | - Patients with other rare diseases |
|  | Subgroup:   - Age, start of intervention, type, and mode of intervention |  |
| Intervention | - Psychosocial support (patient advocacy groups, psychoeducation, counseling, social pediatric care) |  |
| Comparison | - Other or no interventions |  |
| Outcomes | - Resilience factors/protective factors of patients and caregivers - Adherence - Quality of life - Educational/vocational outcomes in adulthood, educational outcomes in childhood/adolescence - Psychosocial well-being - Incidence or severity of psychosocial disorders | - Any other outcomes |
| Geography | No limitations |  |
| Settings | - Any setting |  |
| Publication language | - English, German | - All other languages |
| Study design | - RCTs - Nonrandomized controlled trials - Cohort studies - Case-control studies - Cross-sectional studies - Before-after studies - Case series - Case reports - Systematic reviews and meta-analyses - Evidence-based guidelines | - Nonsystematic reviews |
| Publication type | Any peer-reviewed publication reporting primary data | Abstracts, preprints, publications not reporting primary data (e.g. protocols) |

* change of eligible populations after protocol was published

# Search Strategies

| Search* | Database name, time span, and host | Date searched | Hits |
| --- | --- | --- | --- |
| Round 1: Bibliographic databases | Ovid MEDLINE(R) ALL 1946 to April 24, 2023 | 25.Apr.23 | 1433 |
|  | Embase.com (Elsevier) | 25.Apr.23 | 1761 |
|  | Cochrane Central Register of Controlled Trials Issue 4 of 12, April 2023 (Cochrane Library/Wiley) | 25.Apr.23 | 38 |
|  | CINAHL (Ebsco) | 25.Apr.23 | 198 |
|  | Dissertations & Theses Global (ProQuest) | 25.Apr.23 | 451 |
|  | BASE (https://www.base-search.net) | 25.Apr.23 | 65 |
|  |  | **Total (before deduplication)** | **3946** |
|  |  | **Total (after deduplication)** | **2387** |
| Search* | Database name, time span, and host | Date searched | Hits |
| Round 1: Study registers | ClinicalTrials.gov | 25.Apr.23 | 35 |
|  | WHO International Clinical Trials Registry Platform (ICTRP) (https://trialsearch.who.int/) | 25.Apr.23 | 42 |
|  |  | **Total (before deduplication)** | **77** |
|  |  | **Total (after deduplication)** | **53** |
|  |  |  |  |
| Round 2: Guidelines/SRs rare diseases | Ovid MEDLINE(R) ALL 1946 to June 29, 2023 | 3 July 2023 | 617 |
|  | Cochrane Database of Systematic Reviews Issue 7 of 12, July 2023 (Cochrane Library/Wiley) | 3 July 2023 | 25 |
|  | Epistemonikos.org | 3 July 2023 | 619 |
|  | TRIP (tripdatabase.com/) | 3 July 2023 | 135 |
|  | AWMF Leitlinienregister (register.awmf.org/) | 3 July 2023 | 194 |
|  | Guidelines International Network (GIN) Library (guidelines.ebmportal.com) | 3 July 2023 | 24 |
|  | NCBI Bookshelf (https://www.ncbi.nlm.nih.gov/books/) | 4 July 2023 | 2 |
|  |  | **Total (before deduplication)** | **1616** |
|  |  | **Total (after deduplication)** | **968** |
|  |  |  |  |
| Round 3: eyedrops, Fanconi | Ovid MEDLINE(R) ALL 1946 to July 06, 2023 | 10 July 2023 | 130 |
|  | Cochrane Library (Cochrane Central Register of Controlled Trials, Cochrane Database of Systematic Reviews) | 10 July 2023 | 6 |
|  | Epistemonikos.org | 10 July 2023 | 15 |
|  |  | **Total (before deduplication)** | **151** |
|  |  | **Total (after deduplication)** | **138** |
|  |  |  |  |
| Additional searches | Guidelines - CKD & Kidney transplant -Targeted search for guidelines from specific providers | 29.Jun.23 | 21 |

## **Round 1**

Ovid MEDLINE(R) ALL 1946 to April 24, 2023

| **#** | **Searches** | **Results** |
| --- | --- | --- |
| 1 | Cystinosis/ | 1352 |
| 2 | Cystinosis.ti,ab,kf. | 1465 |
| 3 | cystinotic.ti,ab,kf. | 224 |
| 4 | (Cystine disease or Cystine storage disease).ti,ab,kf. | 20 |
| 5 | Cystine diathesis.ti,ab,kf. | 2 |
| 6 | (defect adj3 (Cystinosin or Cystine Transport)).ti,ab,kf. | 16 |
| 7 | or/1-6 | 1706 |
| 8 | limit 7 to "humans only (removes records about animals)" | 1619 |
| 9 | (english or german).lg. | 31705094 |
| 10 | 8 and 9 | 1433 |

Embase.com (Elsevier) 25.04.2023

| **No.** | **Query** | **Results** |
| --- | --- | --- |
| #1 | 'cystinosis'/exp | 2268 |
| #2 | cystinosis:ti,ab,kw | 1961 |
| #3 | cystinotic:ti,ab,kw | 305 |
| #4 | 'cystine disease':ti,ab,kw OR 'cystine storage disease':ti,ab,kw | 19 |
| #5 | 'cystine diathesis':ti,ab,kw | 0 |
| #6 | (defect NEAR/3 (cystinosin OR 'cystine transport')):ti,ab,kw | 22 |
| #7 | #1 OR #2 OR #3 OR #4 OR #5 OR #6 | 2561 |
| #8 | ('animal'/exp OR 'animal model'/exp OR 'animal experiment'/de) NOT 'human'/exp | 6140167 |
| #9 | #7 NOT #8 | 2401 |
| #10 | #9 NOT ('conference abstract'/it OR 'conference paper'/it OR 'conference review'/it OR 'preprint'/it) | 1997 |
| #11 | #10 AND ([english]/lim OR [german]/lim) | 1761 |

Cochrane Central Register of Controlled Trials Issue 4 of 12, April 2023

| ID | Search | Hits |
| --- | --- | --- |
| #1 | [mh ^Cystinosis] | 26 |
| #2 | Cystinosis:ti,ab,kw | 45 |
| #3 | cystinotic:ti,ab,kw | 3 |
| #4 | ("Cystine disease":ti,ab,kw OR "Cystine storage disease":ti,ab,kw) | 0 |
| #5 | Cystine diathesis:ti,ab,kw | 0 |
| #6 | (defect?:ti,ab,kw NEAR/3 (Cystinosin:ti,ab,kw OR "Cystine Transport":ti,ab,kw)) | 0 |
| #7 | (or #1-#6) | 45 |
| #8 | Conference proceeding:pt or abstract:so | 218535 |
| #9 | ((language next (afr or ara or aze or bos or bul or car or cat or chi or cze or dan or dut or es or est or fin or fre or gre or heb or hrv or hun or ice or ira or ita or jpn or ko or kor or lit or nor or peo or per or pol or por or pt or rom or rum or rus or slo or slv or spa or srp or swe or tha or tur or ukr or urd or uzb)) not (language near/2 (en or eng or english or ger or german or mul or unknown))) | 92049 |
| #10 | #7 not (#8 or #9) in Trials | 38 |

CINAHL (Ebsco) 25.04.2023

| # | Query | Limiters/Expanders | Results |
| --- | --- | --- | --- |
| S1 | Cystinosis OR cystinotic OR "Cystine disease" OR "Cystine storage disease" OR "Cystine diathesis" | Search modes - Find all my search terms | 211 |
| S2 | (defect# N3 (Cystinosin OR "Cystine Transport")) | Search modes - Find all my search terms | 2 |
| S3 | S1 OR S2 | Search modes - Find all my search terms | 211 |
| S4 | (MH animals+ OR MH ("animal studies") OR TI ("animal model*")) NOT MH (human) | Search modes - Find all my search terms | 211,572 |
| S5 | S3 NOT S4 | Limiters - Language: English, German | 198 |

ProQuest Dissertations & Theses Global 25.04.2023

| Set# | Searched for | Results |
| --- | --- | --- |
| S1 | (Cystinosis OR cystinotic) AND la.exact("German" OR "English") | 451 |

BASE (<https://www.base-search.net>) 25.04.2023

| Field | Search terms | Document type | Results |
| --- | --- | --- | --- |
| Entire Document | (Cystinosis cystinotic) | Thesis | 65 |

ClinicaTrials.gov 25.04.2023

| Search | Results |
| --- | --- |
| Other terms: Cystinosis OR cystinotic | 35 |

WHO International Clinical Trials Registry Platform (ICTRP) 25.04.2023

| Search | Results |
| --- | --- |
| Cystinosis OR cystinotic | 42 |

## **Round 2**

Ovid MEDLINE(R) ALL 1946 to July 03, 2023

|  | **#** | **Searches** | **Results** |
| --- | --- | --- | --- |
| **A. rare diseases** | 1 | Rare Diseases/ | 13899 |
|  | 2 | ((rare or orphan) adj (disease? or disorder? or syndrome? or condition?)).ti,bt,kf. | 8030 |
|  | 3 | ((rare or orphan) adj3 (multi-organ* or multiorgan* or systemic or multisystem* or multi-system* or genetic or hereditary or inherited or metabolic or endocrin*) adj3 (disease? or disorder? or syndrome? or condition?)).ti,bt,kf. | 915 |
|  | 4 | Cystic Fibrosis/ | 39862 |
|  | 5 | (Cystic fibrosis or Mucoviscidosis or pancreatic fibrosis).ti,bt,ab,kf. | 52731 |
|  | 6 | Fabry disease/ | 4111 |
|  | 7 | (Fabry disease or Fabry's disease or angiokeratoma corporis diffusum or alpha-galactosidase A deficiency or GLA deficiency or angiokeratoma diffuse).ti,bt,ab,kf. | 4970 |
|  | 8 | Hyperoxaluria, Primary/ | 984 |
|  | 9 | (Hyperoxaluria or Oxalosis).ti,bt,ab,kf. | 3321 |
|  | 10 | neurofibromatoses/ or neurofibromatosis 1/ | 12089 |
|  | 11 | (Neurofibromatosis or von Recklinghausen's disease or von Recklinghausen disease or von Recklinghausen's syndrome or von Recklinghausen syndrome).ti,bt,ab,kf. | 17674 |
|  | 12 | Gaucher Disease/ | 5001 |
|  | 13 | (Gaucher disease or Gaucher's disease or cerebroside lipidosis syndrome or Gaucher splenomegaly or glucocerebrosidase deficiency or glucocerebrosidosis or glucosylceramidase deficiency or glucosyl cerebroside lipidosis or kerasin lipoidosis or kerasin thesaurismosis or (lipid histiocytosis adj1 kerasin type) or "sphingolipidosis 1").ti,bt,ab,kf. | 5519 |
|  | 14 | Glycogen Storage Disease Type II/ | 1943 |
|  | 15 | (Pompe disease or Pompe's disease or (Glycogen storage disease adj1 "type II") or acid maltase deficiency or (Glycogenosis adj1 "type II") or acid alpha-glucosidase deficiency).ti,bt,ab,kf. | 2540 |
|  | 16 | Ubiquinone/df [Deficiency] | 307 |
|  | 17 | (Coenzyme Q10 deficiency or CoQ10 deficiency or (Leigh syndrome adj2 nephro*)).ti,bt,ab,kf. | 316 |
|  | 18 | exp Epidermolysis Bullosa/ | 5620 |
|  | 19 | (Epidermolysis bullosa or Kindler's syndrome or Kindler syndrome or Acrokeratotic poikiloderma or (Congenital poikiloderma adj2 (blisters or keratoses or bullae or cutaneous atrophy)) or Hereditary acrokeratotic poikiloderma or (Hyperkeratosis adj1 hyperpigmentation)).ti,bt,ab,kf. | 6341 |
|  | 20 | Alkaptonuria/ | 1094 |
|  | 21 | (Alkaptonuria or alcaptonuria or Black urine disease or black bone disease).ti,bt,ab,kf. | 1173 |
|  | 22 | or/1-21 | 121883 |
| **B. hypogonadism** | 23 | Hypogonadism/ | 9672 |
|  | 24 | (hypogonadism or hypogonadal).ti,bt,ab,kf. | 14357 |
|  | 25 | ((endocrine adj (deficit? or deficien*)) and (testosterone adj1 (low or deficien* or insufficien*))).ti,bt,ab,kf. | 3 |
|  | 26 | or/23-25 | 17298 |
| **C. Testosterone replacement** | 27 | Hormone Replacement Therapy/ and testosterone.mp. | 2230 |
|  | 28 | (Testosterone adj (replacement or treatment or therapy)).ti,bt,ab,kf. | 5894 |
|  | 29 | 27 or 28 | 6994 |
| **B+C** | 30 | 26 and 29 | 2383 |
| **D. disease management** | 31 | (care or healthcare or Management or ((treatment or therapy) adj2 (diagnosis or evaluation)) or support).ti,bt. | 1257406 |
| **A+D** | 32 | 22 and 31 | 4327 |
| **E. occupational, psychological, educational, behavioral interventions & social support** | 33 | exp Psychotherapy/ | 218403 |
|  | 34 | Occupational Therapy/ | 14848 |
|  | 35 | Patient Education as Topic/ | 88288 |
|  | 36 | Health Education/ | 63660 |
|  | 37 | exp Social Support/ | 79776 |
|  | 38 | ((occupational or education* or behavio* or cognitive or psycholog* or psychosocial or psychoanlay* or self-care or self-efficacy or knowledge or information or Self-management or Psychodynamic or coaching or coping or intellectual capacity or positive affect or resilience or biopsychosocial or social) adj2 (Intervention? or Therap* or Treatment? or Program* or care or support)).ti,bt,ab,kf. | 379197 |
|  | 39 | (psychotherap* or "Acceptance and Commitment" or relaxation training or relaxation treatment? or Relaxation technique? or coping skills training or Problem solving or autogenic feedback or Biofeedback or patient education or health education or finding meaning or Active techniques or Cognitive restructuring or Communication development or distress reduction or Goal setting or Problem-solving or social worker? or (uncertainty adj1 manag*) or patient advocacy or counsel?ing or psychoeducation).ti,bt,ab,kf. | 288109 |
|  | 40 | or/33-39 | 889586 |
| **A+E** | 41 | 22 and 40 | 3387 |
| **F. integrated care** | 42 | Delivery of Health Care, Integrated/ | 14279 |
|  | 43 | ((integrated or integration) adj2 (care or health or service? or system* or management)).ti,bt,ab,kf. | 47310 |
|  | 44 | (care adj1 (accountable or coordination or co-ordination or management or Collaborat* or comprehensive or coordinated or co-ordinated or guided or multidisciplinary or multi-disciplinary or seamless or transmural or interdisciplinary or interprofessional or intersectoral or team? or path*)).ti,bt,ab,kf. | 58866 |
|  | 45 | (collaboration adj1 (multidisciplinary or multi-disciplinary or seamless or transmural or interdisciplinary or interprofessional or intersectoral)).ti,bt,ab,kf. | 7687 |
|  | 46 | (Case management or multidisciplinary team care or new care model or service delivery model).ti,bt,ab,kf. | 13797 |
|  | 47 | or/42-46 | 129214 |
| **A+F** | 48 | 22 and 47 | 636 |
| **G. transition of care** | 49 | Continuity of Patient Care/ | 20665 |
|  | 50 | Transition to Adult Care/ | 2106 |
|  | 51 | Transitional Care/ | 1247 |
|  | 52 | (Transition adj6 (adult? or pediatr* or paediatr* or child* or adolesc* or youth or neurop?ediatr*)).ti,bt,ab,kf. | 9864 |
|  | 53 | (((transfer* or referral or retention or transition*) adj3 (care or healthcare or service? or program*)) and (adult? or pediatr* or paediatr* or child* or adolesc* or youth or neurop?ediatr*)).ti,bt,ab,kf. | 11087 |
|  | 54 | or/49-53 | 39106 |
| **A+G** | 55 | 22 and 54 | 355 |
| **String1: (B+C) or (A+D) or (A+E) or (A+F) or (A+G)** | 56 | 30 or 32 or 41 or 48 or 55 | 10026 |
| **H. guidelines** | 57 | guideline/ or practice guideline/ | 37755 |
|  | 58 | (guideline or practice guideline or consensus development conference or consensus development conference, NIH).pt. | 47654 |
|  | 59 | (guideline* or standards or consensus* or recommendat* or (practice parameter* or position statement* or policy statement* or CPG or CPGs or best practice*) or (care adj2 (path or paths or pathway or pathways or map or maps or plan or plans or standard)) or ((critical or clinical or practice) adj2 (path or paths or pathway or pathways or protocol*)) or (algorithm* and (therap* or treatment* or intervention*))).ti,bt. | 225975 |
|  | 60 | (guideline* or standards or consensus* or recommendat*).au. | 10 |
|  | 61 | or/57-60 | 243636 |
| **String1+H** | 62 | 56 and 61 | 467 |
| **String1+H+humans** | 63 | limit 62 to "humans only (removes records about animals)" | 467 |
| **String1+H+humans+date** | 64 | limit 63 to yr="2013 -Current" | 317 |
| **String1+H+humans+date+language** | 65 | (german or english).lg. | 31994634 |
| **Result: guidelines** | 66 | 64 and 65 | 292 |
| **String2: (B+C) or (A+E) or (A+F) or (A+G)** | 67 | 30 or 41 or 48 or 55 | 6502 |
| **I. SRs** | 68 | (((systematic* and review?) or Systematic overview* or ((Cochrane or systemic or scoping or mapping or Umbrella) adj review*) or ((Cochrane or systemic or scoping or mapping or Umbrella) adj literature review*) or "review of reviews" or "overview of reviews" or meta-review or (integrat* adj (review or overview)) or meta-synthes?s or metasynthes?s or "quantitative review" or "quantitative synthesis" or "research synthesis" or meta-ethnography or "Systematic literature search" or "Systematic literature research" or meta-analys?s or metaanalys?s or "meta-analytic review" or "meta-analytical review").ti,kf,bt. or meta-analysis.pt. or Network Meta-Analysis/ or ((search* or medline or pubmed or embase or Cochrane or scopus or "web of science" or "sources of information" or "data sources" or "following databases") and ("study selection" or "selection criteria" or "eligibility criteria" or "inclusion criteria" or "exclusion criteria")).tw. or "systematic review".pt.) not ((letter or editorial or comment or "case reports" or "historical article").pt. or report.ti. or protocol.ti. or protocols.ti. or withdrawn.ti. or "retraction of publication".pt. or exp "retraction of publication as topic"/ or "retracted publication".pt. or reply.ti. or "published erratum".pt.) | 408388 |
| **String2+I** | 69 | 67 and 68 | 211 |
| **String2+I+humans** | 70 | limit 69 to "humans only (removes records about animals)" | 211 |
| **String2+I+humans+language** | 71 | (german or english).lg. | 31994634 |
| **Result: SRs** | 72 | 70 and 71 | 209 |
| **Total** | 73 | 66 or 72 | 491 |

Cochrane Database of Systematic Reviews Issue 7 of 12, July 2023

| ID | Search | Hits |
| --- | --- | --- |
| #1 | [mh ^"Rare Diseases"] | 60 |
| #2 | ((rare or orphan) NEXT (disease? or disorder? or syndrome? or condition?)):ti,kw | 388 |
| #3 | ((rare or orphan) NEAR/3 (multi-organ* or multiorgan* or systemic or multisystem* or multi-system* or genetic or hereditary or inherited or metabolic or endocrin*) NEAR/3 (disease? or disorder? or syndrome? or condition?)):ti,kw | 20 |
| #4 | [mh ^"Cystic Fibrosis"] | 2520 |
| #5 | ("Cystic fibrosis" or Mucoviscidosis or "pancreatic fibrosis"):ti,ab,kw | 6286 |
| #6 | [mh ^"Fabry disease"] | 118 |
| #7 | ("Fabry disease" or "Fabry's disease" or "angiokeratoma corporis diffusum" or "alpha-galactosidase A deficiency" or "GLA deficiency" or "angiokeratoma diffuse"):ti,ab,kw | 248 |
| #8 | [mh ^"Hyperoxaluria, Primary"] | 20 |
| #9 | (Hyperoxaluria or Oxalosis):ti,ab,kw | 152 |
| #10 | [mh ^neurofibromatoses] OR [mh ^"neurofibromatosis 1"] | 96 |
| #11 | (Neurofibromatosis or "von Recklinghausen's disease" or "von Recklinghausen disease" or "von Recklinghausen's syndrome" or "von Recklinghausen syndrome"):ti,ab,kw | 198 |
| #12 | [mh ^"Gaucher Disease"] | 105 |
| #13 | ("Gaucher disease" or "Gaucher's disease" or "cerebroside lipidosis syndrome" or "Gaucher splenomegaly" or "glucocerebrosidase deficiency" or glucocerebrosidosis or "glucosylceramidase deficiency" or "glucosyl cerebroside lipidosis" or "kerasin lipoidosis" or "kerasin thesaurismosis" or ("lipid histiocytosis" NEAR/1 "kerasin type") or "sphingolipidosis 1"):ti,ab,kw | 215 |
| #14 | [mh ^"Glycogen Storage Disease Type II"] | 44 |
| #15 | ("Pompe disease" or "Pompe's disease" or ("Glycogen storage disease" NEAR/1 "type II") or "acid maltase deficiency" or (Glycogenosis NEAR/1 "type II") or "acid alpha-glucosidase deficiency"):ti,ab,kw | 128 |
| #16 | [mh ^Ubiquinone/df] | 6 |
| #17 | ("Coenzyme Q10 deficiency" or "CoQ10 deficiency" or ("Leigh syndrome" NEAR/2 nephro*)):ti,ab,kw | 21 |
| #18 | [mh "Epidermolysis Bullosa"] | 77 |
| #19 | ("Epidermolysis bullosa" or "Kindler's syndrome" or "Kindler syndrome" or "Acrokeratotic poikiloderma" or ("Congenital poikiloderma" NEAR/2 (blisters or keratoses or bullae or "cutaneous atrophy")) or "Hereditary acrokeratotic poikiloderma" or (Hyperkeratosis NEAR/1 hyperpigmentation)):ti,ab,kw | 208 |
| #20 | [mh ^Alkaptonuria] | 12 |
| #21 | (Alkaptonuria or alcaptonuria or "Black urine disease" or "black bone disease"):ti,ab,kw | 17 |
| #22 | (or #1-#21) | 7837 |
| #23 | (care or healthcare or Management or ((treatment or therapy) NEAR/2 (diagnosis or evaluation)) or support):ti | 104142 |
| #24 | [mh Psychotherapy] or [mh ^"Occupational Therapy"] or [mh ^"Patient Education as Topic"] or [mh ^"Health Education"] or [mh "Social Support"] | 50306 |
| #25 | ((occupational:ti,ab,kw OR education*:ti,ab,kw OR behavio*:ti,ab,kw OR cognitive:ti,ab,kw OR psycholog*:ti,ab,kw OR psychosocial:ti,ab,kw OR psychoanlay*:ti,ab,kw OR self-care:ti,ab,kw OR self-efficacy:ti,ab,kw OR knowledge:ti,ab,kw OR information:ti,ab,kw OR Self-management:ti,ab,kw OR Psychodynamic:ti,ab,kw OR coaching:ti,ab,kw OR coping:ti,ab,kw OR "intellectual capacity":ti,ab,kw OR "positive affect":ti,ab,kw OR resilience:ti,ab,kw OR biopsychosocial:ti,ab,kw OR social:ti,ab,kw) NEAR/2 (Intervention?:ti,ab,kw OR Therap*:ti,ab,kw OR Treatment?:ti,ab,kw OR Program*:ti,ab,kw OR care:ti,ab,kw OR support:ti,ab,kw)) | 138785 |
| #26 | (psychotherap*:ti,ab,kw OR "Acceptance and Commitment":ti,ab,kw OR "relaxation training":ti,ab,kw OR ("relaxation" NEXT treatment?):ti,ab,kw OR ("Relaxation" NEXT technique?):ti,ab,kw OR "coping skills training":ti,ab,kw OR "Problem solving":ti,ab,kw OR "autogenic feedback":ti,ab,kw OR Biofeedback:ti,ab,kw OR "patient education":ti,ab,kw OR "health education":ti,ab,kw OR "finding meaning":ti,ab,kw OR "Active techniques":ti,ab,kw OR "Cognitive restructuring":ti,ab,kw OR "Communication development":ti,ab,kw OR "distress reduction":ti,ab,kw OR "Goal setting":ti,ab,kw OR Problem-solving:ti,ab,kw OR ("social" NEXT worker?):ti,ab,kw OR (uncertainty:ti,ab,kw NEAR/1 manag*:ti) OR "patient advocacy":ti,ab,kw OR counsel?ing:ti,ab,kw OR psychoeducation:ti,ab,kw) | 84584 |
| #27 | [mh ^"Delivery of Health Care, Integrated"] | 517 |
| #28 | ((integrated:ti,ab,kw OR integration:ti,ab,kw) NEAR/2 (care:ti,ab,kw OR health:ti,ab,kw OR service?:ti,ab,kw OR system*:ti,ab,kw OR management:ti,ab,kw)) | 4218 |
| #29 | (care:ti,ab,kw NEAR/1 (accountable:ti,ab,kw OR coordination:ti,ab,kw OR co-ordination:ti,ab,kw OR management:ti,ab,kw OR Collaborat*:ti,ab,kw OR comprehensive:ti,ab,kw OR coordinated:ti,ab,kw OR co-ordinated:ti,ab,kw OR guided:ti,ab,kw OR multidisciplinary:ti,ab,kw OR multi-disciplinary:ti,ab,kw OR seamless:ti,ab,kw OR transmural:ti,ab,kw OR interdisciplinary:ti,ab,kw OR interprofessional:ti,ab,kw OR intersectoral:ti,ab,kw OR team?:ti,ab,kw OR path*:ti,ab,kw)) | 9554 |
| #30 | (collaboration:ti,ab,kw NEAR/1 (multidisciplinary:ti,ab,kw OR multi-disciplinary:ti,ab,kw OR seamless:ti,ab,kw OR transmural:ti,ab,kw OR interdisciplinary:ti,ab,kw OR interprofessional:ti,ab,kw OR intersectoral:ti,ab,kw)) | 329 |
| #31 | ("Case management":ti,ab,kw OR "multidisciplinary team care":ti,ab,kw OR "new care model":ti,ab,kw OR "service delivery model":ti,ab,kw) | 2929 |
| #32 | [mh ^"Continuity of Patient Care"] or [mh ^"Transition to Adult Care"] or [mh ^"Transitional Care"] | 913 |
| #33 | (Transition:ti,ab,kw NEAR/6 (adult?:ti,ab,kw OR pediatr*:ti,ab,kw OR paediatr*:ti,ab,kw OR child*:ti,ab,kw OR adolesc*:ti,ab,kw OR youth:ti,ab,kw OR neurop?ediatr*:ti)) | 662 |
| #34 | (((transfer*:ti,ab,kw OR referral:ti,ab,kw OR retention:ti,ab,kw OR transition*:ti,ab,kw) NEAR/3 (care:ti,ab,kw OR healthcare:ti,ab,kw OR service?:ti,ab,kw OR program*:ti)) AND (adult?:ti,ab,kw OR pediatr*:ti,ab,kw OR paediatr*:ti,ab,kw OR child*:ti,ab,kw OR adolesc*:ti,ab,kw OR youth:ti,ab,kw OR neurop?ediatr*:ti)) | 3388 |
| #35 | (or #23-#34) | 271124 |
| #36 | #22 and #35 | 605 |
| #37 | [mh ^Hypogonadism] | 605 |
| #38 | (hypogonadism or hypogonadal):ti,ab,kw | 1372 |
| #39 | ((endocrine NEAR/3 (deficit? or deficien*)) and (testosterone NEAR/3 (low or deficien* or insufficien*))):ti,ab,kw | 1 |
| #40 | (or #37-#39) | 1372 |
| #41 | [mh ^"Hormone Replacement Therapy"] AND testosterone:ti,ab,kw | 225 |
| #42 | (Testosterone NEAR/1 (replacement or treatment or therapy)):ti,ab,kw | 1123 |
| #43 | #41 or #42 | 1223 |
| #44 | #40 and #43 | 519 |
| #45 | #36 or #44 in Cochrane Reviews | 25 |

Epistemonikos.org 3 July 2023

| Search 1 | Search | Results |
| --- | --- | --- |
|  | title:((rare OR orphan) AND (multi-organ OR multiorgan OR systemic OR multisystem OR multi-system OR genetic OR hereditary OR metabolic OR endocrine) AND (disease* OR disorder* OR syndrome* OR condition*)) OR (title:("Cystic fibrosis" OR "Mucoviscidosis" OR "pancreatic fibrosis" OR "Fabry disease" OR "Fabry's disease" OR "angiokeratoma corporis diffusum" OR "alpha-galactosidase A deficiency" OR "GLA deficiency" OR "angiokeratoma diffuse" OR "Hyperoxaluria" OR "Oxalosis" OR "Neurofibromatosis" OR "Von Recklinghausen's disease" OR "Von Recklinghausen disease" OR "von Recklinghausen's syndrome" OR "von Recklinghausen syndrome" OR "Gaucher disease" OR "Gaucher's disease" OR "cerebroside lipidosis syndrome" OR "Gaucher splenomegaly" OR "glucocerebrosidase deficiency" OR "glucocerebrosidosis" OR "glucosylceramidase deficiency" OR "glucosyl cerebroside lipidosis" OR "kerasin lipoidosis" OR "kerasin thesaurismosis" OR "lipid histiocytosis (kerasin type)" OR "sphingolipidosis 1" OR "Pompe disease" OR "Pompe's disease" OR "Glycogen storage disease type II" OR "acid maltase deficiency" OR "Glycogenosis type II" OR "acid alpha-glucosidase deficiency" OR "Coenzyme Q10 deficiency" OR "CoQ10 deficiency" OR "Leigh syndrome with nephrotic syndrome" OR "Leigh syndrome with nephropathy" OR "Epidermolysis bullosa" OR "Kindler's syndrome" OR "Kindler syndrome" OR "Acrokeratotic poikiloderma" OR "Congenital poikiloderma with blisters AND keratoses" OR "Congenital poikiloderma with bullae AND progressive cutaneous atrophy" OR "Hereditary acrokeratotic poikiloderma" OR "Hyperkeratosis–hyperpigmentation syndrome" OR "Alkaptonuria" OR "alcaptonuria" OR "Black urine disease" OR "black bone disease") OR abstract:("Cystic fibrosis" OR "Mucoviscidosis" OR "pancreatic fibrosis" OR "Fabry disease" OR "Fabry's disease" OR "angiokeratoma corporis diffusum" OR "alpha-galactosidase A deficiency" OR "GLA deficiency" OR "angiokeratoma diffuse" OR "Hyperoxaluria" OR "Oxalosis" OR "Neurofibromatosis" OR "Von Recklinghausen's disease" OR "Von Recklinghausen disease" OR "von Recklinghausen's syndrome" OR "von Recklinghausen syndrome" OR "Gaucher disease" OR "Gaucher's disease" OR "cerebroside lipidosis syndrome" OR "Gaucher splenomegaly" OR "glucocerebrosidase deficiency" OR "glucocerebrosidosis" OR "glucosylceramidase deficiency" OR "glucosyl cerebroside lipidosis" OR "kerasin lipoidosis" OR "kerasin thesaurismosis" OR "lipid histiocytosis (kerasin type)" OR "sphingolipidosis 1" OR "Pompe disease" OR "Pompe's disease" OR "Glycogen storage disease type II" OR "acid maltase deficiency" OR "Glycogenosis type II" OR "acid alpha-glucosidase deficiency" OR "Coenzyme Q10 deficiency" OR "CoQ10 deficiency" OR "Leigh syndrome with nephrotic syndrome" OR "Leigh syndrome with nephropathy" OR "Epidermolysis bullosa" OR "Kindler's syndrome" OR "Kindler syndrome" OR "Acrokeratotic poikiloderma" OR "Congenital poikiloderma with blisters AND keratoses" OR "Congenital poikiloderma with bullae AND progressive cutaneous atrophy" OR "Hereditary acrokeratotic poikiloderma" OR "Hyperkeratosis–hyperpigmentation syndrome" OR "Alkaptonuria" OR "alcaptonuria" OR "Black urine disease" OR "black bone disease")) | 9958 |
|  | Filter: Broad Synthesis (includes guidelines) | 42 |
|  | Filter: 2013-2023 | 28 |
| URL | https://www.epistemonikos.org/advanced_search?q=title:((rare%20OR%20orphan)%20AND%20(multi-organ%20OR%20multiorgan%20OR%20systemic%20%20OR%20multisystem%20%20OR%20multi-system%20%20OR%20genetic%20OR%20hereditary%20OR%20metabolic%20OR%20endocrine)%20AND%20(disease*%20OR%20disorder*%20OR%20syndrome*%20OR%20condition*))%20OR%20(title:(%22Cystic%20fibrosis%22%20OR%20%22Mucoviscidosis%22%20OR%20%22pancreatic%20fibrosis%22%20OR%20%22Fabry%20disease%22%20OR%20%22Fabry%27s%20disease%22%20OR%20%22angiokeratoma%20corporis%20diffusum%22%20OR%20%22alpha-galactosidase%20A%20deficiency%22%20OR%20%22GLA%20deficiency%22%20OR%20%22angiokeratoma%20diffuse%22%20OR%20%22Hyperoxaluria%22%20OR%20%22Oxalosis%22%20OR%20%22Neurofibromatosis%22%20OR%20%22Von%20Recklinghausen%27s%20disease%22%20OR%20%22Von%20Recklinghausen%20disease%22%20OR%20%22von%20Recklinghausen%27s%20syndrome%22%20OR%20%22von%20Recklinghausen%20syndrome%22%20OR%20%22Gaucher%20disease%22%20OR%20%22Gaucher%27s%20disease%22%20OR%20%22cerebroside%20lipidosis%20syndrome%22%20OR%20%22Gaucher%20splenomegaly%22%20OR%20%22glucocerebrosidase%20deficiency%22%20OR%20%22glucocerebrosidosis%22%20OR%20%22glucosylceramidase%20deficiency%22%20OR%20%22glucosyl%20cerebroside%20lipidosis%22%20OR%20%22kerasin%20lipoidosis%22%20OR%20%22kerasin%20thesaurismosis%22%20OR%20%22lipid%20histiocytosis%20(kerasin%20type)%22%20OR%20%22sphingolipidosis%201%22%20OR%20%22Pompe%20disease%22%20OR%20%22Pompe%27s%20disease%22%20OR%20%22Glycogen%20storage%20disease%20type%20II%22%20OR%20%22acid%20maltase%20deficiency%22%20OR%20%22Glycogenosis%20type%20II%22%20OR%20%22acid%20alpha-glucosidase%20deficiency%22%20OR%20%22Coenzyme%20Q10%20deficiency%22%20OR%20%22CoQ10%20deficiency%22%20OR%20%22Leigh%20syndrome%20with%20nephrotic%20syndrome%22%20OR%20%22Leigh%20syndrome%20with%20nephropathy%22%20OR%20%22Epidermolysis%20bullosa%22%20OR%20%22Kindler%27s%20syndrome%22%20OR%20%22Kindler%20syndrome%22%20OR%20%22Acrokeratotic%20poikiloderma%22%20OR%20%22Congenital%20poikiloderma%20with%20blisters%20AND%20keratoses%22%20OR%20%22Congenital%20poikiloderma%20with%20bullae%20AND%20progressive%20cutaneous%20atrophy%22%20OR%20%22Hereditary%20acrokeratotic%20poikiloderma%22%20OR%20%22Hyperkeratosis%E2%80%93hyperpigmentation%20syndrome%22%20OR%20%22Alkaptonuria%22%20OR%20%22alcaptonuria%22%20OR%20%22Black%20urine%20disease%22%20OR%20%22black%20bone%20disease%22)%20OR%20abstract:(%22Cystic%20fibrosis%22%20OR%20%22Mucoviscidosis%22%20OR%20%22pancreatic%20fibrosis%22%20OR%20%22Fabry%20disease%22%20OR%20%22Fabry%27s%20disease%22%20OR%20%22angiokeratoma%20corporis%20diffusum%22%20OR%20%22alpha-galactosidase%20A%20deficiency%22%20OR%20%22GLA%20deficiency%22%20OR%20%22angiokeratoma%20diffuse%22%20OR%20%22Hyperoxaluria%22%20OR%20%22Oxalosis%22%20OR%20%22Neurofibromatosis%22%20OR%20%22Von%20Recklinghausen%27s%20disease%22%20OR%20%22Von%20Recklinghausen%20disease%22%20OR%20%22von%20Recklinghausen%27s%20syndrome%22%20OR%20%22von%20Recklinghausen%20syndrome%22%20OR%20%22Gaucher%20disease%22%20OR%20%22Gaucher%27s%20disease%22%20OR%20%22cerebroside%20lipidosis%20syndrome%22%20OR%20%22Gaucher%20splenomegaly%22%20OR%20%22glucocerebrosidase%20deficiency%22%20OR%20%22glucocerebrosidosis%22%20OR%20%22glucosylceramidase%20deficiency%22%20OR%20%22glucosyl%20cerebroside%20lipidosis%22%20OR%20%22kerasin%20lipoidosis%22%20OR%20%22kerasin%20thesaurismosis%22%20OR%20%22lipid%20histiocytosis%20(kerasin%20type)%22%20OR%20%22sphingolipidosis%201%22%20OR%20%22Pompe%20disease%22%20OR%20%22Pompe%27s%20disease%22%20OR%20%22Glycogen%20storage%20disease%20type%20II%22%20OR%20%22acid%20maltase%20deficiency%22%20OR%20%22Glycogenosis%20type%20II%22%20OR%20%22acid%20alpha-glucosidase%20deficiency%22%20OR%20%22Coenzyme%20Q10%20deficiency%22%20OR%20%22CoQ10%20deficiency%22%20OR%20%22Leigh%20syndrome%20with%20nephrotic%20syndrome%22%20OR%20%22Leigh%20syndrome%20with%20nephropathy%22%20OR%20%22Epidermolysis%20bullosa%22%20OR%20%22Kindler%27s%20syndrome%22%20OR%20%22Kindler%20syndrome%22%20OR%20%22Acrokeratotic%20poikiloderma%22%20OR%20%22Congenital%20poikiloderma%20with%20blisters%20AND%20keratoses%22%20OR%20%22Congenital%20poikiloderma%20with%20bullae%20AND%20progressive%20cutaneous%20atrophy%22%20OR%20%22Hereditary%20acrokeratotic%20poikiloderma%22%20OR%20%22Hyperkeratosis%E2%80%93hyperpigmentation%20syndrome%22%20OR%20%22Alkaptonuria%22%20OR%20%22alcaptonuria%22%20OR%20%22Black%20urine%20disease%22%20OR%20%22black%20bone%20disease%22))&protocol=no&classification=broad-synthesis&min_year=2013&max_year=2023 |  |
| Search 2 | Search | Results |
|  | (title:(management OR occupational OR education OR educational OR behavior OR behaviour OR behavioral OR behavioural OR cognitive OR psychological OR psychosocial OR psychoanlaytic OR self-efficacy OR knowledge OR information OR Psychodynamic OR psychotherap* OR Acceptance OR Commitment OR relaxation OR relaxation OR coping OR coaching OR capacity OR "positive affect" OR resilience OR biopsychosocial OR "Problem solving" OR autogenic OR Biofeedback OR "patient education" OR "health education" OR Accountable OR Care OR Integrated OR Integration OR interprofessional OR multidisciplinary OR multi-disciplinary OR transmural OR interdisciplinary OR Transfer* OR Transition* OR ((child* OR pediatr* OR paediatr* OR adolesc* OR teen*) AND adult*))) AND ("Cystic fibrosis" OR "Mucoviscidosis" OR "pancreatic fibrosis" OR "Fabry disease" OR "Fabry's disease" OR "angiokeratoma corporis diffusum" OR "alpha-galactosidase A deficiency" OR "GLA deficiency" OR "angiokeratoma diffuse" OR "Hyperoxaluria" OR "Oxalosis" OR "Neurofibromatosis" OR "Von Recklinghausen's disease" OR "Von Recklinghausen disease" OR "von Recklinghausen's syndrome" OR "von Recklinghausen syndrome" OR "Gaucher disease" OR "Gaucher's disease" OR "cerebroside lipidosis syndrome" OR "Gaucher splenomegaly" OR "glucocerebrosidase deficiency" OR "glucocerebrosidosis" OR "glucosylceramidase deficiency" OR "glucosyl cerebroside lipidosis" OR "kerasin lipoidosis" OR "kerasin thesaurismosis" OR "lipid histiocytosis (kerasin type)" OR "sphingolipidosis 1" OR "Pompe disease" OR "Pompe's disease" OR "Glycogen storage disease type II" OR "acid maltase deficiency" OR "Glycogenosis type II" OR "acid alpha-glucosidase deficiency" OR "Coenzyme Q10 deficiency" OR "CoQ10 deficiency" OR "Leigh syndrome with nephrotic syndrome" OR "Leigh syndrome with nephropathy" OR "Epidermolysis bullosa" OR "Kindler's syndrome" OR "Kindler syndrome" OR "Acrokeratotic poikiloderma" OR "Congenital poikiloderma with blisters and keratoses" OR "Congenital poikiloderma with bullae and progressive cutaneous atrophy" OR "Hereditary acrokeratotic poikiloderma" OR "Hyperkeratosis–hyperpigmentation syndrome" OR "Alkaptonuria" OR "alcaptonuria" OR "Black urine disease" OR "black bone disease" OR (title:((rare OR orphan) AND (multi-organ OR multiorgan OR systemic OR multisystem OR multi-system OR genetic OR hereditary OR metabolic OR endocrine) AND (disease* OR disorder* OR syndrome* OR condition*)))) | 698 |
|  | Filter: Systematic Review | 132 |
| URL | https://www.epistemonikos.org/advanced_search?q=(title:(management%20%20OR%20occupational%20OR%20education%20OR%20educational%20OR%20behavior%20OR%20behaviour%20OR%20behavioral%20OR%20behavioural%20OR%20cognitive%20OR%20psychological%20OR%20psychosocial%20OR%20psychoanlaytic%20OR%20self-efficacy%20OR%20knowledge%20OR%20information%20OR%20Psychodynamic%20OR%20psychotherap*%20OR%20Acceptance%20%20OR%20Commitment%20OR%20relaxation%20OR%20relaxation%20OR%20coping%20OR%20coaching%20OR%20capacity%20OR%20%22positive%20affect%22%20OR%20resilience%20OR%20biopsychosocial%20OR%20%22Problem%20solving%22%20%20OR%20autogenic%20OR%20Biofeedback%20OR%20%22patient%20education%22%20OR%20%22health%20education%22%20OR%20Accountable%20OR%20Care%20OR%20Integrated%20OR%20Integration%20OR%20interprofessional%20OR%20multidisciplinary%20OR%20multi-disciplinary%20OR%20transmural%20OR%20interdisciplinary%20OR%20Transfer*%20OR%20Transition*%20OR%20((child*%20OR%20pediatr*%20OR%20paediatr*%20OR%20adolesc*%20OR%20teen*)%20AND%20adult*)))%20AND%20(%22Cystic%20fibrosis%22%20OR%20%22Mucoviscidosis%22%20OR%20%22pancreatic%20fibrosis%22%20OR%20%22Fabry%20disease%22%20OR%20%22Fabry%27s%20disease%22%20OR%20%22angiokeratoma%20corporis%20diffusum%22%20OR%20%22alpha-galactosidase%20A%20deficiency%22%20OR%20%22GLA%20deficiency%22%20OR%20%22angiokeratoma%20diffuse%22%20OR%20%22Hyperoxaluria%22%20OR%20%22Oxalosis%22%20OR%20%22Neurofibromatosis%22%20OR%20%22Von%20Recklinghausen%27s%20disease%22%20OR%20%22Von%20Recklinghausen%20disease%22%20OR%20%22von%20Recklinghausen%27s%20syndrome%22%20OR%20%22von%20Recklinghausen%20syndrome%22%20OR%20%22Gaucher%20disease%22%20OR%20%22Gaucher%27s%20disease%22%20OR%20%22cerebroside%20lipidosis%20syndrome%22%20OR%20%22Gaucher%20splenomegaly%22%20OR%20%22glucocerebrosidase%20deficiency%22%20OR%20%22glucocerebrosidosis%22%20OR%20%22glucosylceramidase%20deficiency%22%20OR%20%22glucosyl%20cerebroside%20lipidosis%22%20OR%20%22kerasin%20lipoidosis%22%20OR%20%22kerasin%20thesaurismosis%22%20OR%20%22lipid%20histiocytosis%20(kerasin%20type)%22%20OR%20%22sphingolipidosis%201%22%20OR%20%22Pompe%20disease%22%20OR%20%22Pompe%27s%20disease%22%20OR%20%22Glycogen%20storage%20disease%20type%20II%22%20OR%20%22acid%20maltase%20deficiency%22%20OR%20%22Glycogenosis%20type%20II%22%20OR%20%22acid%20alpha-glucosidase%20deficiency%22%20OR%20%22Coenzyme%20Q10%20deficiency%22%20OR%20%22CoQ10%20deficiency%22%20OR%20%22Leigh%20syndrome%20with%20nephrotic%20syndrome%22%20OR%20%22Leigh%20syndrome%20with%20nephropathy%22%20OR%20%22Epidermolysis%20bullosa%22%20OR%20%22Kindler%27s%20syndrome%22%20OR%20%22Kindler%20syndrome%22%20OR%20%22Acrokeratotic%20poikiloderma%22%20OR%20%22Congenital%20poikiloderma%20with%20blisters%20and%20keratoses%22%20OR%20%22Congenital%20poikiloderma%20with%20bullae%20and%20progressive%20cutaneous%20atrophy%22%20OR%20%22Hereditary%20acrokeratotic%20poikiloderma%22%20OR%20%22Hyperkeratosis%E2%80%93hyperpigmentation%20syndrome%22%20OR%20%22Alkaptonuria%22%20OR%20%22alcaptonuria%22%20OR%20%22Black%20urine%20disease%22%20OR%20%22black%20bone%20disease%22%20OR%20(title:((rare%20OR%20orphan)%20AND%20(multi-organ%20OR%20multiorgan%20OR%20systemic%20%20OR%20multisystem%20%20OR%20multi-system%20%20OR%20genetic%20OR%20hereditary%20OR%20metabolic%20OR%20endocrine)%20AND%20(disease*%20OR%20disorder*%20OR%20syndrome*%20OR%20condition*))))&protocol=no&classification=systematic-review |  |
|  |  |  |
| Search 3 | Search | Results |
|  | (title:(management OR occupational OR education OR educational OR behavior OR behaviour OR behavioral OR behavioural OR cognitive OR psychological OR psychosocial OR psychoanlaytic OR self-efficacy OR knowledge OR information OR Psychodynamic OR psychotherap* OR Acceptance OR Commitment OR relaxation OR relaxation OR coping OR coaching OR capacity OR "positive affect" OR resilience OR biopsychosocial OR "Problem solving" OR autogenic OR Biofeedback OR "patient education" OR "health education" OR Accountable OR Care OR Integrated OR Integration OR interprofessional OR multidisciplinary OR multi-disciplinary OR transmural OR interdisciplinary OR Transfer* OR Transition*) OR abstract:(management OR occupational OR education OR educational OR behavior OR behaviour OR behavioral OR behavioural OR cognitive OR psychological OR psychosocial OR psychoanlaytic OR self-efficacy OR knowledge OR information OR Psychodynamic OR psychotherap* OR Acceptance OR Commitment OR relaxation OR relaxation OR coping OR coaching OR capacity OR "positive affect" OR resilience OR biopsychosocial OR "Problem solving" OR autogenic OR Biofeedback OR "patient education" OR "health education" OR Accountable OR Care OR Integrated OR Integration OR interprofessional OR multidisciplinary OR multi-disciplinary OR transmural OR interdisciplinary OR Transfer* OR Transition*)) AND title:(((rare OR orphan) AND (multi-organ OR multiorgan OR systemic OR multisystem OR multi-system OR genetic OR hereditary OR metabolic OR endocrine) AND (disease* OR disorder* OR syndrome* OR condition*)) OR "Cystic fibrosis" OR "Mucoviscidosis" OR "pancreatic fibrosis" OR "Fabry disease" OR "Fabry's disease" OR "angiokeratoma corporis diffusum" OR "alpha-galactosidase A deficiency" OR "GLA deficiency" OR "angiokeratoma diffuse" OR "Hyperoxaluria" OR "Oxalosis" OR "Neurofibromatosis" OR "Von Recklinghausen's disease" OR "Von Recklinghausen disease" OR "von Recklinghausen's syndrome" OR "von Recklinghausen syndrome" OR "Gaucher disease" OR "Gaucher's disease" OR "cerebroside lipidosis syndrome" OR "Gaucher splenomegaly" OR "glucocerebrosidase deficiency" OR "glucocerebrosidosis" OR "glucosylceramidase deficiency" OR "glucosyl cerebroside lipidosis" OR "kerasin lipoidosis" OR "kerasin thesaurismosis" OR "lipid histiocytosis (kerasin type)" OR "sphingolipidosis 1" OR "Pompe disease" OR "Pompe's disease" OR "Glycogen storage disease type II" OR "acid maltase deficiency" OR "Glycogenosis type II" OR "acid alpha-glucosidase deficiency" OR "Coenzyme Q10 deficiency" OR "CoQ10 deficiency" OR "Leigh syndrome with nephrotic syndrome" OR "Leigh syndrome with nephropathy" OR "Epidermolysis bullosa" OR "Kindler's syndrome" OR "Kindler syndrome" OR "Acrokeratotic poikiloderma" OR "Congenital poikiloderma with blisters AND keratoses" OR "Congenital poikiloderma with bullae AND progressive cutaneous atrophy" OR "Hereditary acrokeratotic poikiloderma" OR "Hyperkeratosis–hyperpigmentation syndrome" OR "Alkaptonuria" OR "alcaptonuria" OR "Black urine disease" OR "black bone disease") | 1967 |
|  | Filter: Systematic Review | 347 |
| URL | https://www.epistemonikos.org/advanced_search?q=(title:(management%20%20OR%20occupational%20OR%20education%20OR%20educational%20OR%20behavior%20OR%20behaviour%20OR%20behavioral%20OR%20behavioural%20OR%20cognitive%20OR%20psychological%20OR%20psychosocial%20OR%20psychoanlaytic%20OR%20self-efficacy%20OR%20knowledge%20OR%20information%20OR%20Psychodynamic%20OR%20psychotherap*%20OR%20Acceptance%20%20OR%20Commitment%20OR%20relaxation%20OR%20relaxation%20OR%20coping%20OR%20coaching%20OR%20capacity%20OR%20%22positive%20affect%22%20OR%20resilience%20OR%20biopsychosocial%20OR%20%22Problem%20solving%22%20%20OR%20autogenic%20OR%20Biofeedback%20OR%20%22patient%20education%22%20OR%20%22health%20education%22%20OR%20Accountable%20OR%20Care%20OR%20Integrated%20OR%20Integration%20OR%20interprofessional%20OR%20multidisciplinary%20OR%20multi-disciplinary%20OR%20transmural%20OR%20interdisciplinary%20OR%20Transfer*%20OR%20Transition*)%20OR%20abstract:(management%20%20OR%20occupational%20OR%20education%20OR%20educational%20OR%20behavior%20OR%20behaviour%20OR%20behavioral%20OR%20behavioural%20OR%20cognitive%20OR%20psychological%20OR%20psychosocial%20OR%20psychoanlaytic%20OR%20self-efficacy%20OR%20knowledge%20OR%20information%20OR%20Psychodynamic%20OR%20psychotherap*%20OR%20Acceptance%20%20OR%20Commitment%20OR%20relaxation%20OR%20relaxation%20OR%20coping%20OR%20coaching%20OR%20capacity%20OR%20%22positive%20affect%22%20OR%20resilience%20OR%20biopsychosocial%20OR%20%22Problem%20solving%22%20%20OR%20autogenic%20OR%20Biofeedback%20OR%20%22patient%20education%22%20OR%20%22health%20education%22%20OR%20Accountable%20OR%20Care%20OR%20Integrated%20OR%20Integration%20OR%20interprofessional%20OR%20multidisciplinary%20OR%20multi-disciplinary%20OR%20transmural%20OR%20interdisciplinary%20OR%20Transfer*%20OR%20Transition*))%20AND%20title:(((rare%20OR%20orphan)%20AND%20(multi-organ%20OR%20multiorgan%20OR%20systemic%20%20OR%20multisystem%20%20OR%20multi-system%20%20OR%20genetic%20OR%20hereditary%20OR%20metabolic%20OR%20endocrine)%20AND%20(disease*%20OR%20disorder*%20OR%20syndrome*%20OR%20condition*))%20OR%20%22Cystic%20fibrosis%22%20OR%20%22Mucoviscidosis%22%20OR%20%22pancreatic%20fibrosis%22%20OR%20%22Fabry%20disease%22%20OR%20%22Fabry%27s%20disease%22%20OR%20%22angiokeratoma%20corporis%20diffusum%22%20OR%20%22alpha-galactosidase%20A%20deficiency%22%20OR%20%22GLA%20deficiency%22%20OR%20%22angiokeratoma%20diffuse%22%20OR%20%22Hyperoxaluria%22%20OR%20%22Oxalosis%22%20OR%20%22Neurofibromatosis%22%20OR%20%22Von%20Recklinghausen%27s%20disease%22%20OR%20%22Von%20Recklinghausen%20disease%22%20OR%20%22von%20Recklinghausen%27s%20syndrome%22%20OR%20%22von%20Recklinghausen%20syndrome%22%20OR%20%22Gaucher%20disease%22%20OR%20%22Gaucher%27s%20disease%22%20OR%20%22cerebroside%20lipidosis%20syndrome%22%20OR%20%22Gaucher%20splenomegaly%22%20OR%20%22glucocerebrosidase%20deficiency%22%20OR%20%22glucocerebrosidosis%22%20OR%20%22glucosylceramidase%20deficiency%22%20OR%20%22glucosyl%20cerebroside%20lipidosis%22%20OR%20%22kerasin%20lipoidosis%22%20OR%20%22kerasin%20thesaurismosis%22%20OR%20%22lipid%20histiocytosis%20(kerasin%20type)%22%20OR%20%22sphingolipidosis%201%22%20OR%20%22Pompe%20disease%22%20OR%20%22Pompe%27s%20disease%22%20OR%20%22Glycogen%20storage%20disease%20type%20II%22%20OR%20%22acid%20maltase%20deficiency%22%20OR%20%22Glycogenosis%20type%20II%22%20OR%20%22acid%20alpha-glucosidase%20deficiency%22%20OR%20%22Coenzyme%20Q10%20deficiency%22%20OR%20%22CoQ10%20deficiency%22%20OR%20%22Leigh%20syndrome%20with%20nephrotic%20syndrome%22%20OR%20%22Leigh%20syndrome%20with%20nephropathy%22%20OR%20%22Epidermolysis%20bullosa%22%20OR%20%22Kindler%27s%20syndrome%22%20OR%20%22Kindler%20syndrome%22%20OR%20%22Acrokeratotic%20poikiloderma%22%20OR%20%22Congenital%20poikiloderma%20with%20blisters%20AND%20keratoses%22%20OR%20%22Congenital%20poikiloderma%20with%20bullae%20AND%20progressive%20cutaneous%20atrophy%22%20OR%20%22Hereditary%20acrokeratotic%20poikiloderma%22%20OR%20%22Hyperkeratosis%E2%80%93hyperpigmentation%20syndrome%22%20OR%20%22Alkaptonuria%22%20OR%20%22alcaptonuria%22%20OR%20%22Black%20urine%20disease%22%20OR%20%22black%20bone%20disease%22)&protocol=no&classification=systematic-review |  |
| Search 4 | Search | Results |
|  | (title:(hypogonadism OR hypogonadal) OR abstract:(hypogonadism OR hypogonadal)) AND (title:("Testosterone replacement" OR "Testosterone therapy" OR "Testosterone treatment" OR TRT) OR abstract:("Testosterone replacement" OR "Testosterone therapy" OR "Testosterone treatment" OR TRT)) | 322 |
|  | Filter: Broad Synthesis (includes guidelines) | 2 |
|  | Filter: 2013-2023 | 2 |
| URL | https://www.epistemonikos.org/advanced_search?q=(title:(hypogonadism%20OR%20hypogonadal)%20OR%20abstract:(hypogonadism%20OR%20hypogonadal))%20AND%20(title:(%22Testosterone%20replacement%22%20OR%20%22Testosterone%20therapy%22%20OR%20%22Testosterone%20treatment%22%20OR%20TRT)%20OR%20abstract:(%22Testosterone%20replacement%22%20OR%20%22Testosterone%20therapy%22%20OR%20%22Testosterone%20treatment%22%20OR%20TRT))&protocol=no&classification=broad-synthesis&min_year=2013&max_year=2023 |  |
|  | Filter: Systematic Review | 96 |
|  | https://www.epistemonikos.org/advanced_search?q=(title:(hypogonadism%20OR%20hypogonadal)%20OR%20abstract:(hypogonadism%20OR%20hypogonadal))%20AND%20(title:(%22Testosterone%20replacement%22%20OR%20%22Testosterone%20therapy%22%20OR%20%22Testosterone%20treatment%22%20OR%20TRT)%20OR%20abstract:(%22Testosterone%20replacement%22%20OR%20%22Testosterone%20therapy%22%20OR%20%22Testosterone%20treatment%22%20OR%20TRT))&protocol=no&classification=systematic-review |  |
|  |  |  |
| Total (including duplicates) | | 605 |

Trip ([www.tripdatabase.com](http://www.tripdatabase.com)) 3 July 2023

| Search 1 |  |  |  |
| --- | --- | --- | --- |
| ALL of these words | Title | rare disease* | Proximity: 6 |
| ANY of these words | Title | multi-organ multiorgan systemic multisystem multi-system genetic hereditary metabolic endocrine |  |
| Filter: | Guidelines |  |  |
| Filter: | 2013-2023 |  |  |
| Result |  |  | 1 |
|  |  |  |  |
| Search 2 |  |  |  |
| ALL of these words | Title | rare disorder* | Proximity: 6 |
| ANY of these words | Title | multi-organ multiorgan systemic multisystem multi-system genetic hereditary metabolic endocrine |  |
| Filter: | Guidelines |  |  |
| Filter: | 2013-2023 |  |  |
| Result |  |  | 0 |
|  |  |  |  |
| Search 3 |  |  |  |
| ALL of these words | Title | rare condition* | Proximity: 6 |
| ANY of these words | Title | multi-organ multiorgan systemic multisystem multi-system genetic hereditary metabolic endocrine |  |
| Filter: | Guidelines |  |  |
| Filter: | 2013-2023 |  |  |
| Result |  |  | 0 |
|  |  |  |  |
| Search 4 |  |  |  |
| ALL of these words | Title | orphan disease* | Proximity: 6 |
| ANY of these words | Title | multi-organ multiorgan systemic multisystem multi-system genetic hereditary metabolic endocrine |  |
| Filter: | Guidelines |  |  |
| Filter: | 2013-2023 |  |  |
| Result |  |  | 0 |
|  |  |  |  |
| Search 5 |  |  |  |
| ALL of these words | Title | orphan disorder* | Proximity: 6 |
| ANY of these words | Title | multi-organ multiorgan systemic multisystem multi-system genetic hereditary metabolic endocrine |  |
| Filter: | Guidelines |  |  |
| Filter: | 2013-2023 |  |  |
| Result |  |  | 0 |
|  |  |  |  |
| Search 6 |  |  |  |
| ALL of these words | Title | orphan condition* | Proximity: 6 |
| ANY of these words | Title | multi-organ multiorgan systemic multisystem multi-system genetic hereditary metabolic endocrine |  |
| Filter: | Guidelines |  |  |
| Filter: | 2013-2023 |  |  |
| Result |  |  | 0 |
|  |  |  |  |
| Search 7 |  |  |  |
| ANY of these words | Title | "cystic fibrosis" "mucoviscidosis" "pancreatic fibrosis" "fabry disease" "fabry's disease" "angiokeratoma corporis diffusum" "alpha-galactosidase a deficiency" "gla deficiency" "angiokeratoma diffuse" "hyperoxaluria" "oxalosis" "neurofibromatosis" "von recklinghausen's disease" "von recklinghausen disease" "von recklinghausen's syndrome" "von recklinghausen syndrome" "gaucher disease" "gaucher's disease" "cerebroside lipidosis syndrome" "gaucher splenomegaly" "glucocerebrosidase deficiency" "glucocerebrosidosis" "glucosylceramidase deficiency" "glucosyl cerebroside lipidosis" "kerasin lipoidosis" "kerasin thesaurismosis" "lipid histiocytosis (kerasin type)" "sphingolipidosis 1" "pompe disease" "pompe's disease" "glycogen storage disease type ii" "acid maltase deficiency" "glycogenosis type ii" "acid alpha-glucosidase deficiency" "coenzyme q10 deficiency" "coq10 deficiency" "leigh syndrome with nephrotic syndrome" "leigh syndrome with nephropathy" "epidermolysis bullosa" "kindler's syndrome" "kindler syndrome" "acrokeratotic poikiloderma" "congenital poikiloderma with blisters AND keratoses" "congenital poikiloderma with bullae AND progressive cutaneous atrophy" "hereditary acrokeratotic poikiloderma" "hyperkeratosis–hyperpigmentation syndrome" "alkaptonuria" "alcaptonuria" "black urine disease" "black bone disease" |  |
| Filter: | Guidelines |  |  |
| Filter: | 2013-2023 |  |  |
| Result |  |  | 3 |
|  |  |  |  |
| Search 8 |  |  |  |
| ALL of these words | Title | management |  |
| ANY of these words | Document | "cystic fibrosis" "mucoviscidosis" "pancreatic fibrosis" "fabry disease" "fabry's disease" "angiokeratoma corporis diffusum" "alpha-galactosidase a deficiency" "gla deficiency" "angiokeratoma diffuse" "hyperoxaluria" "oxalosis" "neurofibromatosis" "von recklinghausen's disease" "von recklinghausen disease" "von recklinghausen's syndrome" "von recklinghausen syndrome" "gaucher disease" "gaucher's disease" "cerebroside lipidosis syndrome" "gaucher splenomegaly" "glucocerebrosidase deficiency" "glucocerebrosidosis" "glucosylceramidase deficiency" "glucosyl cerebroside lipidosis" "kerasin lipoidosis" "kerasin thesaurismosis" "lipid histiocytosis (kerasin type)" "sphingolipidosis 1" "pompe disease" "pompe's disease" "glycogen storage disease type ii" "acid maltase deficiency" "glycogenosis type ii" "acid alpha-glucosidase deficiency" "coenzyme q10 deficiency" "coq10 deficiency" "leigh syndrome with nephrotic syndrome" "leigh syndrome with nephropathy" "epidermolysis bullosa" "kindler's syndrome" "kindler syndrome" "acrokeratotic poikiloderma" "congenital poikiloderma with blisters AND keratoses" "congenital poikiloderma with bullae AND progressive cutaneous atrophy" "hereditary acrokeratotic poikiloderma" "hyperkeratosis–hyperpigmentation syndrome" "alkaptonuria" "alcaptonuria" "black urine disease" "black bone disease" |  |
| Filter: | Guidelines |  |  |
| Filter: | 2013-2023 |  |  |
| Result |  |  | 24 |
|  |  |  |  |
| Search 9 |  |  |  |
| ALL of these words | Title | care |  |
| ANY of these words | Document | "cystic fibrosis" "mucoviscidosis" "pancreatic fibrosis" "fabry disease" "fabry's disease" "angiokeratoma corporis diffusum" "alpha-galactosidase a deficiency" "gla deficiency" "angiokeratoma diffuse" "hyperoxaluria" "oxalosis" "neurofibromatosis" "von recklinghausen's disease" "von recklinghausen disease" "von recklinghausen's syndrome" "von recklinghausen syndrome" "gaucher disease" "gaucher's disease" "cerebroside lipidosis syndrome" "gaucher splenomegaly" "glucocerebrosidase deficiency" "glucocerebrosidosis" "glucosylceramidase deficiency" "glucosyl cerebroside lipidosis" "kerasin lipoidosis" "kerasin thesaurismosis" "lipid histiocytosis (kerasin type)" "sphingolipidosis 1" "pompe disease" "pompe's disease" "glycogen storage disease type ii" "acid maltase deficiency" "glycogenosis type ii" "acid alpha-glucosidase deficiency" "coenzyme q10 deficiency" "coq10 deficiency" "leigh syndrome with nephrotic syndrome" "leigh syndrome with nephropathy" "epidermolysis bullosa" "kindler's syndrome" "kindler syndrome" "acrokeratotic poikiloderma" "congenital poikiloderma with blisters AND keratoses" "congenital poikiloderma with bullae AND progressive cutaneous atrophy" "hereditary acrokeratotic poikiloderma" "hyperkeratosis–hyperpigmentation syndrome" "alkaptonuria" "alcaptonuria" "black urine disease" "black bone disease" |  |
| Filter: | Guidelines |  |  |
| Filter: | 2013-2023 |  |  |
| Result |  |  | 5 |
|  |  |  |  |
| Search 10 |  |  |  |
| ALL of these words | Title | healthcare |  |
| ANY of these words | Document | "cystic fibrosis" "mucoviscidosis" "pancreatic fibrosis" "fabry disease" "fabry's disease" "angiokeratoma corporis diffusum" "alpha-galactosidase a deficiency" "gla deficiency" "angiokeratoma diffuse" "hyperoxaluria" "oxalosis" "neurofibromatosis" "von recklinghausen's disease" "von recklinghausen disease" "von recklinghausen's syndrome" "von recklinghausen syndrome" "gaucher disease" "gaucher's disease" "cerebroside lipidosis syndrome" "gaucher splenomegaly" "glucocerebrosidase deficiency" "glucocerebrosidosis" "glucosylceramidase deficiency" "glucosyl cerebroside lipidosis" "kerasin lipoidosis" "kerasin thesaurismosis" "lipid histiocytosis (kerasin type)" "sphingolipidosis 1" "pompe disease" "pompe's disease" "glycogen storage disease type ii" "acid maltase deficiency" "glycogenosis type ii" "acid alpha-glucosidase deficiency" "coenzyme q10 deficiency" "coq10 deficiency" "leigh syndrome with nephrotic syndrome" "leigh syndrome with nephropathy" "epidermolysis bullosa" "kindler's syndrome" "kindler syndrome" "acrokeratotic poikiloderma" "congenital poikiloderma with blisters AND keratoses" "congenital poikiloderma with bullae AND progressive cutaneous atrophy" "hereditary acrokeratotic poikiloderma" "hyperkeratosis–hyperpigmentation syndrome" "alkaptonuria" "alcaptonuria" "black urine disease" "black bone disease" |  |
| Filter: | Guidelines |  |  |
| Filter: | 2013-2023 |  |  |
| Result |  |  | 0 |
|  |  |  |  |
| Search 11 | All Fields | (title:hypogonadism OR title:hypogonadal) AND ("testosterone replacement" OR "testosterone therapy" OR "testosterone treatment" OR trt) |  |
| Filter: | Guidelines |  |  |
| Filter: | 2013-2023 |  |  |
| Result |  |  | 3 |
|  |  |  |  |
| Search 10 |  |  |  |
| ANY of these words | Title | "cystic fibrosis" "mucoviscidosis" "pancreatic fibrosis" "fabry disease" "fabry's disease" "angiokeratoma corporis diffusum" "alpha-galactosidase a deficiency" "gla deficiency" "angiokeratoma diffuse" "hyperoxaluria" "oxalosis" "neurofibromatosis" "von recklinghausen's disease" "von recklinghausen disease" "von recklinghausen's syndrome" "von recklinghausen syndrome" "gaucher disease" "gaucher's disease" "cerebroside lipidosis syndrome" "gaucher splenomegaly" "glucocerebrosidase deficiency" "glucocerebrosidosis" "glucosylceramidase deficiency" "glucosyl cerebroside lipidosis" "kerasin lipoidosis" "kerasin thesaurismosis" "lipid histiocytosis (kerasin type)" "sphingolipidosis 1" "pompe disease" "pompe's disease" "glycogen storage disease type ii" "acid maltase deficiency" "glycogenosis type ii" "acid alpha-glucosidase deficiency" "coenzyme q10 deficiency" "coq10 deficiency" "leigh syndrome with nephrotic syndrome" "leigh syndrome with nephropathy" "epidermolysis bullosa" "kindler's syndrome" "kindler syndrome" "acrokeratotic poikiloderma" "congenital poikiloderma with blisters AND keratoses" "congenital poikiloderma with bullae AND progressive cutaneous atrophy" "hereditary acrokeratotic poikiloderma" "hyperkeratosis–hyperpigmentation syndrome" "alkaptonuria" "alcaptonuria" "black urine disease" "black bone disease" |  |
| Filter: | Systematic Reviews | |  |
| Result |  |  | 65 |
|  |  |  |  |
| Search 11 | All Fields | (title:hypogonadism OR title:hypogonadal) AND ("testosterone replacement" OR "testosterone therapy" OR "testosterone treatment" OR trt) |  |
| Filter: | Systematic Reviews | |  |
| Result |  |  | 29 |
|  |  |  |  |
| Total (including duplicates) | |  | 130 |

AWMF Leitlinienregister (register.awmf.org/) 3 July 2023

| Search | Status | Dokumenttyp | Entwicklungs-stufe | Results |
| --- | --- | --- | --- | --- |
| Mukoviszidose | Aktuelle Leitlinien | Leitlinie (Langfassung) | S3 | 22 |
| Morbus Fabry | Aktuelle Leitlinien | Leitlinie (Langfassung) | S3 | 2 |
| Hyperoxalurie | Aktuelle Leitlinien | Leitlinie (Langfassung) | S3 | 0 |
| Neurofibromatose | Aktuelle Leitlinien | Leitlinie (Langfassung) | S3 | 8 |
| Morbus Gaucher | Aktuelle Leitlinien | Leitlinie (Langfassung) | S3 | 7 |
| Morbus Pompe | Aktuelle Leitlinien | Leitlinie (Langfassung) | S3 | 1 |
| conenzym q10 | Aktuelle Leitlinien | Leitlinie (Langfassung) | S3 | 0 |
| Epidermolysis bullosa | Aktuelle Leitlinien | Leitlinie (Langfassung) | S3 | 2 |
| Alkaptonuria | Aktuelle Leitlinien | Leitlinie (Langfassung) | S3 | 0 |
| seltene Krankheiten | Aktuelle Leitlinien | Leitlinie (Langfassung) | S3 | 152 |
|  |  |  |  |  |
| Total |  |  |  | 194 |

Guidelines International Network (GIN) Library (guidelines.ebmportal.com) 3 July 2023

| Search | Results |
| --- | --- |
| Cystic fibrosis | 7 |
| Fabry disease | 0 |
| Hyperoxaluria | 0 |
| Neurofibromatosis | 3 |
| Gaucher disease | 1 |
| Pompe disease | 1 |
| Coenzyme Q10 deficiency | 9 |
| Epidermolysis bullosa | 2 |
| Alkaptonuria | 0 |
| rare disease | 1 |
| orphan disease | 0 |
|  |  |
| Total | 24 |

NCBI Bookshelf (<https://www.ncbi.nlm.nih.gov/books/>) 4 July 2023

| Search | Query | Items found |
| --- | --- | --- |
| #6 | Search ((("agency for healthcare research and quality"[Publisher])) OR (("national institute for health and care excellence"[Publisher] OR "national institute for health and care excellence nice"[Publisher] OR "national institute for health and clinical excellence"[Publisher] OR "national institute for health and clinical excellence nice"[Publisher]))) OR ("canadian agency for drugs and technologies in health"[Publisher]) | 152528 |
| #7 | Search ("Cystic fibrosis"[Title] OR "Mucoviscidosis"[Title] OR "pancreatic fibrosis"[Title] OR "Fabry disease"[Title] OR "Fabry's disease"[Title] OR "angiokeratoma corporis diffusum"[Title] OR "alpha-galactosidase A deficiency"[Title] OR "GLA deficiency"[Title] OR "angiokeratoma diffuse"[Title] OR "Hyperoxaluria"[Title] OR "Oxalosis"[Title] OR "Neurofibromatosis"[Title] OR "Von Recklinghausen's disease"[Title] OR "Von Recklinghausen disease"[Title] OR "von Recklinghausen's syndrome"[Title] OR "von Recklinghausen syndrome"[Title] OR "Gaucher disease"[Title] OR "Gaucher's disease"[Title] OR "cerebroside lipidosis syndrome"[Title] OR "Gaucher splenomegaly"[Title] OR "glucocerebrosidase deficiency"[Title] OR "glucocerebrosidosis"[Title] OR "glucosylceramidase deficiency"[Title] OR "glucosyl cerebroside lipidosis"[Title] OR "kerasin lipoidosis"[Title] OR "kerasin thesaurismosis"[Title] OR "lipid histiocytosis (kerasin type)"[Title] OR "sphingolipidosis 1"[Title] OR "Pompe disease"[Title] OR "Pompe's disease"[Title] OR "Glycogen storage disease type II"[Title] OR "acid maltase deficiency"[Title] OR "Glycogenosis type II"[Title] OR "acid alpha-glucosidase deficiency"[Title] OR "Coenzyme Q10 deficiency"[Title] OR "CoQ10 deficiency"[Title] OR "Leigh syndrome with nephrotic syndrome"[Title] OR "Leigh syndrome with nephropathy"[Title] OR "Epidermolysis bullosa"[Title] OR "Kindler's syndrome"[Title] OR "Kindler syndrome"[Title] OR "Acrokeratotic poikiloderma"[Title] OR "Congenital poikiloderma with blisters[Title] AND keratoses"[Title] OR "Congenital poikiloderma with bullae[Title] AND progressive cutaneous atrophy"[Title] OR "Hereditary acrokeratotic poikiloderma"[Title] OR "Hyperkeratosisâ€“hyperpigmentation syndrome"[Title] OR "Alkaptonuria"[Title] OR "alcaptonuria"[Title] OR "Black urine disease"[Title] OR "black bone disease")[Title] OR abstract:("Cystic fibrosis"[Title] OR "Mucoviscidosis"[Title] OR "pancreatic fibrosis"[Title] OR "Fabry disease"[Title] OR "Fabry's disease"[Title] OR "angiokeratoma corporis diffusum"[Title] OR "alpha-galactosidase A deficiency"[Title] OR "GLA deficiency"[Title] OR "angiokeratoma diffuse"[Title] OR "Hyperoxaluria"[Title] OR "Oxalosis"[Title] OR "Neurofibromatosis"[Title] OR "Von Recklinghausen's disease"[Title] OR "Von Recklinghausen disease"[Title] OR "von Recklinghausen's syndrome"[Title] OR "von Recklinghausen syndrome"[Title] OR "Gaucher disease"[Title] OR "Gaucher's disease"[Title] OR "cerebroside lipidosis syndrome"[Title] OR "Gaucher splenomegaly"[Title] OR "glucocerebrosidase deficiency"[Title] OR "glucocerebrosidosis"[Title] OR "glucosylceramidase deficiency"[Title] OR "glucosyl cerebroside lipidosis"[Title] OR "kerasin lipoidosis"[Title] OR "kerasin thesaurismosis"[Title] OR "lipid histiocytosis (kerasin type)"[Title] OR "sphingolipidosis 1"[Title] OR "Pompe disease"[Title] OR "Pompe's disease"[Title] OR "Glycogen storage disease type II"[Title] OR "acid maltase deficiency"[Title] OR "Glycogenosis type II"[Title] OR "acid alpha-glucosidase deficiency"[Title] OR "Coenzyme Q10 deficiency"[Title] OR "CoQ10 deficiency"[Title] OR "Leigh syndrome with nephrotic syndrome"[Title] OR "Leigh syndrome with nephropathy"[Title] OR "Epidermolysis bullosa"[Title] OR "Kindler's syndrome"[Title] OR "Kindler syndrome"[Title] OR "Acrokeratotic poikiloderma"[Title] OR "Congenital poikiloderma with blisters[Title] AND keratoses"[Title] OR "Congenital poikiloderma with bullae[Title] AND progressive cutaneous atrophy"[Title] OR "Hereditary acrokeratotic poikiloderma"[Title] OR "Hyperkeratosisâ€“hyperpigmentation syndrome"[Title] OR "Alkaptonuria"[Title] OR "alcaptonuria"[Title] OR "Black urine disease"[Title] OR "black bone disease"[Title]) | 58 |
| #8 | Search (((rare[Title] OR orphan[Title])) AND (multi-organ[Title] OR multiorgan[Title] OR systemic [Title] OR multisystem [Title] OR multi-system [Title] OR genetic[Title] OR hereditary[Title] OR metabolic[Title] OR endocrine[Title])) AND (disease*[Title] OR disorder*[Title] OR syndrome*[Title] OR condition*[Title]) | 4 |
| #9 | Search ((hypogonadism[Title] OR hypogonadal[Title])) AND ("Testosterone replacement" OR "Testosterone therapy" OR "Testosterone treatment" OR TRT) | 3 |
| #10 | Search (#7 OR #8 OR #9) | 65 |
| #11 | Search (#6 AND #10) | 9 |
| #13 | Search #11 Schema: oldbooks | 9 |
| #14 | Search (#6 AND #10) Filters: Publication date from 2013/01/01 to 2023/12/31 | 8 |
|  | Number of unique documents | 2 |

## **Round 3**

Ovid MEDLINE(R) ALL 1946 to July 06, 2023

|  | **#** | **Searches** | **Results** |
| --- | --- | --- | --- |
| A. eye drops/eye diseases | 1 | exp Ophthalmic Solutions/ | 17540 |
|  | 2 | exp Eye Diseases/ | 636796 |
|  | 3 | (eye? or ocular or Ophthalm*).ti,ab,kf,jw. | 752894 |
|  | 4 | 1 or 2 or 3 | 1010251 |
| B. Benzalkonium chloride | 5 | Benzalkonium Compounds/ | 2536 |
|  | 6 | Benzal?onium.ti,ab,kf,nm. | 4160 |
|  | 7 | alkyldimethylbenzylammonium.ti,ab,kf,nm. | 47 |
|  | 8 | ((BZK or BKC or BAK or BAC or ADBAC) and (preserv* or drop? or solution?)).ti,ab,kf,nm. | 1197 |
|  | 9 | or/5-8 | 4708 |
| A+B | 10 | 4 and 9 | 1200 |
| Filter: humans | 11 | limit 10 to "humans only (removes records about animals)" | 852 |
| Filter: language | 12 | (english or german).lg. | 32019035 |
| A+B, limited to humans, language | 13 | 11 and 12 | 789 |
| C. SRs | 14 | (((systematic* and review?) or Systematic overview* or ((Cochrane or systemic or scoping or mapping or Umbrella) adj review*) or ((Cochrane or systemic or scoping or mapping or Umbrella) adj literature review*) or "review of reviews" or "overview of reviews" or meta-review or (integrat* adj (review or overview)) or meta-synthes?s or metasynthes?s or "quantitative review" or "quantitative synthesis" or "research synthesis" or meta-ethnography or "Systematic literature search" or "Systematic literature research" or meta-analys?s or metaanalys?s or "meta-analytic review" or "meta-analytical review").ti,kf,bt. or meta-analysis.pt. or Network Meta-Analysis/ or ((search* or medline or pubmed or embase or Cochrane or scopus or "web of science" or "sources of information" or "data sources" or "following databases") and ("study selection" or "selection criteria" or "eligibility criteria" or "inclusion criteria" or "exclusion criteria")).tw. or "systematic review".pt.) not ((letter or editorial or comment or "case reports" or "historical article").pt. or report.ti. or protocol.ti. or protocols.ti. or withdrawn.ti. or "retraction of publication".pt. or exp "retraction of publication as topic"/ or "retracted publication".pt. or reply.ti. or "published erratum".pt.) | 409099 |
| Search1: A+B+C, limited to humans, language | 15 | 13 and 14 | 11 |
| E. Fanconi Syndrome | 16 | Fanconi Syndrome/ | 1738 |
|  | 17 | (fanconi* not fanconi an?emia).ti,ab,kf. | 3192 |
|  | 18 | 16 or 17 | 3690 |
| F. calcium supplementation | 19 | exp Calcium/ | 283450 |
|  | 20 | Dietary Supplements/ | 74077 |
|  | 21 | calcium*.ti,ab,kf. | 426827 |
|  | 22 | supplement*.ti. | 81496 |
|  | 23 | ((nutrition* or diet*) and supplement*).ti,ab,kf. | 124346 |
|  | 24 | or/19-23 | 732344 |
| E+F | 25 | 18 and 24 | 203 |
| Filter: humans | 26 | limit 25 to "humans only (removes records about animals)" | 182 |
| Filter: language | 27 | (english or german).lg. | 32019035 |
| E+F, limited to humans, language | 28 | 26 and 27 | 149 |
| Search 2: E+F, limited to humans, language, since 1980 | 29 | limit 28 to yr="1980 -Current" | 119 |
| Combined search result Searches 1 and 2 | 30 | 15 or 29 | 130 |

Cochrane Database of Systematic Reviews Issue 7 of 12, July 2023

Cochrane Central Register of Controlled Trials Issue 7 of 12, July 2023

| ID | Search | Hits |
| --- | --- | --- |
| #1 | [mh "Ophthalmic Solutions"] or [mh "Eye Diseases"] | 26148 |
| #2 | (eye?:ti,ab,kw OR ocular:ti,ab,kw OR Ophthalm*:ti,ab,kw) | 55171 |
| #3 | #1 or #2 | 62816 |
| #4 | [mh ^"Benzalkonium Compounds"] | 145 |
| #5 | Benzal?onium:ti,ab,kw or alkyldimethylbenzylammonium:ti,ab,kw | 396 |
| #6 | ((BZK:ti,ab,kw OR BKC:ti,ab,kw OR BAK:ti,ab,kw OR BAC:ti,ab,kw OR ADBAC:ti,ab,kw) AND (preserv*:ti,ab,kw OR drop?:ti,ab,kw OR solution?:ti,ab,kw)) | 150 |
| #7 | #4 or #5 or #6 | 452 |
| #8 | #3 and #7 in Cochrane Reviews | 2 |
| #9 | [mh ^"Fanconi Syndrome"] | 7 |
| #10 | (fanconi*:ti,ab,kw NOT ("fanconi" NEXT an?emia):ti,ab,kw) | 57 |
| #11 | #9 or #10 | 57 |
| #12 | [mh Calcium] or [mh ^"Dietary Supplements"] | 17592 |
| #13 | calcium*:ti,ab,kw | 33811 |
| #14 | supplement*:ti | 33520 |
| #15 | ((nutrition*:ti,ab,kw OR diet*:ti,ab,kw) AND supplement*:ti,ab,kw) | 39747 |
| #16 | (or #12-#15) | 83456 |
| #17 | #11 and #16 with Publication Year from 1980 to 2023, in Trials | 4 |
| #18 | #8 or #17 | 6 |

Epistemonikos.org 3 July 2023

| Search | Search | Results |
| --- | --- | --- |
|  | (title:(eye OR eyes OR ocular OR Ophthalm*) OR abstract:(eye OR eyes OR ocular OR Ophthalm*)) AND (title:(Benzalkonium OR Benzalconium OR alkyldimethylbenzylammonium OR ((BZK OR BKC OR BAK OR BAC OR ADBAC) AND (preserv* OR drop* OR solution*))) OR abstract:(Benzalkonium OR Benzalconium OR alkyldimethylbenzylammonium OR ((BZK OR BKC OR BAK OR BAC OR ADBAC) AND (preserv* OR drop* OR solution*)))) | 100 |
|  | Filter: Systematic Review | 15 |
| URL | https://www.epistemonikos.org/advanced_search?q=(title:(eye%20OR%20eyes%20OR%20ocular%20OR%20Ophthalm*)%20OR%20abstract:(eye%20OR%20eyes%20OR%20ocular%20OR%20Ophthalm*))%20AND%20(title:(Benzalkonium%20OR%20Benzalconium%20OR%20alkyldimethylbenzylammonium%20OR%20((BZK%20OR%20BKC%20OR%20BAK%20OR%20BAC%20OR%20ADBAC)%20AND%20(preserv*%20OR%20drop*%20OR%20solution*)))%20OR%20abstract:(Benzalkonium%20OR%20Benzalconium%20OR%20alkyldimethylbenzylammonium%20OR%20((BZK%20OR%20BKC%20OR%20BAK%20OR%20BAC%20OR%20ADBAC)%20AND%20(preserv*%20OR%20drop*%20OR%20solution*))))&protocol=no&classification=systematic-review |  |

## **Additional searches**

29 June 2023

| **Webpage searches** | | | | |
| --- | --- | --- | --- | --- |
| Institution | search | Guideline Title | Year | URL |
| NICE | Chronic kidney disease | Chronic kidney disease: assessment and management | 2021 | https://www.nice.org.uk/guidance/ng203 |
| NICE | kidney transplant | N/A |  |  |
| NICE | Renal replacement | Renal replacement therapy and conservative management | 2018 | https://www.nice.org.uk/guidance/ng107 |
| AHRQ | Chronic kidney disease | N/A |  |  |
| AHRQ | kidney transplant | N/A |  |  |
| AHRQ | Renal replacement | N/A |  |  |
| CADTH | Chronic kidney disease | N/A |  |  |
| CADTH | kidney transplant | N/A |  |  |
| CADTH | Renal replacement | N/A |  |  |
| **Database searches** | | | | |
| Institution | Search | Results |  | Results list |
| GIN | Chronic kidney disease | 9 |  | https://guidelines.ebmportal.com/guidelines-international-network?fv%5B64%5D%5B0%5D=1569&g=collection_field_3&search=Chronic%20kidney%20disease&s=asc |
| GIN | kidney transplant | 3 |  | https://guidelines.ebmportal.com/guidelines-international-network?search=kidney%20transplant&type=search |
| GIN | Renal replacement | 7 |  | https://guidelines.ebmportal.com/guidelines-international-network?search=Renal%20replacement&type=search |

# List of excluded studies

## **Ineligible study design (n=34)**

1. Al-Sharefi A, Quinton R. Current National and International Guidelines for the Management of Male Hypogonadism: Helping Clinicians to Navigate Variation in Diagnostic Criteria and Treatment Recommendations. Endocrinol Metab (Seoul). 2020;35(3):526-40.

2. Al-Yateem N. Guidelines for the transition from child to adult cystic fibrosis care. Nurs Child Young People. 2013;25(5):29-34.

3. Alsuhaibani AH, Wagoner MD, Khan AO. Confocal microscopy of the cornea in nephropathic cystinosis [3]. Br J Ophthalmol. 2005;89(11):1530-1.

4. Anonymous. Canadian Agency for Drugs and Technologies in Health. 2018;02:02.

5. Antonini TM, Girard M, Habes D, Goria O, Debray D. Optimization of the transition process of youth with liver disease in adulthood: A position paper from FILFOIE, the French network for paediatric and adult rare liver diseases. Clin Res Hepatol Gastroenterol. 2020;44(2):135-41.

6. Beinart N, Hackett RA, Graham CD, Weinman J, Ostermann M. Mood and illness experiences of adults with cystinosis. Ren Fail. 2015;37(5):835-9.

7. Belzer LT, Wright SM, Goodwin EJ, Singh MN, Carter BS. Psychosocial Considerations for the Child with Rare Disease: A Review with Recommendations and Calls to Action. Children (Basel). 2022;9(7):21.

8. Chabli A, Aupetit J, Raehm M, Ricquier D, Chadefaux-Vekemans B. Measurement of cystine in granulocytes using liquid chromatography-tandem mass spectrometry. Clin Biochem. 2007;40(9-10):692-8.

9. Chabrol B, Jacquin P, Francois L, Broue P, Dobbelaere D, Douillard C, et al. Transition from pediatric to adult care in adolescents with hereditary metabolic diseases: Specific guidelines from the French network for rare inherited metabolic diseases (G2M). Arch Pediatr. 2018;15:15.

10. Choukair D, Hauck F, Bettendorf M, Krude H, Klein C, Baumer T, et al. An Integrated clinical pathway for diagnosis, treatment and care of rare diseases: model, operating procedures, and results of the project TRANSLATE-NAMSE funded by the German Federal Joint Committee. Orphanet J Rare Dis. 2021;16(1):474.

11. Corona G, Rastrelli G, Morgentaler A, Sforza A, Mannucci E, Maggi M. Meta-analysis of Results of Testosterone Therapy on Sexual Function Based on International Index of Erectile Function Scores. European Urology. 2017;72(6):1000-11.

12. Doyle M. Peer Support and Mentorship in a US Rare Disease Community: Findings from the Cystinosis in Emerging Adulthood Study. Patient. 2015;8(1):65-73.

13. Doyle M, Werner-Lin A. That eagle covering me: transitioning and connected autonomy for emerging adults with cystinosis. Pediatr Nephrol. 2015;30(2):281-91.

14. Fode M, Salonia A, Minhas S, Burnett AL, Shindel AW. Late-onset Hypogonadism and Testosterone Therapy - A Summary of Guidelines from the American Urological Association and the European Association of Urology. Eur Urol Focus. 2019;5(4):539-44.

15. Gahl WA, Kuehl EM, Iwata F, Lindblad A, Kaiser-Kupfer MI. Corneal crystals in nephropathic cystinosis: natural history and treatment with cysteamine eyedrops. Mol Genet Metab. 2000;71(1-2):100-20.

16. Graziano S, Spano B, Majo F, Righelli D, Vincenzina L, Quittner A, et al. Rates of depression and anxiety in Italian patients with cystic fibrosis and parent caregivers: Implementation of the Mental Health Guidelines. Respir Med. 2020;172:106147.

17. Guo C, Gu W, Liu M, Peng BO, Yao X, Yang B, et al. Efficacy and safety of testosterone replacement therapy in men with hypogonadism: A meta-analysis study of placebo-controlled trials. Experimental Ther. 2016;11(3):853-63.

18. Has C, El Hachem M, Buckova H, Fischer P, Friedova M, Greco C, et al. Practical management of epidermolysis bullosa: consensus clinical position statement from the European Reference Network for Rare Skin Diseases. J Eur Acad Dermatol Venereol. 2021;35(12):2349-60.

19. Hohenfellner K, Deerberg-Wittram J. Coordinated, Cost-effective Care for Rare Disease: The Cystinosis Outpatient Consultation Program at RoMed. NEJM Catalyst Innovations in Care Delivery. 2020;1(4):1-.

20. Inhestern L, Brandt M, Otto R, Zybarth D, Harter M, Bergelt C. [Healthcare for people with rare diseases: recommendations for successful intersectoral collaboration]. Bundesgesundheitsblatt Gesundheitsforschung Gesundheitsschutz. 2023;13:13.

21. Jayasena CN, Anderson RA, Llahana S, Barth JH, MacKenzie F, Wilkes S, et al. Society for Endocrinology guidelines for testosterone replacement therapy in male hypogonadism. Clin Endocrinol (Oxf). 2022;96(2):200-19.

22. Kolthof HJ, Tjeenk-Kalff AC. A nine-year-old girl with cystinosis and severe memory problems: A drug or disease effect? Journal of Pediatric Neurology. 2007;5(4):335-41.

23. Kowalczyk M, Toro MD, Rejdak R, Sikora P. Diagnostics and treatment of ocular complications in infantile nephropathic cystinosis. Ophthalmology Journal (2450-7873). 2021:206-14.

24. Kwong JCC, Krakowsky Y, Grober E. Testosterone Deficiency: A Review and Comparison of Current Guidelines. J Sex Med. 2019;16(6):812-20.

25. Laney DA, Bennett RL, Clarke V, Fox A, Hopkin RJ, Johnson J, et al. Fabry disease practice guidelines: recommendations of the National Society of Genetic Counselors. J Genet Couns. 2013;22(5):555-64.

26. Morgentaler A, Traish A, Hackett G, Jones TH, Ramasamy R. Diagnosis and Treatment of Testosterone Deficiency: Updated Recommendations From the Lisbon 2018 International Consultation for Sexual Medicine. Sex Med Rev. 2019;7(4):636-49.

27. Pinxten AM, Hua MT, Simpson J, Hohenfellner K, Levtchenko E, Casteels I. Clinical Practice: A Proposed Standardized Ophthalmological Assessment for Patients with Cystinosis. Ophthalmol Ther. 2017;6(1):93-104.

28. Raub W. Cysteamine effective against corneal complications of cystinosis. Journal of the American Medical Association. 1990;264(13):1650.

29. Schierbeek H, Bijsterveld K, Chapman TE, van Luijk WH, Reijngoud DJ, Berger R. Stable isotope dilution analysis of cystine in granulocyte suspensions as cysteine: a powerful method for the diagnosis, the follow-up, and treatment of patients with cystinosis. Clin Chim Acta. 1990;191(1-2):39-47.

30. Spencer-Tansley R, Meade N, Ali F, Simpson A, Hunter A. Mental health care for rare disease in the UK - recommendations from a quantitative survey and multi-stakeholder workshop. BMC Health Serv Res. 2022;22(1):648.

31. Spilkin A, Ballantyne A. Behavior in children with a chronic illness: A descriptive study of child characteristics, family adjustment, and school issues in children with cystinosis. Families, Systems and Health. 2007;25(1):68-84.

32. Twitchell DK, Pastuszak AW, Khera M. Controversies in Testosterone Therapy. Sex Med Rev. 2021;9(1):149-59.

33. Ward AJ, Murphy D, Marron R, McGrath V, Bolz-Johnson M, Cullen W, et al. Designing rare disease care pathways in the Republic of Ireland: a co-operative model. Orphanet J Rare Dis. 2022;17(1):162.

34. Zoch M, Sedlmayr B, Knapp A, Bathelt F, Helfer S, Schmitt J, et al. [Interdisciplinary care path and potential IT support for people with rare diseases in Germany]. Z. 2021;165:68-76.

## **Ineligible publication type (n=36)**

1. Alcantara Montero A. Management of male hypogonadism and testosterone therapy: European Association of Urology position statement on the role of the urologist. Actas Urol Esp (Engl Ed). 2018;42(3):212-3.

2. Alexander E. Nephropathic cystinosis: a case report. Am Fam Physician. 1991;43(5):1533-6.

3. Alsuhaibani AH, Kahn AO, Wagoner MD. Erratum: Confocal microscopy of the cornea in nephropathic cystinosis (British Journal of Ophthalmology (2005) 89 (1530-1531)). Br J Ophthalmol. 2006;90(1):125.

4. Andrews PA, Sacks SH, van't Hoff W. Successful pregnancy in cystinosis. Jama. 1994;272(17):1327-8.

5. Badawy SM, Vasileva A, Bhat RV, Gong S, Liem RI. A case of severe bi-cytopenias and hypocellular bone marrow with uncontrolled nephropathic cystinosis. Pediatr Blood Cancer. 2021;68(6):e28952.

6. Baumner S, Weber LT. Conversion from immediate- to extended-release cysteamine may decrease disease control and increase additional side effects. Pediatr Nephrol. 2017;32(7):1281-2.

7. Betend B, David L, Pugeaut R, Francois R. Infantile cystinosis: A 3-year indomethacin (IDM) treatment. Pediatr Res. 1981;15(8):1190.

8. Castro R, Berjonneau E, Courbier S. Learning from the Pandemic to Improve Care for Vulnerable Communities: The Perspectives and Recommendations from the Rare Disease Community. Int J Integr Care. 2021;21(1):12.

9. Euctr F. CrYSTobs A cohort of patients with cystinosis : compliance to cysteamine and neurological complications An auxiliary study to Raptor RP103 03 and RP103 04 - CrYSTobs. https://trialsearchwhoint/Trial2aspx?TrialID=EUCTR2010-020098-18-FR. 2010.

10. Euctr F. A Randomized, Crossover, Pharmacokinetic and Pharmacodynamic Study to Determine the Safety and Efficacy of Cysteamine Bitartrate Delayed-release Capsules (RP103), Compared to Cystagon® in Patients with Nephropathic Cystinosis. https://trialsearchwhoint/Trial2aspx?TrialID=EUCTR2009-017882-42-FR. 2010.

11. Euctr F. Cysteamine Hydrochloride for nephrOpathic Cystinosis, open-label Phase III pivotal study. https://trialsearchwhoint/Trial2aspx?TrialID=EUCTR2009-012564-13-FR. 2013.

12. Euctr N. A Long-Term, Open-Label, Safety and Efficacy Study of Cysteamine Bitartrate Delayed-release Capsules (RP103) in Patients with Nephropathic Cystinosis. https://trialsearchwhoint/Trial2aspx?TrialID=EUCTR2010-018365-34-NL. 2010.

13. EUCTR2007-006024-35-FR. Adaptive dose regimen of Cystadrops for cOrneal Crystal deposiTs and ocular manifestations in nephropathic cystinosis : an open label, dose-response pilot study - CYSTADROPS OCT-1 pilot study. 2007.

14. EUCTR2009-012564-13-FR. Cysteamine Hydrochloride for nephrOpathic Cystinosis, open-label Phase III pivotal study&#x0D;. 2013.

15. EUCTR2010-018365-34-FR. A Long-Term, Open-Label, Safety and Efficacy Study of Cysteamine Bitartrate Delayed-release Capsules (RP103) in Patients with Nephropathic Cystinosis - RP103-04. 2010.

16. EUCTR2013-003228-35-ES. Study of the preliminary efficacy and safety of topical cysteamine formulated in viscous solution in cystinosis patients. 2013.

17. EUCTR2018-002984-24-FR. Open-label, Single-arm, Multicenter Study to Assess the Safety of Cystadrops® in Pediatric Cystinosis Patients from 6 Months to Less Than 2 Years Old. 2019.

18. EUCTR2018-002984-24-GB. Open-label, Single-arm, Multicenter Study to Assess the Safety of, Cystadrops® in Pediatric Cystinosis Patients,from 6 Months to Less Than 2 Years Old [SCOB2 Study]. 2019.

19. Gahl WA, Thoene JG, Schneider JA, O'Regan S, Kaiser-Kupfer MI, Kuwabara T. NIH conference. Cystinosis: progress in a prototypic disease. Ann Intern Med. 1988;109(7):557-69.

20. Horizon Pharma USA I. Pilot Study of Safety, Tolerability, Pharmacokinetics/Pharmacodynamics of RP103 Compared to Cystagon® in Patients With Cystinosis. https://ClinicalTrials.gov/show/NCT00872729; 2009.

21. Horizon Pharma USA I. Open-Label, Safety and Superior Effectiveness Study of Cysteamine Bitartrate Delayed-Release Capsules (RP103) in Cystinosis. https://ClinicalTrials.gov/show/NCT01733316; 2013.

22. Iwata F, Wozencraft LA, Caruso RC, Li A, Gahl WA, McCain LM, et al. Nephropathic cystinosis: natural history of ocular findings and results of clinical trial of cysteamine eye drop intervention. Iovs. 1995; 36:ARVO Abstract 4869.

23. JPRN-jRCT2021200029. A0003 Eye Drops 0.55% Japan Phase III Study. 2020.

24. Kaps D, VÃ¶lcker HE, Tetz MR. Topical Cysteamine Treatment of Corneal Cystine Crystal Deposits in Juvenile Cystinosis. American academy of ophthalmology. 1990:137.

25. Kasimer RN, Langman CB. Adult complications of nephropathic cystinosis: a systematic review. Pediatr Nephrol. 2021;36(2):223-36.

26. Langman C. Erratum to A randomized controlled crossover trial with delayed-release cysteamine bitartrate in nephropathic cystinosis: Effectiveness on white blood cell cystine levels and comparison of safety (Clin J Am Soc Nephrol,(2012), 7, (1112-1120)). Clinical Journal of the American Society of Nephrology. 2013;8(3):468.

27. Lemire J, Kaplan BS. Prolonged use of indomethacin in cystinosis. Pediatr Res. 1981;15(4 II):No. 1517.

28. Lyon HCd. A Cohort of Patients With Cystinosis : Compliance to Cysteamine and Neurological Complications. https://ClinicalTrials.gov/show/NCT02012114; 2011.

29. Namratha Vaghdevi C, Jahnavi B, Sindhu D, P AL, R R, Shah M, et al. Cystinosis: A 6.5-Year-Follow-up Study. Indian J Pediatr. 2022;89(8):824.

30. Nct. Randomized Study of New Formulation Ophthalmic Cysteamine Hydrochloride for Corneal Cystine Accumulation in Patients With Cystinosis. https://clinicaltrialsgov/show/NCT00010426. 2001.

31. Nct. Phase 3 Study of Cysteamine Bitartrate Delayed-release (RP103) Compared to Cystagon® in Patients With Cystinosis. https://clinicaltrialsgov/show/NCT01000961. 2009.

32. Nct. Long-Term Safety Follow-up Study of Cysteamine Bitartrate Delayed-release Capsules (RP103). https://clinicaltrialsgov/show/NCT01197378. 2010.

33. SA/NV C. Observational Study to Assess the Quality of Life in Nephropathic Cystinosis Patients. https://ClinicalTrials.gov/show/NCT04246060; 2020.

34. Steinherz R. Cystinosis and vitamin D. Arch Dis Child. 1988;63(12):1519.

35. University S. The Effect of Exercise on Muscle Dysfunction in Cystinosis. https://ClinicalTrials.gov/show/NCT04071548; 2019.

36. Williams R. Esomeprazole for cysteamine- induced gastrointestinal symptoms in children with cystinosis. Nature Clinical Practice Nephrology. 2006;2(2):63.

## **Ineligible study population (n=16+2)**

1. Becker J, Ravens E, Pape L, Becker J, Ravens E, Ernst G. Somatic outcomes of young people with chronic diseases participating in transition programs: A systematic review. J Transit Med. 2020;2(1).

2. Buelt A. Testosterone Therapy for Age-Related Low Testosterone: Guidelines from the ACP. Am Fam Physician. 2021;103(1):60-1.

3. Crowley R, Wolfe I, Lock K, McKee M. Improving the transition between paediatric and adult healthcare: a systematic review. Arch Dis Child. 2011;96(6):548-53.

4. Dimitropoulos K, Verze P, Van den Broeck T, Salonia A, Yuan CY, Hatzimouratidis K, et al. What are the benefits and harms of testosterone therapy for male sexual dysfunction?-a systematic review. Int J Impot Res. 2019;31(6):380-91.

5. Ebel F, Greuter L, Guzman R, Soleman J. Transitional Care in Pediatric Brain Tumor Patients: A Systematic Literature Review. Children (Basel). 2022;9(4):02.

6. Garcia-Rodriguez F, Raygoza-Cortez K, Moreno-Hernandez L, Garcia-Perez R, Garza Lopez LE, Arana-Guajardo AC, et al. Outcomes of transitional care programs on adolescent chronic inflammatory systemic diseases: systematic review and meta-analyses. Pediatr. 2022;20(1):15.

7. Guo Y, Zhou YH, Wu XP, Tang CY, Wang M, Mo ZH, et al. Changes in Bone Mineral Density Following Conventional Oral Phosphonate Treatment of Hypophosphatemic Osteomalacia: A Non-Randomized Controlled Study. Int J Gen Med. 2021;14:7925-31.

8. Jackson AC, Liang RP, Frydenberg E, Higgins RO, Murphy BM. Parent education programmes for special health care needs children: a systematic review. J Clin Nurs. 2016;25(11-12):1528-47.

9. Madsen MC, Heijer MD, Pees C, Biermasz NR, Bakker LEH. Testosterone in men with hypogonadism and transgender males: a systematic review comparing three different preparations. Endocrine Connections. 2022;11(8):01.

10. Rice DB, Carboni-Jimenez A, Canedo-Ayala M, Turner KA, Chiovitti M, Levis AW, et al. Perceived Benefits and Facilitators and Barriers to Providing Psychosocial Interventions for Informal Caregivers of People with Rare Diseases: A Scoping Review. Patient. 2020;13(5):471-519.

11. Servais A, Moriniere V, Grunfeld JP, Noel LH, Goujon JM, Chadefaux-Vekemans B, et al. Late-onset nephropathic cystinosis: clinical presentation, outcome, and genotyping. Clin J Am Soc Nephrol. 2008;3(1):27-35.

12. Taniguchi H, Shimada S, Kinoshita H. Testosterone Therapy for Late-Onset Hypogonadism Improves Erectile Function: A Systematic Review and Meta-Analysis. Urologia Internationalis. 2022;106(6):539-52.

13. Udeoji DU, Phan A, Katsiyiannis P, Willix R, Schwarz ER. Topical Testosterone Gel for the Treatment of Male Hypogonadism. Clinical Medicine Insights: Therapeutics. 2012(4):217-30.

14. Vartolomei MD, Kimura S, Vartolomei L, Shariat SF. Systematic Review of the Impact of Testosterone Replacement Therapy on Depression in Patients with Late-onset Testosterone Deficiency. Eur Urol Focus. 2020;6(1):170-7.

15. Waldboth V, Patch C, Mahrer-Imhof R, Metcalfe A. Living a normal life in an extraordinary way: A systematic review investigating experiences of families of young people's transition into adulthood when affected by a genetic and chronic childhood condition. Int J Nurs Stud. 2016;62:44-59.

16. Yeung CH, Santesso N, Zeraatkar D, Wang A, Pai M, Sholzberg M, et al. Integrated multidisciplinary care for the management of chronic conditions in adults: an overview of reviews and an example of using indirect evidence to inform clinical practice recommendations in the field of rare diseases. Haemophilia. 2016;22 Suppl 3:41-50.

**Guidelines from additional searches**

1. Overview | Renal replacement therapy and conservative management | Guidance | NICE [Internet]. NICE; 2018 [cited 2024 Oct 5]. Available from: <https://www.nice.org.uk/guidance/ng107>

2. Overview | Chronic kidney disease: assessment and management | Guidance | NICE [Internet]. NICE; 2021 [cited 2024 Oct 5]. Available from: <https://www.nice.org.uk/guidance/ng203>

## **Ineligible or no intervention (n=19)**

1. Almond PS, Morel P, Troppmann C, Matas A, Najarian JS, Chavers B. Progression of infantile cystinosis after renal transplantation. Transplant Proc. 1991;23(1 Pt 2):1386.

2. Aly R, Makar S, El Bakri A, Soliman NA. Neurocognitive functions and behavioral profiles in children with nephropathic cystinosis. Saudi J Kidney Dis Transpl. 2014;25(6):1224-31.

3. Broyer M, Tete MJ, Guest G, Bertheleme JP, Labrousse F, Poisson M. Clinical polymorphism of cystinosis encephalopathy. Results of treatment with cysteamine. J Inherit Metab Dis. 1996;19(1):65-75.

4. Cabrera-Serrano M, Junckerstorff RC, Alisheri A, Pestronk A, Laing NG, Weihl CC, et al. Cystinosis distal myopathy, novel clinical, pathological and genetic features. Neuromuscul Disord. 2017;27(9):873-8.

5. Coyne I, Sheehan AM, Heery E, While AE. Improving transition to adult healthcare for young people with cystic fibrosis: A systematic review. J Child Health Care. 2017;21(3):312-30.

6. Csorba A, Maka E, Maneschg OA, Szabo A, Szentmary N, Csidey M, et al. Examination of corneal deposits in nephropathic cystinosis using in vivo confocal microscopy and anterior segment optical coherence tomography: an age-dependent cross sectional study. BMC ophthalmol. 2020;20(1):73.

7. Dufier JL, Dhermy P, Gubler MC, Gagnadoux MF, Broyer M. Ocular changes in long-term evolution of infantile cystinosis. Ophthalmic Paediatr Genet. 1987;8(2):131-7.

8. Fink JK, Brouwers P, Barton N, Malekzadeh MH, Sato S, Hill S, et al. Neurologic complications in long-standing nephropathic cystinosis. Arch Neurol. 1989;46(5):543-8.

9. Gahl WA, Ingelfinger J, Mohan P, Bernardini I, Hyman PE, Tangerman A. Intravenous cysteamine therapy for nephropathic cystinosis. Pediatr Res. 1995;38(4):579-84.

10. Gretz N, Manz F, Augustin R, Barrat TM, Bender-Gotze C, Brandis M, et al. Survival time in cystinosis. A collaborative study. Proc Eur Dial Transplant Assoc. 1983;19:582-9.

11. Gultekingil Keser A, Topaloglu R, Bilginer Y, Besbas N. Long-term endocrinologic complications of cystinosis. Minerva Pediatr. 2014;66(2):123-30.

12. Kaiser-Kupfer MI, Caruso RC, Minkler DS, Gahl WA. Long-term ocular manifestations in nephropathic cystinosis. Arch Ophthalmol. 1986;104(5):706-11.

13. Katzir Z, Shvil Y, Landau H, Kidrony G, Popovtzer MM. Nephrogenic diabetes insipidus, cystinosis, and vitamin D. Arch Dis Child. 1988;63(5):548-50.

14. Manz F, Gretz N. Progression of chronic renal failure in a historical group of patients with nephropathic cystinosis. European Collaborative Study on Cystinosis. Pediatr Nephrol. 1994;8(4):466-71.

15. Nichols SL, Press GA, Schneider JA, Trauner DA. Cortical atrophy and cognitive performance in infantile nephropathic cystinosis. Pediatr Neurol. 1990;6(6):379-81.

16. Saeedi R, Jiang SY, Holmes DT, Kendler DL. Fibroblast growth factor 23 is elevated in tenofovir-related hypophosphatemia. Calcified Tissue International. 2014;94(6):665-8.

17. Selvan C, Thukral A, Chakraborthy PP, Bhattacharya R, Roy A, Goswani S, et al. Refractory rickets due to Fanconi's Syndrome secondary to Wilson's disease. Indian Journal of Endocrinology and Metabolism. 2012;16(Suppl 2):S399-401.

18. Sliman GA, Winters WD, Shaw DW, Avner ED. Hypercalciuria and nephrocalcinosis in the oculocerebrorenal syndrome. J Urol. 1995;153(4):1244-6.

19. Wuhl E, Haffner D, Gretz N, Offner G, van't Hoff WG, Broyer M, et al. Treatment with recombinant human growth hormone in short children with nephropathic cystinosis: no evidence for increased deterioration rate of renal function. The European Study Group on Growth Hormone Treatment in Short Children with Nephropathic Cystinosis. Pediatr Res. 1998;43(4 Pt 1):484-8.

## **Ineligible or no comparison (n=3)**

1. Grupcheva CN, Ormonde SE, McGhee C. In vivo confocal microscopy of the cornea in nephropathic cystinosis. Arch Ophthalmol. 2002;120(12):1742-5.

2. Keidel L. Entwicklung von Biomarkern zur Charakterisierung okulärer Manifestationen der Cystinose mittels optischer Kohärenztomographie: Ludwig-Maximilians-Universität München; 2022.

3. Spilkin AM, Ballantyne AO, Trauner DA. Visual and verbal learning in a genetic metabolic disorder. Neuropsychologia. 2009;47(8-9):1883-92.

## **Ineligible outcome(s) (n=43)**

1. Alsuhaibani AH, Khan AO, Wagoner MD. Confocal microscopy of the cornea in nephropathic cystinosis. Br J Ophthalmol. 2005;89(11):1530-1.

2. Ballantyne AO, Spilkin AM, Trauner DA. Executive function in nephropathic cystinosis. Cogn Behav Neurol. 2013;26(1):14-22.

3. Bate KL, Clouston D, Packham D, Ratnaike S, Ebeling PR. Lambda light chain induced nephropathy: a rare cause of the Fanconi syndrome and severe osteomalacia. Am J Kidney Dis. 1998;32(6):E3.

4. Bava S. Reduced microstructural white matter integrity in a genetic metabolic disorder: A diffusion tensor MRI study: eScholarship, University of California; 2007.

5. Bava S, Theilmann RJ, Sach M, May SJ, Frank LR, Hesselink JR, et al. Developmental changes in cerebral white matter microstructure in a disorder of lysosomal storage. Cortex. 2010;46(2):206-16.

6. Becerir T, Girisgen I, Yilmaz N, Gungor O, Un ES, Sagtas E, et al. A case with kidney transplant and cystinosis: Answers. Pediatr Nephrol. 2021;36(7):2145-7.

7. Becerir T, Girisgen I, Yilmaz N, Gungor O, Un ES, Sagtas E, et al. A case with kidney transplant and cystinosis: Questions. Pediatr Nephrol. 2021;36(7):2143-4.

8. Berryhill A, Bhamre S, Chaudhuri A, Concepcion W, Grimm PC. Cysteamine in renal transplantation: A report of two patients with nephropathic cystinosis and the successful re-initiation of cysteamine therapy during the immediate post-transplant period. Pediatr Transplant. 2016;20(1):141-5.

9. Besouw M, Cornelissen E, Cassiman D, Kluijtmans L, van den Heuvel L, Levtchenko E. Carnitine Profile and Effect of Suppletion in Children with Renal Fanconi Syndrome due to Cystinosis. JIMD rep. 2014;16:25-30.

10. Besouw MT, Kremer JA, Janssen MC, Levtchenko EN. Fertility status in male cystinosis patients treated with cysteamine. Fertil Steril. 2010;93(6):1880-3.

11. Besouw MT, Levtchenko EN, Willemsen MA, Noordam K. Growth hormone producing prolactinoma in juvenile cystinosis: a simple coincidence? Pediatr Nephrol. 2008;23(2):307-10.

12. Besouw MT, Schneider J, Janssen MC, Greco M, Emma F, Cornelissen EA, et al. Copper deficiency in patients with cystinosis with cysteamine toxicity. J Pediatr. 2013;163(3):754-60.

13. Colah S, Trauner DA. Tactile recognition in infantile nephropathic cystinosis. Dev Med Child Neurol. 1997;39(6):409-13.

14. Corona G, Giagulli VA, Maseroli E, Vignozzi L, Aversa A, Zitzmann M, et al. THERAPY OF ENDOCRINE DISEASE: Testosterone supplementation and body composition: results from a meta-analysis study. Eur. 2016;174(3):R99-116.

15. Corona G, Rastrelli G, Di Pasquale G, Sforza A, Mannucci E, Maggi M. Testosterone and Cardiovascular Risk: Meta-Analysis of Interventional Studies. J Sex Med. 2018;15(6):820-38.

16. Dureau P, Broyer M, Dufier JL. Evolution of ocular manifestations in nephropathic cystinosis: a long-term study of a population treated with cysteamine. J Pediatr Ophthalmol Strabismus. 2003;40(3):142-6.

17. Fallara G, Pozzi E, Belladelli F, Corsini C, Boeri L, Capogrosso P, et al. Cardiovascular Morbidity and Mortality in Men - Findings From a Meta-analysis on the Time-related Measure of Risk of Exogenous Testosterone. J Sex Med. 2022;19(8):1243-54.

18. Francisco AA, Berruti AS, Kaskel FJ, Foxe JJ, Molholm S. Assessing the integrity of auditory processing and sensory memory in adults with cystinosis (CTNS gene mutations). Orphanet J Rare Dis. 2021;16(1):177.

19. Francisco AA, Foxe JJ, Horsthuis DJ, Molholm S. Impaired auditory sensory memory in Cystinosis despite typical sensory processing: A high-density electrical mapping study of the mismatch negativity (MMN). Neuroimage (Amst). 2020;25:102170.

20. Gahl WA, Bernardini I, Dalakas M, Rizzo WB, Harper GS, Hoeg JM, et al. Oral carnitine therapy in children with cystinosis and renal Fanconi syndrome. J Clin Invest. 1988;81(2):549-60.

21. Garcia-Villoria J, Hernandez-Perez JM, Arias A, Ribes A. Improvement of the cystine measurement in granulocytes by liquid chromatograhy-tandem mass spectrometry. Clin Biochem. 2013;46(3):271-4.

22. Gertsman I, Johnson WS, Nishikawa C, Gangoiti JA, Holmes B, Barshop BA. Diagnosis and Monitoring of Cystinosis Using Immunomagnetically Purified Granulocytes. Clin Chem. 2016;62(5):766-72.

23. Ghane Sharbaf F, Bitzan M, Szymanski KM, Bell LE, Gupta I, Tchervenkov J, et al. Native nephrectomy prior to pediatric kidney transplantation: biological and clinical aspects. Pediatr Nephrol. 2012;27(7):1179-88.

24. Goldschneider KR, Good J, Harrop E, Liossi C, Lynch-Jordan A, Martinez AE, et al. Pain care for patients with epidermolysis bullosa: best care practice guidelines. BMC Med. 2014;12:178.

25. Hohenfellner K, Bergmann C, Fleige T, Janzen N, Burggraf S, Olgemoller B, et al. Molecular based newborn screening in Germany: Follow-up for cystinosis. Mol Genet Metab Rep. 2019;21:100514.

26. Horsthuis DJ, Molholm S, Foxe JJ, Francisco AA. Event-related potential (ERP) evidence of early visual processing differences in cystinosis. bioRxiv. 2023;02:02.

27. Kastrup O, Koeppen S, Schwechheimer K, Keidel M, Diener HC. Myopathy in two siblings with nephropathic cystinosis. Eur J Neurol. 1998;5(6):609-12.

28. King A, Hanley H, Popenhagen M, Perez F, Thompson K, Purvis D, et al. Supporting sexuality for people living with epidermolysis bullosa: clinical practice guidelines. Orphanet J Rare Dis. 2021;16(1):9.

29. Linden S, Klank S, Harms E, Gruneberg M, Park JH, Marquardt T. Cystinosis: Therapy adherence and metabolic monitoring in patients treated with immediate-release cysteamine. Mol Genet Metab Rep. 2020;24:100620.

30. Medic G, van der Weijden M, Karabis A, Hemels M. A systematic literature review of cysteamine bitartrate in the treatment of nephropathic cystinosis. Curr Med Res Opin. 2017;33(11):2065-76.

31. Mirdehghan M, Ahmadzadeh A, Bana-Behbahani M, Motlagh I, Chomali B. Infantile cystinosis. Indian Pediatr. 2003;40(1):21-4.

32. Muller M, Baumeier A, Ringelstein EB, Husstedt IW. Long-term tracking of neurological complications of encephalopathy and myopathy in a patient with nephropathic cystinosis: a case report and review of the literature. J Med Case Reports. 2008;2:235.

33. Proesmans W, Baten E, Hoogmartens J, Bruyneel P. Nephropathic cystinosis: effect of long-term cysteamine therapy. Clin Nephrol. 1987;27(6):309-12.

34. Rakheja D, Wooten DC, Gomez AM. Infantile cystinosis. Arch Pathol Lab Med. 2005;129(1):126-7.

35. Robert JJ, Tete MJ, Guest G, Gagnadoux MF, Niaudet P, Broyer M. Diabetes mellitus in patients with infantile cystinosis after renal transplantation. Pediatr Nephrol. 1999;13(6):524-9.

36. Scarvie KM, Ballantyne AO, Trauner DA. Visuomotor performance in children with infantile nephropathic cystinosis. Percept Mot Skills. 1996;82(1):67-75.

37. Theodoropoulos DS, Shawker TH, Heinrichs C, Gahl WA. Medullary nephrocalcinosis in nephropathic cystinosis. Pediatr Nephrol. 1995;9(4):412-8.

38. Trauner DA, Chase C, Scheller J, Katz B, Schneider JA. Neurologic and cognitive deficits in children with cystinosis. J Pediatr. 1988;112(6):912-4.

39. Trauner DA, Spilkin AM, Williams J, Babchuck L. Specific cognitive deficits in young children with cystinosis: evidence for an early effect of the cystinosin gene on neural function. J Pediatr. 2007;151(2):192-6.

40. Trauner DA, Williams J, Ballantyne AO, Spilkin AM, Crowhurst J, Hesselink J. Neurological impairment in nephropathic cystinosis: motor coordination deficits. Pediatr Nephrol. 2010;25(10):2061-6.

41. Van Stralen KJ, Emma F, Jager KJ, Verrina E, Schaefer F, Laube GF, et al. Improvement in the renal prognosis in nephropathic cystinosis. Clin J Am Soc Nephrol. 2011;6(10):2485-91.

42. Walton H, Hudson E, Simpson A, Ramsay AIG, Kai J, Morris S, et al. Defining Coordinated Care for People with Rare Conditions: A Scoping Review. Int J Integr Care. 2020;20(2):14.

43. Zeng B, Qiu S, Xiong X, Su X, Zhang Z, Wei Q, et al. The effect of different administrations of testosterone therapy on adverse prostate events: A Bayesian network meta-analysis. Front Endocrinol (Lausanne). 2022;13:1009900.

## **Ineligible language (n=4)**

1. Ariceta G, Camacho JA, Fernandez-Obispo M, Fernandez-Polo A, Gamez J, Garcia-Villoria J, et al. A coordinated transition model for patients with cystinosis: from pediatrics to adult care. Nefrologia. 2016;36(6):616-30.

2. Broyles HV, Dockery PW, Warner DB. Corneal cystinosis following eight years of systemic and topical treatment. J Fr Ophtalmol. 2023;17:17.

3. Guevara-Morales JM, Echeverri-Peña OY. Implementation of a method to quantify white blood cell cystine as a diagnostic support for cystinosis. Nefrologia. 2020;40(1):99-103.

4. Wang X, Zhang BL, Chen XY, Guo Z. Cystinosis induced by CTNS gene mutation: a rare disease study. Zhongguo Dang Dai Er Ke Za Zhi. 2021;23(12):1276-81.

## **Full text not retrievable (n=4)**

1. Barth J, Jaeger W. Benign cystinosis. Two siblings with cystine-crystals in cornea and conjunctiva. Berichte der Deutschen Ophthalmologischen Gesellschaft. 1981;Vol. 78:1061-5.

2. Calçada MBP. Quando a oftalmologia diagnostica uma patologia sistémica : a propósito de um caso clínico 2016.

3. MacLeod H. Rare World: Investigating Social Support in Rare Disease and Common Chronic Illness Communities 2018.

4. Sadek SA, El-Tantawy A, Nasr M. Infantile nephropathic cystinosis: Case series and review of literature. Kuwait Medical Journal. 2013;45(1):55-9.

# List of included studies but not considered due to staggered approach

**Includes – not considered for KQ4**

| **Reference** | **Study Design** |
| --- | --- |
| Klusmann M, Van't Hoff W, Monsell F, Offiah AC. Progressive destructive bone changes in patients with cystinosis. Skeletal Radiol. 2013;28:28. | case series |
| Topaloglu R, Gulhan B, Inozu M, Canpolat N, Yilmaz A, Noyan A, et al. The Clinical and Mutational Spectrum of Turkish Patients with Cystinosis. Clin J Am Soc Nephrol. 2017;12(10):1634-41. | non-comparative retrospective cohort study |
| Sikora P, Grenda R, Kowalczyk M, Kiec-Wilk B, Bienias B, Rubik J, et al. Nephropathic cystinosis in Poland: a 40-year retrospective study. Pol Arch Intern Med. 2022;132(11):25. | non-comparative retrospective cohort study |
| Kluck R, Muller S, Jagodzinski C, Hohenfellner K, Buscher A, Kemper MJ, et al. Body growth, upper arm fat area, and clinical parameters in children with nephropathic cystinosis compared with other pediatric chronic kidney disease entities. J Inherit Metab Dis. 2022;45(2):192-202. | non-comparative prospective cohort study |
| Vill K, Muller-Felber W, Landfarth T, Koppl C, Herzig N, Knerr C, et al. Neuromuscular conditions and the impact of cystine-depleting therapy in infantile nephropathic cystinosis: A cross-sectional analysis of 55 patients. J Inherit Metab Dis. 2022;45(2):183-91. | non-comparative cross-sectional study |
| Elkhateeb N, Selim R, Soliman NA, Atia FM, Abouelwoun, II, Elmonem MA, et al. Clinical and neurophysiological characterization of early neuromuscular involvement in children and adolescents with nephropathic cystinosis. Pediatr Nephrol. 2022;37(7):1555-66. | case series |
| Atmis B, A KB, Cevizli D, Kor D, Fidan HB, Bisgin A, et al. More than tubular dysfunction: cystinosis and kidney outcomes. Journal of Nephrology. 2022;35(3):831-40. | non-comparative retrospective cohort study |
| Polat E, Torun EG, Akkaya B. The Importance of the Early Diagnosis of Infantile Nephropathic Cystinosis: A case report. Klin Padiatr. 2021;233(2):79-82. | case report |
| Topaloglu R, Gultekingil A, Gulhan B, Ozaltin F, Demir H, Ciftci T, et al. Cystinosis beyond kidneys: gastrointestinal system and muscle involvement. BMC Gastroenterol. 2020;20(1):242. | case series |
| Ewert A, Leifheit-Nestler M, Hohenfellner K, Buscher A, Kemper MJ, Oh J, et al. Bone and Mineral Metabolism in Children with Nephropathic Cystinosis Compared with other CKD Entities. J Clin Endocrinol Metab. 2020;105(8):01. | Non-comparative cross-sectional study |
| Curie A, Touil N, Gaillard S, Galanaud D, Leboucq N, Deschenes G, et al. Neuropsychological and neuroanatomical phenotype in 17 patients with cystinosis. Orphanet J Rare Dis. 2020;15(1):59. | case series |
| Servais A, Saitovitch A, Hummel A, Boisgontier J, Scemla A, Sberro-Soussan R, et al. Central nervous system complications in adult cystinosis patients. J Inherit Metab Dis. 2020;43(2):348-56. | case series |
| Sousa-Neves F, Ribeiro AC, Saraiva E, Ribeiro L, Sequeira J, Varandas R. Ocular manifestations of intermediate cystinosis: To treat or not to treat? Eur J Ophthalmol. 2020;30(6):NP7-NP10. | case report |
| Bertholet-Thomas A, Claramunt-Taberner D, Gaillard S, Deschenes G, Sornay-Rendu E, Szulc P, et al. Teenagers and young adults with nephropathic cystinosis display significant bone disease and cortical impairment. Pediatr Nephrol. 2018;33(7):1165-72. | case series |
| Bertholet-Thomas A, Berthiller J, Tasic V, Kassai B, Otukesh H, Greco M, et al. Worldwide view of nephropathic cystinosis: results from a survey from 30 countries. BMC Nephrol. 2017;18(1):210. | Non-comparative cross-sectional study |
| Eroglu FK, Besbas N, Ozaltin F, Topaloglu R, Ozen S. Lupus in a patient with cystinosis: is it drug induced? Lupus. 2015;24(13):1452-4. | case report |
| Ahn MB, Kim SE, Cho WK, Jung MH, Suh BK. Endocrine complications during and after adolescence in a patient with cystinosis. Ann. 2016;21(3):174-8. | case report |
| El-Naggari MA, Elnour I, Al-Kindy H, Al-Shahrabally A, Abdelmogheth AA. Successful management of a neglected case of nephropathic cystinosis. Sultan Qaboos Univ Med J. 2014;14(2):e245-8. | case report |
| Besouw MT, Bowker R, Dutertre JP, Emma F, Gahl WA, Greco M, et al. Cysteamine toxicity in patients with cystinosis. J Pediatr. 2011;159(6):1004-11. | case series |
| Nakhaii S, Hooman N, Otukesh H. Gastrointestinal manifestations of nephropathic cystinosis in children. Iran J Kidney Dis. 2009;3(4):218-21. | non-comparative retrospective cohort study |
| Soliman NA, El-Baroudy R, Rizk A, Bazaraa H, Younan A. Nephropathic cystinosis in children: An overlooked disease. Saudi J Kidney Dis Transpl. 2009;20(3):436-42. | non-comparative prospective cohort study |
| Krischock L, Horsfield C, D'Cruz D, Rigden SP. Drug-induced lupus and antiphospholipid syndrome associated with cysteamine therapy. Nephrol Dial Transplant. 2009;24(6):1997-9. | case report |
| Ulmer FF, Landolt MA, Vinh RH, Huisman TA, Neuhaus TJ, Latal B, et al. Intellectual and motor performance, quality of life and psychosocial adjustment in children with cystinosis. Pediatr Nephrol. 2009;24(7):1371-8. | case series |
| Bendel-Stenzel MR, Steinke J, Dohil R, Kim Y. Intravenous delivery of cysteamine for the treatment of cystinosis: association with hepatotoxicity. Pediatr Nephrol. 2008;23(2):311-5. | case report |
| Ueda M, O'Brien K, Rosing DR, Ling A, Kleta R, McAreavey D, et al. Coronary artery and other vascular calcifications in patients with cystinosis after kidney transplantation. Clin J Am Soc Nephrol. 2006;1(3):555-62. | non-comparative cohort study |
| Geelen JM, Monnens LA, Levtchenko EN. Follow-up and treatment of adults with cystinosis in the Netherlands. Nephrol Dial Transplant. 2002;17(10):1766-70. | case series |
| Vester U, Schubert M, Offner G, Brodehl J. Distal myopathy in nephropathic cystinosis. Pediatr Nephrol. 2000;14(1):36-8. | case series |
| Wenner WJ, Murphy JL. The effects of cysteamine on the upper gastrointestinal tract of children with cystinosis. Pediatr Nephrol. 1997;11(5):600-3. | case series |
| Gahl WA, Charnas L, Markello TC, Bernardini I, Ishak KG, Dalakas MC. Parenchymal organ cystine depletion with long-term cysteamine therapy. Biochem Med Metab Biol. 1992;48(3):275-85. | non-comparative cross-sectional study |
| Sonies BC, Ekman EF, Andersson HC, Adamson MD, Kaler SG, Markello TC, et al. Swallowing dysfunction in nephropathic cystinosis. N Engl J Med. 1990;323(9):565-70. | non-comparative cross-sectional study |
| Gahl WA, Thoene JG, Schneider JA, O'Regan S, Kaiser-Kupfer MI, Kuwabara T. NIH conference. Cystinosis: progress in a prototypic disease. Ann Intern Med. 1988;109(7):557-69. | Redundant study data |
| Avner ED, Ellis D, Jaffe R. Veno-occlusive disease of the liver associated with cysteamine treatment of nephropathic cystinosis. J Pediatr. 1983;102(5):793-6. | case report |
| Yudkoff M, Foreman JW, Segal S. Effects of cysteamine therapy in nephropathic cystinosis. N Engl J Med. 1981;304(3):141-5. | case series |
| Corden BJ, Schulman JD, Schneider JA, Thoene JG. Adverse reactions to oral cysteamine use in nephropathic cystinosis. Dev Pharmacol Ther. 1981;3(1):25-30. | case series |
| Treikauskas U, Zerell K, Harms E, Aghayan-Ugurluoglu R, Brangenberg R, Herzig N, et al. Nephropathic cystinosis: Late complications of a multisystemic disorder - Results from the multidisciplinary cystinosis clinic Traunstein. Nieren- und Hochdruckkrankheiten. 2016;45(11):432-8. | non-comparative retrospective cohort study |
| Theodoropoulos DS, Krasnewich D, Kaiser-Kupfer MI, Gahl WA. Classic nephropathic cystinosis as an adult disease. Jama. 1993;270(18):2200-4. | case series |

**Includes – not considered for KQ 5**

| **Reference** | **Study Design** |
| --- | --- |
| Dohil R, Cabrera BL. Treatment of cystinosis with delayed-release cysteamine: 6-year follow-up. Pediatr Nephrol. 2013;28(3):507-10. | case series |
| Besouw M, Tangerman A, Cornelissen E, Rioux P, Levtchenko E. Halitosis in cystinosis patients after administration of immediate-release cysteamine bitartrate compared to delayed-release cysteamine bitartrate. Mol Genet Metab. 2012;107(1-2):234-6. | case series |
| Dohil R, Gangoiti JA, Cabrera BL, Fidler M, Schneider JA, Barshop BA. Long-term treatment of cystinosis in children with twice-daily cysteamine. J Pediatr. 2010;156(5):823-7. | case series |
| Dohil R, Fidler M, Gangoiti JA, Kaskel F, Schneider JA, Barshop BA. Twice-daily cysteamine bitartrate therapy for children with cystinosis. J Pediatr. 2010;156(1):71-5.e1-3. | case series |

**Includes – not considered for KQ 9**

| **Reference** | **Study Design** |
| --- | --- |
| Haycock GB, Al-Dahhan J, Mak RH, Chantler C. Effect of indomethacin on clinical progress and renal function in cystinosis. Arch Dis Child. 1982;57(12):934-9. | case series |

**Includes – not considered for KQ 15**

| **Reference** | **Study Design** |
| --- | --- |
| Bahillo-Curieses MP, Garrote-Molpeceres R, Minambres-Rodriguez M, Del Real-Llorente MR, Tobar-Mideros C, Rellan-Rodriguez S. Glycosuria and hyperglycemia in the neonatal period as the first clinical sign of Fanconi-Bickel syndrome. Pediatr Diabetes. 2018;19(1):180-3. | case report |
| Sahu KK, Law AD, Jain N, Khadwal A, Suri V, Malhotra P, et al. Fanconi Syndrome: A Rare Initial Presentation of Acute Lymphoblastic Leukemia. Indian Journal of Hematology & Blood Transfusion. 2016;32(Suppl 1):5-7. | case report |
| Shafqat H, Alquadan KF, Olszewski AJ. Severe hypocalcemia after denosumab in a patient with acquired Fanconi syndrome. Osteoporos Int. 2014;25(3):1187-90. | case report |
| Taylor HC, Elbadawy EH. Renal tubular acidosis type 2 with Fanconi's syndrome, osteomalacia, osteoporosis, and secondary hyperaldosteronism in an adult consequent to vitamin D and calcium deficiency: effect of vitamin D and calcium citrate therapy. Endocr Pract. 2006;12(5):559-67. | case report |

**Includes – not considered for KQ 16**

| **Reference** | **Study Design** |
| --- | --- |
| Kleta R, Bernardini I, Ueda M, Varade WS, Phornphutkul C, Krasnewich D, et al. Long-term follow-up of well-treated nephropathic cystinosis patients. J Pediatr. 2004;145(4):555-60. | case series |

**Includes – not considered for KQ 17***

** we only considered the most recent systematic reviews and guidelines with high methodological quality*

| **Reference** | **Study Design** |
| --- | --- |
| Bhasin S, Brito JP, Cunningham GR, Hayes FJ, Hodis HN, Matsumoto AM, et al. Testosterone Therapy in Men With Hypogonadism: An Endocrine Society Clinical Practice Guideline. J Clin Endocrinol Metab. 2018;103(5):1715-44. | guideline |
| Corona G, Goulis DG, Huhtaniemi I, Zitzmann M, Toppari J, Forti G, et al. European Academy of Andrology (EAA) guidelines on investigation, treatment and monitoring of functional hypogonadism in males: Endorsing organization: European Society of Endocrinology. Andrology. 2020;8(5):970-87. | guideline |
| Diem SJ, Greer NL, MacDonald R, McKenzie LG, Dahm P, Ercan-Fang N, et al. Efficacy and Safety of Testosterone Treatment in Men: An Evidence Report for a Clinical Practice Guideline by the American College of Physicians. Ann Intern Med. 2020;172(2):105-18. | guideline |
| Hackett G, Kirby M, Edwards D, Jones TH, Wylie K, Ossei-Gerning N, et al. British Society for Sexual Medicine Guidelines on Adult Testosterone Deficiency, With Statements for UK Practice. J Sex Med. 2017;14(12):1504-23. | guideline |
| Hackett G, Kirby M, Rees RW, Jones TH, Muneer A, Livingston M, et al. The British Society for Sexual Medicine Guidelines on Male Adult Testosterone Deficiency, with Statements for Practice. The World Journal of Mens Health. 2023;41(3):508-37. | guideline |
| Mulhall JP, Trost LW, Brannigan RE, Kurtz EG, Redmon JB, Chiles KA, et al. Evaluation and Management of Testosterone Deficiency: AUA Guideline. J Urol. 2018;200(2):423-32. | guideline |
| Elliott J, Kelly SE, Millar AC, Peterson J, Chen L, Johnston A, et al. Testosterone therapy in hypogonadal men: a systematic review and network meta-analysis. BMJ Open. 2017;7(11):e015284. | systematic review |
| Houghton DE, Alsawas M, Barrioneuvo P, Tello M, Farah W, Beuschel B, et al. Testosterone therapy and venous thromboembolism: A systematic review and meta-analysis. Thromb Res. 2018;172:94-103. | systematic review |
| Hudson J, Cruickshank M, Quinton R, Aucott L, Aceves-Martins M, Gillies K, et al. Adverse cardiovascular events and mortality in men during testosterone treatment: an individual patient and aggregate data meta-analysis. Lancet Healthy Longev. 2022;3(6):e381-e93. | systematic review |
| Kohn TP, Mata DA, Ramasamy R, Lipshultz LI. Effects of Testosterone Replacement Therapy on Lower Urinary Tract Symptoms: A Systematic Review and Meta-analysis. European Urology. 2016;69(6):1083-90. | systematic review |
| Lee JH, Shah PH, Uma D, Salvi DJ, Rabbani R, Hamid P. Testosterone Replacement Therapy in Hypogonadal Men and Myocardial Infarction Risk: Systematic Review & Meta-Analysis. Cureus. 2021;13(8):e17475. | systematic review |
| Loo SY, Chen BY, Yu OHY, Azoulay L, Renoux C. Testosterone replacement therapy and the risk of stroke in men: A systematic review. Maturitas. 2017;106:31-7. | systematic review |
| Nian Y, Ding M, Hu S, He H, Cheng S, Yi L, et al. Testosterone replacement therapy improves health-related quality of life for patients with late-onset hypogonadism: a meta-analysis of randomized controlled trials. Andrologia. 2017;49(4). | systematic review |
| Walther A, Breidenstein J, Miller R. Association of Testosterone Treatment With Alleviation of Depressive Symptoms in Men: A Systematic Review and Meta-analysis. JAMA Psychiatry. 2019;76(1):31-40. | systematic review |
| Miller JA, Nguyen TT, Loeb C, Khera M, Yafi FA. Oral testosterone therapy: past, present, and future. Sex Med Rev. 2023. | systematic review |

**Includes – not considered for KQ 20**

| **Reference** | **Study Design** |  |
| --- | --- | --- |
| Dohil R, Newbury RO, Sellers ZM, Deutsch R, Schneider JA. The evaluation and treatment of gastrointestinal disease in children with cystinosis receiving cysteamine. J Pediatr. 2003;143(2):224-30. | before-after study (same participants as in Dohil et al. 2005) | |

**Includes – not considered for KQ 25**

| **Reference** | **Study Design** |
| --- | --- |
| Anonymous. Canadian Agency for Drugs and Technologies in Health. 2019;07:07. | Systematic review (included in Liang et al. 2017) |
| Kowalczyk M, Toro MD, Rejdak R, Zaluska W, Gagliano C, Sikora P. Ophthalmic Evaluation of Diagnosed Cases of Eye Cystinosis: A Tertiary Care Center's Experience. Diagnostics (Basel). 2020;10(11):07. | case series |
| Bayram-Suverza M, Virgen-Batista MI, Vazquez-Lara Y. Importance of adherence to topical cysteamine in infantile ocular cystinosis: An illustrative case. Indian J Ophthalmol. 2022;70(7):2636-8. | case report |
| Kaur S, Sarma P, Kaur H, Prajapat M, Shekhar N, Bhattacharyya J, et al. Efficacy and Safety of Topical Cysteamine in Corneal Cystinosis: A Systematic Review and Meta-Analysis. Am J Ophthalmol. 2021;223:275-85. | Systematic review (low methodological quality) |
| Sousa-Neves F, Ribeiro AC, Saraiva E, Ribeiro L, Sequeira J, Varandas R. Ocular manifestations of intermediate cystinosis: To treat or not to treat? Eur J Ophthalmol. 2020;30(6):NP7-NP10. | case report |
| Helmi HA, El Mansoury J, Al Hazzaa S, Al Zoba A, Dirar QS. Asymmetrical Ocular Manifestations of Nephropathic Cystinosis; A case report. Am J Case Rep. 2019;20:1308-13. | case report |
| Florenzano P, Ferreira C, Nesterova G, Roberts MS, Tella SH, de Castro LF, et al. Skeletal Consequences of Nephropathic Cystinosis. J Bone Miner Res. 2018;33(10):1870-80. | case report |
| Peeters F, Cassiman C, Van Keer K, Levtchenko E, Veys K, Casteels I. Ophthalmic Outcome in a Belgian Cohort of Cystinosis Patients Treated with a Compounded Preparation of Cysteamine Eye Drops: Retrospective Analysis. Ophthalmol Ther. 2019;8(4):623-33. | case series |
| Biswas S, Sornalingam K. The Ocular Status of Cystinosis Patients Receiving a Hospital Pharmacy-Made Preparation of Cysteamine Eye Drops: A case series. Ophthalmol Ther. 2019;8(1):125-36. | case series |
| Tavares R, Coelho D, Macario MC, Torres A, Quadrado MJ, Murta J. Evaluation of treatment with cysteamine eyedrops for cystinosis with confocal microscopy. Cornea. 2009;28(8):938-40. | case report |
| Soliman NA, El-Baroudy R, Rizk A, Bazaraa H, Younan A. Nephropathic cystinosis in children: An overlooked disease. Saudi J Kidney Dis Transpl. 2009;20(3):436-42. | non-comparative prospective cohort study |
| Graf M, Grote A, Wagner F. [Cysteamine eyedrops for treatment of corneal cysteine deposits in infantile cystinosis]. Klin Monatsbl Augenheilkd. 1992;201(1):48-50. | case report |
| Liang H, Labbe A, Baudouin C, Plisson C, Giordano V. Long-term follow-up of cystinosis patients treated with 0.55% cysteamine hydrochloride. Br J Ophthalmol. 2021;105(5):608-13. | Non-comparative retrospective cohort study |

**Includes – not considered for KQ 27**

| **Reference** | **Study Design** |
| --- | --- |
| Peeters F, Cassiman C, Van Keer K, Levtchenko E, Veys K, Casteels I. Ophthalmic Outcome in a Belgian Cohort of Cystinosis Patients Treated with a Compounded Preparation of Cysteamine Eye Drops: Retrospective Analysis. Ophthalmol Ther. 2019;8(4):623-33. | case series |

**Includes – not considered for KQ 28**

| **Reference** | **Study Design** |
| --- | --- |
| Kowalczyk M, Toro MD, Rejdak R, Zaluska W, Gagliano C, Sikora P. Ophthalmic Evaluation of Diagnosed Cases of Eye Cystinosis: A Tertiary Care Center's Experience. Diagnostics (Basel). 2020;10(11):07. | case series |

**Includes – not considered for KQ 31**

| **Reference** | **Study Design** |
| --- | --- |
| Savage E, Beirne PV, Ni Chroinin M, Duff A, Fitzgerald T, Farrell D. Self-management education for cystic fibrosis. Cochrane Database Syst Rev. 2014(9):CD007641. | systematic review |

# Study characteristics of included studies

Table S32: Study characteristics of included primary studies

| **Author, Year,**  **Sponsor** | **Design,**  **Country,**  **Duration** | **KQ,**  **Risk of bias** | **N total (randomized),**  **Interventions,**  **N group** | **Population** | **Inclusion and exclusion criteria** | **Age, Age at diagnosis, Female, Ethnicity, Body mass index** | **eGFR, Kidney status, Cysteamine dose** | **Reported outcomes** |
| --- | --- | --- | --- | --- | --- | --- | --- | --- |
| Ahlenstiel-Grunow et al. 2017 [1]  Industry | Before-after study,  Germany,  Median follow-up: 14 months (range: 3-18) | KQ 5  high | N=12  G1: delayed release cysteamine (PROCYSBI®) taken every 12 hours  G2: immediate-release cysteamine treatment (CYSTAGON®) taken every 6 hours  *G1:* 12  *G2:* 12 | Cystinosis phenotype:  Nephropathic cystinosis | *Key inclusion criteria:*  Switch from IR to ER of cystinosis patients treated at Division of Pediatric Nephrology of Hannover Medical School  *Key exclusion criteria:*  NR | *Age in years:*  Median (range) 12 (1-18)  *Age at diagnosis:*  Median (range), months  10.5 (2-56)  *Female:*  59% (n=6)  *Ethnicity:*  NR  *Body mass index (kg/m²):*  NR | *eGFR (ml/min per 1.73 m²)*  Median (range) 67 (26-90)  *Kidney status:*  Overall:  3/12 (25%)  G1: NR  G2: NR  *Cysteamine dose:*  G1 median (range): 900 mg/m²/day (100-1200)  G2 median (range): 1200 mg/m²/day (200-1900) | Cystine level  Adverse events  Renal involvement |
| Ahmad et al. 2020 [2]  Internal funding | Retrospective cohort study,  Iraq,  NA | KQ 16  high | N=30  G1: start of cysteamine treatment 0-24 months  G2: start of cysteamine treatment >24 months  *G1:* 13  *G2:* 17 | Cystinosis phenotype:  Nephropathic cystinosis | *Key inclusion criteria:*  Cystinotic children less than 10 years, and diagnosed to have cystinosis according to clinical symptoms of disease and eye examination; demonstrating corneal cystine crystals.  *Key exclusion criteria:*  Patients that have other inborn error of metabolism.  Patients that have any endocrinopathy (thyroid disorder, congenital adrenal hyperplasia).  Patients that have other renal disorders (nephrotic syndrome, CKD, Fanconi syndrome/not related to cystinosis). | *Age in years:*  Mean ± SD, years  Overall: 5.4 ± 2.86  *Age at diagnosis:*  Mean ± SD, months  25.60 ± 20.88  *Female:*  43% (n=13)  *Ethnicity:*  NR  *Body mass index (kg/m²):*  Mean ± SD, kg/m²  Overall: 14.68 ± 2.36 | *eGFR (ml/min per 1.73 m²)*  Mean ± SD  Overall: 44.09 ± 37.64  *Chronic Kidney disease stage:*  Mean ± SD  Overall:  Serum Creatinine (µmol/L) 179.76 ± 177.53  Chitotriosidase(nmol/hour/ml) 308.30 ± 134.789  *Cysteamine dose:*  Mean, SD  Overall: 56.67 ± 7.581 mg/kg/day | Renal function |
| Al-Hemidan et al. 2017 [3]  NR | Before-after study,  Saudi Arabia,  2-8 years | KQ 25  high | N=32  G1: topical cysteamine 0.55% eye drops (every two hours while awake to both eyes)  *G1:*  32  (baseline)  *G2:*  32 | Cystinosis phenotype:  Nephropatic cystinosis | *Key inclusion criteria:*  Patients with diagnosed cystinosis  *Key exclusion criteria:* | *Age in years:*  *Median (range)* 8 (8 months - 19 years)  *Age at diagnosis:*  NR  *Female:*  56% (19/32)  *Ethnicity:*  NR  *Body mass index (kg/m²):*  NR | *eGFR (ml/min per 1.73 m²)*  NR  *Kidney status:*  NR  *Cysteamine dose:*  NR | Vision loss  Photophobia  Adherence |
| Blakey et al. 2019 [4]  NR | Case series,  UK,  NR | KQ 19  high | N=2 women (8 pregnancies)  G1*:* no cysteamine treatment  *NA* | Cystinosis phenotype:  Nephropatic cystinosis | *Key inclusion criteria:*  Pregnancy  *Key exclusion criteria:*  NA | *Age in years:*  26 and 33  *Age at diagnosis:*  NR  *Female:*  2/2 (100%)  *Ethnicity:*  NR  *Body mass index (kg/m²):*  NR | *eGFR (ml/min per 1.73 m²)*  74 and 40  *Kidney status:*  End-stage renal disease; both women with renal transplantation  *Cysteamine dose:*  Suspended after pregnancy test | Progression of cystinosis and CKD  Stillbirth  Pregnancy and birth complications  Child ICU admission  SGA |
| Bradbury et al. 1991 [5]  NR | RCT,  UK,  6 month | KQ 25  high | N=6 entered, 5 remained in the trial  G1: 0,2% cysteamine hydrochloride drops, 6 drops/daily  G2: placebo (sodium chloride 0.9%), 6 drops/daily  *G1:*  5  *G2:*  5 | Cystinosis phenotype:  Nephropathic cystinosis | *Key inclusion criteria:*  Patients with diagnosed cystinosis  *Key exclusion criteria:*  NR | *Age in years:*  8-16 years  *Age at diagnosis:*  1-2 years  *Female:*  2/5 (40%)  *Ethnicity:*  NR  *Body mass index (kg/m²):*  NR | *eGFR (ml/min per 1.73 m²)*  NR  *Kidney status:*  Kidney transplant: 80% (4/5)  *Cysteamine dose:*  NR | Vision loss  Photophobia  Adherence |
| Brodin-Sartorius et al. 2012 [6]  Non-governmental | Retrospective cohort study,  France,  Mean follow-up ± SD: 24.6 ± 7.0  G1: 21.9 ± 4.5  G2: 25.5 ± 7.3  G3: 27.2 ± 8.0 | KQ 4  high | N=86  G1: Cysteamine started <5 years  G2: Cysteamine started 5 years and more  G3: untreated before end-stage renal disease  *G1:*  40  *G2:*  8  *G3:*  38 | Cystinosis phenotype:  Nephropathic cystinosis | *Key inclusion criteria:*  Adult patients, at least 15 years old, with nephropathic cystinosis  *Key exclusion criteria:*  NR | *Age in years:*  Mean ± SD  overall: 26.7 ± 7.0  G1: 23.4 ± 4.4  G2: 28.0 ± 6.0  G3: 29.8 ± 8.0  *Age at diagnosis:*  Mean ± SD, years  overall: 2.2 ± 2.1  G1: 1.8 ± 1.8  G2: 2.5 ± 2.0  G3: 2.6 ± 2.5  *Female:*  Overall: 48.8% (n=42)  G1: 20/40 (50%)  G2: 3/8 (37.5%)  G3: 19/38 (59%)  *Ethnicity:*  NR  *Body mass index (kg/m²):*  NR | *eGFR (ml/min per 1.73 m²)*  NR  *Kidney status:*  ESRD  Overall: 78/86 (91%) at a mean age of 11.1 years (median 9.9; range 5.7–22.8).  Kidney transplantation:  Overall: 65/86 (76%)  CKD stage of those with naive kidneys (N=8):  1: n=5  3: n=1  4: n=2  *Cysteamine dose:*  NR | Survival  Renal involvement  Extra-renal involvement |
| Chan et al. 2022 [7]  NR | Case report,  Canada,  NR | KQ 19  Some concerns | N=1  G1: no cysteamine treatment  *NA* | Cystinosis phenotype:  Nephropatic cystinosis | *Key inclusion criteria:*  Pregnant women with cystinosis  *Key exclusion criteria:*  NA | *Age in years:*  24  *Age at diagnosis:*  Early in infacy due to older sibling with cystinosis  *Female:*  1/1 (100%)  *Ethnicity:*  NR  *Body mass index (kg/m²):*  NR | *eGFR (ml/min per 1.73 m²)*  89  *Kidney status:*  G2A3  *Cysteamine dose:*  Discontinued at confirmation of pregnancy, reinitiated immediately after delivery | Progression of cystinosis and CKD  Child development  Stillbirth  Pregnancy and birth complications  Child ICU admission  SGA |
| Clarke et al. 1995 [8]  NR | Before-after study,  USA,  46 months | KQ 15  high | N=11  G1: Supplementation with calcium (1-1.5g/day) , phosphate (250mg x4/day) and various types and dosages of vitamin D replacement  *G1:*  11 | Cystinosis phenotype: NA  Osteomalacia associated with adult Fanconi's syndrome | *Key inclusion criteria:*  Patients with osteomalacia associated with adult acquired Fanconi's syndrome  *Key exclusion criteria:*  (1) Evidence of inherited metabolic disorders, (2) Other known causes of osteomalacia including end-stage renal failure (one included patient had an initial creatinine clearance of 18 ml/min/ l 73 m^2^ and increased intact PTH level) or 25-hydroxyvitamin D deficiency syndromes (2 patients had low 1,25-dihydroxyvitamin D with normal 25-hydroxyvitamin D levels), and (3) Therapeutic use of medications, such as calcium, phosphate, vitamin D, anticonvulsants, aluminium-containing phosphate binders, bisphosphonates, or fluoride, which might affect vitamin D metabolism of bone mineralization in the year preceding diagnosis. | *Age in years:*  Mean (SEM; range):  54.2 (12.1; 33 to 69)  *Age at diagnosis:*  NR  *Female:*  4/11 (36%)  *Ethnicity:*  NR  *Body mass index (kg/m²):*  NR | *eGFR (ml/min per 1.73 m²)*  NR  *Kidney status:*  NR  *Cysteamine dose:*  NA | Rate & severity of rickets/osteomalacia Plasma Calcium |
| Dohil et al. 2005 [9]  Mixed | Before-after study,  USA,  16 weeks | KQ 20  high | N=12  G1: omeprazole: children <10 years of age received 10 mg twice daily (maximum of 20 mg twice daily) and children >=10 years received 20 mg twice daily (40 mg twice daily); mean (start) 1.3 mg/kg/day and (finish) 2.6 mg/kg/day  *G1:* 12 | Cystinosis phenotype:  Nephropatic cystinosis | *Key inclusion criteria:*  Children with cystinosis ≤ 18 years, on regular cysteamine bi-tartrate therapy,with GI symptoms  *Key exclusion criteria:*  NR | *Age in years:*  Mean (range): 5.8 (2.4-9.8 )  *Age at diagnosis:*  Mean (range), years  1.8 (0-5.5)  *Female:*  8/12 (67%)  *Ethnicity:*  NR  *Body mass index (kg/m²):*  NR | *eGFR (ml/min per 1.73 m²)*  Mean: 96.0  *Kidney status:*  NR (none with renal transplantation)  *Cysteamine dose:*  Mean (mg/kg/day): 51.8 | Heart burn  Rate of gastric ulcers  Nausea  GI perforation  Weight gain/Body Mass Index  Adverse events |
| Emma et al. 2021 [10]  Mixed | non-comparative retrospective cohort study  UK, France, Germany, Austria, Italy, Spain, Belgium, Netherlands, Turkey,  Minimum follow-up: 3 years | KQ 4, KQ9, KQ16  high | N=453  *KQ4:*  G1: oral cysteamine treatment  *G1:* 453  KQ9:  G1: "received": at least 5 years or 50% of observation period indomethacin  G2: no indomethacin  *G1:* 186  *G2:* 247 | Cystinosis phenotype:  Nephropathic cystinosis | *Key inclusion criteria:*  Nephropathic cystinosis  *Key exclusion criteria:*  NR | *Age in years:*  Median (range)  Overall: 15.3 (9.3-21.2)  *Age at diagnosis:**  CKD5: 405/453 (89.4%);  median (range), years: 1.6 (1.0-2.8)  ** Only patients that started treatment before reaching end-stage kidney disease*  *Female:*  Overall: 217/453 (47.9%)  *Ethnicity:*  NR  *Body mass index (kg/m²):*  NR | *eGFR (ml/min per 1.73 m²)*  NR  *Kidney status:*  Dialysis: 206/453 (45.5%)  Transplant: 147/329 (44.7%)  *Cysteamine dose:*  NR | *KQ4:*  Renal involvement  *KQ9:*  Disease progression  *KQ16:*  Renal function |
| Gahl et al. 2007 [11]  Governmental | Non-concurrent retrospective cohort study,  USA,  40 years, grouped in 10-year increments | KQ 4  high | N=100  G1: 10 years of continued oral cysteamine treatment  G2: 10 years without adequate cysteamine treatment  *G1:*  NR  *G2:*  NR | Cystinosis phenotype:  Nephropathic cystinosis | *Key inclusion criteria:*  Diagnosis with nephropathic cystinosis  *Key exclusion criteria:*  Intermediate or ocular cystinosis | *Age in years:*  Mean age in years ± SD  26.2 ± 6.5  *Age at diagnosis:*  NR  *Female:*  42/100 (42%)  *Ethnicity:*  NR  *Body mass index (kg/m²):*  NR | *eGFR (ml/min per 1.73 m²)*  NR  *Kidney status:*  Received a transplant: 92/100 (92%)  *Cysteamine dose:*  NR | Survival |
| Gahl et al. 1993 [12]  NR | Case series,  USA,  On average 5 years | KQ 21  Some concerns | N=23  G1: with carnitine supplementation (92 mg L-carnitine/kg/day given every 6 hours for an average of 62 months)  G2: without carnitine supplementation  *G1:*  6  *G2:*  17 | Cystinosis phenotype:  Nephropatic cystinosis | *Key inclusion criteria:*  NR  *Key exclusion criteria:*  NR | *Age in years:*  0-12  *Age at diagnosis:*  NR  *Female:*  NR  *Ethnicity:*  NR  *Body mass index (kg/m²):*  NR | *eGFR (ml/min per 1.73 m²)*  NR  *Kidney status:*  NR (no patient with kidney transplantation)  *Cysteamine dose:*  1.3 -1.95g of free base/m^2^/day divided every 6 hours | Muscle function  Adverse events |
| Gahl et al. 1987 [13]  Mixed | Non-concurrent prospective cohort study,  USA,  Up to 73 months | KQ 4  high | N=148  G1: oral cysteamine treatment  G2: historical control (placebo or ascorbic acid)  *G1:*  93  *G2:*  55 (28 placebo, 27 ascorbic acid) | Cystinosis phenotype:  Nephropathic cystinosis | *Key inclusion criteria:*  Nephropathic cystinosis  *Key exclusion criteria:*  NR | *Age in years:*  Age on final evaluation; mean ± SE  Overall: NR  G1: 6.79 ± 0.27  G2: 4.40 ± 0.28  *Age at diagnosis:*  NR  *Female:*  NR  *Ethnicity:*  NR  *Body mass index (kg/m²):*  NR | *eGFR (ml/min per 1.73 m²)*  NR  *Kidney status:*  NR  *Cysteamine dose:*  mean ± SE, mg/kg/d:  overall: NR  G1: 51.3 ± 7.2  G2: NA | Survival  Cystine level  Growth |
| Gaillard et al. 2021 [14]  Governmental | Prospective cohort study,  France,  12 months | KQ 5  high | N=17  G1: delayed release cysteamine treatment (PROCYSBI®) every 12 hours (25 mg and 75 mg capsule; dose adapted to cystine levels)  G2: immediate-release cysteamine treatment (CYSTAGON®) taken every 6 hours (50 mg and 150 mg capsules; dose adapted to cystine levels)  *G1:*  4  *G2:*  17 | Cystinosis phenotype:  Nephropathic cystinosis | *Key inclusion criteria:*  Children >4 years with confirmed nephropatic cystinosis, with oral cysteamine medication  *Key exclusion criteria:*  Pregnant women or women not accepting effective birth control if sexually active | *Age in years:*  Median (range) 13.9 (5.4-33)  *Age at diagnosis:*  Median (range), months  17 (3-76.9)  *Female:*  59% (n=10)  *Ethnicity:*  NR  *Body mass index (kg/m²):*  NR | *eGFR (ml/min per 1.73 m²)*  Median (range) 61.5 (7.9-132.2)  *Kidney status:*  Kidney status:  Transplanted: 10/17 (58.8%)  Under dialysis: 2/17 (11.8%)  *Cysteamine dose:*  G1: median (range): 1.06 g/m²/day (0.56-1.42)  G2: median (range): 1.29 g/m²/day (0.97-1.64) | Adherence |
| Greco et al. 2010 [15]  NR | Non-comparative retrospective cohort study  Italy,  Median follow-up in years (range): 17.6 (6.3-27.8) | KQ4, KQ9  high | N=23  *KQ4:*  G1: oral cysteamine treatment  *G1:*  23  *KQ9:*  G1: indomethacin treatment  G2: no indomethacin treatment  *G1:*  10  *G2:*  13 | Cystinosis phenotype:  Nephropathic cystinosis | *Key inclusion criteria:*  Diagnosis with nephropathic cystinosis  *Key exclusion criteria:*  NR | *Age in years:*  NR  *Age at diagnosis:*  Overall:  Median (range), months 21 (3 - 60)  *Female:*  Overall:  11/23 (48%)  *Ethnicity:*  NR  *Body mass index (kg/m²):*  NR | *eGFR (ml/min per 1.73 m²)*  eGFR>90 : 8/23 (34.8%)  *Kidney status:*  Abnormal renal function at diagnosis:  Overall: 15/23 (65%)  *Cysteamine dose:*  Dose (g/m^2^), mean ± SD  Overall: 1.35 ± 0.49  Dose (mg/Kg), mean ± SD  Overall: 53.5 ± 17.8 | *KQ4:*  Renal involvement  Growth  *KQ9:*  Growth  Disease progression |
| Haase et al. 2006 [16]  NR | Case report,  Germany,  4 years | KQ 19  Some concerns | N=1 (5 in case series, but only 1 with cystinosis)  G1: oral cysteamine treatment | Cystinosis phenotype:  Nephropatic cystinosis | *Key inclusion criteria:*  Pregnant women with cystinosis on dialysis  *Key exclusion criteria:*  NA | *Age in years:*  21  *Age at diagnosis:*  NR  *Female:*  1  *Ethnicity:*  NR  *Body mass index (kg/m²):*  NR (weight 56kg at admission) | *eGFR (ml/min per 1.73 m²)*  NR  *Kidney status:*  Patient on dialysis  *Cysteamine dose:*  Cystagon 3x300mg/day during pregnancy | Child development  Stillbirth  Pregnancy and birth complications  Child ICU admission  SGA |
| Hamed et al. 2022 [17]  Internal funding | Cross-sectional study,  Egypt,  NA | KQ 4  high | N=15  G1: oral ysteamine treatment (CYSTAGON®) (compliant), not defined  G2: oral cysteamine treatment (non-compliant)  *G1:*  5  *G2:*  10 | Cystinosis phenotype:  Nephropathic cystinosis | *Key inclusion criteria:*  Patients with confirmed diagnosis of cystinosis  *Key exclusion criteria:*  Patients with cardiac impairment secondary to chest affection | *Age in years:*  Mean ± SD, years  Overall: 8.53 ± -2.85  *Age at diagnosis:*  Mean ± SD, years  Overall: 3.68 ± 2.79  *Female:*  6/15 (40%)  *Ethnicity:*  NR  *Body mass index (kg/m²):*  NR | *eGFR (ml/min per 1.73 m²)*  NR  *Kidney status:*  Renal replacement therapy: 7/15 (47%):  Renal transplantation: 3/15 (20%)  Hemodialysis: 4/15 (26%)  CKD stage:  1a: 4/15 (26%)  3: 3/15 (20%)  4: 2/15 (13%)  5: 6/15 (40%)  *Cysteamine dose:*  NR | Renal involvement  Growth |
| Hohenfellner et al. 2022 [18]  Non-governmental | Case series,  Germany,  NA | KQ 6  Some concerns | N=6  G1: electrolytes supplementation  P1: no suppl  P2: no suppl  P3: no suppl  P4: no suppl  P5: suppl of potassium, bicarbonate and phosphate  P6: suppl of potassium, bicarbonate, citrate, phosphate, and calcium  *G1:*6 | Cystinosis phenotype:  Nephropathic cystinosis | *Key inclusion criteria:*  Children diagnosed and treated within 2 months after birth, follow-up data available  *Key exclusion criteria:*  NR | *Age in years:*  2 years 7 months to 18 years 7 months at last visit  *Age at diagnosis:*  2 months to 1 year 6 months  *Female:*  NR  *Ethnicity:*  NR  *Body mass index (kg/m²):*  NR | *eGFR (ml/min per 1.73 m²)*  Range: 79-136  *Kidney status:*  NR  *Cysteamine dose:*  Mean ± SD, range: 48 ± 16 to 71 ± 10 | Serum levels of electrolytes  Growth |
| Iwata et al. 1998 [19]  NR | RCT (before-after study for KQ25)  USA,  8-20 months | KQ 25, KQ27  Some concerns | N=14 (2 drop outs)  G1: cystamine eye drops + oral cysteamine treatment, taken every hour while awake  G2: cysteamine eye drops with 0.01% benzalkonium + oral cysteamine treatment, taken every hour while awake  *G1:*  14  *G2:*  14 | Cystinosis phenotype:  NR | *Key inclusion criteria:*  Patients with diagnosed cystinosis taking oral cysteamine who had never received topical cysteamine  *Key exclusion criteria:*  NR | *Age in years:*  Mean, years (range): 15 (3-29)  *Age at diagnosis:*  NR  *Female:*  57 % (8/14)  *Ethnicity:*  100% (14/14) Caucasian  *Body mass index (kg/m²):*  NR | *eGFR (ml/min per 1.73 m²)*  NR  *Kidney status:*  NR  *Cysteamine dose:*  NR | *KQ 25:*  Vision loss  Photophobia  Adherence  Adverse events  *KQ27:*  Adherence  Adverse events |
| Iyob-Tessema et al. 2021 [20]  NR | Cross-sectional study,  USA,  NA | KQ 5  Some concerns | N=76  G1: delayed-release cysteamine treatment (PROCYSBI®)  G2: immediate-release cysteamine treatment (CYSTAGON®)  *G1:*  50  *G2:*  25 | Cystinosis phenotype:  Nephropathic cystinosis | *Key inclusion criteria:*  All patients ≥6 years of age with a diagnosis of cystinosis were eligible  *Key exclusion criteria:*  NR | *Age in years:*  Mean (IQR)  Overall: 26.0 (15.0 to 32.0)  *Age at diagnosis:*  Median (IQR), months  17 (12 to 24)  *Female:*  Overall: 38/76 (50%)  *Ethnicity:*  NR  *Body mass index (kg/m²):*  NR | *eGFR (ml/min per 1.73 m²)*  Median (IQR)  55 (37–74)  *Kidney status:*  History of dialysis:  Overall: 30/76 (39%)  History of kidney transplantation:  Overall: 50/76 (66%)  CKD stage  Overall:  1: 4/76 (7%)  2: 27/76 (36%)  3A: 12/76 (21%)  3B: 13/76 (22%)  4: 8/76 (14%)  History of kidney transplantation  Overall: 50/76 (66%)  *Cysteamine dose:*  NR | Extra-renal involvement |
| Kaiser-Kupfer et al. 1990 [21]  NR | RCT,  USA,  4-24 months | KQ 25  high | N=29 entered the trial, 25 remained  G1: 0.1% or 0.5% cysteamine eye drops, one drop every hour awake  G2: Placebo, one drop every hour awake  *G1:*  25  *G2:*  25 | Cystinosis phenotype:  Nephropatic cystinosis | *Key inclusion criteria:*  Diagnosis with cystinosis  *Key exclusion criteria:*  NR | *Age in years:*  2 months - 31 years  *Age at diagnosis:*  NR  *Female:*  12/25 (48%)  *Ethnicity:*  NR  *Body mass index (kg/m²):*  NR | *eGFR (ml/min per 1.73 m²)*  NR  *Kidney status:*  NR  *Cysteamine dose:*  NR | Adherence  Adverse events |
| Kaiser-Kupfer et al. 1987 [22]  NR | RCT,  USA,  4-5 months | KQ 25  Some concerns | N=2  G1: 0.11% cysteamine eye drops, one drop every hour awake  G2: placebo (saline), one drop every hour awake  *G1:*  2  *G2:*  2 | Cystinosis phenotype:  Nephropatic cystinosis | *Key inclusion criteria:*  NR  *Key exclusion criteria:*  NR | *Age in years:*  13-14 months  *Age at diagnosis:*  NR  *Female:*  ½ (50%)  *Ethnicity:*  2/2 (100%) Caucasian  *Body mass index (kg/m²):*  NR | *eGFR (ml/min per 1.73 m²)*  NR  *Kidney status:*  NR  *Cysteamine dose:*  Oral cysteamine: 60-70mg per kilogram of body weight per day | Adherence  Adverse events |
| Kimonis et al. 1995 [23]  NR | Retrospective cohort study,  USA,  NR | KQ 16  high | N=101  G1: start of cysteamine therapy < 2 years of age  G2: start of cysteamine therapy between 2 to 5 years of age, or poorly compliant  G3: start of cysteamine therapy > 5 years of age  *G1:*  28  *G2:*  26  *G3:*  47 | Cystinosis phenotype:  nephropathic cystinosis | *Key inclusion criteria:*  Diagnosis with cystinosis  *Key exclusion criteria:*  NR | *Age in years:*  Mean age (range):  G1: 8.7 (0.6 to 16.7)  G2: 11.9. (4.5 to 19.0)  G3: 21.8 (9.0 to 36.2)  *Age at diagnosis:*  Mean (range)months  G1: 0.9 (NR)  G2: 1.8 (NR)  G3: 4.1 (NR)  *Female:*  NR  *Ethnicity:*  NR  *Body mass index (kg/m²):*  NR | *eGFR (ml/min per 1.73 m²)*  NR  *Chronic Kidney disease stage:*  Renal transplant:  G1: 1/28 (3.6%)  G2: 7/26 (26.9%)  G3: 43/47 (91.5%)  Mean age at transplant, years:  G1: 10.9  G2: 11.6  G3: 12.6  *Cysteamine dose:*  Pretransplant: 1.3 to 1.95 g/m² per day  Posttransplant: 50mg /kg per day | Growth |
| Kuczborska et al. 2019 [24]  NR | Case report,  Poland,  NR | KQ 19  Some concerns | N=1  G1: no cysteamine treatment  *NA* | Cystinosis phenotype:  Nephropatic cystinosis | *Key inclusion criteria:*  Pregant women with cystinosis  *Key exclusion criteria:*  NA | *Age in years:*  27  *Age at diagnosis:*  <12 months  *Female:*  1  *Ethnicity:*  NR  *Body mass index (kg/m²):*  NR | *eGFR (ml/min per 1.73 m²)*  NR  *Kidney status:*  ESRD at 10 years, 2 years haemodyalysis, 2 kidney transplantations (at the age of 12 and 19 years)  *Cysteamine dose:*  Irregular cysteamine tratement after 2nd renal transplantation (due to limited availability), then regular half-dose treatment due to intolerability of higher doses; stopped at confirmation of pregnancy | Progression of cystinosis and CKD  Stillbirth  Pregnancy and birth complications  Child ICU admission  SGA |
| Labbe et al. 2009 [25]  Non-governmental | Case series,  France,  NA (cross-sectional) | KQ 28  high | N=8 (16 eyes)  G1: AS-OCT  G2: slit lamp  G3: IVCM  *G1:*  8  AS-OCT  *G2:*  8  *G3:*  8 | Cystinosis phenotype:  Nephropathic Cystinosis | *Key inclusion criteria:*  Diagnosis with infantile nephropathic cystinosis  *Key exclusion criteria:*  NA | *Age in years:*  Range: 8-21  *Age at diagnosis:*  NR  *Female:*  6/8 (75%)  *Ethnicity:*  NR  *Body mass index (kg/m²):*  NR | *eGFR (ml/min per 1.73 m²)*  NR  *Kidney status:*  NR  *Cysteamine dose:*  NR | Cornea thinning |
| Labbe et al. 2014 [26]  Industry | Before-after study,  France,  4 years | KQ27  Some concerns | N=8 (16 eyes)  G1: cysteamine hydrochloride 0.55% eye drops (including benzalkonium chloride solution)  *G1:*  8 | Cystinosis phenotype:  Nephropathic cystinosis | *Key inclusion criteria:*  Patients diagnosed with nephropathic cystinosis, receiving, prior to enrolment, a fixed dose regimen of 0.1% cysteamine hydrochloride eye drop formulation  *Key exclusion criteria:*  NR | *Age in years:*  Mean ± SD  12.1 ± 4.6  *Age at diagnosis:*  Mean ± SD  17.5 ± 10.8 months  *Female:*  6/8 (75%)  *Ethnicity:*  NR  *Body mass index (kg/m²):*  NR | *eGFR (ml/min per 1.73 m²)*  NR  *Kidney status:*  NR  *Cysteamine dose:*  NR | Adherence  Adverse events |
| Langman et al. 2012 [27]  Mixed | Cross-over RCT,  US, European Union,  2 weeks run-in period  6 weeks (3 weeks each arm) | KQ 5  Some concerns | N=43  G1: delayed-release cysteamine treatment (PROCYSBI®), 25 mg and 75 mg, orally, taken every 12 hours  G2: immediate-release cysteamine treatment (CYSTAGON®), 50 mg and 150 mg, orally, taken every 6 hours  *G1:*  43  *G2:*  43 | Cystinosis phenotype:  Nephropathic cystinosis | *Key inclusion criteria:*  Children and adults with cystinosis able to swallow Cystagon capsules, taking a stable dose of Cystagon, with their own kidneys, with an eGFR >30 ml/min per 1.73 m² body surface area, continuing all current treatments  *Key exclusion criteria:*  NR | *Age in years:*  Mean ± SD  11.7 ± 4.2  *Age at diagnosis:*  NR  *Female:*  19/43 (44%)  *Ethnicity:*  NR  *Body mass index (kg/m²):*  mean ± SD  17.8 ± 2.8 | *eGFR (ml/min per 1.73 m²)*  Mean ± SD  86 ± 34  *Kidney status:*  NR  *Cysteamine dose:*  Daily cysteamine dose, mean ± SD  Per protocol (N=38); mg/d:  G1: 1513 ± 477  G2: 1801 ± 511 | Cystine level  Adverse events  Serious adverse events |
| Langman et al. 2014 [28]  Mixed | Before-after study,  US,  24 months | KQ 5  high | N=40  G1: delayed release cysteamine treatment (PROCYSBI®), taken every 12 hours  G2: immediate release cysteamine treatment (trade name: NR), taken every 6 hours  *G1:*  40  *G2:*  40 | Cystinosis phenotype:  Nephropathic cystinosis | *Key inclusion criteria:*  Patients with nephropathic cystinosis, with native kidneys and eGFR >30 mL/min/1.73 m^2^ (>0.5 mL/s/1.73 m2)  *Key exclusion criteria:*  NR | *Age in years:*  Mean ± SD  11.5 ± 3.6  *Age at diagnosis:*  NR  *Female:*  17/40 (43%)  *Ethnicity:*  NR  *Body mass index (kg/m²):*  mean ± SD  18.2 ± 3.1 | *eGFR (ml/min per 1.73 m²)*  Mean ± SD  63 ± 25  *Kidney status:*  NR  *Cysteamine dose:*  Baseline:  G1 (average % of previous IR dose ± SD): 83.7 ± 7.9  G2, mean ± SD: 1807 mg/day ±720  G1: mean ± SD dose reduction from 43.5 ± 10.8 mg/kg/day at baseline to 40.1 ± 13.1 mg/kg/day at follow-up mg/kg/day (p=0.05) | Quality of life  Cystine level  Adverse events  Serious adverse events  Renal involvement  Extra-renal involvement |
| Levtchenko et al. 2003 [29]  NR | Case series,  The Netherlands,  3 months | KQ 11  high | N=5  G1: enalapril therapy 0.15 mg/kg once daily  N*A* | Cystinosis phenotype:  Cystinosis with Fanconi syndrome | *Key inclusion criteria:*  NR  *Key exclusion criteria:*  NR | *Age in years:*  Range: 4-9 years  *Age at diagnosis:*  NR (age starting cysteamine therapy: median 14 months (range 7-22 months)  *Female:*  NR  *Ethnicity:*  NR  *Body mass index (kg/m²):*  NR | *eGFR (ml/min per 1.73 m²)*  NR  *Kidney status:*  NR  *Cysteamine dose:*  50-100 mg/kg (divided in 4-5 daily doses) | Adverse events  Albuminuria |
| Liang et al. 2017 [30]  Industry | Before-after study (RCT but only one arms used),  France,  90 days | KQ 25  high | N=32 entered, 31 remained in the trial. Only 15 (0.55% cysteamine) extracted  G1 (baseline): viscous cysteamine 3.8 mg/ml (equivalent to 0.55% cysteamine) solution (Cystadrops), one drop in each eye 4x/day  *G1:*  15 | Cystinosis phenotype:  Nephropatic cystinosis | *Key inclusion criteria:*  Two years of age or older, had a diagnosis of nephropathic cystinosis, and had corneal cystine crystal scores demonstrated by slit-lamp examination (no previous treatment)  *Key exclusion criteria:*  Less than 2 years of age or had an uncontrolled hepatic disorder, cardiovascular disease, neurologic disease, or cancer; hypersensitivity to cysteamine or any drop excipients; laboratory test results out of the normal range according to the reference laboratory values, unless they were considered clinically insignificant; or were pregnant, breast-feeding, or of child-bearing potential and not using an effective contraception method. | *Age in years:*  Mean ± SD  19.2 ± 15.5 (n=15)  *Age at diagnosis:*  NR  *Female:*  8/15 (53.3%)  *Ethnicity:*  NR  *Body mass index (kg/m²):*  NR | *eGFR (ml/min per 1.73 m²)*  NR  *Kidney status:*  NR  *Cysteamine dose:*  NR | Vision loss  Photophobia  Neovascularization of the cornea, need for cornea transplantation  Itchy eyes  Adherence  Adverse events |
| Liang et al. 2015 [31]  Non-governmental | Case series,  France,  NA (cross-sectional) | KQ 28  Some concerns | N=20 (40 eyes)  G1: AS-OCT  G2: slit lamp  G3: IVCM  G4: self-assessed photophobia (Grade 0 no  Photophobia to Grade 5 cannot bear sunlight even with sunglasses)  NA | Cystinosis phenotype:  Nephropathic cystinosis | *Key inclusion criteria:*  Patients with cystinosis: typical clinical history associated with an intraleukocyte cystine concentration above 3 nmol half-cystine/milligram  protein.  *Key exclusion criteria:*  NR | *Age in years:*  Mean ± SD  17.10 ± 9.55; range: 7-37)  *Age at diagnosis:*  NR  *Female:*  10/20 (50%)  *Ethnicity:*  Caucasian 100% (20/20)  *Body mass index (kg/m²):*  NR | *eGFR (ml/min per 1.73 m²)*  NR  *Kidney status:*  5/20 (25%) had renal transplantation  *Cysteamine dose:*  NR | Cornea thinning  Photophobia |
| MacDonald et al. 1990 [32]  NR | nRCT,  Canada,  7 months | KQ 25  high | N=4 (8 eyes)  G1: 0.3% cysteamine eye drops, 4 drops/daily  G2: placebo (saline), 4 drops/daily  *G1:*  4  *G2:*  4 | Cystinosis phenotype:  Nephropatic cystinosis | *Key inclusion criteria:*  Individuals affected by infantile nephropathic cystinosis  *Key exclusion criteria:*  *NR* | *Age in years:*  2-21 years  *Age at diagnosis:*  NR  *Female:*  ¾ (75%)  *Ethnicity:*  NR  *Body mass index (kg/m²):*  NR | *eGFR (ml/min per 1.73 m²)*  NR  *Kidney status:*  NR  *Cysteamine dose:*  NR | Vision loss |
| Markello et al. 1993 [33]  NR | Retrospective cohort study,  USA,  Mean follow-up 4.5 to 7.1 years | KQ 4  high | N=76  G1: adequate cysteamine treatment, beginning cysteamine treatment before 2 years of age, median leukocyte cystine levels ≤ 2nmol of 1/2 cystine per mg protein  G2: partial treatment cysteamine, beginning cysteamine treatment after 2 years of age, median leukocyte cystine levels > 2nmol of 1/2 cystine per mg protein  G3: no cysteamine  *G1:*  17  *G2:*  32  *G3:*  27 | Cystinosis phenotype:  Nephropathic cystinosis | *Key inclusion criteria:*  Children with diagnosed nephropathic cystinosis  *Key exclusion criteria:*  Children with only one assessment | *Age in years:*  Overall: NR  Mean ± SD  G1: 8.3 ± 3.8  G2: 10.5 ± 2.7  G3: 8.3 ± 1.9  *Age at diagnosis:*  Overall: NR  Mean ± SD, years  G1: 1.0 ± 0.5  G2: 2.4 ± 1.5  G3: 2.2 ± 2.1  *Female:*  NR  *Ethnicity:*  NR  *Body mass index (kg/m²):*  NR | *eGFR (ml/min per 1.73 m²)*  NR  *Kidney status:*  NR  *Cysteamine dose:*  before 1987: cysteamine hydrochloride: mean 50 to 60 mg of cysteamine free base/ kg of body weight  from 1987: cysteamine phosphocysteamine: 1.3 or 1.95g/m² body surface area/day | Renal involvement  Cystine level |
| Nesterova et al. 2015 [34]  NR | Retrospective cohort study,  USA,  NR | KQ 4  high | N=147  G1: cysteamine treatment, "Some cysteamine"  G2: no cysteamine treatment  *G1:*  53  *G2:*  94 | Cystinosis phenotype:  Nephropathic cystinosis | *Key inclusion criteria:*  Patients with nephropathic cystinosis  *Key exclusion criteria:*  Not transplanted or reaching renal failure; patients with a gap ≥3 years without recorded leucocyte cystine levels | *Age in years:*  Overall (years (range)):  28 (11-48)  G1: NR  G2: NR  *Age at diagnosis:*  NR  *Female:*  NR  *Ethnicity:*  NR  *Body mass index (kg/m²):*  NR | *eGFR (ml/min per 1.73 m²)*  NR  *Kidney status:*  NR  *Cysteamine dose:*  NR | Renal involvement  Cystine level |
| Nießl et al. 2022 [35]  Mixed | Retrospective cohort study,  Germany, Austria, Switzerland,  23 years of follow-up (longitudinal data from 1997-2020) | KQ 16  high | N=52  G1: oral cysteamine treatment start at ≥2 months of age  G2: oral cysteamine treatment start at <2 months of age  *G1:*  46  *G2:*  6 | Cystinosis phenotype:  Nephropathic cystinosis | *Key inclusion criteria:*  Patients with nephropathic cystinosis  *Key exclusion criteria:*  Data after dialysis or transplantation of kidney; measurements before or within 4 weeks of treatment start; data with missing cystine levels or eGFR | *Age in years:*  Age at last visit, mean ± SD years:  overall: 11.9 ± 6.37  treatment start at ≥2 months of age: 12.4 ± 6.22  treatment start at <2 monthsof age: 8.2 ± 6.87  *Age at diagnosis:*  Mean ± SD years:  overall: 1.45 ± 1.05  treatment start at ≥2 months of age: 1.63 ± 0.97  treatment start at <2 monthsof age: 0.04 ± 0.03  *Female:*  Overall: 23/52 (44.2%)  Treatment start at ≥2 months of age: 20/46 (43.5%)  treatment start at <2 monthsof age: 3/6 (50%)  *Ethnicity:*  NR  *Body mass index (kg/m²):*  NR | *eGFR (ml/min per 1.73 m²)*  Mean eGFRcr ± SD last visit:  Overall: 87.9 ± 35.0; N=52  *Chronic Kidney disease stage:*  NR  *Cysteamine dose:*  NR | Renal function |
| O'Connell et al. 2022 [36]  Industry | Retrospective cohort study,  Germany, Austria,  NR Data of patients from 1986-2020 (34 years), Median survey duration was 2 years (IQR: 1–5, range: 1-17) | KQ 16  high | N=74 (68 analysed)  G1: cystinosis diagnosed <18 months of age G2: cystinosis diagnosed >18 months of age  *G1:*  34  *G2:*  34 | Cystinosis phenotype:  Nephropathic cystinosis | *Key inclusion criteria:*  Infantile nephropathic cystinosis  *Key exclusion criteria:*  NR | *Age in years:*  Median (IQR; range): 11 (6-15; 0-42)  *Age at diagnosis:*  Median (IQR; range), months: 15 (10-29; 0-110)  *Female:*  35/74 (47.3%)  *Ethnicity:*  NR  *Body mass index (kg/m²):*  NR | *eGFR (ml/min per 1.73 m²)*  NR  *Chronic Kidney disease stage:*  NR  *Cysteamine dose:*  N=67; median (IQR; range) g/m^2^/d:  overall/G1: 1.26 (1.03–1.48; 0.22–1.99) | Renal function |
| Ozdemir et al. 2019 [37]  No funding | Case report,  Turkey,  1 year | KQ 28  high | N=1 (2 eyes)  G1: AS-OCT  G2: IVCM  *G1:*  1  *G2:*  1 | Cystinosis phenotype:  Non-nephropathic ocular cystinosis | *Key inclusion criteria:*  NR  *Key exclusion criteria:*  NR | *Age in years:*  36  *Age at diagnosis:*  36  *Female:*  1/1 (100%)  *Ethnicity:*  NR  *Body mass index (kg/m²):*  NR | *eGFR (ml/min per 1.73 m²)*  NR  *Kidney status:*  NR  *Cysteamine dose:*  Cysteamine drops (Cystadrops 0.55%) 4x1 drops/day for a year; no systemic cysteamine | Cornea thinning  Photophobia |
| Quinaux et al. 2021 [38]  Mixed | Cross-sectional study,  France,  NA | KQ 5  Some concerns | N=17  G1: delayed release cysteamine treatment (PROCYSBI®)  G2: short acting cysteamine treatment (CYSTAGON®)  *G1:*  10  *G2:*  7 | Cystinosis phenotype:  Nephropathic cystinosis | *Key inclusion criteria:*  Patients older than 2 years of age, confirmed diagnosis of nephropathic cystinosis, and ongoing oral cysteamine therapy at inclusion  *Key exclusion criteria:*  NR | *Age in years:*  Median (range)  G1: 12 (4-61)  G2: 15 (2-30)  *Age at diagnosis:*  G1: 0.9 to 6.5 years  G2: 0.1 to 4.0 years  *Female:*  G1: 3/10 (33%)  G2: 6/7 (86%)  *Ethnicity:*  NR  *Body mass index (kg/m²):*  NR | *eGFR (ml/min per 1.73 m²)*  Median (range)  G1: 65 (33–84)  G2: 46 (16–149)  p=n.s.  *Kidney status:*  Conservative kidney management: 12/17 (71%)  G1: 7/10 (70%)  G2: 5/7 (71%)  Kidney transplant: 5/17 (29%)  G1: 3/10 (30%)  G2: 2/7 (29%)  *Cysteamine dose:*  G1: 1012 (368 to 1902) (mg/m²)  G2: 1632 (1236 to 3607) (mg/m²)  p<0.05 | Cystine level  Extra-renal involvement |
| Ramappa et al. 2010 [39]  NR | Case report,  UK,  NR | KQ 19  Some concerns | N=1  G1: no cysteamine treatment  *NA* | Cystinosis phenotype:  Nephropatic cystinosis | *Key inclusion criteria:*  Pregnant women with cystinosis  *Key exclusion criteria:*  NA | *Age in years:*  NR  *Age at diagnosis:*  3 years  *Female:*  1/1 (100%)  *Ethnicity:*  Caucasian  *Body mass index (kg/m²):*  NR | *eGFR (ml/min per 1.73 m²)*  NR  *Kidney status:*  On haemodyalisis post unsuccessful renal transplant  *Cysteamine dose:*  Stopped at confirmation of pregnancy | Stillbirth  Pregnancy and birth complications  Child ICU admission  SGA |
| Reiss et al. 1988 [40]  Governmental | Case report,  USA,  NR | KQ 19  Some concerns | N=1  G1: no cysteamine treatment  *NA* | Cystinosis phenotype:  Nephropatic cystinosis | *Key inclusion criteria:*  Pregnant women with cystinosis  *Key exclusion criteria:*  NA | *Age in years:*  20  *Age at diagnosis:*  6 years  *Female:*  1/1 (100%)  *Ethnicity:*  NR  *Body mass index (kg/m²):*  25.2 | *eGFR (ml/min per 1.73 m²)*  NR  *Kidney status:*  Kidney transplantation at the age of 9y  *Cysteamine dose:*  None | Child development  Stillbirth  Pregnancy and birth complications  Child ICU admission  SGA |
| Robertson et al. 2022 [41]  NR | Case report,  South Africa,  NR | KQ 19  Some concerns | N=1  G1: oral cysteamine treatment until 18 weeks  *NA* | Cystinosis phenotype:  Nephropatic cystinosis | *Key inclusion criteria:*  Pregnancy  *Key exclusion criteria:*  NA | *Age in years:*  17  *Age at diagnosis:*  2 years  *Female:*  1/1 (100%9  *Ethnicity:*  African  *Body mass index (kg/m²):*  NR | *eGFR (ml/min per 1.73 m²)*  62  *Kidney status:*  NR  *Cysteamine dose:*  Discontinued at 18 weeks of pregnancy (when patient presented to pedriatric nephrology) | Stillbirth  Pregnancy and birth complications  Child ICU admission  SGA |
| Sadjadi et al. 2020 [42]  Non-governmental | Before after study,  USA,  13 months | KQ 22  high | N=20  G1: 5 weeks expiratory muscle strength training  *G1:*  20 | Cystinosis phenotype:  Nephropathic cystinosis | *Key inclusion criteria:*  NR  *Key exclusion criteria:*  NR | *Age in years:*  Median, range  29, 20-64)  *Age at diagnosis:*  NR  *Female:*  13/20 (65%)  *Ethnicity:*  NR  *Body mass index (kg/m²):*  NR | *eGFR (ml/min per 1.73 m²)*  NR  *Kidney status:*  18/20 (90%) with renal transplantation  *Cysteamine dose:*  NR (16/20, 60%, with oral cysteamine) | Muscle strength  Quality of life  Physical functioning  Respiratory function |
| Servais et al. 2022 [43]  Governmental | Case series,  France, UK, the Netherlands, Belgium, Spain, Italy,  NR | KQ 19  Some concerns | N=12 women (19 pregnancies)  G1: no cysteamine treatment  *NA* | Cystinosis phenotype:  Nephropatic cystinosis | *Key inclusion criteria:*  Infantile nephropatic cystinosis, confirmed pregnancies  *Key exclusion criteria:*  NR | *Age in years:*  Median (range): 28.5 (21-36)  *Age at diagnosis:*  Median (range), years  1.4 (0.5-4.0)  *Female:*  12  *Ethnicity:*  *Body mass index (kg/m²):*  NR (median height 150.5 cm, median weight 53.4kg) | *eGFR (ml/min per 1.73 m²)*  Median (range)  50 (23-111)  *Kidney status:*  CDK 4: 1/19 (5.3%); CDK 5: 18/19 (94.7%); 1/19 on haemodyalis, all others transplanted  *Cysteamine dose:*  Stopped before pregnancy: 7/19 (36.8%), on confirmation of pregnancy: 11/19 (57.9%), at 5 weeks: 1/19 (5.3%) | Progression of cystinosis and CKD  Child development  Stillbirth  Pregnancy and birth complications  Child ICU admission  SGA |
| Sonies et al. 2005 [44]  NR | Cross-sectional study,  USA,  NR | KQ 4  high | N=101  G1: variable years of cysteamine therapy  *G1:*  101 | Cystinosis phenotype:  Nephropathic cystinosis | *Key inclusion criteria:*  Patients with diagnosed nephropathic cystinosis  *Key exclusion criteria:*  Patients with ocular or non-nephropathic cystinosis, or with a cerebrovascular infarction causing swallowing difficulty | *Age in years:*  Mean (range)  28.2 (6-45)  *Age at diagnosis:*  NR  *Female:*  40/101 (39.6%)  *Ethnicity:*  NR  *Body mass index (kg/m²):*  NR | *eGFR (ml/min per 1.73 m²)*  NR  *Kidney status:*  Renal transplant: 80.2% (n=81)  *Cysteamine dose:*  NR  Mean ± SD  Years without cysteamine: 16.2 ± 11.4  Years with cysteamine: 6.7 ± 6.7 | Extra-renal involvement |
| Spicer et al. 2015 [45]  Internal funding | Retrospective non-concurrent cohort study,  Australia, New Zealand,  Mean follow-up: 12.8 ± 6.8 (range 0.9-24.2) years | KQ 4  high | N=36  G1: cysteamine treatment "late group 1995-2008"  G2: no cysteamine treatment "early group 1980-1994"  *G1:*  17    *G2:*  19 | Cystinosis phenotype:  Nephropathic cystinosis | *Key inclusion criteria:*  Patients with cystinosis receiving first kidney transplants  *Key exclusion criteria:*  NR | *Age in years:*  NR  *Age at diagnosis:*  NR  *Female:*  Overall N (%):  16/36 (44.4%)  G1: 5/17 (29.4%)  G2: 11/19 (57.9%)  *Ethnicity:*  White:  Overall: 34/36 (94.4%)  G1: 17/17 (100%)  G2: 17/19 (89.5%)  *Body mass index (kg/m²):*  NR | *eGFR (ml/min per 1.73 m²)*  eGFR at first renal replacement therapy (median (IQR)):  overall: 6.4 (5-7.9)  G1: 8.1 (6.5-11.9)  G2: 5.1 (4.9-6.8)  p=0.006  *Kidney status:*  1 kidney transplant:  Overall: 28/36 (77.8%)  G1: 14/17 (82.4%)  G2: 14/19 (73.7%)  2 kidney transplants:  overall: 8/36 (22.2%)  G1: 3/17 (17.6%)  G2: 5/19 (26.3%)  *Cysteamine dose:*  NR | Survival  Renal involvement |
| Tsilou et al. 2003 [46]  Mixed | Before-after study (RCT but before-after assessments of both arms used)  USA,  1 year | KQ25, KQ27  Some concerns | N= 15  G1: standard cysteamine formulation 0.55%,  cysteamine hydrochloride solution with benzalkonium chloride  0.01%  1 drop in each eye, every waking hour  G2: 0.55% cysteamine hydrochloride solution with  monosodium phosphate 1.85%, disodium EDTA 0.10%, and  benzalkonium chloride 0.01%  1 drop in each eye, every waking hour  *G1:*  15  *G2:*  15 | Cystinosis phenotype:  Nephropathic cystinosis | *Key inclusion criteria:*  Efficacy study: patients with cystinosis 2–12 years of age who had never received topical cysteamine and whose corneal cystine crystal score was >1.00  *Key exclusion criteria:*  history of non-compliance with eye drops or the follow up schedule | *Age in years:*  years, median (range): 6 (2–11)  *Age at diagnosis:*  NR  *Female:*  7/15 (53%)  *Ethnicity:*  Caucasian: 15/15 (100%)  *Body mass index (kg/m²):*  NR | *eGFR (ml/min per 1.73 m²)*  NR  *Kidney status:*  NR  *Cysteamine dose:*  NR | Itchy eyes  Adherence  Adverse events |
| Tsilou et al. 2006 [47]  NR | Case series,  USA,  28 years follow up | KQ 26  Some concerns | N=208  G1: oral cysteamine treatment, considered adequate if it lowered leucocyte cystine levels  below 2.5 nmol half-cystine/mg protein;  *NA* | Cystinosis phenotype:  Nephropathic cystinosis | *Key inclusion criteria:*  NR  *Key exclusion criteria:*  NR | *Age in years:*  Mean 17.3 ( SD 8.2), range 6 month - 42 years  *Age at diagnosis:*  NR  *Female:*  99/208 (48%)  *Ethnicity:*  NR  *Body mass index (kg/m²):*  NR | *eGFR (ml/min per 1.73 m²)*  NR  *Kidney status:*  NR  *Cysteamine dose:*  NR | Vision loss  Retinal pigment epithelium changes Visual field loss  Maculopathy  Neovascularization of the cornea |
| Vaisbich et al. 2010 [48]  NR | Non-concurrent cohort study,  Brazil,  9 years | KQ 4, KQ16  high | N=102  *KQ4:*  G1: oral cysteamine treatment  *G1:*  102  *KQ16:*  G2: starting cysteamine treatment under 2 years of age  G3: historical group without mercaptamine treatment until 1998; began receiving cysteamine treatment after 2 years of age  *G2:*  20  *G3:*  18 | Cystinosis phenotype:  Nephropathic cystinosis | *Key inclusion criteria:*  Diagnosis with nephropathic cystinosis  *Key exclusion criteria:*  NR | *Age in years:*  Mean ± SD, years:  G1: 11.5 ± 6.2  G2: NR  G3: NR  *Age at diagnosis:*  Mean ± SD, months:  G1: 55 ± 49  G2: NR  G3: NR  *Female:*  G1: 42/102 (41.2%)  G2: NR  G3: NR  *Ethnicity:*  NR  *Body mass index (kg/m²):*  NR | *eGFR (ml/min per 1.73 m²)*  NR  *Kidney status:*  CKD stage:  1: 23/102 (22.5%)  2-4: 19/102 (18.7%)  5: 60/102 (58.8%)  Dialysis: 26/102 (25.5%)  Kidney transplantation: 34/102 (33.3%)  *Cysteamine dose:*  60-90 mg/kg Cysteamine intake:  2000: 10/55 (18.2%)  2008: 81/102 (79.5%) | *KQ4:*  Extra-renal involvement Cystine level  *KQ16:*  Renal function  Growth |
| Vaisbich et al. 2022 [49]  Industry | Before-after study,  Brazil, USA,  0.5 to 21 months | KQ 5  high | N=15  G1: delayed-release cysteamine treatment (trade name: NR) administered twice daily, orally or via gastrostomy tube (G-tube) in patients with an established G-tube (n = 3), after a 2-h fast. The starting dose was one-quarter of the targeted maintenance dose based on age, weight, and body surface area (BSA). The dose was gradually escalated, in 10% increments every 2 weeks, based on monitoring of mixed leukocyte WBC cystine levels measured 30 min after the morning dose and collected bi-monthly (every 2 weeks), until the patient's WBC cystine level was <1 nmol ½ cystine/mg protein,  *G1:*  15 | Cystinosis phenotype:  Nephropathic cystinosis | *Key inclusion criteria:*  Confirmed, untreated, diagnosis of nephropathic cystinosis, no clinically significant change in liver function tests or renal function within 6 months prior to screening. Required hemoglobin level was >10 g/dl at screening  *Key exclusion criteria:*  History of active inflammatory bowel disease or prior resection of the small intestine, heart disease, or active bleeding disorder within 90 days prior to screening, malignant disease within 2 years prior to screening, kidney transplant, or using dialysis at time of trial. | *Age in years:*  Mean ± SD  2.2 ± 1.0 years (range of 1.0 to 4.5 years).  *Age at diagnosis:*  NR  *Female:*  7/15 (47%)  *Ethnicity:*  Non-White: 4/15 (27%)  *Body mass index (kg/m²):*  Mean ± SD Z score  -1.0 ± 1.1 | *eGFR (ml/min per 1.73 m²)*  eGFR:  Mean ± SD  Baseline: 55.93 ± 22.43 (n=15)  Study exit: 63.79 ± 21.44 (n=14)  Mean change of 8.14 ± 15.48  *Kidney status:*  NR  *Cysteamine dose:*  200 to 600 mg daily | Cystine level  Adverse events  Serious adverse events  Renal involvement |
| Van't Hoff et al. 1995 [50]  Mixed | Before-after study,  United Kingdom, Ireland,  4 months | KQ 4  high | N=59  G1: cysteamine or phosphocysteamine treatment  *G1:*  59 | Cystinosis phenotype:  Nephropathic cystinosis | *Key inclusion criteria:*  Patients with cystinosis  *Key exclusion criteria:*  NR | *Age in years:*  NR  *Age at diagnosis:*  Median (range), years  Overall: 1.6 (0.4-23)  *Female:*  30/59 (50.8%)  *Ethnicity:*  Caucasian: 45/59 (76%)  Asian: 12/59 (20%)  Middle Eastern: 1/59 (2%)  Asian/Caucasian: 1/59 (2%)  *Body mass index (kg/m²):*  NR | *eGFR (ml/min per 1.73 m²)*  NR  *Kidney status:*  Renal replacement therapy: 15/69 (25.4%)  Mean (range) age when renal replacement therapy was required, years: 8.6 (5.3-21.4)  *Cysteamine dose:*  mean dose  cysteamine: 33 mg /kg per day (n=14)  phosphocysteamine: 84 mg/kg per day (equivalent to 37 mg cysteamine base/kg per day) (n=24) | Cystine level  Growth |
| Van Stein et al. 2021 [51]  Mixed | Before-after study,  Germany,  1 day (several assessment points) | KQ 5  high | N=17  G1: delayed release cysteamine (PROCYSBI®), every 12 hours  G2: immediate-release cysteamine treatment (CYSTAGON®), taken every 6 hours  *G1:*  )  17  *G2:*  17 | Cystinosis phenotype:  Nephropathic cystinosis | *Key inclusion criteria:*  NR  *Key exclusion criteria:*  NR | *Age in years:*  Mean ± SD  22.8 ± 10.5  *Age at diagnosis:*  NR  *Female:*  4/17 (24%)  *Ethnicity:*  NR  *Body mass index (kg/m²):*  NR | *eGFR (ml/min per 1.73 m²)*  NR  *Kidney status:*  NR  *Cysteamine dose:*  according to age and body weight  mean ± SD  G1: single dose 741.2 ± 203.5; daily dose: 1482.4 mg/day  G2: single dose: 550.7 mg ± 159.6; daily dose: 2202.8 mg/day | Cystine level  Adverse events |
| Veys et al. 2023 [52]  Mixed | Retrospective cohort study,  Belgium, France, Germany, The Netherlands, Ireland, Poland, Spain, Turkey, UK,  Up to 20 years follow-up | KQ 16  high | N=52  G1: cysteamine treatment initiation < age 10 months (presymptomatic siblings)  G2: cysteamine treatment initiation ≥ age 10 months (symptomatic siblings)  *G1:*  16  *G2:*  36 | Cystinosis phenotype:  Nephropathic cystinosis | *Key inclusion criteria:*  Cystinosis diagnosis (index) and presymptomatic or symptomatic siblings (sibling)  *Key exclusion criteria:*  Twins with cystinosis; the youngest sibling if there was a triplet of cystinosis patients within the same family | *Age in years:*  Index: 23 ± 11 years  Sibling: 19 ± 11 years  *Age at diagnosis:*  Index: 22 (18; 29) months  Sibling: 6 (0.2; 14) months  *Female:*  NR  *Ethnicity:*  NR  *Body mass index (kg/m²):*  NR | *eGFR (ml/min per 1.73 m²)*  Index: 46 (25; 81)  Sibling: 73 (59; 93)  *Chronic Kidney disease stage:*  ESKD:  Index: 16/26 (61.5%)  Sibling: 10/26 (38.5%)  *Cysteamine dose:*  NR | Renal function |
| Viltz et al. 2013 [53]  Governmental | Cross-sectional study,  USA,  NR | KQ 16  high | N=46  G1: early cysteamine treatment (prior to or by the age of 2 years)  G2: later cysteamine treatment (after the age of 2 years)  *G1:*  32  *G2:*  14 | Cystinosis phenotype:  Nephropathic cystinosis | *Key inclusion criteria:*  Children and adolescents with diagnosis with nephropathic cystinosis  *Key exclusion criteria:*  Patients with other medical issues (ie, untreated thyroid dysfunction, uncorrected vision problems, and patients in renal failure), individuals on dialysis, acutely ill, or had any other condition that might adversely affect cognitive function | *Age in years:*  mean ± SD: 7.3 ± 4.5 years (NR for G1/G2)  *Age at diagnosis:*  NR  *Female:*  NR  *Ethnicity:*  NR  *Body mass index (kg/m²):*  NR | *eGFR (ml/min per 1.73 m²)*  NR  *Chronic Kidney disease stage:*  NR  *Cysteamine dose:*  NR | Intellectual function  Risk of visual spatial problems |

Abbreviations: AS-OCT, anterior segment optical coherence tomography; CKD, chronic kidney disease; EDTA, ethylenediaminetetraacetic acid; eGFR, estimated glomerular filtration rate; eGFRcr, estimated glomerular filtration rate from serum creatinine; ESRD, end-stage renal disease; GI, gastrointestinal; ICU, intensive care unit; IQR, interquartile range; IR, immediate-release; IVCM, in vivo confocal microscopy; KQ, key question; NA, not applicable; NR, not reported; N, number; RCT, randomized controlled trial; SD, standard deviation; SE, standard error; SEM, standard error of mean ; SGA, small gestational age;

**Guidelines/Systematic-Reviews**

Table S33: List of included guidelines or systematic reviews

| **Author, year,**  **sponsor** | **Design,**  **country** | **KQ,**  **risk of bias** | **Number of included participants, number of included studies,**  **intervention, control** | **Population** | **Inclusion and exclusion criteria** | **Age, female, ethnicity** | **Reported outcomes** |
| --- | --- | --- | --- | --- | --- | --- | --- |
| Dawson et al. 2023 [54]  Non-govermental | Systematic Review,  UK, | KQ 31  Low | N=1642  Included studies:  10  Intervention:  psychological intervention (self-management intervention comprising digital platform and behaviour change sessions; life coaching intervention; problem-solving intervention  Control:  treatment as usual (which usually consists of an annual review or routine reviews (or combination of both) with a specialist cystic fibrosis multidisciplinary team) | 1642 people with cystic fibrosis (or caregiver/parent-child dyads) | *Key inclusion criteria:*  participants of any age, gender, and ethnicity who had a diagnosis of cystic fibrosis and who were prescribed inhaled therapies participating in psychological interventions (patient education, medication reminders, rewards, psychological therapies) as well as the multidisciplinary team providing specialist care to the person with cystic fibrosis (e.g. training clinicians to communicate more effectively with people with cystic fibrosis), families of people with cystic fibrosis if they were the target participants of an intervention aimed at improving adherence to inhaled therapies in people with cystic fibrosis (e.g. increasing supervision with medication, family therapy);  *Key exclusion criteria:*  NR | *Age in years:*  Overall: NR  range: children 6-18 years to adults (no age range reported)  *Female:*  54.3%  *Ethnicity:*  NR | Adherence  Quality of life  Incidence or severity of psychosocial disorders |
| Martin et al. 2019 [55]  Non-govermental | Guideline,  UK, | KQ 31  Some concerns | N=NA  Included studies:  36  Intervention:  NR  Control:  NR | Children and adults of all ages, diagnosed with any of the four major types of epidermolysis bullosa: epidermolysis bullosa Simplex, Junctional epidermolysis bullosa, Dystrophic epidermolysis bullosa and Kindler Syndrome; family members of someone with epidermolysis bullosa; professionals caring for epidermolysis bullosa patients and their families | *Key inclusion criteria:*  Studies about psychosocial care of patients or caregivers/family of epidermolysis bullosa  *Key exclusion criteria:*  NR | *Age in years:*  NA  *Female:*  NA  *Ethnicity:*  NA | Quality of life  Psychosocial well-being |
| Ponce et al. 2018 [56]  Non-governmental | Systematic Review,  NR, | KQ 17  Low | N=1779  Included studies:  11 reports of 4 RCTs  Intervention:  transdermal testosterone (testosterone solution or gel or patch 1-2%; 5-60mg daily)  Control:  placebo | Hypogonadal Men | *Key inclusion criteria:*  RCTs investigating efficacy and adverse events of testosterone replacement therapy vs placebo in adult men with hypogonadism and morning total testosterone levels ≤ 300 ng/dL  *Key exclusion criteria:*  Trials in which patients received < 3 months of testosterone replacement therapy or placebo and randomized trials of testosterone formulations, such as selective androgen receptor modulators or an androgen other than testosterone; trials that included transgender individuals, patients with specific comorbidities (such as diabetes mellitus, human immunodeficiency virus-associated weight loss, or chronic obstructive lung disease), or men with drug-induced testosterone deficiency, such as that associated with the use of opioids or gonadotropinreleasing hormone agonists or antagonists; trials that used only surrogate measures or endpoints that were not deemed patient or clinically important (e.g.,lean body mass, inflammation markers) | *Age in years:*  Range: 48.4-72.3  *Female:*  0  *Ethnicity:*  NR | Mood (depression)  Libido  Erectile disfunction Adverse events |

Abbreviations: KQ, key question; NA, not applicable; N, number; RCT, randomized controlled trial

# Risk of bias of included studies

## **Risk of bias of RCTs, assessed with RoB 2.0**


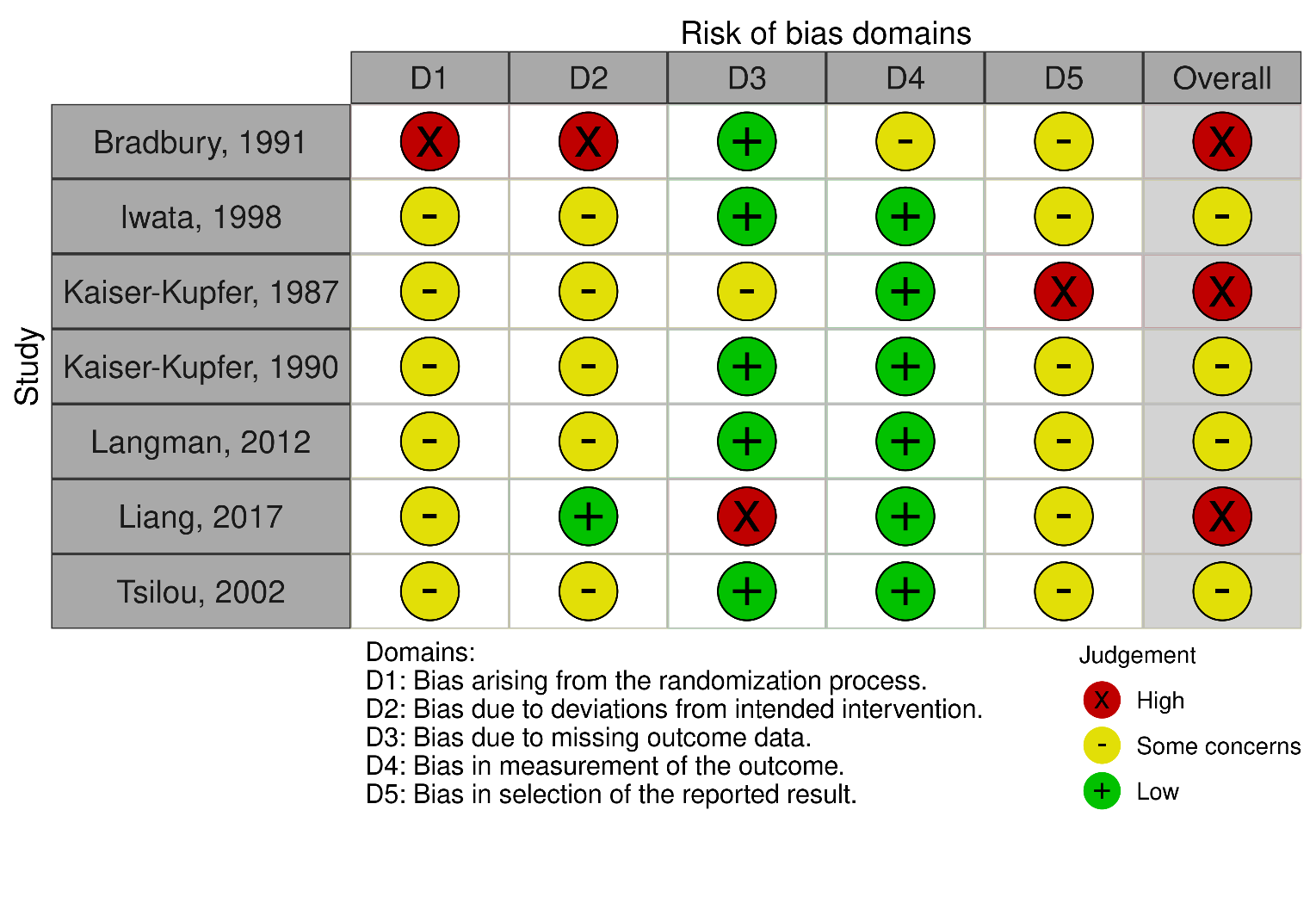


Figure S1: Risk of bias of RCTs, assessed with RoB 2.0

## **Risk of bias of non-randomized trials and cohort studies, assessed with ROBINS-I**


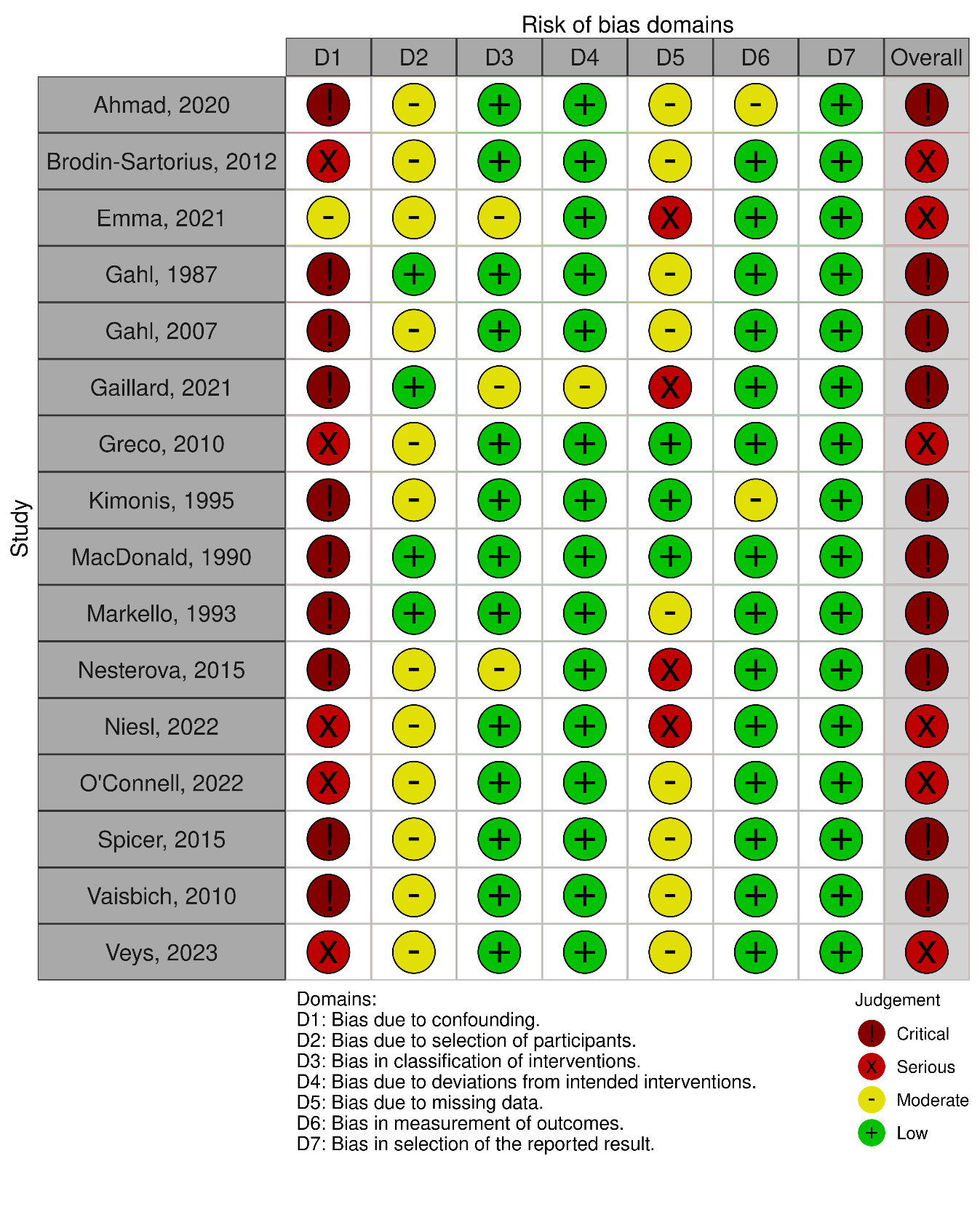


Figure S2: Risk of bias of non-randomized trials and cohort studies, assessed with ROBINS-I

## **Risk of bias of uncontrolled before-after studies, assessed with EPHHP**


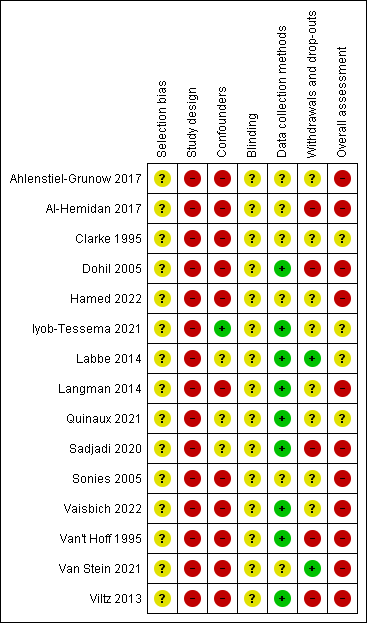


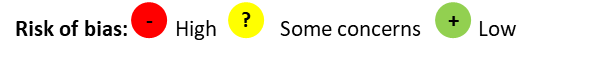
Figure S3: Risk of bias of uncontrolled before-after studies, assessed with EPHHP

## **Risk of bias of case series, assessed with the JBI checklist for case series**


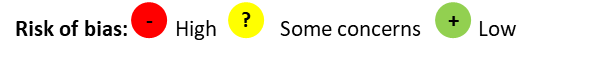

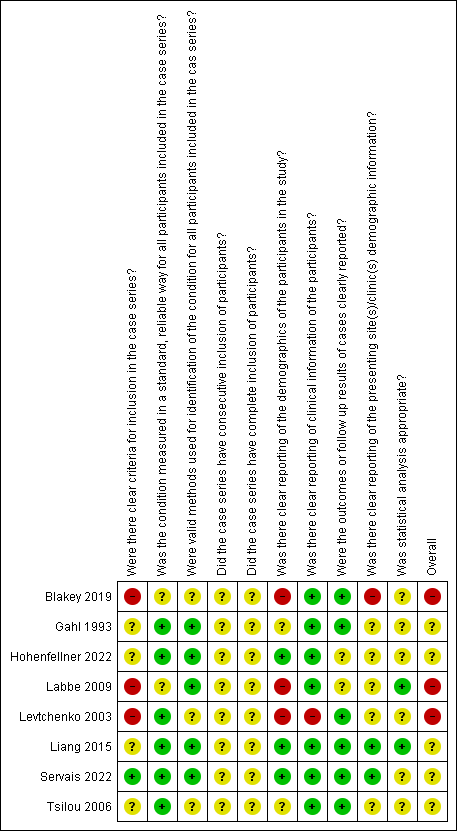


Figure S4: Risk of bias of case series, assessed with the JBI checklist for case series

## **Risk of bias of case reports, assessed with the JBI checklist for case reports**


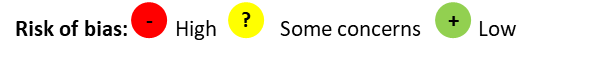

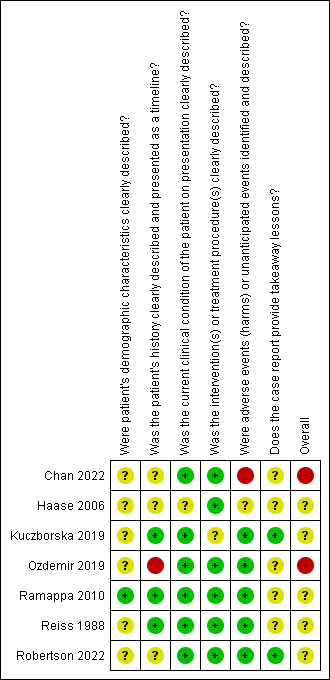


Figure S5: Risk of bias of case reports, assessed with the JBI checklist for case reports

## **Risk of bias of guidelines, assessed with the AGREE II tool**

Table S34: Risk of bias of guidelines

| Study | Domain 1: Scope and Purpose | Domain 2: Stakeholder Involvement | Domain 3: Rigour of Development | Domain 4: Clarity of presentation | Domain 5: Applicability | Domain 6: Editorial Independence | OVERALL GUIDELINE ASSESSMENT | I would recommend this guideline for use. |
| --- | --- | --- | --- | --- | --- | --- | --- | --- |
| Martin et al. 2019 [55] | 7 | 7 | 1 | 7 | 2 | 4 | 3 | yes |

AGREE scale 1-7; 1=strongly disagree; 7=strongly agree

## **Risk of bias of systematic reviews, assessed with the AMSTAR 2 tool**

| Study | DOMAIN 1: Study eligibility criteria | DOMAIN 2: Identification and selection of studies | DOMAIN 3: Data collection and study appraisal | DOMAIN 4: Synthesis and findings | Overall rating |
| --- | --- | --- | --- | --- | --- |
| Dawson et al. 2023 [54] | low | low | low | low | low |
| Ponce et al. 2018 [56] | low | low | low | low | low |

Table S35: Risk of bias of systematic reviews

# Summary of findings tables*

* tables for KQs with at least one outcome with evidence; KQs without evidence are not displayed in this chapter

Table S36: Summary of findings for KQ4

| **KQ4: What is the effectiveness and safety of systemic cysteamine therapy compared to no therapy/usual care in patients with confirmed cystinosis?** | | | | | | |
| --- | --- | --- | --- | --- | --- | --- |
| Outcome № of participants (studies) | Relative effect (95% CI) | **Anticipated absolute effects (95% CI)** | | | Certainty | What happens |
|  |  |  |  | **Difference** |  |  |
| Survival № of participants: 184 (3 observational studies) [6; 13; 45] | Patients treated with cysteamine had better survival during follow-up: 38/40 (95.0%) vs. without cysteamine: 4/11 (36.4%), p<0.001 for difference in one study (Brodin-Sartorius et al. 2012). Two other studies showed similar survival between treated and untreated patients: 15/17 (88.2%) vs. without cysteamine 16/19 (84.2%), p=0.9 (survival analysis, 10 years after kidney transplantation) (Spicer et al. 2015) and 92/93 (98.9%) vs. 51/55 (92.7%) (up to 73 months) (Gahl et al. 1987). | | | | ⨁◯◯◯ Very low^a,b^ | The evidence is very uncertain about the effect of systemic cysteamine therapy compared to no therapy/usual care on survival in patients with confirmed cystinosis. |
| Renal involvement  № of participants: 337 (4 observational studies) [6; 33; 34; 45] | Patients with cysteamine had better 10-year survival of first graft than those without cysteamine: Kaplan Meyer estimates: NR (86%) vs. NR (62%), p=0.09 (Spicer et al. 2015). They reached ESRD at older age than those without cysteamine (mean ± SEM/SD): 15.4±0.7 vs. 10.3±0.3, y=0.30x +8.82 (R2=0.61) and 13.4±4.8 vs. 9.5±2.0, p<0.001 (Nesterova et al. 2015; Brodin-Sartorius et al. 2012). The predicted age of renal failure was higher in those receiving treatment than those without, years: 74 (95%CI 41 to 243) vs. 10 (95%CI 8 to 12) (Markello et al. 1993).^c^ | | | | ⨁◯◯◯ Very low^a,d^ | The evidence is very uncertain about the effect of systemic cysteamine therapy compared to no therapy/usual care on renal involvement in patients with confirmed cystinosis. |
| Quality of life (general discomfort, pain) № of participants: (0 studies) | No evidence | | | | - |  |
| Extra-renal involvement  № of participants: 78 (1 observational study) [6] | Patients with cysteamine were on average (mean ± SD, years) older at diagnosis of hypothyroidism (13.5 ± 4.7 vs. 11.7 ± 5.9, p=0.010) and diabetes (17.6 ± 6.1 vs. 14.4 ± 5.3, p=n.s.) but younger at diagnosis of neuromuscular disorders than those without cysteamine (17.8 ± 5.2 vs. 21.9 ± 5.5, p=0.012) (Brodin-Sartorius et al. 2012).^e^ | | | | ⨁◯◯◯ Very low^f,g^ | The evidence is very uncertain about the effect of systemic cysteamine therapy compared to no therapy/usual care on extra-renal involvement in patients with confirmed cystinosis. |
| Cystine level (surrogate outcome) № of participants: 44 (1 observational study) [33] | Patients with cysteamine had lower levels of cystine than those without cysteamine (mean ± SD nmol/1/2 cystine/mg of protein): 1.1 ± 0.7 vs. 8.8 ± 5.5, p=0.001 (Markello et al. 1993).^h^ | | | | ⨁◯◯◯ Very low^i,j^ | The evidence is very uncertain about the effect of systemic cysteamine therapy compared to no therapy/usual care on cystine level (surrogate outcome) in patients with confirmed cystinosis. |
| Growth  № of participants: 148 (1 observational study) [13] | Cysteamine treated patients increased in height by +0.24 SD of the normal mean, whereas controls decreased on average by -0.59 SD of the normal mean. During the first year of treatment, the mean height increase (± SEM) was 73.5 ± 3.4 percent of the normal, as compared with 59.2 ± 3.7 in untreated patients (Gahl et a. 1987).^k^ | | | | ⨁◯◯◯ Very low^i^ | The evidence is very uncertain about the effect of systemic cysteamine therapy compared to no therapy/usual care on growth in patients with confirmed cystinosis. |
| Adverse events  № of participants: (0 studies) | No evidence | | | | - |  |
| ***The risk in the intervention group** (and its 95% confidence interval) is based on the assumed risk in the comparison group and the **relative effect** of the intervention (and its 95% CI). **Abbreviation:** CI**:** Confidence Interval; ESRD: End Stage Renal Disease, №: Number, NR: Not reported, n.s.: not significant, SD: Standard Deviation, SEM: Standard Error of the Mean, vs: versus | | | | | | |

Explanations

a. We used the Risk Of Bias In Non-randomized Studies – Of Interventions (ROBINS-I) to rate the risk of bias of the non-randomized studies. All studies have bias due to possible confounding, bias due to the participant selection, and bias due to missing data. We downgraded 3 steps because of additional risk of bias.

b. Few events; downgraded 1 step for imprecision.

c. A cross-sectional study by Hamed et al. 2022 [17] showed no difference in CKD stage 5 (1/5 (20%) vs. 5/10 (50%), p=0.170) and need of renal replacement therapy (3/5 (60%) vs. 5/10 (50%), p=0.221 between the cysteamine compliant and cysteamine non-compliant patients.

d. Large variation; downgraded 2 steps for imprecision.

e. A cross-sectional study showed a positive association between swallowing severity score (slope 0.04 units/years, p<0.0001) or oral muscle composite score (slope 0.04 units/years, p=0.0002) and years without oral cysteamine therapy, but the difference disappeared when age was used as covariate (Sonies et al. 2005) [44].

f. We use the Risk Of Bias In Non-randomized Studies - Of Interventions (ROBINS-I) to rate the risk of bias of the non-randomized studies. The study has bias due to possible confounding and bias due to missing data. We downgraded 2 steps. No further downgrading was considered necessary.

g. Small sample size; downgraded 2 steps for imprecision.

h. Results of three before-after studies showed higher levels of cystine before receiving cysteamine (mean ± SD nnmol/1/2 cystine/mg of protein): 2.95 ± 1.84 vs. 1.67 ± 0.99; 6.98 ± NR vs. 4.79 ± NR; 9.3 ± 1.0 vs. 1.7 ± 0.2; (Vaisbich et al. 2010 [48]; Van't Hoff et al. 1995 [50]; Gahl et al. 1987 [13])

i. We use the Risk Of Bias In Non-randomized Studies - Of Intervention (ROBINS-I) to rate the risk of bias of the non-randomized studies. The study has bias due to possible confounding and bias due to missing data. We downgraded 3 levels because of additional risk of bias.

j. Small sample size; downgraded 1 step for imprecision.

k. Results of a before-after study show no difference in Median Height Standard Deviation Score (HtSDS) pre-treatment: -3.26 and post-treatment: -3.25 (p>0.20, 95%CI -0.40 to +0.41) (Van't Hoff et al. 1995 [50]). A cross-sectional study found no significant relation between cysteamine compliance and weight or height affection: p=0.37 and 0.129 (no data) (Hamed et al. 2022 [17]).

Table S37: Summary of findings for KQ5

| **KQ5: What is the effectiveness and safety of delayed -release cysteamine therapy compared to immediate-release cysteamine therapy in patients with confirmed cystinosis?** | | | | | | |
| --- | --- | --- | --- | --- | --- | --- |
| Outcome № of participants (studies) | Relative effect (95% CI) | **Anticipated absolute effects (95% CI)** | | | Certainty | What happens |
|  |  |  |  | **Difference** |  |  |
| Treatment adherence № of participants: 21 (1 observational study) [14] | Patients with delayed-release cysteamine had a better daily treatment adherence score (median, range: 1.8 (0.1-2) vs. 0.5 (0.3 -1)), more percentage days at good adherence level (median % of days, range: 88 (1-99) vs. 2 (0-22)) and partial or good adherence level (median % of days, range: 91 (5-99) vs. 43 (27-78)) and more hours of daily exposition to treatment (median, range: 22.8 (6.1-23.9) vs. 14.9 (9.2-20.5)) than those with immediate-release cysteamine (Gaillard et al. 2021). | | | | ⨁◯◯◯ Very low^a,b^ | The evidence is very uncertain about the effect of delayed -release cysteamine therapy compared to immediate-release cysteamine therapy on treatment adherence in patients with confirmed cystinosis. |
| Quality of life (general discomfort, pain, family quality of life, night sleep) № of participants: 40 (1 observational study) [28] | Patients showed a change (intercept) in PedsQL as they switched from immediate-release cysteamine to delayed-release cysteamine in social function (11.23, p=0.049), school function (14.27, p=0.004), and total function (5.99, p=0.048) but not physical function (6.62, p=0.160) and emotional function (6.62, p=0.136). There were no changes (slope) in PedsQL from baseline to after 2 years of delayed-release cysteamine (total: 0.302, p=0.054; physical: 0.019, p=0.890; emotional: 0.492, p=0.201; social: 0.126, p=0.598; school: 0.184, p=0.072) (Langman et al. 2014). | | | | ⨁◯◯◯ Very low^b,c^ | The evidence is very uncertain about the effect of delayed -release cysteamine therapy compared to immediate-release cysteamine therapy on quality of life in patients with confirmed cystinosis. |
| Cystine level (surrogate outcome) № of participants: 43 (1 RCT) [27] | Cystine level in nmol 1/2 cystine/mg protein (least-squares mean ± SEM)  With delayed-release cysteamine: 0.70 ± 0.19  With immediate-release cysteamine: 0.97 ± 0.19  Mean difference: -0.27 ± 0.36 (95% CI -0.63 to 0.09), p<0.001  0.3 noninferiority margin defined a priori (Langman et al. 2012)^d^ | | | | ⨁⨁◯◯ Low^e^ | The evidence suggests that delayed-release cysteamine therapy results in little to no difference compared to immediate-release cysteamine therapy in cystine level (surrogate outcome) in patients with confirmed cystinosis. |
| Survival № of participants: (0 studies) | No evidence | | | | - |  |
| Adverse events  № of participants: 43 (1 RCT) [27] | Total adverse events:  With delayed-release cysteamine: 75/43  With immediate-release cysteamine: 26/43  (Langman et al. 2012)^f^ | | | | ⨁⨁◯◯ Low^e^ | The evidence suggests delayed-release cysteamine therapy results in a slight increase in adverse events compared to immediate-release cysteamine therapy in patients with confirmed cystinosis. |
| Renal involvement  № of participants: 67 (3 observational studies) [1; 28; 49] | The eGFR (ml/min per 1.73 m²) was similar before switching to delayed-release cysteamine and after in two studies (mean ± SD: 63 ± 25 vs. 57 ± 25, p=0.32 (Langman et al. 2014); median (range): 67 (26-90) vs. 67 (21-91), p=1 (Ahlenstiel-Grunow et al. 2017). In untreated patients, the eGFR (mean ± SD) increased from 55.93 ± 22.43 to 63.79 ± 21.44, p=NR after treatment with delayed-release cysteamine (Vaisbich et al. 2022). | | | | ⨁◯◯◯ Very low^c,g^ | The evidence is very uncertain about the effect of delayed-release cysteamine therapy compared to immediate-release cysteamine therapy on renal involvement in patients with confirmed cystinosis. |
| Extra-renal involvement  № of participants: 55 (2 observational studies) [28; 49] | Height z-score was similar before and after switching to delayed-release cysteamine (Mean ± SD): −1.15 ± −0.93 vs. −1.21 ± −0.96, p=0.46 (Langman et al. 2014). In untreated patients, height z-score (- 3.2 ± 1.6 vs. 0.1 ± 2.0, p=NR) and weight z-score (-4.0 ± 2.1 vs. -1.10 ± 1.8, p=NR) improved after treatment with delayed-release cysteamine. Body surface area z-score (-1.8 ± 1.1 vs. -1.3 ± 1.1, p=NR) and body mass index z-score (-1.0 ± 1.1 vs. -1.2 ± 1.3, p=NR) remained similar (Vaisbich et al. 2022).^h^ | | | | ⨁◯◯◯ Very low^c,g^ | The evidence is very uncertain about the effect of delayed-release cysteamine therapy compared to immediate-release cysteamine therapy on extra-renal involvement in patients with confirmed cystinosis. |
| ***The risk in the intervention group** (and its 95% confidence interval) is based on the assumed risk in the comparison group and the **relative effect** of the intervention (and its 95% CI). **Abbreviation:** CI**:** onfidence nterval; eGFR: estimated Glomerular Filtration Rate, MD**:** Mean Difference, №: Number, PedsQL: Pediatric Quality of Life, QoL: Quality of Life, RCT: Randomized Controlled Trial, NR: Not reported, SD: Standard Deviation, SEM: Standard Error of the Mean, SR: Systematic Review, vs: versus | | | | | | |

Explanations

a. We use the Risk Of Bias In Non-randomized Studies - Of Intervention (ROBINS-I) to rate the risk of bias of the non-randomized studies. The study has bias due to possible confounding, bias in classification of interventions, bias due to deviations from intended interventions, and bias due to missing data. We downgraded 3 steps because of additional risk of bias.

b. Very small sample size; downgraded 3 steps for imprecision.

c. We used the Effective Public Health Practice Project (EPHPP) tool to assess risk of bias in the before–after studies. GRADE starts with low certainty of evidence. We further downgraded 1 step for high risk of bias.

d. The results of 2 before-after studies showed no change in cystine levels (nmol ½ cystine/mg protein) before and after switching to delayed-release cysteamine (median, range): 1 (0.2–5.7) vs. 1 (0–2.5), p=0.64 (Ahlenstiel-Grunow et al. 2017) [4] or higher cystine levels (mean ± SD: 3.2 ± 3.0 vs. 0.8 ± 0.8) before treatment with delayed-release cysteamine in untreated populations (Vaisbich et al. 2022) [5].

e. Very small sample size; downgraded 2 steps for imprecision.

f. Patients showed similar proportions of adverse events before switching to delayed-release cysteamine and after in one study (5/11 (45.5%) vs. 7/12 (58.3%), p=NR) (Ahlenstiel-Grunow et al. 2017) [1]. One study showed higher proportions of GI side effects after ingestion of immediate-release cysteamine (15/17; 88.2%) compared to delayed-release cysteamine (6/17; 35.3%) (Van Stein et al. 2021) [51]. Another study found adverse events in all participants after treatment with delayed-release cysteamine, severe events were gastroenteritis (5/15 (33.3%)), dehydration (4/15 (26.7%)), vomiting (4/15 (26.7%)), electrolyte imbalance (2/15 (13.3%)), and gastrostomy (2/15 (13.3%)) (Vaisbich et al. 2022) [49].

g. Large variation and very small sample size; downgraded 3 steps for imprecision.

h. One cross-sectional study found no difference in mean grip strength between patients treated with delayed-release cysteamine and immediate-release cysteamine (mean z-score -0.38, 95%CI -0.88 to 0.11, p=0.13) (Iyob-Tessema et al. 2021) [20]. Another cross-sectional study reported similar proportions of any bone symptoms in both treatment groups (5/10 (50%) vs. 3/7 (43%), p=n.s.) (Quinaux et al. 2021) [38].

Table S38: Summary of findings for KQ6

| **KQ6: What is the effectiveness and safety of electrolytes vs no intervention on consequences of FANCONI-Syndrome in patients with cystinosis?** | | | | | | |
| --- | --- | --- | --- | --- | --- | --- |
| Outcome № of participants (studies) | Relative effect (95% CI) | **Anticipated absolute effects (95% CI)** | | | Certainty | What happens |
|  |  |  |  | **Difference** |  |  |
| Rickets (clinical and biochemical markers) № of participants: (0 studies) | No evidence | | | | - |  |
| Serum levels of electrolytes (surrogate) № of participants: 6 (1 observational study) [18] | 2 out of 6 patients received oral electrolyte supplementation. Serum electrolyte levels of all patients were within the normal range: potassium (mmol/l): 3.5 to 4.4; bicarbonate (mmol/l): 21 to 24.2; phosphate (mmol/l): 0.74 to 1.64; calcium (mmol/l): 2.26 to 2.46 (Hohenfellner et al. 2022). | | | | ⨁◯◯◯ Very low^a,b^ | The evidence is very uncertain about the effect of electrolytes compared to no intervention on serum levels of electrolytes (consequences of FANCONI-Syndrome) in patients with confirmed cystinosis. |
| Dehydration episodes № of participants: (0 studies) | No evidence | | | | - |  |
| Growth № of participants: 5 (1 observational study) [18] | 1 out of 5 patients received oral electrolyte supplementation. Anthropometric scores of all patients were within the normal range: height (percentile) range 27 to 90; weight (percentile) range 14 to 88; weight (kg) range 15.1 to 62.7 (Hohenfellner et al. 2022). | | | | ⨁◯◯◯ Very low^a,b^ | The evidence is very uncertain about the effect of electrolytes compared to no intervention on growth (consequences of FANCONI-Syndrome) in patients with confirmed cystinosis. |
| Quality of life № of participants: (0 studies) | No evidence | | | | - |  |
| Neurologic development and milestones № of participants: (0 studies) | No evidence | | | | - |  |
| Adverse events № of participants: (0 studies) | No evidence | | | | - |  |
| ***The risk in the intervention group** (and its 95% confidence interval) is based on the assumed risk in the comparison group and the **relative effect** of the intervention (and its 95% CI). **Abbreviations:** CI**:** Confidence Interval, №: Number | | | | | | |

Explanations

a. We used the JBI tool to assess risk of bias in the case series studies. GRADE starts with low certainty of evidence. We did not further downgrade for risk of bias.

b. Very small sample; downgraded 3 steps for imprecision.

Table S39: Summary of findings for KQ9

| **KQ9: What is the effectiveness and safety of indomethacin therapy vs no indomethacin therapy on consequences of Fanconi syndrome in patients with cystinosis?** | | | | | | |
| --- | --- | --- | --- | --- | --- | --- |
| Outcome № of participants (studies) | Relative effect (95% CI) | **Anticipated absolute effects (95% CI)** | | | Certainty | What happens |
|  |  |  |  | **Difference** |  |  |
| Dehydration episodes/polyuria № of participants: (0 studies) | No evidence | | | | - |  |
| Quality of life № of participants: (0 studies) | No evidence | | | | - |  |
| Serum levels of electrolytes № of participants: (0 studies) | No evidence | | | | - |  |
| Growth № of participants: 23 (1 observational study) [15] | Indomethacin use was not associated with the likelihood of having a HtSDS (height standard deviation scores) below –2 SD (HR=0.30, 95% CI 0.05 to 1.94, p=0.20) (Greco et al. 2010). | | | | ⨁◯◯◯ Very low^a,b^ | The evidence is very uncertain about the effect of indomethacin therapy compared to no indomethacin therapy on growth in patients with cystinosis and polyuria and hypokalemia. |
| Rickets (clinical and biochemical markers) № of participants: (0 studies) | No evidence | | | | - |  |
| Bicarbonate  № of participants: (0 studies) | No evidence | | | | - |  |
| Disease progression (deterioration of kidney function) № of participants: 383 (2 observational studies) [10; 15] | Indomethacin use was not associated with risk of stage III chronic renal failure (HR=1.10, 95% CI 0.35 to 3.36, p=0.87) (Greco et al. 2010) or risk of CKD stage 5 in children with cysteamine treatment before the age of 8 years (HR=0.95, 95% CI 0.68 to 1.34, p=0.78) (Emma et al. 2021). | | | | ⨁◯◯◯ Very low^c,d^ | The evidence is very uncertain about the effect of indomethacin therapy compared to no indomethacin therapy on disease progression (deterioration of kidney function) in patients with cystinosis and polyuria and hypokalemia. |
| Adverse events  № of participants: (0 studies) | No evidence | | | | - |  |
| ***The risk in the intervention group** (and its 95% confidence interval) is based on the assumed risk in the comparison group and the **relative effect** of the intervention (and its 95% CI). **Abbreviations:** CKD: Chronic Kidney Disease, CI**:** Confidence Interval, e.g.: for example, HR**:** Hazard Ratio, №: Number, SD: Standard Deviation | | | | | | |

Explanations

a. We used the Risk Of Bias In Non-randomized Studies – Of Interventions (ROBINS-I) to rate the risk of bias of the non-randomized studies. The study has bias due to possible confounding, and bias due to the participant selection. We downgraded 2 steps for risk of bias. No further downgrading was considered necessary.

b. Very small sample size and wide CI; downgraded 3 steps for imprecision.

c. We used the Risk Of Bias In Non-randomized Studies – Of Interventions (ROBINS-I) to rate the risk of bias of the non-randomized studies. The studies have bias due to possible confounding, bias due to the participant selection, bias in classification of interventions, and bias due to missing data. We downgraded 2 steps for risk of bias. No further downgrading was considered necessary.

d. CI includes appreciable benefit or harm; downgraded 1 step for imprecision.

Table S40: Summary of findings for KQ11

| **KQ11: What is the effectiveness and safety of RAAS blockade (Angiotensin Converting Enzyme [ACE]-inhibitor/Angiotensin receptor blockers [ARB]) in patients with cystinosis compared to no intervention in terms of renal function?** | | | | | | |
| --- | --- | --- | --- | --- | --- | --- |
| Outcome № of participants (studies) | Relative effect (95% CI) | **Anticipated absolute effects (95% CI)** | | | Certainty | What happens |
|  |  |  |  | **Difference** |  |  |
| Adverse events  № of participants: 5 (1 observational study) [29] | 2 out of 5 patients (40%) treated with the ACE inhibitor experienced hypotension (fatigue and dizziness) (Levtchenko et al. 2003). | | | | ⨁◯◯◯ Very low^a,b^ | The evidence is very uncertain about the effect of RAAS blockade (ACE inhibitor/ARB) compared to no intervention on adverse events in patients with cystinosis. |
| Estimated glomerular filtration rate (eGFR) № of participants: (0 studies) | No evidence | | | | - |  |
| Albuminuria № of participants: 5 (1 observational study) [29] | The mean reduction of albuminuria was 43% (range 4-72%). Albuminuria diminished in all 5 patients (Levtchenko et al 2003). | | | | ⨁◯◯◯ Very low^a,c^ | The evidence is very uncertain about the effect of RAAS blockade (ACE inhibitor/ARB) compared to no intervention on albuminuria in patients with cystinosis. |
| Quality of life № of participants: (0 studies) | No evidence | | | | - |  |
| ***The risk in the intervention group** (and its 95% confidence interval) is based on the assumed risk in the comparison group and the **relative effect** of the intervention (and its 95% CI). **Abbreviations:** ACE: Angiotensin Converting Enzyme, ARB: Angiotensin Receptor Blockers CI: Confidence Interval, eGFR: estimated Glomerular Filtration Rate, №: Number, RAAS: Renin-Angiotensin-Aldosteron-System | | | | | | |

Explanations

a. We used the JBI tool to assess risk of bias in the case series studies. GRADE starts with low certainty of evidence. We further downgraded 1 step for risk of bias.

b. Very few events and very small sample size; downgraded 3 steps for imprecision.

c. Very small sample size; downgraded 3 steps for imprecision.

Table S41: Summary of findings for KQ15

| **KQ15: What is the effectiveness and safety of calcium supplementation compared to no calcium in patients with Fanconi-syndrome on risk of hyperparathyroidism, and persistent rickets/osteomalacia?** | | | | | | |
| --- | --- | --- | --- | --- | --- | --- |
| Outcome № of participants (studies) | Relative effect (95% CI) | **Anticipated absolute effects (95% CI)** | | | Certainty | What happens |
|  |  |  |  | **Difference** |  |  |
| Rate of fractures № of participants: (0 studies) | No evidence | | | | - |  |
| Rate and severity of rickets/osteomalacia № of participants: (0 studies) | No evidence | | | | - |  |
| Plasma calcium № of participants: 11 (1 observational study) [8] | Mean calcium serum level before and after treatment with calcium, phosphate and various types and dosages of vitamin D replacement (nmol/l, SEM): 2.33 (0.04) vs. 2.20 (0.03) (Clarke et al. 1995). | | | | ⨁◯◯◯ Very low^a,b,c^ | The evidence is very uncertain about the effect of calcium supplementation compared to no calcium on plasma calcium in patients with Fanconi-Syndrome. |
| Radiologic evidence of active rickets № of participants: (0 studies) | No evidence | | | | - |  |
| Bone deformity № of participants: (0 studies) | No evidence | | | | - |  |
| Growth impairment № of participants: (0 studies) | No evidence | | | | - |  |
| Quality of life № of participants: (0 studies) | No evidence | | | | - |  |
| Adverse events № of participants: (0 studies) | No evidence | | | | - |  |
| ***The risk in the intervention group** (and its 95% confidence interval) is based on the assumed risk in the comparison group and the **relative effect** of the intervention (and its 95% CI). **Abbreviations:** CI: Confidence Interval, №: Number, SEM: Standard Error of the Mean, vs: versus | | | | | | |

Explanations

a. Before-after studies are inherently at higher risk of bias than designs with comparators and using concurrent controls. We downgraded 2 steps.

b. The intervention included phosphate and Vitamin in addition to Calcium; downgraded 2 steps for indirectness.

c. Very small sample size; downgraded 3 steps for imprecision.

Table S42: Summary of findings for KQ16

| **KQ16: Does early cysteamine treatment compared to late cysteamine treatment affect growth impairment and risk of neurological symptoms in infants, children, and adolescents with cystinosis?** | | | | | | |
| --- | --- | --- | --- | --- | --- | --- |
| Outcome № of participants (studies) | Relative effect (95% CI) | **Anticipated absolute effects (95% CI)** | | | Certainty | What happens |
|  |  |  |  | **Difference** |  |  |
| Renal function (CKD stage) № of participants:755^§^ (7 observational studies) [2; 6; 10; 35; 36; 48; 52] | Patients diagnosed >18 months of age had a better median renal survival than patients <18 months of age: 21 years (95% CI 16 to NR) vs. 13 years (95% CI 10 to NR), p=0.033 (O´Connell et al. 2022). Age at start of treatment was associated with risk of CKD Stage 5 (HR=1.24, 95% CI 1.09 to 1.42) (Emma et al. 2021), and the proportion of patients with CKD stage 5 was lower among patients who had started treatment earlier: 1/20 (5.0%) vs. 9/18 (50.0%) (Vaisbich et al. 2010). Other studies showed lower proportions of ESRD in patients who had started treatment earlier: 32/40 (80%) vs. 8/8 (100%) (Brodin-Sartorius et al. 2012) and 0/16 (0%) vs. 18/36 (50%), p=0.002 (Veys et al. 2023). The proportion of patients requiring kidney transplantation was lower among those who started treatment earlier: 1/20 (5.0%) vs. 4/18 (22.2%) (Vaisbich et al. 2010) and 0/6 (0%) vs. 11/46 (23.9%) (Niesl et al. 2022). eGFR (ml/min/1.73 m^2^) was higher in patients who had started treatment earlier: mean rank 23.54 vs. 9.35, p=0.0001 (Ahmad et al. 2020) and mean (SD) 123 (32.0) vs. 88.8 (35.0) (Niesl et al. 2022). | | | | ⨁⨁◯◯ Low^a^ | The evidence suggests early cysteamine treatment results in an increase in renal function compared to late cysteamine treatment in infants, children, adolescents with cystinosis. |
| Quality of life № of participants: (0 studies) | No evidence | | | | - |  |
| Growth № of participants: 139 (2 observational studies) [23; 48] | Patients who started treatment early had better mean height z scores ± SEM/SD than those receiving treatment at a later age: -2.17 ± 0.39 vs. -4.07 ± 0.39, p=0.001 (age-adjusted) (Kimonis et al. 1995) and -3.64 ± 1.92 vs. -4.16 ±1.37 (Vaisbich et al. 2010). | | | | ⨁◯◯◯ Very low^b,c^ | The evidence is very uncertain about the effect of early cysteamine treatment compared to late cysteamine treatment on growth in infants, children, adolescents with cystinosis. |
| Adverse events  № of participants: (0 studies) | No evidence | | | | - |  |
| Intellectual function assessed with: Wechsler Intelligence Scale № of participants: 46 (1 observational study) [53] | Patients who were treated early showed no difference in intellectual function (full scale IQ) to patients treated later (mean ± SD): 94.0 ± 12.5 vs. 83.0 ± 15.1, p=0.13 (Viltz et al. 2013). | | | | ⨁◯◯◯ Very low^d,e^ | The evidence is very uncertain about the effect of early cysteamine treatment compared to late cysteamine treatment on intellectual function in infants, children, adolescents with cystinosis. |
| Risk of visual spatial problems assessed with: Woodcock-Johnson Psychoeducational Battery № of participants: 46 (1 observational study) [53] | Patients who were treated early scored higher on a test of visual spatial skills than patients treated later (mean ± SD): 104.5 ± 12.0 vs. 92.7 ± 22.6, p= 0.038 (Viltz et al. 2013). | | | | ⨁◯◯◯ Very low^d,e^ | The evidence is very uncertain about the effect of early cysteamine treatment compared to late cysteamine treatment on risk of visual spatial problems in infants, children, adolescents with cystinosis. |
| Motor coordination № of participants: (0 studies) | No evidence | | | | - |  |
| * **The risk in the intervention group** (and its 95% confidence interval) is based on the assumed risk in the comparison group and the **relative effect** of the intervention (and its 95% CI).  § There is probably an overlap of patient data from the different studies, although the exact extent of overlap is unknown as no individual patient data were available in the cited studies. **Abbreviations:** CI**:** Confidence Interval, CKD: Chronic Kidney Disease, eGFR: estimated Glomerular Filtration Rate, ESRD: End Stage Renal Disease, HR: Hazard Ratio, IQ: intelligence Quotient, № = Number, NR: Not reported, SD: Standard Deviation, SEM: Standard Error of the Mean, vs: versus | | | | | | |

Explanations

a. We used the Risk Of Bias In Non-randomized Studies – Of Interventions (ROBINS-I) to rate the risk of bias of the non-randomized studies. All studies have bias due to possible confounding, bias due to the participant selection, and bias due to missing data. We downgraded 2 steps. No further downgrading was considered necessary.

b. We used the Risk Of Bias In Non-randomized Studies – Of Interventions (ROBINS-I) to rate the risk of bias of the non-randomized studies. The studies have bias due to possible confounding, bias due to the participant selection, and bias due to missing data. We downgraded 3 steps because of additional risk of bias.

c. Large variation; downgraded 1 step for imprecision.

d. We used the Effective Public Health Practice Project (EPHPP) tool to assess risk of bias in the before–after studies. GRADE starts with low certainty of evidence. We further downgraded 1 step for high risk of bias.

e. Very small sample size; downgraded 2 steps for imprecision.

Table S43: Summary of findings for KQ17

| **KQ17: What is the effectiveness and safety of testosterone replacement therapy compared to no testosterone replacement therapy on health outcomes in adolescents1383 and adult males with endocrine deficits (i.e. hypogonadism) and low testosterone levels?** | | | | | | |
| --- | --- | --- | --- | --- | --- | --- |
| Outcome № of participants (studies) | Relative effect (95% CI) | **Anticipated absolute effects (95% CI)** | | | Certainty | What happens |
|  |  |  |  | **Difference** |  |  |
| Mood (depression) № of participants: 1179 (2 RCTs included in this SR) [56] | - | - | - | SMD 0.08 SD higher (0.03 lower to 0.2 higher) | ⨁⨁⨁◯ Moderate^a,b^ | Testosterone replacement therapy probably results in a slight improvement of mood compared to no testosterone replacement therapy in adolescents and adult males with endocrine deficits and low testosterone levels. |
| Well-being № of participants: (0 studies) | No evidence | | | | - |  |
| Muscle strength № of participants: (0 studies) | No evidence | | | | - |  |
| Quality of life № of participants: (0 studies) | No evidence | | | | - |  |
| Libido № of participants: 1383 (3 RCTs included in this SR) [56] | - | - | - | SMD 0.17 SD higher (0.01 higher to 0.34 higher) | ⨁⨁⨁⨁ High^b^ | Testosterone replacement therapy results in a slight increase of libido compared to no testosterone replacement therapy in adolescents and adult males with endocrine deficits and low testosterone levels. |
| Erectile function № of participants: 1344 (4 RCTs included in this SR) [56] | - | - | - | SMD 0.16 SD higher (0.06 higher to 0.27 higher) | ⨁⨁⨁⨁ High^b^ | Testosterone replacement therapy results in a slight increase of erectile function compared to no testosterone replacement therapy in adolescents and adult males with endocrine deficits and low testosterone levels. |
| Sexual competence № of participants: (0 studies) | No evidence | | | | - |  |
| Adverse events assessed with: lower urinary tract symptoms score № of participants: 866 (2 RCTs included in this SR) [56] | - |  | - | MD 0.38 higher (0.67 lower to 1.43 higher) | ⨁⨁⨁◯ Moderate^a,b^ | Testosterone replacement therapy probably results in an increase of adverse events compared to no testosterone replacement therapy in adolescents and adult males with endocrine deficits and low testosterone levels. |
| ***The risk in the intervention group** (and its 95% confidence interval) is based on the assumed risk in the comparison group and the **relative effect** of the intervention (and its 95% CI). **Abbreviations**: CI: Confidence Interval; i.e.: that is, MD: Mean Difference; №: Number, RCTs: Randomized Controlled Trials, SD: Standard Deviation, SMD: Standardized Mean Difference, SR: Systematic Review | | | | | | |

Explanations

a. GRADE assessment of the systematic review by Ponce et al. 2018. [56]

b. GRADE assessment of the systematic review by Ponce et al. 2018 but downgraded for indirectness. [56]

Table S44: Summary of findings for KQ19

| **KQ19: Does cysteamine therapy in pregnancy compared to no cysteamine therapy affect maternal and child health in women with cystinosis?** | | | | | | |
| --- | --- | --- | --- | --- | --- | --- |
| Outcome № of participants (studies) | Relative effect (95% CI) | **Anticipated absolute effects (95% CI)** | | | Certainty | What happens |
|  |  |  |  | **Difference** |  |  |
| Progression of cystinosis and CKD № of participants: 16 (4 observational studies) [4; 7; 24; 43] | One patient showed stable graft function during pregnancy and no evidence of deterioration in renal function or cystinosis progression postnatally (serum creatinine <100 µmol/l). Another patient showed worsening renal function during pregnancy (up to serum creatinine 260 µmol/l) but comparable renal function to pregnancy levels at 6 months postpartum (Blakey et al. 2019). One patient showed increased eGFR during pregnancy (115 to 120ml/min/1.73m²) but stabilized postnatally (<80ml/min/1.73m²) (Chan et al. 2022). One patient had normal renal parameters during pregnancy (Kuczborska et al. 2019). Median eGFR of 12 patients was 43.0 ml/min (11.6–114.0) postnatally, corresponding to a 5.3 ml/min median eGFR decrease. 1 of them reached end-stage renal disease and started haemodialysis 6 months after delivery; 3 patients had a significant increase of serum creatinine by 20.3%, 32.3%, and 92.2% and decrease of eGFR of 8, 20, and 53 ml/min/1.73m² postnatally (Servais et al. 2022). | | | | ⨁◯◯◯ Very low^a,b^ | The evidence is very uncertain about the effect of cysteamine therapy compared to no cysteamine therapy on progression of cystinosis and chronic kidney disease in pregnant women with cystinosis and chronic kidney disease. |
| Fetotoxicity (e.g., malformations) № of participants: (0 studies) | No evidence | | | | - |  |
| Child development № of participants: 16 (4 observational studies) [7; 16; 40; 43] | All children (n=16) were healthy and developing well at last follow up (18 months, Chan et al. 2022; median age up to 3.9 years (0.5–35), Servais et al. 2022; 5 years and 3 months, Haase et al. 1995; 11 1/2 months, Reiss et al. 1988). | | | | ⨁◯◯◯ Very low^a,b^ | The evidence is very uncertain about the effect of cysteamine therapy compared to no cysteamine therapy on child development in pregnant women with cystinosis and chronic kidney disease. |
| Stillbirth № of participants: 20 (8 observational studies) [4; 7; 16; 24; 39-41; 43] | 19 women had no stillbirth (Chan et al. 2022, Haase et al. 2006, Kuczborska et al. 2019, Reiss et al. 1988, Robertson et al. 2022; Servais et al. 2022, Blakey et al. 2019). 1 women experienced stillbirth at 25 weeks (Ramappa et al. 2010). | | | | ⨁◯◯◯ Very low^a,b^ | The evidence is very uncertain about the effect of cysteamine therapy compared to no cysteamine therapy on stillbirth in pregnant women with cystinosis and chronic kidney disease. |
| Pregnancy and birth complications (gestational diabetes, preeclampsia, caesarean section, ...) № of participants: 20 (8 observational studies) [4; 7; 16; 24; 39-41; 43] | Out of 25 pregnancies, there were 7 early miscarriages, 2 ectopic pregnancies, and 1 preterm birth with neonatal death at 24 weeks (Blakey et al. 2019, Servais et al. 2022). A caesarean section was performed in 20 pregnancies, 15 due to prematurity and/or preeclampsia (Servais et al. 2022, Kuczborska et al. 2019), fetal distress, maternal respiratory compromise & pre-eclampsia (Blakey et al. 2019), 5 caesarean sections were planned or ≥36 weeks (Blakey et al. 2019, Reiss et al. 1988, Robertson et al. 2022). 2 pregnancies resulted in preterm vaginal labour due to spontaneous preterm rupture of membranes (Chan et al. 2022) or cervix insufficiency (Haase et al. 1995). Recurrent urinary tract infections occurred in 2 pregnancies (Chan et al. 2022, Kuczborska et al. 2019), acute cardiac failure leading to stillbirth in 1 pregnancy (Ramappa et al. 2010), mild pre-eclampsia, possible Streptococcus B amnionitis, cephalopelvic disproportion in 1 pregnancy (Reiss et al. 1988), severe back pain, in 1 pregnancy (Robertson et al. 2022), pre-eclampsia in 16 pregnancies (Servais et al. 2022, Kuczborska et al. 2019, Blakey et al. 2019, Reiss et al. 1988), and gestational diabetes in 2 pregnancies (Servais et al. 2022). | | | | ⨁◯◯◯ Very low^a,b^ | The evidence is very uncertain about the effect of cysteamine therapy compared to no cysteamine therapy on pregnancy and birth complications in pregnant women with cystinosis and chronic kidney disease. |
| Child ICU admission № of participants: 21 (7 observational studies) [4; 7; 16; 24; 39-41; 43] | 9 out of 21 children were admitted to ICU (Chan et al. 2022, Haase et al. 2006, Kuczborska et al. 2019, Reiss et al. 1988, Robertson et al. 2022; Servais et al. 2022, Blakey et al. 2019). | | | | ⨁◯◯◯ Very low^a,b^ | The evidence is very uncertain about the effect of cysteamine therapy compared to no cysteamine therapy on child intensive care unit asmission in pregnant women with cystinosis and chronic kidney disease. |
| SGA (small for gestational age) № of participants: 21 (7 observational studies) [4; 7; 16; 24; 39-41; 43] | Birth weight was appropriate for age in 8 children (Blakey et al. 2019, Chan et al. 2022, Haase et al. 2006, Kuczborska et al. 2019, Reiss et al. 1988, Robertson et al. 2022). They study by Servais et al. 2022 reported a median (range) birth weight of 2175g (620g-3374g) for 13 children. | | | | ⨁◯◯◯ Very low^a,b^ | The evidence is very uncertain about the effect of cysteamine therapy compared to no cysteamine therapy on small for gestational age occurance in pregnant women with cystinosis and chronic kidney disease. |
| ***The risk in the intervention group** (and its 95% confidence interval) is based on the assumed risk in the comparison group and the **relative effect** of the intervention (and its 95% CI). **Abbreviations**: CI: Confidence Interval; CKD: Chronic Kidney Disease, e.g.: for example, eGFR: estimated Glomerular Filtration Rate, ICU: Intensive Care Unit, n: number, №: Number, SGA: Small for Gestational Age | | | | | | |

Explanations

a. We used the JBI tool to assess risk of bias in the case series studies. GRADE starts with low certainty of evidence. We did not further downgrade for risk of bias.

b. Very small sample size; downgraded 3 steps for imprecision.

Table S45: Summary of findings for KQ20

| **KQ20: What is the effectiveness and safety of additional PPI (proton pump inhibitors) to cysteamine therapy compared to cysteamine therapy alone on health outcomes/ adverse events in patients with cystinosis?** | | | | | | |
| --- | --- | --- | --- | --- | --- | --- |
| Outcome № of participants (studies) | Relative effect (95% CI) | **Anticipated absolute effects (95% CI)** | | | Certainty | What happens |
|  |  |  |  | **Difference** |  |  |
| Quality of life № of participants: (0 studies) | No evidence | | | | - |  |
| Heart burn № of participants: 12 (1 observational study) [9] | The mean symptom score for heart burn decreased from 1.0 at baseline to 0 after 16 weeks of PPI therapy (p=0.016) (Dohil et al. 2005). | | | | ⨁◯◯◯ Very low^a,b^ | The evidence is very uncertain about the effect of proton pump inhibitors compared to no proton pump inhibitors on heart burn in patients with cystinosis who receive cysteamine therapy. |
| Rate of gastric ulcers № of participants: 12 (1 observational study) [9] | None of the patients (0/12) had gastric ulcers (Dohil et al. 2005). | | | | ⨁◯◯◯ Very low^a,b^ | The evidence is very uncertain about the effect of proton pump inhibitors compared to no proton pump inhibitors on rate of gastric ulcer in patients with cystinosis who receive cysteamine therapy. |
| Nausea № of participants: 12 (1 observational study) [9] | The mean symptom score for nausea/vomiting decreased from 1.2 at baseline to 0.1 after 16 weeks of PPI therapy (p=0.0039) (Dohil et al. 2005). | | | | ⨁◯◯◯ Very low^a,b^ | The evidence is very uncertain about the effect of proton pump inhibitors compared to no proton pump inhibitors on nausea in patients with cystinosis who receive cysteamine therapy. |
| GI perforation № of participants: 12 (1 observational study) [9] | None of the patients (0/12) had GI perforations (Dohil et al. 2005). | | | | ⨁◯◯◯ Very low^a,b^ | The evidence is very uncertain about the effect of proton pump inhibitors compared to no proton pump inhibitors on gastrointestinal perforation in patients with cystinosis who receive cysteamine therapy. |
| Vomiting № of participants: 12 (1 observational study) [9] | The mean symptom score for nausea/vomiting decreased from 1.2 at baseline to 0.1 after 16 weeks of PPI therapy (p=0.0039) (Dohil et al. 2005). | | | | ⨁◯◯◯ Very low^a,b^ | The evidence is very uncertain about the effect of proton pump inhibitors compared to no proton pump inhibitors on vomiting in patients with cystinosis who receive cysteamine therapy. |
| Appetite № of participants: (0 studies) | No evidence | | | | - |  |
| Weight gain/BMI № of participants: 12 (1 observational study) [9] | The weight gain or loss ranged from -0.8 to 2 kg after 16 weeks of PPI therapy (Dohil et al. 2005), | | | | ⨁◯◯◯ Very low^a,b^ | The evidence is very uncertain about the effect of proton pump inhibitors compared to no proton pump inhibitors on weight gain/BMI in patients with cystinosis who receive cysteamine therapy. |
| Adverse events № of participants: 12 (1 observational study) [9] | 1 out of 12 children (8.3%) experienced mild headaches and recurrence of oral canker sores (aphthous ulcers) during therapy (Dohil et al. 2005). | | | | ⨁◯◯◯ Very low^a,b^ | The evidence is very uncertain about the effect of proton pump inhibitors compared to no proton pump inhibitors on adverse events in patients with cystinosis who receive cysteamine therapy. |
| ***The risk in the intervention group** (and its 95% confidence interval) is based on the assumed risk in the comparison group and the **relative effect** of the intervention (and its 95% CI). **Abbreviations:** CI**:** Confidence Interval, GI: Gastrointestinal, №: Number | | | | | | |

Explanations

a. We used the Effective Public Health Practice Project (EPHPP) tool to assess risk of bias in the before–after studies. GRADE starts with low certainty of evidence. We further downgraded 1 step for high risk of bias.

b. Very small sample size; downgraded 3 steps for imprecision.

Table S46: Summary of findings for KQ21

| **KQ21: What is the effectiveness and safety of L-Carnitine and/or Co-Enzyme Q10 supplementation compared to no supplementation on muscle function in patients with cystinosis?** | | | | | | |
| --- | --- | --- | --- | --- | --- | --- |
| Outcome № of participants (studies) | Relative effect (95% CI) | **Anticipated absolute effects (95% CI)** | | | Certainty | What happens |
|  |  |  |  | **Difference** |  |  |
| Muscle strength № of participants: (0 studies) | No evidence | | | | - |  |
| Participation in professional and social life № of participants: (0 studies) | No evidence | | | | - |  |
| Quality of life № of participants: (0 studies) | No evidence | | | | - |  |
| Muscle function № of participants: 6 (1 observational study) [12] | Children receiving L-Carnitine had higher total muscle carnitine levels (nmol/mg protein) than untreated children, mean ± SD: 27.1 ± 5.4 (within normal range) vs. 10.9 ± 3.6 (outside normal range) (Gahl et al. 1993). | | | | ⨁◯◯◯ Very low^a,b^ | The evidence is very uncertain about the effect of L-Carnitine and/or Co-Enzyme Q10 supplementation compared to no supplementation on muscle function in patients with cystinosis. |
| Physical functioning № of participants: (0 studies) | No evidence | | | | - |  |
| Respiratory function № of participants: (0 studies) | No evidence | | | | - |  |
| Adverse events № of participants: 6 (1 observational study) [12] | None of the 6 children treated long-term with oral L-carnitine experienced any adverse events including nausea, vomiting, diarrhea, or a fish-like odor to their breath and skin (Gahl et al. 1993). | | | | ⨁◯◯◯ Very low^a,b^ | The evidence is very uncertain about the effect of L-Carnitine and/or Co-Enzyme Q10 supplementation compared to no supplementation on adverse events in patients with cystinosis. |
| ***The risk in the intervention group** (and its 95% confidence interval) is based on the assumed risk in the comparison group and the **relative effect** of the intervention (and its 95% CI). **Abbreviations:** CI**:** Confidence Interval, №: Number | | | | | | |

Explanations

a. We used the JBI tool to assess risk of bias in the case series studies. GRADE starts with low certainty of evidence. We did not further downgrade for risk of bias.

b. Very small sample size; downgraded 3 steps for imprecision.

Table S47: Summary of findings for KQ22

| **KQ22: What is the effectiveness and safety of preventive physiotherapy (i.e. strength training) compared to no physiotherapy on muscle function in patients with cystinosis?** | | | | | | |
| --- | --- | --- | --- | --- | --- | --- |
| Outcome № of participants (studies) | Relative effect (95% CI) | **Anticipated absolute effects (95% CI)** | | | Certainty | What happens |
|  |  |  |  | **Difference** |  |  |
| Muscle strength № of participants: 20 (1 observational study) [42] | The mean grip myometry did not change after 5 weeks of training: 22.2 KgF (95% CI 17.6 to 26.7) to 19.7 KgF (95% CI 15.4 to 24.0), p=0.131 (Sadjadi et al. 2020). | | | | ⨁◯◯◯ Very low^a,b^ | The evidence is very uncertain about the effect of preventive physiotherapy (strength training) compared to no physiotherapy on muscle strength in patients with cystinosis. |
| Participation in professional and social life № of participants: (0 studies) | No evidence | | | | - |  |
| Quality of life № of participants: 20 (1 observational study) [42] | QoL did not change after 5 weeks of training regarding emotional QoL (mean): 23.3 (95% CI 20.7 to 25.8) to 23.3 (95% CI 21.2 to 25.4), p=0.966, functional QoL (mean): 18.5 (95% CI 16.3 to 20.8) to 19.1 (95% CI 18.1 to 20.1), p=0.625, composite QoL (mean): 71.3 (95% CI 62.8 to 79.8), to 72.8 (95% CI 66.1 to 79.6), p=0.769, and physical QoL (mean): 29.7 (95% CI 24.1 to 35.3) to 31.2 (95% CI 26.9 to 35.4), p=0.668 (Sadjadi et al. 2020) | | | | ⨁◯◯◯ Very low^a,b,c^ | The evidence is very uncertain about the effect of preventive physiotherapy (strength training) compared to no physiotherapy on quality of life in patients with cystinosis. |
| Muscle function № of participants: (0 studies) | No evidence | | | | - |  |
| Physical functioning № of participants: 20 (1 observational study) [42] | Physical functioning did not change after 5 weeks of training when assessed by 9-Hole Peg Test (mean): 22.7 sec (95%CI 19.7 to 25.8) to 22.4 sec (95% CI 19.2 to 25.7), p=0.726, Timed Up and Go Test (mean): 7.9 sec (95% CI 7.0 to 8.8) to 8.1 sec (95% CI 6.9 to 9.3), p=0.870, Timed 25-Foot Walk (mean): 5.7 sec (95% CI 5.2 to 6.3) to 5.7 sec (95% CI 5.2 to 6.3), p=0.288, or 10-item Eating Assessment Tool (mean): 6.7 (95% CI 2.5 to 10.9) to 6.3 (95% CI 2.6 to 10.0), p=0.149 (Sadjadi et al. 2020) | | | | ⨁◯◯◯ Very low^a,b^ | The evidence is very uncertain about the effect of preventive physiotherapy (strength training) compared to no physiotherapy on physical functioning in patients with cystinosis. |
| Respiratory function № of participants: 20 (1 observational study) [42] | The mean MEP (maximum expiratory pressure) significantly improved after 5 weeks of training: 63.4 cmH_2_O (95% CI 45.4 to 81.4) to 80.9 cm H2O (95% CI 60.4 to 101.5), p<0.001. The mean PCF (peak cough flow) also significantly improved after 5 weeks of training: 233.2 l/min (95% CI 184.9 to 281.5) to 259.0 l/min (95% CI 207.5 to 310.5), p=0.022 (Sadjadi et al. 2020). | | | | ⨁◯◯◯ Very low^a,b^ | The evidence is very uncertain about the effect of preventive physiotherapy (strength training) compared to no physiotherapy on respiratory function in patients with cystinosis. |
| Adverse events № of participants: (0 studies) | No evidence | | | | - |  |
| ***The risk in the intervention group** (and its 95% confidence interval) is based on the assumed risk in the comparison group and the **relative effect** of the intervention (and its 95% CI). **Abbreviations:** CI**:** Confidence Interval, KgF: Kilogram Force, MEP: Maximum Expiratory Pressure, № = Number, PCF: Peak Cough Flow, QoL: Quality of Life, sec: seconds | | | | | | |

Explanations

a. We used the Effective Public Health Practice Project (EPHPP) tool to assess risk of bias in the before–after studies. GRADE starts with low certainty of evidence. We further downgraded 1 step for high risk of bias.

b. Very small sample size; downgraded 2 steps for imprecision.

c. M.D. Anderson Dysphagia Inventory (MDADI) tool developed to evaluate the impact of dysphagia on the quality of life (QOL) of patients with head and neck cancer; downgraded 1 step for indirectness.

Table S48: Summary of findings for KQ25

| **KQ25: What is the effectiveness and safety of combined treatment of cysteamine with concomitant cysteamine eye drops compared to systemic cysteamine treatment alone on vision loss and photophobia in patients with cystinosis?** | | | | | | |
| --- | --- | --- | --- | --- | --- | --- |
| Outcome № of participants (studies) | Relative effect (95% CI) | **Anticipated absolute effects (95% CI)** | | | Certainty | What happens |
|  |  |  |  | **Difference** |  |  |
| Vision loss № of participants: 5 (1 RCT) [5] | There were no substantial increases in visual acuity for any patient in the study although 3/5 (60%) patients showed a slight improvement in visual acuity in the treated compared with the untreated eye (Bradbury et al. 1991).^a^ | | | | ⨁◯◯◯ Very low^b,c^ | The evidence is very uncertain about the effect of systemic and local cysteamine hydrochloride eye drops compared to systemic cysteamine on vision loss in patients with cystinosis. |
| Photophobia № of participants: 5 (1 RCT) [5] | Subjective improvement in symptoms of photophobia in all patients (5/5) (Bradbury et al. 1991).^d^ | | | | ⨁◯◯◯ Very low^b,c^ | The evidence is very uncertain about the effect of systemic and local cysteamine hydrochloride eye drops compared to systemic cysteamine on photophobia in patients with cystinosis. |
| Neovascularization of the cornea, need for corneal transplantation № of participants: 15 (1 RCT) [30] | 0/15 (0%) patients experienced corneal neovascularization after treatment with local cysteamine eye drops (Liang et. al. 2017). | | | | ⨁◯◯◯ Very low^c,e^ | The evidence is very uncertain about the effect of systemic and local cysteamine hydrochloride eye drops compared to systemic cysteamine on neovascularization of the cornea, need for corneal transplantation in patients with cystinosis. |
| Quality of life № of participants: (0 studies) | No evidence | | | | - |  |
| Ocular surface disease № of participants: (0 studies) | No evidence | | | | - |  |
| Itchy eyes № of participants: 30 (2 RCTs) [30; 46] | 6/15 (40%) (Liang et al. 2017) and 1/15 (6.7%) (Tsilou et al. 2003) patients reported itchy eyes after treatment with local cysteamine eye drops. | | | | ⨁◯◯◯ Very low^c,f,g^ | The evidence is very uncertain about the effect of systemic and local cysteamine hydrochloride eye drops compared to systemic cysteamine on itchy eyes in patients with cystinosis. |
| Adherence № of participants: 32 (3 RCTs) [5; 21; 22] | Compliance ranged from 3/5 (60%) "good-very good" (Bradbury et al. 1991), 2/2 (100%) "excellent" (Kaiser-Kupfer et al. 1987) to better adherence in those responding to treatment (diminution in crystal density in treated eye): 40% (10/25) responded to treatment (Kaiser-Kupfer et al. 1990).^h^ | | | | ⨁◯◯◯ Very low^c,i^ | The evidence is very uncertain about the effect of systemic and local cysteamine hydrochloride eye drops compared to systemic cysteamine on adherence in patients with cystinosis. |
| Adverse events № of participants: 27 (2 RCTs) [21; 22] | None of the patients (0/27) with or without local cysteamine eye drops reported adverse events (Kaiser-Kupfer et al 1987; Kaiser-Kupfer et al. 1990).^j^ | | | | ⨁◯◯◯ Very low^c,i^ | The evidence is very uncertain about the effect of systemic and local cysteamine hydrochloride eye drops compared to systemic cysteamine on adverse events in patients with cystinosis. |
| ***The risk in the intervention group** (and its 95% confidence interval) is based on the assumed risk in the comparison group and the **relative effect** of the intervention (and its 95% CI). **Abbreviations:** CI**:** Confidence Interval, №: Number, RCT: Randomized Controlled Trial | | | | | | |

Explanations

a. Two before-after studies showed no change in vision or visual acuity (logMAR scale) after treatment with local cysteamine eye drops: 0/14 (0%) showed decreased vision (Iwata et al. 1998) [19] and mean, range: 0.8 (0.5 to 1.0) vs. 0.8 (0.3 to 1.0) (Al-Hemidan et al. 2017) [3]. A non-RCT found no difference in the visual acuity of the treated and untreated eyes (MacDonald et al. 1990) [32]. One study reported an absolute change of visual acuity (mean ± SD): 0.10 ± 0.15 after 90 days (Liang et al. 2017) [30].

b. The study has high concerns for risk of bias in the randomization process, deviations of intended interventions, measurement of outcome data, and selection of reported results. We downgraded 1 step for risk of bias.

c. Very small sample size; downgraded 3 steps for imprecision.

d. Three studies reported improvement in photophobia after treatment: improvement in photophobia (mean, SD): -0.63 (0.77), clinician assessed (Liang et al. 2017) [30]; at baseline 11/14 patients reported photophobia. After treatment, 5/6 patients who had reduction in crystal density reported subjective improvement of photophobia and/or discomfort. 1 did not have symptoms of photophobia at baseline (Iwata et al. 1998) [19]. Another study found that, compared to baseline (18/32, 56.3%, Grade 1=9, Grade 2=9), 4 more patients had photophobia after treatment with cysteamine eye drops (22/32, 68.8%, Grade 1=13, Grade 2=9). Among those with photophobia, 7/18 (38.9%) had improved symptoms, 6/18 (33.3%) were stable and 27.8% (5/18) developed more severe symptoms (Al-Hemidan et al. 2017) [3].

e. The study is an RCT but we only used data from one arm. It has some concerns for risk of bias in the randomization process, missing outcome data, and selection of reported results. We downgraded 2 steps.

f. The studies are RCTs but we only used data from one arm. They have some concerns for risk of bias in the randomization process, deviation from intended interventions, missing outcome data, and/or selection of reported results. We downgraded 2 steps.

g. Large difference in proportion patients with itchy eyes between the two studies.

h. Four studies reported good or excellent adherence data during treatment with local cysteamine eye drops: 93% (14/15) had compliance of <90% Study (Liang et al. 2017) [30] ; good compliance among all patients (Al-Hemidan et al. 2017) [3]; good to excellent: 95 % (18/19); good to excellent: (100%) 15/15 (Tsilou et al. 2003) [46]; adherence to eye drops 8 or more times a day: 8/14 and 5-7 times a day: 2/14 (Iwata et a. 1998) [19]. One study reported good compliance in 3/6 patients (Mac Donald et al. 1990) [32].

i. The studies have high or some concerns for risk of bias in the randomization process, deviations of intended interventions, measurement of outcome data, and selection of reported results. We downgraded 1 step for risk of bias.

j. Other studies reported 5/15 (33.3%) patients with eye specific adverse events (Liang et al. 2017) [30]; 4/15 (27%) total eyes with at least one adverse event (Tsilou et al. 2003) [46]; 1/14 (7.1%) patient with burning sensation in the eye, 1/14 (7.1%) patient with ameotrpic amblyopia, and 1/14 (7.1%) patient with vitreous hemorrhages (Iwata et al. 1998) [19].

Table S49: Summary of findings for KQ26

| **KQ26: What is the effectiveness and safety of systemic cysteamine treatment compared to no treatment on vision loss and photophobia in patients with cystinosis?** | | | | | | |
| --- | --- | --- | --- | --- | --- | --- |
| Outcome № of participants (studies) | Relative effect (95% CI) | **Anticipated absolute effects (95% CI)** | | | Certainty | What happens |
|  |  |  |  | **Difference** |  |  |
| Vision loss № of participants: 199 (1 observational study) [47] | 188/199 (94.5%) patients receiving oral cysteamine therapy had measurable visual acuity, 11/199 (5.5%) patients had counting fingers to no light perception vision in at least one eye. Measurable logMAR visual acuity ranged from 0.2 (Snellen equivalent, 20/12.5) to 1.5 (Snellen equivalent, 20/640) (Tsilou et al. 2006). | | | | ⨁◯◯◯ Very low^a,b^ | The evidence is very uncertain about the effect of systemic cysteamine treatment compared to no treatment on vision loss in patients with cystinosis. |
| Retinal pigment epithelium (RPE) changes № of participants: 153 (1 observational study) [47] | 90/153 (58.8%) patients receiving oral cysteamine therapy had hypopigmentation of the retinal pigment epithelium in the periphery with pigmentary stippling. 7/153 (4.6%) had a picture resembling retinitis pigmentosa with bone spicules and pigment clumps. 1/153 (0.7%) had severe chorioretinal atrophy (Tsilou et al. 2006). | | | | ⨁◯◯◯ Very low^a,b^ | The evidence is very uncertain about the effect of systemic cysteamine treatment compared to no treatment on retinal pigment epithelium changes in patients with cystinosis. |
| Visual field loss № of participants: 112 (1 observational study) [47] | Approximately half of 112 patients receiving oral cysteamine therapy had mild to severe abnormalities of the visual field. The frequency of visual field abnormalities increased with age (Tsilou et al. 2006). | | | | ⨁◯◯◯ Very low^a,b^ | The evidence is very uncertain about the effect of systemic cysteamine treatment compared to no treatment on visual field loss in patients with cystinosis. |
| Maculopathy № of participants: 112 (1 observational study) [47] | 14/153 (9.2%) patients receiving oral cysteamine therapy had macular pigmentary changes, 1/153 (0.7%) had retinal pigment epithelial atrophy in the perimacular area (Tsilou et al. 2006). | | | | ⨁◯◯◯ Very low^a,b^ | The evidence is very uncertain about the effect of systemic cysteamine treatment compared to no treatment on quality of life in patients with cystinosis. |
| Quality of life № of participants: (0 studies) | No evidence | | | | - |  |
| Intracranial changes № of participants: (0 studies) | No evidence | | | | - |  |
| Optic nerve changes № of participants: (0 studies) | No evidence | | | | - |  |
| Adverse events № of participants: (0 studies) | No evidence | | | | - |  |
| ***The risk in the intervention group** (and its 95% confidence interval) is based on the assumed risk in the comparison group and the **relative effect** of the intervention (and its 95% CI). **Abbreviations:** CI**:** Confidence Interval, №: Number | | | | | | |

Explanations

a. We used the JBI tool to assess risk of bias in the case series studies. GRADE starts with low certainty of evidence. We did not further downgrade for risk of bias.

b. Small sample size; downgraded 1 step for imprecision.

Table S50: Summary of findings for KQ27

| **KQ27: What is the adherence and safety of cysteamine eye drops with benzalkonium chloride compared to cysteamine eye drops without benzalkonium chloride on adverse events in patients with lifelong need for eye therapy?** | | | | | | |
| --- | --- | --- | --- | --- | --- | --- |
| Outcome № of participants (studies) | Relative effect (95% CI) | **Anticipated absolute effects (95% CI)** | | | Certainty | What happens |
|  |  |  |  | **Difference** |  |  |
| Adherence № of participants: 14 (1 RCT) [19] | Adherence to eye drops was 8 or more times a day in 8/14 (57.1%) and 5-7 times a day in 2/14 (14.3%), independent of eye drops with or without benzalkonium chloride (Iwata et al. 1998).^a^ | | | | ⨁◯◯◯ Very low^b^ | The evidence is very uncertain about the effect of eye drops with benzalkonium chloride compared to no cysteamine eye drops on adherence in patients with lifelong need for eye therapy (starting from childhood). |
| Quality of life № of participants: (0 studies) | No evidence | | | | - |  |
| Adverse events № of participants: 14 (1 RCT) [19] | There was no difference in burning sensation in the eyes between patients with benzalkonium chloride and without: 1/14 (7.1%) and 1/14 (7.1%) (Iwata et al. 1998).^c^ | | | | ⨁◯◯◯ Very low^b^ | The evidence is very uncertain about the effect of eye drops with benzalkonium chloride compared to no cysteamine eye drops on adverse events in patients with lifelong need for eye therapy (starting from childhood). |
| Obstacles/access to care № of participants: (0 studies) | No evidence | | | | - |  |
| ***The risk in the intervention group** (and its 95% confidence interval) is based on the assumed risk in the comparison group and the **relative effect** of the intervention (and its 95% CI). **Abbreviations:** CI**:** Confidence Interval, №: Number, RCT: Randomized Controlled Trial | | | | | | |

Explanations

a. Two before-after studies reported good to excellent adherence to eye drops containing benzalkonium chloride: compliance score >95% (Labbe et al. 2014) [26] and 95% good to excellent (Tsilou et al. 2003) [46].

b. Very small sample size; downgraded 3 steps for imprecision.

c. Two before-after studies reported either no adverse or serious adverse events related to eye drops containing benzalkonium chloride 0/8 (0%) (Labbe et al. 2014) [26] or 10/35 (28.6%) adverse events (Tsilou et al. 2003) [46].

Table S51: Summary of findings for KQ28

| **KQ28: What is the accuracy, reliability, repeatability of Optical Coherence Tomography (OCT) or corneal densitometry or slit lamp photography compared to in vivo confocal microscopy (IVCM) to monitor corneal cystine crystals in patients with cystinosis?** | | | | | | |
| --- | --- | --- | --- | --- | --- | --- |
| Outcome № of participants (studies) | Relative effect (95% CI) | **Anticipated absolute effects (95% CI)** | | | Certainty | What happens |
|  |  |  |  | **Difference** |  |  |
| Dynamics of crystal deposition or resolution under treatment № of participants: (0 studies) | No evidence | | | | - |  |
| Crystal load № of participants: (0 studies) | No evidence | | | | - |  |
| Reliability № of participants: (0 studies) | No evidence | | | | - |  |
| Distribution of crystals № of participants: (0 studies) | No evidence | | | | - |  |
| Repeatability № of participants: (0 studies) | No evidence | | | | - |  |
| Cornea thinning № of participants: 9 (2 observational studies) [25; 37] | There was no difference in CCT between AS-OCT and IVCM, mean ± SD: 543.47 ± 29.62 μm vs. 531.87 ± 34.77 μm. p=0.07 (Labbe et al. 2009) and 559 µm (right eye), 556 µm (left eye) vs. 551 μm (right eye) and 554 μm (left eye) (Ozdemir et al. 2019). | | | | ⨁◯◯◯ Very low^a,b^ | The evidence is very uncertain about the effect of OCT or corneal densitometry or slit lamp photography compared to IVCM on cornea thinning in patients with cystinosis. |
| Photophobia № of participants: 20 (1 observational study) [31] | The OCT-CysP (percentage of crystal infiltration) was correlated with clinician- and self-assessed photophobia scores (R²=0.33, p=0.0001 and R²=0.49, p<0.0001). The IVCM-CysS (density of crystal deposition) was correlated with clinician- and self-assessed photophobia scores (R²= 0.21, p=0.003 and R²=0.33, p=0.0001). The IVCM-CysS was also correlated with OCT-CysP (R²=0.27 and p=0.0006) (Liang et al. 2015). | | | | ⨁◯◯◯ Very low^b,c^ | The evidence is very uncertain about the effect of OCT or corneal densitometry or slit lamp photography compared to IVCM on photophobia in patients with cystinosis. |
| ***The risk in the intervention group** (and its 95% confidence interval) is based on the assumed risk in the comparison group and the **relative effect** of the intervention (and its 95% CI). **Abbreviations:** AS-OCT: Anterior Segment Optical Coherence Tomography, CCT: Central Cornea Thickness, CI**:** Confidence Interval, №: Number, OCT: Optical Coherence Tomography, R²: Regression coefficient, SD: Standard Deviation | | | | | | |

Explanations

a. We used the JBI tool to assess risk of bias in the case series studies. GRADE starts with low certainty of evidence. We further downgraded 1 step for risk of bias.

b. Very small sample size; downgraded 3 steps for imprecision.

c. We used the JBI tool to assess risk of bias in the case series studies. GRADE starts with low certainty of evidence. We did not further downgrade for risk of bias.

Table S52: Summary of findings for KQ31

| **KQ31: What is the efficacy and safety of interventions for psychosocial support compared to no psychosocial support to improve psychosocial well-being in patients with rare multi-organ diseases and caregivers?** | | | | | | |
| --- | --- | --- | --- | --- | --- | --- |
| Outcome № of participants (studies) | Relative effect (95% CI) | **Anticipated absolute effects (95% CI)** | | | Certainty | What happens |
|  |  |  |  | **Difference** |  |  |
| Resilience factors/protective factors of patients and caregivers № of participants: (0 studies) | No evidence | | | | - |  |
| Adherence assessed with: % of prescribed inhaled therapies taken follow-up: 12 months № of participants: 588 (1 RCT included in this SR) [54] | - |  | - | MD 9.5 higher (8.6 higher to 10.4 higher) | ⨁⨁⨁⨁ High^a^ | Psychosocial support increases adherence compared to no psychosocial support in patients with rare multi-organ diseases. |
| Quality of life assessed with: Treatment burden (Scale from: 0 to 100 -higher score indicates better QoL) follow-up: 12 months № of participants: 539 (1 RCT included in this SR) [54] | - |  | - | MD 3.9 higher (1.2 higher to 6.6 higher) | ⨁⨁⨁◯ Moderate^aA,b^ | Psychosocial support probably increases quality of life compared to no psychosocial support in patients with rare multi-organ diseases. |
| Educational/vocational outcomes in adulthood, educational outcomes in childhood/adolescence № of participants: (0 studies) | No evidence | | | | - |  |
| Psychosocial well-being № of participants: (0 studies) | No evidence | | | | - |  |
| Incidence or severity of psychosocial disorders  assessed with: Anxiety (scale from: 0 to 21 - higher score indicates greater anxiety) and  follow-up: 12 months № of participants: 535 (1 RCT included in this SR) [54] | - |  | - | MD 0.3 higher (0.4 lower to 1 higher) | ⨁⨁⨁◯ Moderate^a,b^ | There is probably no difference in the incidence or severity of anxiety-related psychosocial disorders in patients with rare multi-organ diseases with or without psychosocial support. |
| ***The risk in the intervention group** (and its 95% confidence interval) is based on the assumed risk in the comparison group and the **relative effect** of the intervention (and its 95% CI). **Abbreviation:** CI**:** Confidence Interval; MD**:** Mean Difference, №: Number, QoL: Quality of Life, RCT: Randomized Controlled Trial, SR: Systematic Review | | | | | | |

Explanations

a. Assessment taken from Dawson et al. 2023 but upgraded due to no indirectness [54].

b. The study had unclear risk of bias due to detection bias; downgraded 1 step for risk of bias.

**GRADE Working Group grades of evidence:**

**High certainty:** we are very confident that the true effect lies close to that of the estimate of the effect.
**Moderate certainty:** we are moderately confident in the effect estimate: the true effect is likely to be close to the estimate of the effect, but there is a possibility that it is substantially different.
**Low certainty:** our confidence in the effect estimate is limited: the true effect may be substantially different from the estimate of the effect.
**Very low certainty:** we have very little confidence in the effect estimate: the true effect is likely to be substantially different from the estimate of effect.

# Results

KQ4

Table S53: Results for KQ4

| **Author, Year** | **Study design,**  **Study duration,**  **Intervention arms,**  **Sample size** | **Survival** | **Renal involvement *** | **Quality of life **** | **Extra-renal involvement ***** | **Cystine level (nmol/1/2 Cystine/mg protein)** | **Growth** | **Adverse events ****** | **Cardiovascular involvement** |
| --- | --- | --- | --- | --- | --- | --- | --- | --- | --- |
| Brodin-Sartorius et al. 2012 [6] | Retrospective cohort study,  mean follow-up ± SD: 24.6 ± 7.0,  G1: cysteamine treatment <5 years  G2: cysteamine treatment 5 years and more  G3: untreated before end-stage renal disease,  N=86 | Overall: 62/86 (72.1%)  G1: 38/40 (95.0%)  G2: 20/35 (57.1%)  G3: 4/11 (36.4%)  G1 vs. G3 p<0.001  Kaplan Meyer estimates:  G1: NR  G2: NR  G3: NR  Survival curves show that life expectancy is significantly improved in the patients treated before the age of 5 years when compared with the absence of treatment (P = 0.03). Starting cysteamine therapy after 5 years of age still significantly improves the life expectancy in comparison with the untreated patients (P<0.05). | Incidence of ESRD  Overall: 78/86 (90.7%)  G1: 32/40 (80%)  G2: 8/8 (100%)  G3: 38/38 (100%)  p=0.006  Age at ESRD (Mean ± SD years)  G1: 13.4 ± 4.8  G2: 9.6 ± 2.6  G3: 9.5 ± 2.0  p<0.001 | NR | Incidence of hyothyroidism  G1: 21/40 (52.5%)  G2: 11/15 (73.3%)  G3: 30/31 (96.8%)  p<0.001  Age at hypothyroidism (mean ± SD, years)  G1: 13.5 ± 4.7  G2: 17.9 ± 7.3  G3: 11.7 ± 5.9  p=0.010  Incidence of diabetes  G1: 11/40 (27.5%)  G2: 11/17 (64.7%)  G3: 26/29 (89.7%)  p<0.001  Age at diabetes (mean ± SD, years)  G1: 17.6 ± 6.1  G2: 21.6 ± 10.1  G3: 14.4 ± 5.3  p=n.s.  Incidence of neuromuscular disorders  G1: 6/40 (15%)  G2: 15/28 (53.6%)  G3: 11/18 (61.1%)  p<0.001  Age at neuromuscular disorders (mean ± SD, years)  G1: 17.8 ± 5.2  G2: 26.6 ± 5.4  G3: 21.9 ± 5.5  p=0.012 | NR | NR | NR | NR |
| Emma et al. 2021 [10] | Non-comparative retrospective cohort study ^§^,  minimum follow-up: 3 years,  G1: cysteamine treatment  N=453 | NR | Median gain in renal survival (years): 9.1 | NR | NR | NR | NR | NR | NR |
| Gahl et al. 2007 [11] | Non-concurrent retrospective cohort study ^§§^,  40 years, grouped in 10-year increments,  G1: oral cysteamine treatment  G2: no cysteamine treatment,  N=100 | Overall: 67/67 (100%)  G1: NR  G2: NR | NR | NR | Patients with disease  0-10 years:  Diabetes  G1: n=73  G2: n=24  Myopathy  G1: n=73  G2: n=24  Pulmonary dysfunction  G1: n=53  G2: n=21  Death  G1: n=73  G2: n=21  11-20 years:  Diabetes  G1: n=22  G2: n=28  Myopathy  G1: n=22  G2: n=28  Pulmonary dysfunction  G1: n=20  G2: n=10  Death  G1: n=22  G2: n=28  21-30 years:  Diabetes  G1: n=5  G2: n=38  Myopathy  G1: n=5  G2: n=38  Pulmonary dysfunction  G1: n=4  G2: n=28  Death  G1: n=5  G2: n=38  31-40 years:  Diabetes  G1: n=0  G2: n=10  Myopathy  G1: n=0  G2: n=10  Pulmonary dysfunction  G1: n=0  G2: n=10  Death  G1: n=0  G2: n=10 | NR | NR | NR | NR |
| Gahl et al. 1987 [13] | Non-concurrent prospective cohort study ^§^,  up to 73 months,  G1: cysteamine treatment  G2: historical control,  N=148 | Overall: 143/148 (96.6%)  G1: 92/93 (98.9%)  G2: 51/55 (92.7%)  p=NR  Kaplan Meyer estimates:  G1: NR  G2: NR | NR | NR | NR | Mean ± SE  Overall: NR  G1:  baseline (before): 9.3 ± 1.0;  follow-up (after): 1.7 ± 0.2  G2: NR | Height increase, between 2-3 years of age:  G1: +0.24 SD of the normal mean  G2: -0.59 SD of the normal mean  Height increase in percent of normal during the first year of treatment, mean ± SEM:  G1: 73.5 ± 3.4  G2: 59.2 ± 3.7 | NR | NR |
| Greco et al. 2010 [15] | Non-comparative retrospective cohort study ^§§§^  Median follow-up: 17.6 years (range: 6.3-27.8),  G1: cysteamine treatment,  N=23 | NR | Factors associated with Stage 3 chronic renal failure (HR, 95%CI):  Age at last follow-up (years): 1.26 (1.10 to 1.45)  Cysteamine: age started (years): 1.32 (1.09 to 1.61)  Cysteamine: dose (g/m²/day): 0.19 (0.04 to 0.89) | NR | NR | NR | HtSDS < -2SD (OR, 95%CI):  Age at last follow-up (years): 1.23 (1.01 to 1.50)  Cysteamine: age started (years): 1.18 (0.84 to 1.64)  Cysteamine: dose (g/m²/day): 0.71 (0.07 to 6.89) | NR | NR |
| Hamed et al. 2022 [17] | Cross-sectional study,  NA,  G1: Cysteamine treatment (compliant)  G2: Cysteamine treatment (non-compliant),  N=15 | NR | CKD stage 5:  G1: 1/5 (20%)  G2: 5/10 (50%)  p=0.170  Renal replacement therapy:  G1: 3/5 (60%)  G2: 5/10 (50%)  p= 0.221 | NR | NR | NR | Overall, mean ± SD:  Weight: −2.48 ± 0.75  Height: −4.44 ± 2.31  No significant relation between cysteamine compliance and weight or height affection: p=0.37 and 0.129 (no data) | NR | NR |
| Markello et al. 1993 [33] | Retrospective cohort study,  Mean follow-up 4.5 to 7.1 years,  G1: adequate cysteamine treatment  G2: partial cysteamine treatment  G3: no cysteamine treatment,  N=76 | NR | Renal failure at study exit  G1: 0/17 (0%)  G2: 21/32 (65.6%)  G3: 16/27 (59.3%)  Predicted age of renal failure, years (95%CI):  G1: 74 (41 to 243)  G2: 20 (16 to 25)  G3: 10 (8 to 12) | NR | NR | Mean ± SD:  G1: 1.1 ± 0.7  G2: 1.7 ± 2.1  G3: 8.8 ± 5.5  p=0.001 | NR | NR | NR |
| Nesterova et al. 2015 [34] | Retrospective cohort study^§§§§^,  NR,  G1: cysteamine treatment  G2: no cysteamine treatment,  N=147 | NR | mean age at ESRD (years ± SEM):  G1: 15.4 ± 0.7  G2: 10.3 ± 0.3  Regression analysis:  Age at ESRD and cysteamine compliance: y=0.30x + 8.82 (R^2^ = 0.61);  slope of 0.30 years per unit of compliance and a correlation coefficient of 0.61 | NR | NR | Overall, mean (SEM):  2.35 ± 0.26  G1: NR  G2: NR | NR | NR | NR |
| Sonies et al. 2005 [44] | Cross-sectional study,  NR,  G1: cysteamine treatment,  N=101 | NR | NR | NR | Swallowing difficulty:  Swallowing Severity Score plotted against years without oral cysteamine therapy:  positive slope 0.04 units/years (p < 0.0001)  Swallowing Severity Score plotted against years on oral cysteamine therapy:  negative slope -0.04 units/years (p = 0.0012).  No difference when age is used as covariable.  Oral Muscle Composite Score plotted against years without oral cysteamine therapy:  positive slope 0.04 units/years (p = 0.0002).  Oral Muscle Composite Score plotted against years on oral cysteamine therapy:  negative slope 0.04 units/years (p < 0.0001).  No difference when age is used as covariable. | NR | NR | NR | NR |
| Spicer et al. 2015 [45] | Retrospective non-concurrent cohort study,  Mean follow-up: 12.8 years (SD ± 6.8; range: 0.9-24.2),  G1: cysteamine treatment  G2: no cysteamine treatment,  N=36 | 10-year survival after transplantation:  Number of patients with events and at-risk (n/N, %)  overall: 31/36 (86.1%)  G1: 15/17 (88.2%)  G2: 16/19 (84.2%)  Kaplan Meyer estimates:  G1: NR  G2: NR  p=0.9 | 10-year survival of first transplant (censored for death):  G1: NR  G2: NR  Kaplan Meyer estimates:  G1: n/N=NR (86%)  G2: n/N=NR (62%)  p=0.09 | NR | NR | NR | NR | NR | NR |
| Vaisbich et al. 2010 [48] | Non-concurrent cohort study,  NR,  G1: cysteamine treatment,  G2: Patients starting cysteamine treatment under 2 years of age  G3: Historical group without mercaptamine treatment until 1998; began receiving cysteamine treatment after 2 years of age  N=102 | NR | NR | NR | Overall:  Hypothyroidism: 63/102 (61.7%)  Diabetes: (n=8/102 (7.8%)  Muscular involvement: 7/102 (6.8%)  Hepatic involvement: 5/102 (4.9%)  Swallowing problem: 2/102 (1.9%)  Compromised central nervous system: 5/102 (4.9%) | Before cysteamine treatment, mean ± SD: 2.95 ± 1.84 (n=51);  After 3 months: 1.67 ± 0.99 (n=32) | NR | NR | NR |
| Van't Hoff et al. 1995 [50] | Before-after study,  4 months,  G1: cysteamine or phosphocysteamine treatment,  N=59 | NR | NR | NR | NR | Pretreatment (mean): 6.98  Post-treatment (mean): 4.79  p>0.20, 95% CI -0.02 to 4.39 | HtSDS:  Pre-treatment: -3.26  Post-treatment: -3.25  p>0.20, 95%CI -0.40 to +0.41 | NR | NR |

Abbreviations: CKD, chronic kidney disease; CI, confidence interval; ESRD, end-stage renal disease; HR, hazard ratio; HtSDS, Height standard deviation score; R², regression coefficient; SD, standard deviation; SE, standard error; SEM, standard error of mean; NR, not reported; N, number; n.s., not statistically significant; OR, odds ratio;

* including FANCONI-syndrome, kidney function, renal survival

** including general discomfort and pain

*** including muscle involvement (general muscle involvement, pulmonary involvement, late swallowing difficulty); neurological involvement (including neurocognitive involvement, pseudo tumor cerebri, strokes, seizures)

**** including halitosis, skin lesions, skin striae, vomiting, diarrhoea

^§^ labelled as “cohort study” by authors

^§§^ labelled as “case series” by authors

^§§§^ labelled as “retrospective study” by authors

^§§§§^ labelled as “retrospective chart analysis” by authors

KQ5

Table S54: Results for KQ5

| **Author, Year** | **Study design,**  **Study duration,**  **Intervention arms, Sample size** | **Treatment adherence** | **Quality of life *** | **Cystine level (WBC: nmol 1/2 cystine/mg protein)** | **Survival** | **Adverse events **** | **Renal involvement (eGFR: ml/min per 1.73 m²) ***** | **Extra-renal involvement ****** |
| --- | --- | --- | --- | --- | --- | --- | --- | --- |
| Ahlenstiel-Grunow et al. 2017 [1] | Before-after study ^§^,  Median follow-up: 14 months (range 3-18),  G1: delayed-release cysteamine treatment (PROCYSBI®)  G2: immediate-release cysteamine treatment (CYSTAGON®),  N=12 | NR | NR | WBC, median (range):  G1 (after/14 m): 1 (0–2.5)  G2: (before/baseline): 1 (0.2–5.7)  p=0.64 | NR | G1: 5/11 (45.5%)  G2: 7/12 (58.3%)  p=NR | eGFR, median (range):  G1 (after/13-15 m; n=5): 67 (21-91)  G2: (before/baseline/4 months before switch): 67 (26-90)  p=1.0 | NR |
| Gaillard et al. 2021 [14] | Prospective cohort study,  12 months,  G1: delayed-release cysteamine treatment (PROCYSBI®)  G2: immediate-release cysteamine treatment (CYSTAGON®),  N=17 | Adherence score, median (range):  G1: 1.8 (0.1-2)  G2: 0.5 (0.3 -1)  good adherence, median % of days (range):  G1: 88 (1-99)  G2: 2 (0-22)  partial or good adherence, median % of days (range):  G1: 91 (5-99)  G2: 43 (27-78)  daily exposition to treatment, hours/day (range):  G1: 22.8 (6.1-23.9)  G2: 14.9 (9.2-20.5) | NR | NR | NR | NR | NR | NR |
| Iyob-Tessema et al. 2021 [20] | Cross-sectional study,  NA,  G1: delayed-release cysteamine treatment (PROCYSBI®)  G2: immediate-release cysteamine treatment (CYSTAGON®),  N=76 | NR | NR | NR | NR | NR | NR | Grip strength, mean grip strength z-score (95% CI):  G1: NR  G2: NR  -0.38 (-0.88 to 0.11), p=0.13  adjusted for explanatory variables with a p<0.05 |
| Langman et al. 2012 [27] | Cross-over RCT,  2 weeks run-in period  6 weeks (3 weeks each arm),  G1: delayed-release cysteamine treatment (RP103)  G2: immediate-release cysteamine treatment (CYSTAGON®),  N=43 | NR | NR | Peak WBC cystine levels measured every morning over 3 consecutive days, least-squares mean (± SEM):  G1: 0.70 ± 0.19  G2: 0.97 ± 0.19  mean difference: -0.27 ± 0.36 (95.8% CI -0.63 to 0.09), p<0.001  0.3 noninferiority margin defined a priori | NR | Number of adverse events (can be more than one per person)  G1: 75  G2: 26  Gastrointestinal disorders  G1: 24  G2: 8  Metabolism and nutrition disorders  G1: 2  G2: 1  Nervous system disorders  G1: 3  G2: 0  Cardiac disorders  G1: 1  G2: 0  General disorders and administration site conditions  G1: 1  G2: 0  Infections and infestations  G1: 1  G2: 0  Renal and urinary disorders  G1: 1  G2: 0  Skin and subcutaneous tissue disorders  G1: 1  G2: 0  Vascular disorders  G1: 1  G2: 0 | NR | NR |
| Langman et al. 2014 [28] | Before-after study ^§§^,  24 months,  G1: delayed-release cysteamine treatment (PROCYSBI®)  G2: immediate-release cysteamine treatment (trade name: NR),  N=40 | NR | PedsQL percentage change from switch of IR to DR upon entering the study, intercept (p-value):  total: 5.99 (p=0.048)  physical: 6.62 (p=0.160)  emotional: 6.62 (p=0.136)  social: 11.23 (p=0.049)  school: 14.27 (p=0.004)  PedsQL percentage change from baseline to follow-up (ER treatment only), slope (p-value):  total: 0.302 (p=0.054)  physical: 0.019 (p=0.890)  emotional: 0.492 (p=0.201)  social: 0.126 (p=0.598)  school: 0.184 (p=0.072) | WBC, mean (± SD)  G1 (after/24 m): 0.55 ± 0.34  G2: (before/baseline): 0.43 ± 0.15  p=0.38 | NR | AE incidence per person per month  G1: 0.059  G2: 0.30  p=NR  overall: 40/40 (100%)  GI disorders: 35/40 (87.5%)  emesis: 28/40 (70.0%)  headache: 14/40 (35.0%)  upper respiratory tract symptoms: 9/40 (22.5%)  diarrhea: 8/40 (20.0%) | eGFR, mean (± SD):  G1 (after/24 m): 57 (± 25)  G2: (before/baseline): 63 (± 25)  p=0.32 | Height Z-score, mean (± SD):  G1 (after/24 m): −1.21 (± 0.96)  G2: (before/baseline): −1.15 (± 0.93)  p=0.46 |
| Quinaux et al. 2021 [38] | Cross-sectional study,  NA,  G1: delayed-release cysteamine treatment (PROCYSBI®)  G2: immediate-release cysteamine treatment (CYSTAGON®),  N=17 | NR | NR | Patients in the target for leukocyte hemicystin levels  G1: 9/10 (90%)  G2: 3/7 (43%)  p<0.05 | NR | NR | NR | Any bone symptoms:  G1: 5/10 (50%)  G2: 3/7 (43%)  p= n.s. |
| Vaisbich et al. 2022 [49] | Before-after study ^§§§^,  0.5 to 21 months,  G1: delayed-release cysteamine treatment (trade name: NR),  N=15 | NR | NR | Baseline; mean ± SD: 3.2 ± 3.0  Study exit: 0.8 ± 0.8  Percentage who reached WBC:  baseline: 3/15 (20%)  Study exit: 10/13 (77%) | NR | Incidence of adverse events:  15/15 (100%)  Vomiting: 12/15 (80%)  Upper respiratory tract infection: 8/15 (53.3%)  Gastroenteritis: 7/15 (46.7%)  Diarrhea: 5/15 (33.3%)  Dehydration: 4/15 (26.7%)  Pyrexia: 4/15 (26.7%)  Cough: 4/15 (26.7%)  Rhinitis: 3/15 (20.0%)  Breath odor: 3/15 (20.0%)  Nausea: 3/15 (20.0%)  Electrolyte imbalance: 2/15 (13.3%)  Rhinorrhea: 2/15 (13.3%)  Gastrostomy: 2/15 (13.3%)  Headache: 2/15 (13.3%)  Dermatitis diaper: 2/15 (13.3%) | eGFR, mean (± SD)  Baseline: 55.93 ± 22.43 (n = 15)  Study exit: 63.79 ± 21.44 (n = 14)  mean change of 8.14 ± 15.48 | Z score standing height, mean (± SD):  Baseline: - 3.2 (± 1.6)  Study exit: 0.1 (± 2.0)  Z score weight, mean (± SD):  Baseline: -4.0 (± 2.1)  Study exit: -1.10 (± 1.8)  Z score body surface are, mean (± SD):  Baseline: -1.8 (± 1.1)  Study exit: -1.3 (± 1.1)  Z score body mass index, mean (± SD):  Baseline: -1.0 (± 1.1)  Study exit: -1.2 (± 1.3) |
| Van Stein et al. 2021 [51] | Before-after study^§^,  1 day (several assessment points),  G1: delayed-release cysteamine treatment (PROCYSBI®)  G2: immediate-release cysteamine treatment (CYSTAGON®),  N=17 | NR | NR | Mean minimum concentration of cystine (in nmol cystine/mg protein; mean ± SD), after ingestion of a single dose  G1: 0.41 ± 0.41 (reached after 180min)  G2: 0.38 ± 0.3 (reached after 90min)  p=0.59 | NR | GI side effects (including nausea, vomiting, abdominal pain)  G1: 6/17 (35.3%)  G2: 15/17 (88.2%) | NR | NR |

Abbreviations: CI, confidence interval; DR, delayed-release cysteamine; eGFR, estimated glomerular filtration rate; GI, gastrointestinal; IR, immediate-release cysteamine; NA, not applicable; NR, not reported; N, number; PEDsQL, Pediatric Quality of Life Inventory; RCT, randomized controlled trial; SD, standard deviation; WBC, white blood cell;

* (general discomfort, pain, family quality of life, night sleep)

**(including halitosis, skin lesions, skin striae, vomiting, diarrhea)

*** (FANCONI-syndrome, kidney function, renal survival)

**** Neurological involvement (including neurocognitive involvement, pseudo tumor cerebri, strokes, seizures); Gastrointestinal involvement (including, delayed gastric emptying, motility problems, ulcers, liver involvement, pancreas involvement

^§^ labelled as “retrospective study” by authors

^§§^ labelled as “prospective, controlled, open label, single-arm study” by authors

^§§§^ labelled as “long-term, prospective open-label evaluation” by authors

KQ6

Table S55: Results for KQ6

| **Author, Year** | **Study design,**  **Study duration,**  **Intervention arms, Sample size** | **Rickets *** | **Serum levels of electrolytes** | **Dehydration episodes** | **Growth** | **Quality of life** | **Neurologic development and milestones** | **Adverse events **** |
| --- | --- | --- | --- | --- | --- | --- | --- | --- |
| Hohenfellner et al. 2022 [18] | Case series,  NA,  G1: electrolytes,  N=6 | NR | Serum values  Potassium (mmol/l): 3.5 to 4.4; 6/6 (100%) within normal range  Bicarbonate (mmol/l): 21 to 24.2; 6/6 (100%) within normal range  Phosphate (mmol/l): 0.74 to 1.64; 6/6 (100%) within normal range  Calcium (mmol/l): 2.26 to 2.46; 6/6 (100%) within normal range | NR | Height (Percentile), range: 27 to 90; 5/6 (83.3%) within normal range (no data for P5)  Weight (Percentile), range: 14 to 88; 5/6 (83.3%) within normal range (no data for P5)  Weight (kg), range: 15.1 to 62.7 | NR | NR | NR |

Abbreviations: NA, not applicable, NR, not reported; N, number;

* (clinical and biochemical markers)

** (e.g., nephrocalcinosis, urolithiasis)

KQ9

Table S56: Results for KQ9

| **Author, Year** | **Study design,**  **Study duration,**  **Intervention arms, Sample size** | **Dehydration episodes/polyuria** | **Quality of life** | **Serum levels of electrolytes** | **Growth** | **Rickets *** | **Bicarbonate** | **Disease progression **** | **Adverse events ***** |
| --- | --- | --- | --- | --- | --- | --- | --- | --- | --- |
| Emma et al. 2021 [10] | Retrospective cohort study,  37 years,  G1: indomethacin****  G2: no indomethacin  N=433 | NR | NR | NR | NR | NR | NR | Risk of CKD stage 5:  HR 0.95 (95% CI 0.68 to 1.34), p=0.78; n=368 (only children with cysteamine treatment before the age of 8 years) | NR |
| Greco et al. 2010 [15] | Retrospective cohort study,  Median follow-up: 17.6 years (range: 6.3–27.8 years),  G1: indomethacin*****  G2: no indomethacin,  N=23 | NR | NR | NR | Association of indomethacin with poor growth: HR 0.30 (95%CI 0.05 to 1.94), p=0.20 | NR | NR | Association of indomethacin with stage III chronic renal failure:  HR 1.10 (95% CI 0.35 to 3.36), p=0.87 | NR |

Abbreviations: CI, confidence interval; CKD, chronic kidney disease; HR hazard ratio; NR, not reported; N, number;

* (clinical and biochemical markers)

** (deterioration of kidney function)

*** (e.g. stomach ulcers)

**** 186 of 433 (43%) received indomethacin

***** 10 of 23 patients (43%) with indomethacin therapy

KQ11

Table S57: Results for KQ11

| **Author, Year** | **Study design,**  **Study duration,**  **Intervention arms, Sample size** | **Adverse events *** | **Estimated glomerular filtration rate (eGFR)** | **Albuminuria** | **Quality of life** |
| --- | --- | --- | --- | --- | --- |
| Levtchenko et al. 2003 [29] | Case series,  3 months,  G1: enalapril therapy,  N=5 | Hypotension (fatigue and dizziness): 2/5 (40%) | NR | Mean reduction: 43% (range 4-72%); albuminuria diminished in all patients | NR |

Abbreviations: NR, not reported; N, number;

* (including hyperkalemia, acute renal failure, hypotension, hypokalemia, ulcer, GI bleeding, hypertension)

KQ15

Table S58: Results for KQ15

| **Author, Year** | **Study design,**  **Study duration,**  **Intervention arms,**  **Sample size** | **Rate of fractures** | **Rate & severity of rickets/osteomalacia** | **Plasma Calcium** | **Radiologic evidence of active rickets** | **Bone deformity** | **Adverse events** | **Growth impairment** | **Quality of life** |
| --- | --- | --- | --- | --- | --- | --- | --- | --- | --- |
| Clarke et al. 1995 [8] | Before-after study ^§^,  46 months,  G1: calcium supplementation,  N=11 | NR | Pre-treatment (before): Osteomalacia: 9/11 (81.8%) | Pre-treatment (before): mean Calcium (mmol/l), (SEM): 2.33 (0.04)  Post-treatment (after): mean Calcium (mmol/l), (SEM): 2.20 (0.03) | NR | NR | NR | NR | NR |

Abbreviations: NR, not reported; N, number; SEM, standard error of the mean;

^§^ labeled as “Retrospective Maya Clinic case-note review” by authors

KQ16

Table 59: Results for KQ16

| **Author, Year** | **Study design,**  **Study duration,**  **Intervention arms,**  **Sample size** | **Renal function (CKD stage)** | **Quality of life** | **Growth** | **Adverse events** | **Intellectual function** | **Risk of visual spatial problems** | **Motor coordination** |
| --- | --- | --- | --- | --- | --- | --- | --- | --- |
| Ahmad et al. 2020 [2] | Retrospective cohort study ^§^,  NA,  G1: start of cysteamine treatment 0-24 months  G2: start of cysteamine treatment >24 months,  N=30 | eGFR (ml/min/1.73 m2)  Mean rank:  G1: 23.54  G2: 9.35  p=0.0001  Chitotriosidase (ŋmol/hr/ml)  Mean rank:  G1: 7.54  G2: 21.59  p=0.0001 | NR | NR | NR | NR | NR | NR |
| Brodin-Sartorius et al. 2012 [6] | Retrospective cohort study,  Mean follow-up (SD):  Overall: 24.6 (± 7.0): G1: 21.9 )± 4.5),  G2: 25.5 (± 7.3),  G3: 27.2 (± 8.0)  G1: Cysteamine treatment start <5 years  G2: Cysteamine treatment start 5 years and more  G3: untreated before end-stage renal disease  N=86 | ESRD:  G1: 32/40 (80%)  G2: 8/8 (100%)  G3: 38/28 (100%)  p=0.006 | NR | NR | NR | NR | NR | NR |
| Emma et al. 2021 [10] | Retrospective cohort study,  Minimum follow-up: 3 years  G1: cysteamine treatment  N=453 | Age at start of treatment and risk of CKD Stage 5:  HR 1.24 (95% CI 1.09 to 1.42) | NR | NR | NR | NR | NR | NR |
| Kimonis et al. 1995 [23] | Retrospective cohort study,  NR,  G1: start of cysteamine treatment < 2 years of age  G2: start of cysteamine treatment between 2 to 5 years of age, or poorly compliant  G3: start of cysteamine treatment > 5 years of age,  N=101 | NR | NR | Age-adjusted height z scores, mean ± SEM:  G1: -2.17 ± 0.39  G2: -3.04 ± 0.33  G3: -4.07 ± 0.39  G1 vs. G2 (p=0.09)  G1 vs G3 (p=0.001)  G2 vs G3 (p=0.033)  Mean bone age deficit, years:  G1: 1.37  G2: 2.42  G3: 3.76  Coefficient -0.06 (p=0.03), each year dealy in treatment with cysteamine resulted in decline in the z-score of 0.06.  Coefficient 0.149 (p=0.0003) increase in birth age deficit with increasing age at start of cysteamine therapy | NR | NR | NR | NR |
| Nießl et al. 2022 [35] | Retrospective cohort study,  23 years of follow-up (longitudinal data from 1997-2020)  G1: treatment start at ≥2 months of age  G2: treatment start at <2 months of age  N=52 | Kidney replacement:  Overall: 11/52 (21.2%)  G1: 11/46 (23.9%)  G2: 0/6 (0%)  eGFR (ml/min/1.73 m^2^) at last visit, mean (SD):  Overall: 87.9 (35.0); N=52  G1 (good adherence): 88.8 (35.0); N=34  G1 (less good adherence): 67.6 (20.2); N=12  G2: 123 (32.0); N=6 | NR | NR | NR | NR | NR | NR |
| O'Connell et al. 2022 [36] | Retrospective cohort study,  Data of patients from 1986-2020 (34 years): NR  Median survey duration was 2 years (IQR: 1–5, range: 1-17)  G1: oral cysteamine treatment,  N=74 | Renal survival, median (n=34):  Diagnosed <18 months of age: 21 years (95%CI: 16 to NR)  Diagnosed >18 months of age: 13 years (95%CI: 10 to NR)  p=0.033 | NR | NR | NR | NR | NR | NR |
| Vaisbich et al. 2010 [48] | Non-concurrent cohort study,  9 years  G1: oral cysteamine treatment  G2: starting cysteamine treatment under 2 years of age  G3: historical group without mercaptamine treatment until 1998; began receiving cysteamine treatment after 2 years of age,  N=48 | CKD stage:  1:  G1: 15/20 (75.0%)  G2: 3/18 (16.5%)  2-4:  G1: 5/20 (20.0%)  G2: 6/18 (33.5%)  5:  G1: 1/20 (5.0%)  G2: 9/18 (50.0%)  Dialysis:  G1: 0/20 (0%)  G2: 5/18 (27.8%)  Kidney transplantation:  G1: 1/20 (5.0%)  G2: 4/1 (22.2%) | NR | Growth (z-score), mean ± SD:  Weight Baseline:  G2: -4.35 ± 2.09  G3: NR  Weight Final:  G2: -2.78 ± 2.46  G3: NR  Stature Baseline:  G2: -3.93 ± 1.82  G3: -4.62 ± 1.57  Stature Final:  G2: -3.64 ± 1.92  G3: -4.16 ± 1.37 | NR | NR | NR | NR |
| Veys et al. 2023 [52] | Retrospective cohort study,  Up to 20 years follow-up,  G1: cysteamine treatment initiation < age 10 months (presymptomatic siblings)  G2: cysteamine treatment initiation ≥ age 10 months (symptomatic siblings),  N=52 | subjects reaching ESRD at age 12 years:  G1: 0% *  G2: 50% **  p=0.002 | NR | NR | NR | NR | NR | NR |
| Viltz et al. 2013 [53] | Cross-sectional study,  NR  G1: early cysteamine treatment (prior to or by the age of 2 years)  G2: later cysteamine treatment (after the age of 2 years),  N=46 | NR | NR | NR | NR | Wechsler Intelligence Scale, mean ± SD:  verbal IQ:  G1: 98.2 ± 11.1  G2: 88.6 ± 15.2  p=0.22  performance IQ:  G1: 92.7 ± 15.3  G2: 81.6 ± 13.2  p=0.23  full scale IQ:  G1: 94.0 ± 12.5  G2: 83.0 ± 15.1  p=0.13 | Test from the Woodcock-Johnson Psychoeducational Battery:  spatial relations:  G1: 104.5 ± 12.0  G2: 92.7 ± 22.6  p= 0.038 | NR |

Abbreviations: CI, confidence interval; CKD, chronic kidney disease; eGFR, estimated glomerular filtration rate; ESRD, end-stage renal disease; HR, hazard ratio; IQ, intelligence quotient; IQR, interquartile range; NA, not applicable; N, number; SD, standard deviation; SEM, standard error of the mean;

* subjects in G1 are mainly symptomatic and presymptomatic children with cystinosis

** subjects in G2 are mainly index children with cystinosis

^§^ labeled as “case control study” by authors

KQ17

Table S60: Results for KQ17

| **Author, Year** | **Study design,**  **Study duration,**  **Intervention arms, Sample size** | **Mood (depression)** | **Well-being** | **Muscle strength** | **Quality of life** | **Libido** | **Erectile function** | **Sexual competence** | **Adverse events** |
| --- | --- | --- | --- | --- | --- | --- | --- | --- | --- |
| Ponce at al. 2018 [56] | Systematic Review,  NA,  Arms: transdermal testosterone vs placebo,  N=1779 (4 studies) | SMD 0.08 (95%CI -0.03 to 0.20, N=1179, 2 trials; moderate COE (imprecision)) | NR | NR | NR | Sexual desire:  SMD 0.17 (95%CI 0.01 to 0.34; N=1383, 3 trials; high COE) | SMD 0.16 (95% CI 0.06 to 0.27; N=1344, 4 trials; high COE) | NR | Overall: NR  Lower urinary tract symptoms:  MD 0.38 (95% CI -0.67 to 1.43; N=866, 2 trials; moderate COE (imprecision))  erythrocytosis:  RR 8.14 (95% CI 1.87 to 35.40; N=1579, 3 trials; high COE) |

Abbreviations: CI, confidence interval; COE, certainty of evidence; MD, mean difference; NA, not applicable; NR not reported; N, number; RR, relative risk; SMD, standardized mean difference;

KQ19

Table S61: Results for KQ19

| **Author, Year** | **Study design,**  **Study duration,**  **Intervention arms, Sample size** | **Progression of cystinosis and CKD** | **Fetotoxicity** | **Child development** | **Stillbirth** | **Pregnancy and birth complications *** | **Child ICU admission** | **SGA** |
| --- | --- | --- | --- | --- | --- | --- | --- | --- |
| Blakey et al. 2019 [4] | Case series,  NR,  NA,  N=2 | P1: Stable graft function during pregancy and no deterioration in renal function or cystinosis progression postnatally (serum creatinine <100 µmol/L)  P2: Worsening renal function during pregnancy (up to serum creatinine 260 µmol/L) but comparable renal function to prepregnancy levels at 6 months postpartum | NR | NR | 0/2 (0%) | 2 cesarean sections at 37 weeks, one emergency CS in week 29+5 due to fetal distress, maternal respiratory compromise & pre-eclampsia, 4 first trimester miscarriages and 1 ectopic pregnancy in one women. | 10 days/ 8 weeks ICU: 2/3 (66.7%) | NR (3203g, 3374g, 1207g) |
| Chan et al. 2022 [7] | Case report,  NR,  NA,  N=1 | eGFR increased during pregancy (115 to 120mL/min/1.73m²), but stabilized postnatally (<80mL/min/1.73m²) | NA | Normal growth and development at 18 months | 0/1 (0%) | Recurrent UTIs, preterm labour (33w+1) due to spontanous preterm rupture of membranes | First 23 days | 1886g |
| Haase et al. 2006 [16] | Case report,  4 years,  NA,  N=1 | NR | NR | Infant without cystinosis and developing well at last follow-up (5 years 3 months) | 0% (0/1) | Preterm labour (31+5 weeks), cervix insufficiency | 3 days (respiratory dysfunction, 2 days CPAP+theophylline) | NR (2215g) |
| Kuczborska et al. 2019 [24] | Case report,  NR,  NA,  N=1 | Renal parameters remained normal in pregnancy | NR | NR | 0% (0/1) | Recurrent UTIs, 32weeks cesarean section due to pre-eclampsia | 0% (0/1) | NR (41 cm, 1400g) |
| Ramappa et al. 2010 [39] | Case report,  NR,  ,  N=1  NA | NR | NR | NA | 100% (1/1) - 25w | Acute cardiac failure | NA | NA |
| Reiss et al. 1988 [40] | Case report,  NR,  NA,  N=1 | NR | NA | Infant without cystinosis and developing well at last follow-up (to 11 1/2 months) | 0% (0/1) | Mild pre-eclampsia, possible Streptococcus B amnionitis, cephalopelvic disproportion - cesarean section at 36 weeks | 0% (0/1) | NR (48 cm, 2790g) |
| Robertson et al. 2022 [41] | Case report,  NR,  NA,  N=1 | NR | NR | NR | 0% (0/1) | Severe back pain, planned sectio (38 weeks) | 0 days (mother 24h at ICU) | No (50 cm/ 2415g; constitutional small infant) |
| Servais et al. 2022 [43] | Case series,  NR,  NA,  N=12 (19 pregnancies) | Median eGFR was 43.0 ml/min (11.6–114.0) postnatally, corresponding to a 5.3 ml/min median eGFR decrease. 1 out of 12 patients reached end-stage renal disease and started hemodialysis 6 months after delivery; in 3 patients significant increase of serum creatinine by 20.3%, 32.3%, and 92.2% and decrease of eGFR of 8, 20, and 53 l/min/1.73m² postnatally | NR | Children (n=13) were healthy at a median age at last follow up to 3.9 years (0.5–35) | 0% (0/12) | Pre-eclampsia: 7/15 (46.7%)  Gestational diabetes: 2/15 (13.3%)  19 pregnancies, 3 pontaneous early miscarriages (before 12 weeks), 1 ectopic pregnancy, 1 early pre-eclampsia (at 21 weeks), and 1 preterm birth with neonatal death at 24 weeks;  A cesarean section was performed in all patients (13/13, (100%)) due to prematurity and/or preeclampsia | 5/13 (38.5%)  Duration, mean: 8 weeks, range: 1.4-14 | NR (median birth weight 2175 (620-3374)) |

Abbreviations: CKD, chronic kidney disease; CPAP; continuous positive airway pressure; eGFR, estimated glomerular filtration rate; ICU, intensive care unit; NA, not applicable; NR, not reported; N, number; SGA, small gestational age; UTI, urinary tract infection;

* including: gestational diabetes, preeclampsia, cesarean section and others

KQ20

Table S62: Results for KQ20

| **Author, Year** | **Study design,**  **Study duration,**  **Intervention arms,**  **Sample size** | **Quality of life** | **Heart burn** | **Rate of gastric ulcers** | **Nausea** | **GI perforation** | **Vomiting** | **Appetite** | **Weight gain/ BMI** | **Adverse events** |
| --- | --- | --- | --- | --- | --- | --- | --- | --- | --- | --- |
| Dohil at al. 2005 [9] | Before-after study,  16 weeks,  G1: omeprazole,  N=12 | NR | Mean symptom score,  baseline: 1.0  16 weeks: 0  p=0.016 | 0% (1: duodenal erosions, but resolved after 16 weeks of esomeprazole) | Mean symptom score (nausea/vomiting) baseline: 1.2  16 weeks: 0.1 p=0.0039 | 0% | Mean symptom score (nausea/vomiting) baseline: 1.2  16 weeks: 0.1 p=0.0039 | NR (mean symptom score (anorexia); baseline: 1.2; 16 weeks: 0.3  p=0.0039 | Range, kg: -0.8 to 2 | Mild headaches and recurrence of oral canker sores with esomeprazole: 1/12 (8.3%) |

Abbreviations: NR, not reported; N, number;

KQ21

Table S63: Results for KQ21

| **Author, Year** | **Study design,**  **Study duration,**  **Intervention arms, Sample size** | **Muscle strength** | **Participation in professional life and social life** | **Quality of life** | **Muscle function** | **Physical functioning** | **Respiratory function** | **Adverse events** |
| --- | --- | --- | --- | --- | --- | --- | --- | --- |
| Gahl et al. 1993 [12] | Case series,  On average 5 years,  G1: with carnitine supplementation G2: without carnitine supplementation,  N=23 | NR | NR | NR | Total muscle carnitine (nmol/mg protein), mean ± SD:  G1: 27.1 ± 5.4 (within normal range)  G2: 10.9 ± 3.6 (outside normal range) | NR | NR | 0% (nausea, vomiting, diarrhea, or a fish-like odor to breath and skin) |

Abbreviations: NR, not reported; N, number; SD, standard deviation;

KQ22

Table S64: Results for KQ22

| **Author, Year** | **Study design,**  **Study duration,**  **Intervention arms, Sample size** | **Muscle strength** | **Participation in professional life and social life** | **Quality of life** | **Muscle function** | **Physical functioning** | **Respiratory function** | **Adverse events** |
| --- | --- | --- | --- | --- | --- | --- | --- | --- |
| Sadjadi et al. 2020 [42] | Before-after study,  13 months,  G1: expiratory muscle strength training,  N=22 | Grip myometry (kilogram force), mean:  Baseline: 22.2 (95%CI 17.6 to 26.7)  5 weeks: 19.7 (95%CI 15.4 to 24.0)  p= 0.131 (no significant changes after respiratory training) | NR | MDADI self-administered questionnaire designed to evaluate impact of dysphagia on QoL:  *Emotional (mean):* Baseline: 23.3 (95%CI 20.7 to 25.8)  5 weeks: 23.3 (95%CI 21.2 to 25.4)  p=0.966  *Functional (mean):* Baseline: 18.5 (95%CI 16.3 to 20.8), 5 weeks: 19.1 (95%CI 18.1 to 20.1), p=0.625  *Composite (mean):* Baseline: 71.3 (95%CI 62.8 to 79.8)  5 weeks: 72.8 (95%CI 66.1 to 79.6)  p=0.769  *Physical (mean):* Baseline: 29.7 (95%CI 24.1 to 35.3)  5 weeks: 31.2 (95%CI 26.9 to 35.4)  p=0.668 | NR | 9-Hole Peg Test (9-HPT) (mean):  Baseline: 22.7sec (19.7;25.8)  5 weeks: 22.4sec (19.2;25.7)  p=0.726  Timed Up and Go Test (TUG) (mean):  Baseline: 7.9sec (95%CI 7.0 to 8.8)  5 weeks: 8.1sec (95%CI 6.9 to 9.3)  p=0.870  Timed 25-Foot Walk (25-FW) (mean):  Baseline: 5.7sec (95%CI 5.2 to 6.3)  5 weeks: 5.7sec (95%CI 5.2 to 6.3)  p=0.288  10-item Eating Assessment Tool (EAT-10) (mean): Baseline: 6.7 (95%CI 2.5 to 10.9)  5 weeks: 6.3 (95%CI 2.6 to 10.0)  p=0.149 | Mean MEP (maximum expiratory pressure): Baseline: 63.4cmH^2^O (95% CI 45.4 to 81.4)  5 weeks: 80.9cmH^2^O (95% CI 60.4 to 101.5)  p<0.001  Mean PCF (peak cough flow): Baseline: 233.2L/min (95% CI 184.9 to 281.5)  5 weeks: 259.0L/min (95% CI 207.5 to 310.5)  p=0.022 | NR |

Abbreviations: CI, confidence interval; EAT-10, Eating Assessment Tool; 9-HPT, 9-Hole Peg Test; MDADI, MD Anderson Dysphagia Inventory; MEP, maximum expiratory pressure; NR, not reported; N, number; PCF, peak cough flow; QoL, quality of life; SD, standard deviation; TUG, Timed Up and Go Test; 25-FW, Timed 25 Foot Walk;

KQ25

Table S65: Results for KQ25

| **Author, Year** | **Study design,**  **Study duration,**  **Intervention arms,**  **Sample size** | **Vision loss** | **Photophobia** | **Neovascularization of the cornea, need for corneal transplantation** | **Quality of Life** | **Ocular surface disease** | **Itchy eyes** | **Adherence** | **Adverse events** |
| --- | --- | --- | --- | --- | --- | --- | --- | --- | --- |
| Al-Hemidan et al. 2017 [3] | Before-after study,  2-8 years,  G1: topical cysteamine 0.55% eye drops,  N=32 | Visual acuity (mean, range):  Before treatment: 0.8 (0.5 to 1.0)  After treatment: 0.8 (0.3 to 1.0) | Baseline: photophobia grade 0 = 14, grade 1 = 9, grade 2 = 9;  After treatment: from those with grade 0, 10 remained stable, 4 progressed to grade 1.  7/18 (38.9% )with photophobia had improved symptoms, 6/18 (33.3%) were stable and 5/18 (27.8%) developed more severe symptoms | NR | NR | NR | NR | Good compliance among all patients | NR |
| Bradbury et al. 1991 [5] | RCT,  6 month,  G1: 0,2% cysteamine drops  G2: placebo (saline),  N=5 | There were no substantial increases in visual acuity for any patient in the study, although 3/5 (60%) of patients showed a slight improvement in visual acuity in the treated compared with the untreated eye | Subjective improvement in symptoms (photophobia, blepharosspasm, visual acuity) and decreased corneal crystal density scores: 5/5 (100%) | NR | NR | NR | NR | Good-very good: 3/5 (60%),  Poor 2/5 (40%) * | NR |
| Iwata et al. 1998 [19] | Before-after study ^§^,  8-20 months,  G1: cysteamine drops with benzalkonium + oral cysteamine treatment,  N=14 | Decreased vision: 0/14 (0%) | Baseline: Photophobia of various degrees was reported by 11 of the 14 patients  General: "most patients did not recognize a difference in photophobia between the two eyes". Only reported for 6 patients who had reduction in crystal density: 5/6 (: 83.3%) subjective improvement of photophobia and/or discomfort, 1 patient did not complain about photophobia at baseline | NR | NR | NR | NR | Adherence to eye drops  8 or more times a day: 8/14 (57%)  5-7 times a day: 2/14 (14%)  1-4 times a day: 1/14 (7%)  discontinued: 1/14 (7%)  NA: 2/14 (14%) | Burning sensation in the eye: 1/14 (7.1%)  Ameotrpic amblyopia: 1/14 (7.1%)  Vitreous hemorrhages: 1/14 (7.1%) |
| Kaiser-Kupfer et al. 1987 [22] | RCT ^§§^,  4-5 months,  G1: 0.11% cysteamine eye drops  G2: placebo (saline),  N=2 | NR | NR | NR | NR | NR | NR | G1: 1/1 (100%)  G2: 1/1 (100%) | G1: 0/1 (0%)  G2: 0/1 (0%) |
| Kaiser-Kupfer et al. 1990 [21] | RCT ^§§^,  4-24 months,  G1: 0.1% or 0.5% cysteamine eye drops  G2: Placebo,  N=29 (25 remained in the trial) | NR | NR | NR | NR | NR | NR | Better compliance in those reaching the endpoint (response to treatment) | G1: 0/25 (0%)  G2: 0/25 (0%) |
| Liang et al. 2017 [30] | Before-after study ^§^,  90 days,  G1(baseline): viscous cysteamine 3.8 mg/ml (equivalent to 0.55% cysteamine) solution (Cystadrops),  N=32 (31 remained in the trial) | Visual acuity (logMAR scale), mean ± SD: Absolute change at day 90: 0.10 ± 0.15 | Investigator rated:  mean change before-after ± SD: -0.63 ±0.77 | 0/15 (0%) | NR | NR | Eye irritation:  G1: 6/15 (40%) | Compliance of <90%: 14/15 (93%) | Patients with one or more SAE (gastroenteritis, fatigue): 2/15 (3.3%)  Patients with one or more AE: 10/15 (66.7%)  Patients with eye specific AEs: 5/15 (66.7%) |
| MacDonald et al. 1990 [32] | nRCT,  7 months,  G1: 0.3% cysteamine eye drops  G2: placebo (saline),  N=4 | after 7 months, there was no difference in the visual acuity of the treated and untreated eyes. | NR | NR | NR | NR | NR | NR | NR |
| Tsilou et al. 2003 [46] | Before-after study ^§^,  1 year,  G1: standard cysteamine formulation 0.55%,  cysteamine hydrochloride solution with benzalkonium chloride  0.01%  G2 (after): 0.55% cysteamine hydrochloride solution with  monosodium phosphate 1.85%, disodium EDTA 0.10%, and  benzalkonium chloride 0.01%,  N=15 | NR | NR | NR | NR | NR | G1: 1/15 (7%) G2: 1/15 (7%) | Overall:  good to excellent: 15/15 (100%) | Total eyes with at least one AEs: G1: 4/15 (27%) G2: 4/15 (27%) |

Abbreviations: AE, adverse event; EDTA, ethylenediaminetetraacetic acid; NR, not reported; N, number; nRCT, non-randomized controlled trial; RCT, randomized controlled trial; SAE, serious adverse event; SD, standard deviation;

* self-categorized based on case description

^§^ labeled as “Randomized controlled trial (in person comparison)” by authors

^§§^ labeled as “Non-RCT” by authors

KQ26

Table S66: Results for KQ26

| **Author, Year** | **Study design,**  **Study duration,**  **Intervention arms, Sample size** | **Vision loss** | **Retinal pigment epithelium changes** | **Visual field loss** | **Maculopathy** | **Quality of life** | **Intracranial changes** | **Optic nerve changes** | **Neovascularization of the cornea** | **Adverse events** |
| --- | --- | --- | --- | --- | --- | --- | --- | --- | --- | --- |
| Tsilou et al. 2006 [47] | Case series,  28 years follow up,  G1: oral cysteamine treatment,  N=208 | 188/199 (94.5%) had visual acuity, 11/199 (5.5%) had counting fingers to no light perception vision in at least one eye | Hypopigmentation of the retinal pigment epithelium in the periphery with pigmentary stippling was present in 90/153 (58.8%) patients who underwent fundus examination. Other pigmentation changes: 7/153 patients (4.6%) had a picture resembling retinitis pigmentosa with bone spicules and pigment clumps; 1/153 (0.7%) had significant retinal pigment epithelial atrophy in the perimacular area | Of the 112 patients who underwent Humphrey static perimetry, approximately half had mild to severe abnormalities. The frequency of visual field abnormalities increased with age in cystinosis patients | 14/153 (9%) had macular pigmentary changes, 1/153 (0.5%) had retinal pigment epithelial atrophy in the perimacular area | NR | NR | NR | Submacular neovascular membrane: 1/208 (0.5%) | NR |

KQ27

Table 67: Results for KQ27

| **Author, Year** | **Study design,**  **Study duration,**  **Intervention arms, Sample size** | **Adherence** | **Quality of life** | **Adverse events** | **Obstacles/access to care** |
| --- | --- | --- | --- | --- | --- |
| Iwata et al. 1998 [19] | RCT,  8-20 months,  G1: cysteamine drops + oral cysteamine treatment  G2: cysteamine eye drops with benzalkonium + oral cysteamine treatment,  N=14 | Adherence to eye drops  Overall:  8 or more times a day: 8/14 (57.1%)  5-7 times a day: 2/14 (14.2%)  1-4 times a day: 1/14 (7.1%)  discontinued: 1/14 (7.1%)  NR: 2/14 (14.2%)  G1: NR  G2: NR | NR | Burning sensation in the eyes  Overall: 2/28 (7.1%)  G1: 1/14 (7.1%)  G2: 1/14 (7.1%)  Ametropic amblyopia  Overall: 1/14 (7.1%)  G1: NR  G2: NR  Vitreous hemorrhages  Overall: 1/14 (7.1%)  G1: NR  G2: NR | NR |
| Labbe et al. 2014 [26] | Before-after study ^§^,  4 years,  G1: cysteamine hydrochloride 0.55% eye drops (including benzalkonium chloride solution),  N=8 (16 eyes) | Mean compliance score until M18 above 95%. (daily reporting in diaries was difficult to maintain afterwards) | NR | SAE or AE related to eye drops:  G1: 0/8 (0%)  Patients with at least one AE (but related to underlying disease or unknown relationship): 7/8 (87.5%) | NR |
| Tsilou et al. 2003 [46] | Before-after study ^§§^,  6 months,  G1: standard cysteamine formulation eye drops  G2: new formulation eye drops | Overall:  Good to excellent: 18/19 (95%) | NR | Total eyes with at least one AE:  G1: 4/20 (20%)  G2: 6/15 (30%) | NR |

Abbreviations: AE, adverse event; NR, not reported; N, number; SAE, serious adverse event;

^§^ labeled as “open label, dose-response pilot study” by authors

^§§^ labeled as “RCT (in person comparison)” by authors

KQ28

Table S68: Results for KQ28

| **Author, Year** | **Study design,**  **Study duration,**  **Intervention arms, Sample size** | **Dynamics of crystal deposition or resolution under treatment** | **Crystal load** | **Reliability** | **Distribution of crystals** | **Repeatability** | **Cornea thinning** | **Photophobia** |
| --- | --- | --- | --- | --- | --- | --- | --- | --- |
| Labbe et al. 2009 [25] | Case series,  NA (cross-sectional),  G1: AS-OCT  G2: slit lamp  G3: IVCM,  N=8 (16 eyes) | NR | NR | NR | NR | NR | CCT (μm), mean:  G1: 543.47 ± 29.62  G2: NR  G3: 531.87 ± 34.77  P = 0.07 | NR |
| Liang et al. 2015 [31] | Case series,  NA (cross-sectional),  G1: AS-OCT  G2: slit lamp  G3: IVCM  G4: self-assessed photophobia,  N=20 (40 eyes) | NR | NR | NR | NR | NR | Depth of crystal deposition in cornea:  OCT-CysP, average: 49.56 ± 27.31% for a mean CCT of 558.63 ± 48.83µm. The OCT-CysP was correlated with the two evaluations of photophobia (R²=0.33, P=0.0001 for the clinician-assessed evaluation and 0 R²=0.49, P<0.0001 for the self-assessed evaluation) | G1: OCT-CysP was correlated with the  two evaluations of photophobia  G2: clinician-assessed photophobia: 1.70 ± 1.41  G3: A correlation  was observed between clinician- and self-assessed photophobia  scores, and the density of crystal deposition evaluated with the  IVCM-CysS  G4: self-assessed photophobia, mean ± SD: 2.10 ± 1.28  Mean assessments were correlated: R² = 0.61, p <0.0001 |
| Ozdemir et al. 2019 [37] | Case report,  1 year,  G1: AS-OCT  G2: IVCM,  N=1 (2 eyes) | NR | NR | NR | NR | NR | G1: cystine crystals as hyperreflective dots in the anterior and mid‑stroma with the depth of 415 μm (CCT: 559µm) in the right eye and 238 µm (CCT: 556µm) in the left eye.  G2 (IVCM): Depth of crystal accumulation (DC) was 338 μm (CCT: 551 μm) in the right eye and 371 μm (CCT: 554 μm) in the left eye. Bilateral CDS was 2 in the subbasal nerve layer, 3 in the anterior stroma, 3 in the middle stroma, and 1 in the posterior stroma (total 9 points) | relieved during the treatment |

Abbreviations: AS-OCT, anterior segment optical coherence tomography; CCT, central corneal thickness; IVCM, in vivo confocal microscopy; IVCM-CysS, crystal density score detected by IVCM; NR, not reported; N, number; OCT-CysP, percentage of the corneal thickness detected by OCT; R² regression coefficient; SD, standard deviation;

KQ31

Table S69: Results for KQ31

| **Author, Year** | **Study design,**  **Study duration,**  **Intervention arms, Sample size** | **Resilience factors/protective factors of patients and caregivers** | **Adherence** | **Quality of life** | **Educational/vocational outcomes in adulthood, educational outcomes in childhood/adolescence** | **Psychosocial well-being** | **Incidence or severity of psychosocial disorders** |
| --- | --- | --- | --- | --- | --- | --- | --- |
| Martin et al. 2019 [55] | Guideline,  NA,  NA,  NA | NR | NR | We strongly recommend easy access to psychosocial support to improve Quality of life (QoL);  Grade strength: B;  Quality of evidence (average): 2++ 5 studies A multidisciplinary approach in treating EB improves QoL for individuals with EB • Psychological support and close monitoring of EB improves QoL. • They facilitate participation in social activities. • Patients with all types of EB including EBS report a great impairment in QoL due to restrictions in physical and social activities.  We strongly recommend access to psychosocial family support to improve the family QoL; Grade strength: B;  Quality of evidence (average): 2+ 3 studies Early psychosocial support to improve QoL of the family unit for all subtypes EB and children with high infantile mortality: • As caregivers QoL may also be impacted. • Psychological support and close monitoring helps. • Support is essential for family of palliative patients with EB | NR | We strongly recommend psychosocial support to improve well-being; Grade strength: C;  Quality of evidence (average): 2+ 5 studies To promote self-efficacy and support around body image to aid psychological well-being • Having access to knowledge and resources about EB can help people have a greater role in managing their EB. This self-management can help improve well-being. • Improved self-efficacy and locus of control, as well as support around body-image could help to develop a more positive sense of well-being. For support during transition periods in life (school transitions, transition into adulthood) • Communication and education about EB to improve people’s understanding. • Support from families, EB healthcare professionals and DEBRA.  We strongly recommend psychosocial support to improve the family well-being Grade strength: C;  Quality of evidence (average): 2- 6 studies Support for the family to reduce emotional burden of caring for someone with EB and improve well-being for the family unit: • Home nursing can provide much needed relief and support for primary caregivers and could reduce the need for hospital admission. • Actively assist in seeking counselling before the family unit is irreparably destroyed. • Provide information about the nature, course and outcome of EB. • Provide training in the management of patient symptoms. • Access to Social media and face to face EB support groups might be beneficial for families. Promoting family well-being can help the family enhance their strong and positive influence for those living with EB • The way the family reacts to EB can be psychologically assimilated by the person with EB, particularly children. • Acceptance of the EB by the family is important and can make it more bearable for the patient. | NR |
| Dawson et al. 2023 [54] | Systematic Review,  NA,  G1: psychological intervention (self-management intervention comprising digital platform and behaviour change sessions; life coaching intervention; problem-solving intervention)  G2: treatment as usual (which usually consists of an annual review or routine reviews (or combination of both) with a specialist CF multidisciplinary team),  N=1642 (10 studies) | NR | % of prescribed inhaled therapies taken (mean, SD), follow-up 12 months, 1 study (n=588):  G1: NR  G2: NR  MD (adjusted) 9.50 (95%CI 8.60 to 10.40), moderate COE (downgraded once due to indirectness. The evidence includes only adults with CF aged 16 years and older and was not designed to answer the specific question posed in this review.) | Treatment burden (Scale from: 0 to 100 -higher score indicates better QoL), (mean, SD), follow-up 12 months, 1 study (n=539):  G1: NR  G2: NR  MD (adjusted) 3.90 (95%CI 1.20 to 6.60), low COE (downgraded once due to indirectness. The evidence includes only adults with CF aged 16 years and older and was not designed to answer the specific question posed in this review. Downgraded once due to unclear risk of detection bias, as outcome assessors were not blinded.) | NR | NR | Anxiety (scale from: 0 to 21, higher score indicates greater anxiety), (mean, SD), follow-up 12 months, 1 study (n=535):  G1: NR  G2: NR  MD (adjusted) 0.30 (95%CI -0.40 to 1.00), low COE (Downgraded once due to indirectness. The evidence includes only adults with CF aged 16 years and older and was not designed to answer the specific question posed in this review. Downgraded once due to unclear risk of detection bias, as outcome assessors were not blinded.)  Depression (scale from: 0 to 24, higher score indicates greater depression), (mean, SD), follow-up 12 months, 1 study (n=534):  G1: NR  G2: NR  MD -0.10 (95%CI -0.80 to 0.60), low COE (downgraded once due to indirectness. The evidence includes only adults with CF aged 16 years and older and was not designed to answer the specific question posed in this review. Downgraded once due to unclear risk of detection bias, as outcome assessors were not blinded.)  No evidence of treatment-related adverse events. |

Abbreviations: CF, cystic fibrosis; CI, confidence interval; COE, certainty of evidence; EB, epidermolysis bullosa; SMD, standardized mean difference; MD, mean difference; NA, not applicable; NR not reported; N, number; QoL, quality of life

# Subgroup results

KQ 4

Table S70: Subgroup results for KQ4

| **Author, Year** | **Age** | **Age at start of treatment** | **Dosage per age and body weight/**  **dosage/**  **dosage regimes/**  **maximum dosage/**  **frequency of administration** | **Type of cystinosis** | **Pre-/post transplantation** |
| --- | --- | --- | --- | --- | --- |
| Nesterova et al. 2015 [34] | NR | NR | no correlation between age at ESRD and mean cysteamine dosage in mg/kg/day: y=−0.0194x +16.647 (R²=0.0016) | NR | NR |
| Greco et al. 2010 [15] | NR | Factors associated with Stage 3 chronic renal failure  Cysteamine: age started (years): HR 1.32 (95%CI 1.09 to 1.61)    HtSDS < -2SD:  Cysteamine: age started (years): OR 1.18 (95%CI 0.84 to 1.64) | Factors associated with Stage 3 chronic renal failure:  Cysteamine: dose (mg/kg/day): HR 0.97 (95%CI  0.93 to 1.00)  Cysteamine: dose (g/m²/day):HR 0.19 (95%CI  0.04 to 0.89)    HtSDS < -2SD  Cysteamine (dose mg/Kg/day): OR 0.98 (95%CI 0.92 to 1.04)  Cysteamine (dose: g/m²/day): OR 0.71 (95% CI 0.07 to 6.89) | NR | NR |
| Vaisbich et al. 2010 [48] | NR | G2: start of cysteamine treatment under 2 years of age (n=20)  G3:  start of cysteamine treatment above 2 years of age (n=18)    Hypothyroidism:  G2: 2/20 (10%)  G3: 11/18 (61.1%)  Diabetes:  G2: 1/20 (5%)  G3: 0/18 (0%)  Muscular involvement:  G2: 0/20 (0%)  G3: 0/18 (0%)  Hepatic involvement:  G2: 0/20 (0%)  G3: 1/18 (5.5%)  Swallowing problem:  G2: 0/20 (0%)  G3: 0/18 (0%)  Compromised central nervous system:  G2: 0/20 (0%)  G3: 5.5% (n=1/18)    CKD stage:  1:  G2: 15/20 (75%)  G3: 3/18 (16.5%)  2-4:  G2: 5/20 (20%)  G3: 6/18 (33.5%)  5:  G2: 1/20 (5%)  G3: 9/18 (50%)    Dialysis:  G2: 0  G3: 5/18 (27.8%)  Kidney transplantation:  G2: 1/20 (5%)  G3: 4/18 (22.2%)    Growth (z-score), mean ± SD:  Weight Baseline:  G2: -4.35 ± 2.09  G3: NR  Weight Final:  G2: -2.78 ± 2.46  G3: NR  Stature Baseline:  G2: -3.93 ± 1.82  G3: -4.62 ± 1.57  Stature Final:  G2: -3.64 ± 1.92  G3: -4.16 ± 1.37    Recombinant growth hormone:  G2: 0/20 (0%)  G3: 15/18 (83.3%) | NR | NR | NR |
| Gahl et al. 2007 [11] | Duration of cysteamine treatment:  G1: <8 years  G2: ≥8 years  Mean ± SD  Height, cm  G1: 143.6 ± 11.2  G2: 154.7 ± 10.8  Weight, kg  G1: 45.3 ± 10.7  G2: 53.2 ± 10.4  Age at transplantation, y  G1: 11.0 ± 3.2)  G2: 14.8 ± 4.6)  Complications* per patient, n  G1: 4.0 ± 2.0  G2: 2.2 ± 2.2  Hypothyroidism  G1: 53/61 (87%)  G2: 22/39 (56%)  Deaths  G1: 30/61 (49%)  G2: 3/39 (8%) | NR | NR | NR | NR |
| Van't Hoff et al. 1995 [50] | NR | Patients who started cysteamine at less than 2 years of age (n=20):  HtSDS, mean:  Pre-treatment: -2.59  Post-treatment: -2.84  (P>0.20, 95%CI -0.89 to 0.30) | NR | NR | NR |

Abbreviations: CI, confidence interval; CKD, chronic kidney disease; ESRD, end-stage renal disease; HR, hazard ratio; HtSDS, height standard deviation score; NR, Not reported; N, number; OR, odds ratio; R²; regression coefficient; SD, standard deviation;

* composite of hypothyroidism, pulmonary dysfunction, swallowing abnormalities, myopathy, retinopathy, vascular calcification, diabetes mellitus, and cerebral calcification

KQ 5

Table S71: Subgroup results for KQ5

| **Author, Year** | **Age** | **Frequency of administration** | **Dietary recommendation for administering Cysteamine** |
| --- | --- | --- | --- |
| Iyob-Tessema et al. 2021 [20] | Difference in grip strength z-score, mean (95%CI):  -0.006 (–0.01 to -0.001),  p=0.02 (older age at treatment initiation associated with lower grip strength) | NR | NR |

Abbreviations: CI, confidence interval; NR, Not reported;

KQ 16

Table S72: Subgroup results for KQ16

| **Author, Year** | **Age at start of treatment** |
| --- | --- |
| Veys et al. 2023 [52] | Presymptomatic siblings:  age: 0.95 months (IQR: 0.2; 1.4)  ESRD: 0/9 (0%)  age at ESRD: NA  extra-renal complications: 1 (0; 2)  Index counterparts:  age: 22 months (IQR: 16; 28); p=0.004  ESRD: 2/7 (28.6%); p=0.47  age at ESRD: 13 ± 3 years; p=NA  extra-renal complications: 2 (0.5; 2.5); p=0.25  Symptomatic siblings:  age: 12 months (IQR: 8; 31 months)  ESRD: 10/17 (58.8%)  age at ESRD: 13 ± 3 years  extra-renal complications: 2 (1; 4)  Index counterparts:  age: 41 months (IQR: 21; 75 months); p=0.0001  ESRD: 14/17 (82.4%); p=0.26  age at ESRD: 10 ± 3 years; p=0.002  extra-renal complications: 2 (1; 3); p=0.63 |

Abbreviations: ESKD, end-stage kidney disease; IQR, interquartile range; NA, not applicable;

KQ 26

Table S73: Subgroup results for KQ26

| **Author, Year** | **Age** |
| --- | --- |
| Tsilou et al. 2006 [47] | Visual acuity  Visual acuity decreased with age patients < 10 years: Mean ± standard error of the mean logMAR visual acuity was 0.17± 0.043 (Snellen equivalent, 20/32+1); 10-19 years: 0.07± 0.024 (Snellen equivalent, 20/25+1); 20-29 years: 0.16 ± 0.032 (Snellen equivalent of 20/32+2); 30 y and older: 0.38 ± 0.054 (Snellen equivalent of 20/50+1)  Visual fields  The frequency of visual field abnormalities increased with age in cystinosis patients  Age retinal changes  The mean standard error of the mean age of patients without retinal crystals was 15.6 ± 0.6 years, whereas the mean age of patients with retinal crystals was 27.6 ± 1.4 years (P<0.001).  ERG findings  ERG was performed in 31 patients aged 15 to 40 years. The mean age of the 15 patients with a normal ERG was 23.5 years, whereas the mean age of the 16 patients with abnormal ERG results was 27.0 years. |

Abbreviations: ERG, electroretinography

KQ 28

Table S74: Subgroup results for KQ28

| **Author, Year** | **Age** |
| --- | --- |
| Labbe et al. 2009 [25] | Photophobia  Photophobia scores were moderately associated with the patient’s age (clinician-assessed score: R² =0.18 and P = 0.006; self-assessed score: R²= 0.30 and P = 0.0003).  Photophobia intraleukocyte cystine concentration  There was no correlation between ICC and photophobia scores ( clinician assessed score: R²=0.02, P = 0.42; self-assessed score: Fig. 1D, R²=0.03, P=0.25).  Photophobia and renal transplantation  A medical history of renal transplantation was also correlated with the self-assessed photophobia score (R²=0.32, P=0.04). |

# References

1. Ahlenstiel-Grunow T, Kanzelmeyer NK, Froede K, Kreuzer M, Drube J, Lerch C, et al. Switching from immediate- to extended-release cysteamine in nephropathic cystinosis patients: a retrospective real-life single-center study. Pediatr Nephrol. 2017;32(1):91-7.

2. Ahmad Z, Shatha HA, Sameh H. Impact of age at starting cysteamine therapy on serum chitotriosidase in cystinotic iraqi children. Medico-Legal Update. 2020;20(4):911-6.

3. Al-Hemidan A, Shoughy SS, Kozak I, Tabbara KF. Efficacy of topical cysteamine in nephropathic cystinosis. Br J Ophthalmol. 2017;101(9):1234-7.

4. Blakey H, Proudfoot-Jones J, Knox E, Lipkin G. Pregnancy in women with cystinosis. Clin Kidney J. 2019;12(6):855-8.

5. Bradbury JA, Danjoux JP, Voller J, Spencer M, Brocklebank T. A randomised placebo-controlled trial of topical cysteamine therapy in patients with nephropathic cystinosis. Eye. 1991;5(Pt 6):755-60.

6. Brodin-Sartorius A, Tete MJ, Niaudet P, Antignac C, Guest G, Ottolenghi C, et al. Cysteamine therapy delays the progression of nephropathic cystinosis in late adolescents and adults. Kidney Int. 2012;81(2):179-89.

7. Chan L, Wichart J, Kiang T, Khurana R, Gangoiti JA, Barshop BA, et al. Pregnancy and Breastfeeding in Nephropathic Cystinosis With Native Kidneys. KI Rep. 2022;7(7):1716-9.

8. Clarke BL, Wynne AG, Wilson DM, Fitzpatrick LA. Osteomalacia associated with adult Fanconi's syndrome: clinical and diagnostic features. Clin Endocrinol (Oxf). 1995;43(4):479-90.

9. Dohil R, Fidler M, Barshop B, Newbury R, Sellers Z, Deutsch R, et al. Esomeprazole therapy for gastric acid hypersecretion in children with cystinosis. Pediatr Nephrol. 2005;20(12):1786-93.

10. Emma F, Hoff WV, Hohenfellner K, Topaloglu R, Greco M, Ariceta G, et al. An international cohort study spanning five decades assessed outcomes of nephropathic cystinosis. Kidney Int. 2021;100(5):1112-23.

11. Gahl WA, Balog JZ, Kleta R. Nephropathic cystinosis in adults: natural history and effects of oral cysteamine therapy. Ann Intern Med. 2007;147(4):242-50.

12. Gahl WA, Bernardini IM, Dalakas MC, Markello TC, Krasnewich DM, Charnas LR. Muscle carnitine repletion by long-term carnitine supplementation in nephropathic cystinosis. Pediatr Res. 1993;34(2):115-9.

13. Gahl WA, Reed GF, Thoene JG, Schulman JD, Rizzo WB, Jonas AJ, et al. Cysteamine therapy for children with nephropathic cystinosis. N Engl J Med. 1987;316(16):971-7.

14. Gaillard S, Roche L, Lemoine S, Deschenes G, Morin D, Vianey-Saban C, et al. Adherence to cysteamine in nephropathic cystinosis: A unique electronic monitoring experience for a better understanding. A prospective cohort study: CrYSTobs. Pediatr Nephrol. 2021;36(3):581-9.

15. Greco M, Brugnara M, Zaffanello M, Taranta A, Pastore A, Emma F. Long-term outcome of nephropathic cystinosis: a 20-year single-center experience. Pediatr Nephrol. 2010;25(12):2459-67.

16. Haase M, Morgera S, Bamberg C, Halle H, Martini S, Dragun D, et al. Successful pregnancies in dialysis patients including those suffering from cystinosis and familial Mediterranean fever. Journal of Nephrology. 2006;19(5):677-81.

17. Hamed DH, Halim RMA, El Attar MM, Soliman NA, Osman HM. Pulmonary dysfunction in children with Cystinosis: single center study, original article. Egyptian Pediatric Association Gazette. 2022;70(1).

18. Hohenfellner K, Niesl C, Haffner D, Oh J, Okorn C, Palm K, et al. Beneficial effects of starting oral cysteamine treatment in the first 2 months of life on glomerular and tubular kidney function in infantile nephropathic cystinosis. Mol Genet Metab. 2022;136(4):282-8.

19. Iwata F, Kuehl EM, Reed GF, McCain LM, Gahl WA, Kaiser-Kupfer MI. A randomized clinical trial of topical cysteamine disulfide (cystamine) versus free thiol (cysteamine) in the treatment of corneal cystine crystals in cystinosis. Mol Genet Metab. 1998;64(4):237-42.

20. Iyob-Tessema H, Wang CS, Kennedy S, Reyes L, Shin S, Greenbaum LA, et al. Grip Strength in Adults and Children with Cystinosis. KI Rep. 2021;6(2):389-95.

21. Kaiser-Kupfer MI, Gazzo MA, Datiles MB, Caruso RC, Kuehl EM, Gahl WA. A randomized placebo-controlled trial of cysteamine eye drops in nephropathic cystinosis. Arch Ophthalmol. 1990;108(5):689-93.

22. Kaiser-Kupfer MI, Fujikawa L, Kuwabara T, Jain S, Gahl WA. Removal of corneal crystals by topical cysteamine in nephropathic cystinosis. N Engl J Med. 1987;316(13):775-9.

23. Kimonis VE, Troendle J, Rose SR, Yang ML, Markello TC, Gahl WA. Effects of early cysteamine therapy on thyroid function and growth in nephropathic cystinosis. J Clin Endocrinol Metab. 1995;80(11):3257-61.

24. Kuczborska K, Gozdowska J, Lewandowska D, Grenda R, Galazka Z, Nazarewski S, et al. Therapeutic Problems and Pregnancy in a Patient With Infantile Nephropathic Cystinosis: A Case Report. Transplant Proc. 2019;51(2):545-7.

25. Labbe A, Niaudet P, Loirat C, Charbit M, Guest G, Baudouin C. In vivo confocal microscopy and anterior segment optical coherence tomography analysis of the cornea in nephropathic cystinosis. Ophthalmology. 2009;116(5):870-6.

26. Labbe A, Baudouin C, Deschenes G, Loirat C, Charbit M, Guest G, et al. A new gel formulation of topical cysteamine for the treatment of corneal cystine crystals in cystinosis: the Cystadrops OCT-1 study. Mol Genet Metab. 2014;111(3):314-20.

27. Langman CB, Greenbaum LA, Sarwal M, Grimm P, Niaudet P, Deschenes G, et al. A randomized controlled crossover trial with delayed-release cysteamine bitartrate in nephropathic cystinosis: effectiveness on white blood cell cystine levels and comparison of safety. Clin J Am Soc Nephrol. 2012;7(7):1112-20.

28. Langman CB, Greenbaum LA, Grimm P, Sarwal M, Niaudet P, Deschenes G, et al. Quality of life is improved and kidney function preserved in patients with nephropathic cystinosis treated for 2 years with delayed-release cysteamine bitartrate. J Pediatr. 2014;165(3):528-33.e1.

29. Levtchenko E, Blom H, Wilmer M, van den Heuvel L, Monnens L. ACE inhibitorenalapril diminishes albuminuria in patients with cystinosis. Clin Nephrol. 2003;60(6):386-9.

30. Liang H, Labbe A, Le Mouhaer J, Plisson C, Baudouin C. A New Viscous Cysteamine Eye Drops Treatment for Ophthalmic Cystinosis: An Open-Label Randomized Comparative Phase III Pivotal Study. Invest Ophthalmol Vis Sci. 2017;58(4):2275-83.

31. Liang H, Baudouin C, Tahiri Joutei Hassani R, Brignole-Baudouin F, Labbe A. Photophobia and corneal crystal density in nephropathic cystinosis: an in vivo confocal microscopy and anterior-segment optical coherence tomography study. Invest Ophthalmol Vis Sci. 2015;56(5):3218-25.

32. MacDonald IM, Noel LP, Mintsioulis G, Clarke WN. The effect of topical cysteamine drops on reducing crystal formation within the cornea of patients affected by nephropathic cystinosis. J Pediatr Ophthalmol Strabismus. 1990;27(5):272-4.

33. Markello TC, Bernardini IM, Gahl WA. Improved renal function in children with cystinosis treated with cysteamine. N Engl J Med. 1993;328(16):1157-62.

34. Nesterova G, Williams C, Bernardini I, Gahl WA. Cystinosis: renal glomerular and renal tubular function in relation to compliance with cystine-depleting therapy. Pediatr Nephrol. 2015;30(6):945-51.

35. Niesl C, Boulesteix AL, Oh J, Palm K, Schlingmann P, Wygoda S, et al. Relationship between age at initiation of cysteamine treatment, adherence with therapy, and glomerular kidney function in infantile nephropathic cystinosis. Mol Genet Metab. 2022;136(4):268-73.

36. O'Connell N, Oh J, Arbeiter K, Buscher A, Haffner D, Kaufeld J, et al. Patients With Infantile Nephropathic Cystinosis in Germany and Austria: A Retrospective Cohort Study. Front Med (Lausanne). 2022;9:864554.

37. Ozdemir HB, Ozmen MC, Aktas Z, Hasanreisoglu M. In vivo confocal microscopy and anterior segment optical coherence tomography follow-up of cysteamine treatment in corneal cystinosis. Indian J Ophthalmol. 2019;67(1):153-5.

38. Quinaux T, Bertholet-Thomas A, Servais A, Boyer O, Vrillon I, Hogan J, et al. Response to Cysteamine in Osteoclasts Obtained from Patients with Nephropathic Cystinosis: A Genotype/Phenotype Correlation. Cells. 2021;10(9):21.

39. Ramappa AJ, Pyatt JR. Pregnancy-associated cardiomyopathy occurring in a young patient with nephropathic cystinosis. Cardiol Young. 2010;20(2):220-2.

40. Reiss RE, Kuwabara T, Smith ML, Gahl WA. Successful pregnancy despite placental cystine crystals in a woman with nephropathic cystinosis. N Engl J Med. 1988;319(4):223-6.

41. Robertson G, McCulloch M, Wearne N, Jones E, Barday Z, Blumenthal A, et al. A Successful Adolescent Pregnancy in a Patient With Cystinosis and CKD Not Yet on Kidney Replacement Therapy. KI Rep. 2022;7(7):1711-5.

42. Sadjadi R, Sullivan S, Grant N, Thomas SE, Doyle M, Hammond C, et al. Clinical trial readiness study of distal myopathy and dysphagia in nephropathic cystinosis. Muscle Nerve. 2020;62(6):681-7.

43. Servais A, Janssen MCH, Blakey H, Greco M, Lemoine S, Martin-Moreno PL, et al. Pregnancy in cystinosis patients with chronic kidney disease: A European case series. J Inherit Metab Dis. 2022;45(5):963-8.

44. Sonies BC, Almajid P, Kleta R, Bernardini I, Gahl WA. Swallowing dysfunction in 101 patients with nephropathic cystinosis: benefit of long-term cysteamine therapy. Medicine (Baltimore). 2005;84(3):137-46.

45. Spicer RA, Clayton PA, McTaggart SJ, Zhang GY, Alexander SI. Patient and graft survival following kidney transplantation in recipients with cystinosis: a cohort study. Am J Kidney Dis. 2015;65(1):172-3.

46. Tsilou ET, Thompson D, Lindblad AS, Reed GF, Rubin B, Gahl W, et al. A multicentre randomised double masked clinical trial of a new formulation of topical cysteamine for the treatment of corneal cystine crystals in cystinosis. Br J Ophthalmol. 2003;87(1):28-31.

47. Tsilou ET, Rubin BI, Reed G, Caruso RC, Iwata F, Balog J, et al. Nephropathic cystinosis: posterior segment manifestations and effects of cysteamine therapy. Ophthalmology. 2006;113(6):1002-9.

48. Vaisbich MH, Koch VH. Report of a Brazilian multicenter study on nephropathic cystinosis. Nephron. 2010;114(1):c12-8.

49. Vaisbich MH, Caires Ferreira J, Price H, Young KD, Sile S, Checani G, et al. Cysteamine bitartrate delayed-release capsules control leukocyte cystine levels and promote statural growth and kidney health in an open-label study of treatment-naive patients <6 years of age with nephropathic cystinosis. JIMD rep. 2022;63(1):66-79.

50. van't Hoff WG, Gretz N. The treatment of cystinosis with cysteamine and phosphocysteamine in the United Kingdom and Eire. Pediatr Nephrol. 1995;9(6):685-9.

51. van Stein C, Klank S, Gruneberg M, Ottolenghi C, Grebe J, Reunert J, et al. A comparison of immediate release and delayed release cysteamine in 17 patients with nephropathic cystinosis. Orphanet J Rare Dis. 2021;16(1):387.

52. Veys K, Zadora W, Hohenfellner K, Bockenhauer D, Janssen MCH, Niaudet P, et al. Outcome of infantile nephropathic cystinosis depends on early intervention, not genotype: A multicenter sibling cohort study. J Inherit Metab Dis. 2023;46(1):43-54.

53. Viltz L, Trauner DA. Effect of age at treatment on cognitive performance in patients with cystinosis. J Pediatr. 2013;163(2):489-92.

54. Dawson S, Girling CJ, Cowap L, Clark-Carter D. Psychological interventions for improving adherence to inhaled therapies in people with cystic fibrosis. Cochrane Database Syst Rev. 2023;3:CD013766.

55. Martin K, Geuens S, Asche JK, Bodan R, Browne F, Downe A, et al. Psychosocial recommendations for the care of children and adults with epidermolysis bullosa and their family: evidence based guidelines. Orphanet J Rare Dis. 2019;14(1):133.

56. Ponce OJ, Spencer-Bonilla G, Alvarez-Villalobos N, Serrano V, Singh-Ospina N, Rodriguez-Gutierrez R, et al. The efficacy and adverse events of testosterone replacement therapy in hypogonadal men: A systematic review and meta-analysis of randomized, placebo-controlled trials. J Clin Endocrinol Metab. 2018;17:17.
